# Axial dysprosium cyclopentadienyl-amide single-molecule magnets with hysteresis up to 92 kelvin

**DOI:** 10.1038/s41467-026-77104-z

**Published:** 2026-08-26

**Authors:** Jack Emerson-King, Benjamin E. Atkinson, William J. A. Blackmore, Jacinta Rees, Sophie C. Corner, Jack Baldwin, Nicholas F. Chilton, David P. Mills

**Affiliations:** 1 Department of Chemistry, The University of Manchester; Oxford Road, Manchester, UK; 2 Research School of Chemistry, The Australian National University; Sullivans Creek Road, Canberra, ACT Australia

**Keywords:** Synthetic chemistry methodology, Magnetic properties and materials, Ligands

## Abstract

Molecules showing magnetic memory effects at high temperatures could be used in high-density data storage devices. A magnetic hysteresis temperature (*T*_H_) of 100 K has been achieved for a near-linear dysprosium bis(amide)-alkene single-molecule magnet (SMM), and a bent dysprosium cyclopentadienyl-amide SMM has shown *T*_H_ = 73 K. However, both these complexes exhibit fast magnetic relaxation at lower temperatures compared to dysprosium bis(cyclopentadienyl) SMMs. Here we report the synthesis of two highly axial dysprosium cyclopentadienyl-amide SMMs, [Dy{N(Si^i^Pr_3_)_2_}(Cp^R^)][Al{OC(CF_3_)_3_}_4_] (**1-Dy**, Cp^R^ = Cp^ttt^, C_5_H_2_^t^Bu_3_−1,2,4; **2-Dy**, Cp^R^ = Cpʹʹʹ, C_5_H_2_(SiMe_3_)_3_−1,2,4). We find *T*_H_ = 91 K for **1-Dy** and 92 K for **2-Dy**; ab initio calculations on **1-Dy** show that magnetic relaxation is controlled by a combination of short dysprosium-ligand distances, a near-linear N–Dy–Cpʹʹʹ_centroid_ angle, and constrained vibrational modes. Together, this work demonstrates that dysprosium SMMs with large *T*_H_ values can be achieved with a judicious combination of π-aromatic and σ-donor ligands.

## Abstract (web summary)

Molecules showing magnetic memory effects at high temperatures could be used in high-density data storage devices but often exhibit fast magnetic relaxation at lower temperatures. Here the authors report the synthesis of two highly axial dysprosium cyclopentadienyl-amide single molecule magnets with magnetic hysteresis temperatures of 91 K and 92 K.

## Introduction

Magnetic memory effects have been observed in both lanthanide atoms^1,2^ and molecules^3^, providing promising candidates for high-density data storage technologies^4^. Single-molecule magnets (SMMs) have been known for over thirty years^5^, and since 2011 lanthanide SMMs have provided the highest magnetic hysteresis temperatures (*T*_H_, open hysteresis loops at zero-field for a magnetic field sweep rate of *ca*. 20 Oe/s)^6^. Lanthanide SMMs with carefully designed crystal field (CF) splitting have huge effective energy barriers to magnetic reversal (*U*_eff_), which can deliver higher *T*_H_ and *T*_B_ (the temperature at which the magnetic relaxation rate is 100 s) values if magnetic relaxation mechanisms are sufficiently controlled^7–11^. For dysprosium SMMs a linear geometry is most effective in stabilising magnetic states with the largest |*m*_*J*_| projections, while destabilising those with the smallest |*m*_*J*_| projections. Deviations from linearity or equatorial ligand coordination introduce transverse components in the CF that mix *m*_*J*_ states, diminishing *U*_eff_, and to a greater extent *T*_H_ and *T*_B_^4,12,13^.

In 2017 the first dysprosocenium ([Dy(Cp^R^)_2_]^+^, Cp^R^ = C_5_R_5_) SMM [Dy(Cp^ttt^)_2_][B(C_6_F_5_)_4_] (herein referred to as **[Dy(Cp**^**ttt**^**)**_**2**_**]**^**+**^, Cp^ttt^ = C_5_H_2_^t^Bu_3_−1,2,4) was shown to have *U*_eff_ = 1780(13) K, *T*_H_ = 60 K and *T*_B_ = 53 K, due to a combination of a strong axial CF and rigid Cp^ttt^ ligands that substantially reduced two-phonon Raman relaxation^14–16^. In the interim, analogous axial dysprosium complexes with two π-aromatic ligands have shown similar properties^12,13^, with the leading example [Dy(Cp^iPr5^)(Cp*)][B(C_6_F_5_)_4_] (herein referred to as **[Dy(Cp**^**iPr5**^)(Cp*)]^**+**^, Cp^iPr5^ = C_5_^i^Pr_5_; Cp* = C_5_Me_5_) having *U*_eff_ = 2230(10) K, *T*_H_ = 80 K and *T*_B_ = 65 K, due to its large Cp^iPr5^–Dy–Cp* angle (162.51(2)°^17,18^). A related SMM, [Dy_2_(Cp^iPr5^)_2_(μ-I)_3_], has *U*_eff_ = 2345(12) K, *T*_H_ = 80 K and *T*_B_ = 72 K, but it sits in a slightly different class due to the strong exchange coupling arising from a 1 e^–^ Dy–Dy bond^19^. In 2025 the dysprosium bis(amide)-alkene SMM [Dy{N(Si^i^Pr_3_)[Si(^i^Pr)_2_C(CH_3_) = CHCH_3_]}{N(Si^i^Pr_3_)(Si^i^Pr_2_Et)}][Al{OC(CF_3_)_3_}_4_] (herein referred to as **[Dy(amide)(amide-alkene)]**^**+**^) set new record *U*_eff_ = 2652(16) K and *T*_H_ = 100 K as it shows N–Dy–N angles up to 165.3(8)° and Dy–N distances as short as 2.166(12) Å, but its *T*_B_ could not be extracted as at 2 K full relaxation takes less than 100 s; this was ascribed to the flexible bis-amide ligand environment promoting effective under-barrier relaxation processes^20^. Also in 2025, the dysprosium cyclopentadienyl-amide SMM [Dy{N(Si^i^Pr_3_)_2_}(Cp*)][Al{OC(CF_3_)_3_}_4_] (herein referred to as **[Dy{N(Si**^**i**^**Pr**_**3**_**)**_**2**_}(Cp*)]^**+**^) was shown to have a high *U*_eff_ (2192(33) K), but its *T*_H_ is limited to 73 K and *T*_B_ was not defined; fast magnetic relaxation was attributed to both the presence of a flexible amide ligand and bent N–Dy–Cp*_centroid_ angle (*ca*. 152.5(2)°) giving excited states with mixed *m*_*J*_ contributions^21^.

As dysprosium bis(cyclopentadienyl) (e.g., **[Dy(Cp**^**iPr5**^)(Cp*)]^**+**^^17^) and bis(amide) (e.g., **[Dy(amide)(amide-alkene)]**^**+**^^20^) SMMs with respective Cp^R^_centroid_–Dy–Cp^R^_centroid_ and N–Dy–N angles closest to linearity have provided the best SMMs of their class, we reasoned that improved performance could be obtained by targeting dysprosium cyclopentadienyl-amide SMMs that are less bent than **[Dy{N(Si**^**i**^**Pr**_**3**_**)**_**2**_}(Cp*)]^**+**^^21^. Coordinatively unsaturated lanthanide complexes tend to deviate from regular geometries due to their predominantly ionic, non-directional bonding regimes^22,23^. We envisaged that high-performance dysprosium SMMs with near-linear N–Dy–Cp^R^_centroid_ angles could be achieved by fine-tuning the steric complementarity of the two ligands.

## Results and discussion

### Synthesis

**Fig. 1:**
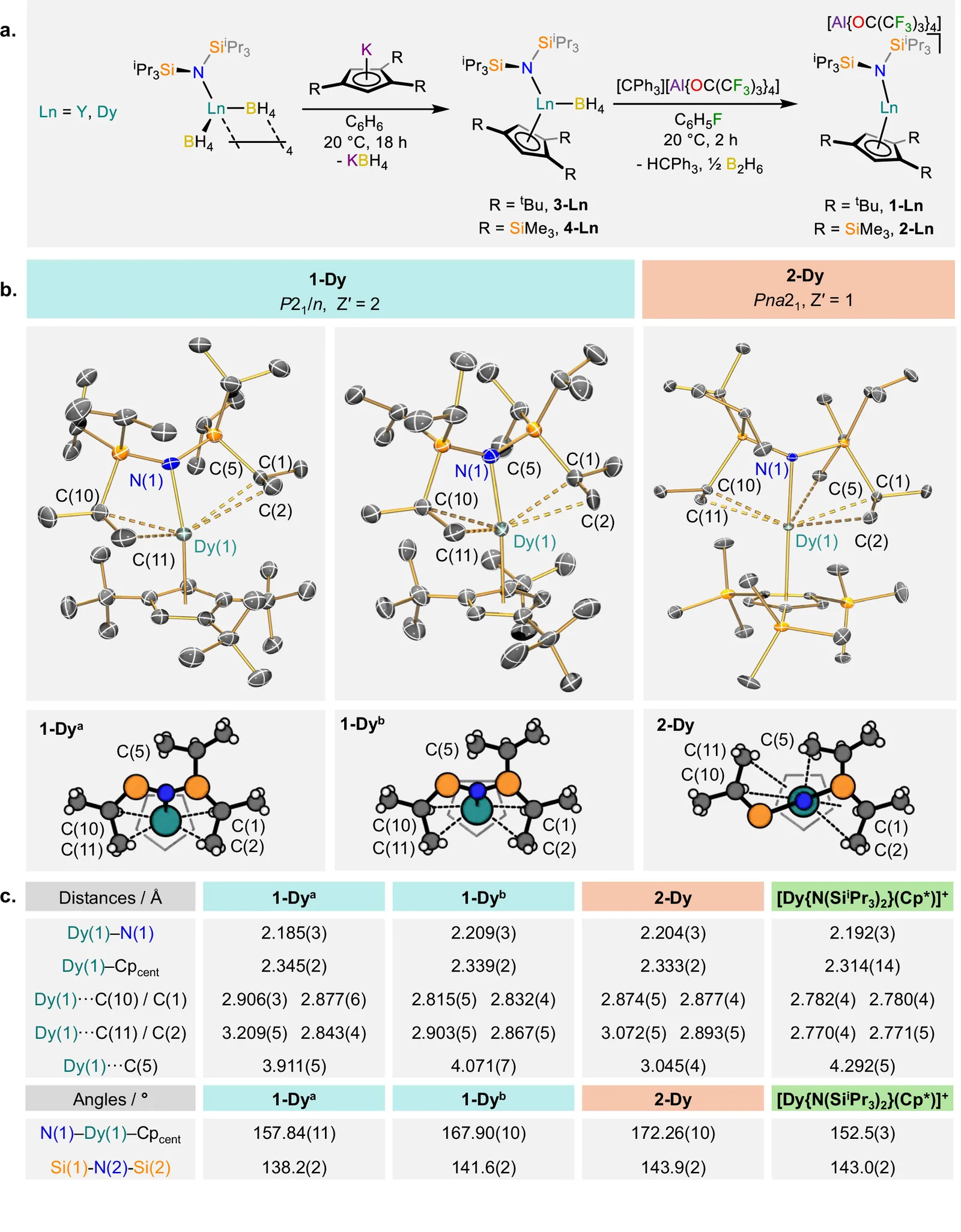
Synthesis of complexes 1-Ln, 2-Ln, 3-Ln and 4-Ln, and selected single crystal XRD data for the cations of 1-Dy, 2-Dy and [Dy{N(Si^i^Pr_3_)_2_}(Cp*)]^+^. **a** Synthetic route to complexes **1-Ln,** **2-Ln,** **3-Ln** and **4-Ln** (Ln = Dy, Y). **b** Single crystal XRD structures (displacement ellipsoids set at 50%) of the cations of **1-Dy** (Z′ = 2; **1-Dy**^**a**^ and **1-Dy**^**b**^) and **2-Dy** at 100 K, with hydrogen atoms omitted and key atoms labelled, together with top-down schematics of the cations of **1-Dy**^**a**^, **1-Dy**^**b**^ and **2-Dy**, illustrating the contrasting equatorial coordination environment (Dy = cyan, Si = orange, N = blue, C = grey, H = white). **c** Key metrics from single crystal XRD structures for **1-Dy**^**a**^, **1-Dy**^**b**^, **2-Dy** and **[Dy{N**(**Si**^**i**^**Pr**_**3**_)_**2**_**}**(Cp*)**]**^**+**^^21^, the number in brackets represents 1 estimated standard deviation (esd).

The lanthanide cyclopentadienyl-amide complexes, [Ln{N(Si^i^Pr_3_)_2_}(Cp^R^)][Al{OC(CF_3_)_3_}_4_] (**1-Ln**, Ln = Y, Dy, Cp^R^ = Cp^ttt^; **2-Ln**, Ln = Y, Dy, Cp^R^ = Cpʹʹʹ, C_5_H_2_(SiMe_3_)_3_−1,2,4), were synthesised in 37–48% yields by hydride abstraction reactions of their corresponding precursors [Ln{N(Si^i^Pr_3_)_2_}(Cp^R^)(BH_4_)] (**3-Ln**, Ln = Y, Dy, Cp^R^ = Cp^ttt^; **4-Ln**, Ln = Y, Dy, Cp^R^ = Cpʹʹʹ) with [CPh_3_][Al{OC(CF_3_)_3_}_4_]^24^ in fluorobenzene at ambient temperature for 2 h, followed by recrystallisation from fluorobenzene solutions layered with *n*-hexane (Fig. 1a). Complexes **3-Ln** and **4-Ln** were themselves prepared by salt metathesis reactions of [Ln{N(Si^i^Pr_3_)_2_}(µ-BH_4_)_2_]_4_^25^ with the respective KCp^R^ salt^26,27^ in benzene at ambient temperature for 18 h, followed by extraction into *n*-hexane and filtration to remove the KBH_4_ byproduct. Recrystallisation from saturated *n*-hexane solutions at –35 °C gave **3-Ln** and **4-Ln** in 62–83% crystalline yields.

### Single crystal X-ray diffraction (XRD)

XRD data were collected on single crystals of **1-4-Ln** (see Supplementary Information Figures S1–S10 and Tables S1–S4). The structures of **3-Ln** and **4-Ln** are similar to those of [Ln{N(Si^i^Pr_3_)_2_}(Cp*)(BH_4_)] (Ln = Y, Dy)^21^, though unusually we observe that **3-Dy** and **3-Y** are not isomorphic, crystallising in *C*2/*c* and *P*2_1_/*n* space groups, respectively. In the solid-state the [Ln{N(Si^i^Pr_3_)_2_}(Cp^R^)]^+^ cations and [Al{OC(CF_3_)_3_}_4_]^–^ anions of **1-Ln** and **2-Ln** are fully separated. The yttrium complexes **1-Y** and **2-Y** are isomorphic and isostructural with their respective dysprosium counterparts, so for brevity we focus our discussion on the cations of **1-Dy** and **2-Dy** (Fig. 1b). There are two unique cations in the monoclinic (*P*2_1_/*n*) unit cell of **1-Dy** with distinct geometries (**1-Dy**^**a**^ and **1-Dy**^**b**^), and one unique cation in the orthorhombic (*Pna*2_1_) cell of **2-Dy**. Key metrical parameters for all three cations, alongside the similar cation of **[Dy{N(Si**^**i**^**Pr**_**3**_**)**_**2**_}(Cp*)]^**+**^^21^, are collated in Fig. 1c (See Supplementary Information Table S1–S2 for further metrics of all complexes).

All the cations of **1-Dy**^**a**^, **1-Dy**^**b**^, **2-Dy** and **[Dy{N(Si**^**i**^**Pr**_**3**_**)**_**2**_}(Cp*)]^**+**^^21^ have piano stool motifs with opposing {N(Si^i^Pr_3_)_2_} and Cp^R^ ligands, flanked equatorially by isopropyl groups of the bis(silyl)amide ligands. The cation of **2-Dy** has the greatest N–Dy–Cp^R^_centroid_ angle (172.26(10)°), and **[Dy{N(Si**^**i**^**Pr**_**3**_**)**_**2**_}(Cp*)]^**+**^ the smallest (152.5(3)°)^21^. The two cations of **1-Dy** show significantly different N–Dy–Cp^ttt^_centroid_ angles between these extremes (**1-Dy**^**a**^: 157.84(11)°; **1-Dy**^**b**^: 167.90(10)°). The Dy–N (2.204(3) Å) and Dy–Cp^R^_centroid_ (2.332(2) Å) distances of **2-Dy** are within error of the more linear cation of **1-Dy** (**1-Dy**^**b**^: Dy–N: 2.209(3) Å; Dy–Cp^R^_centroid_: 2.339(2) Å), and these are longer than the corresponding distances in **[Dy{N(Si**^**i**^**Pr**_**3**_**)**_**2**_}(Cp*)]^**+**^ (Dy–N: 2.192(3) Å; Dy–Cp^R^_centroid_: 2.314(14) Å)^21^; the Dy–Cp^ttt^_centroid_ distance of the more bent cation of **1-Dy**^**a**^ (2.345(2) Å) is statistically equivalent to the more linear cation, but it has a shorter Dy–N distance (2.185(3) Å).

The greater linearity of the cation of **2-Dy** over both those of **1-Dy** corresponds with a conformational difference in the bis(silyl)amide isopropyl groups (Fig. 1b). The cations of **1-Dy** exhibit the same pincer-like motif as **[Dy{N(Si**^**i**^**Pr**_**3**_**)**_**2**_}(Cp*)]^**+**^^21^, with the two isopropyl groups straddling the dysprosium centre in a mirror-symmetric fashion. The resultant Dy···C distances of **1-Dy**^**a**^ and **1-Dy**^**b**^ between 2.815(5) and 3.209(5) Å engage in close electrostatic agostic-type interactions between methine C(1) and C(10) and methyl C(2) and C(11) C–H bonds to the Dy(III) ion, each in an η^2^-fashion. By contrast, in **2-Dy** the two flanking isopropyl groups are orientated in opposing directions on opposite sides of the Dy(III) ion and show Dy···C distances in a narrower range (2.874(5)–3.072(5) Å). An additional proximal C(5) methyl group provides an extra η^3^-H–C–H electrostatic interaction, 3.045(4) Å distant, giving a more saturated equatorial environment. The corresponding C(5) methyl group in **[Dy{N(Si**^**i**^**Pr**_**3**_**)**_**2**_}(Cp*)]^**+**^^21^ and both cations of **1-Dy** are found in similar vicinities, but remain remote from the dysprosium centres (Dy···C > 3.91 Å).

For both **1-Dy** and **2-Dy** the bulkier 1,2,4-substituted Cp^R^ ligands give more linear metal coordination geometries than seen in **[Dy{N(Si**^**i**^**Pr**_**3**_**)**_**2**_}(Cp*)]^**+**^^21^, which we attribute to the complementarity of the steric bulk of Cp^ttt^ and Cp′′′ to that of the bis(silyl)amide ligand. The C_ring_–SiMe_3_ bonds of **2-Dy** (1.888(4)–1.892(4) Å) are longer than the C_ring_–C^t^Bu bonds in **1-Dy**^**a**^ and **1-Dy**^**b**^ (1.524(7)–1.550(7) Å), which potentially enables the distinctive conformation of the {N{Si^i^Pr_3_)_2_} isopropyl groups in the cation of **2-Dy**. For all cations of **1-Dy** and **2-Dy** the combined ligand periphery fully encapsulates the dysprosium centre. This was quantified by the *AtomAccess* code^28^, which uses the solid angle (SA) metric to assess the exposed surface area of a complexed ion (see Supplementary Information Figures S11–S13). A total 3.9% SA of the Dy(III) ion is visible in **2-Dy**, with the largest potential binding site being only 1.6% SA. The two Dy(III) ions in **1-Dy** have larger total SA (both 6.2%) and SA of the largest binding sites (both 2.8%), but these values are still lower than those seen for the relatively unencumbered **[Dy{N(Si**^**i**^**Pr**_**3**_**)**_**2**_}(Cp*)]^**+**^ (mean total SA: 10.0%, mean largest binding site SA: 6.6%)^21^.

### Bulk characterisation data

Powder XRD data were collected on all complexes. Le Bail analysis^29^ of the diffractograms obtained closely matched the single crystal XRD parameters and showed the absence of any other crystalline phase (see Supplementary Information Figures S14–S21). The ATR-IR spectra (see Supplementary Information Figures S22–S33) of **1-Ln,** **2-Ln** and **4-Ln** exhibit superimposable absorption bands for each Y/Dy pair, while the **3-Ln** pair show very similar spectra with slight deviations, especially at low frequency, consistent with our observation of the differing crystal phases exhibited by these two compounds. Absorptions corresponding to vibrational modes of the [Al{OC(CF_3_)_3_}_4_]^–^ anions dominate the ATR-IR spectra of **1-Ln** and **2-Ln**, but strong electrostatic interactions of several methine and methyl groups of {N(Si^i^Pr_3_)_2_} ligands with the coordinatively unsaturated Ln(III) cations is evidenced by the presence of lower-frequency C–H stretching modes between 2800 and 2580 cm^−1^. The ATR-IR spectra of **3-Ln** and **4-Ln** exhibit characteristic resonances for κ^3^-BH_4_ groups at ~2500 cm^−1^ (terminal B–H) and between 2300–2100 cm^–1^ (Ln-bound B–H)^30^, alongside the distinctive agostic-type C–H stretch at ~ 2790 cm^−1^ for all four complexes. Elemental analysis results obtained for **1-4-Ln** are in not always in close agreement with the expected values, but this is consistent with standard issues associated with this form of analysis^31^, which is exacerbated by fluorine-rich compounds such as **1-Ln** and **2-Ln**^32^.

### Solution characterisation data

Multinuclear NMR spectroscopy was performed on fluorobenzene solutions of **1-Ln** and **2-Ln**, and on *d*_6_-benzene solutions of **3-Ln** and **4-Ln** (see Supplementary Information Figures S34–S63). The dysprosium complexes **3-Dy** and **4-Dy** exhibit paramagnetically broadened and shifted signals in their solution ^1^H NMR spectra, while **1-Dy** and **2-Dy** appear entirely featureless between 200 and –200 ppm, save for solvent resonances. With all four dysprosium complexes the ^11^B, ^13^C{^1^H} and ^29^Si NMR spectra obtained are similarly featureless apart from the ^13^C solvent resonances, consistent with our previous report of the Cp*-ligated analogues of these complexes^21^. A broad resonance for the [Al{OC(CF_3_)_3_}_4_]^–^ counteranion is observed in the ^19^F NMR spectra at –94.8 ppm and –90.4 ppm for **1-Dy** and **2-Dy**, respectively, alongside a highly paramagnetically broadened and shifted fluorobenzene solvent resonance, at –110.9 and –108.8 ppm, respectively (*cf*. 113.1 ppm for unshifted fluorobenzene). Conversely, the ^1^H, ^13^C{^1^H}, ^19^F and ^11^B NMR spectra of the yttrium analogues **1-4-Y** are unambiguously assignable, in accord with the proposed structures, and are comparable to closely related complexes^20,21,25^.

### Magnetometry

**Fig. 2:**
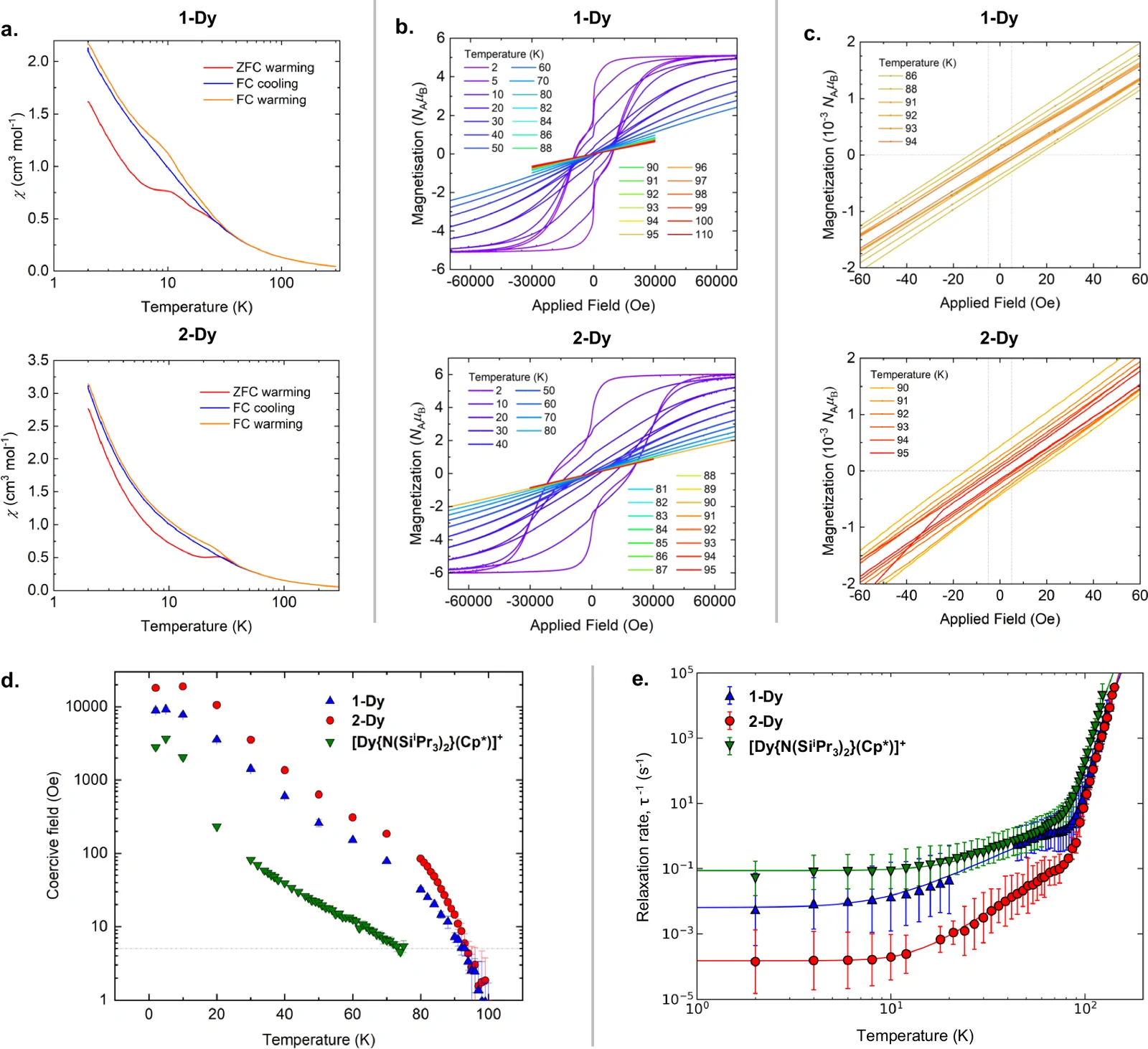
Selected magnetic data for complexes 1-Dy, 2-Dy and [Dy{N(Si^i^Pr_3_)_2_}(Cp*)]^+^. **a** Zero-field cooled and field cooled curves of **1-Dy** (top) and **2-Dy** (bottom) performed at 0.1 T. **b** M *vs*. H hysteresis loops at 22 Oe s^−1^ for **1-Dy** between 2 and 110 K (top) and **2-Dy** between 2 and 95 K (bottom). **c** Enlargement of the closing 22 Oe s^−1^ hysteresis loops for **1-Dy** between 86 and 94 K (top) and **2-Dy** between 90 and 95 K (bottom). **d** Coercive field (*H*_c_) as a function of temperature for **1-Dy,** **2-Dy** and **[Dy{N**(**Si**^**i**^**Pr**_**3**_)_**2**_**}**(Cp*)**]**^**+**^^21^ as extracted from hysteresis measurements; closed hysteresis is determined to be *H*_c_ < 5 Oe (grey dashed line, field calibrated with a Pd standard). **e** Zero-field relaxation rates of the main relaxation processes in **1-Dy,** **2-Dy** and **[Dy{N**(**Si**^**i**^**Pr**_**3**_)_**2**_**}**(Cp*)**]**^**+**^^21^ fitted to Orbach, Raman and QTM contributions; for **1-Dy,** *U*_eff_ = 2466(35) K, *A* = –11.97(13) log_10_[s], *R* = –4.73(15) log_10_[s^–1^ K^–*n*^], *n* = 2.66(8) and *Q* = 2.19(6) log_10_[s]. Bars represent 1 esd of the distribution of rates; for **2-Dy,** *U*_eff_ = 2743(28) K, *A* = –12.9(1) log_10_[s], *R* = –8.2(2) log_10_[s^–1^ K^–*n*^], *n* = 3^.^9(1) a*n*d *Q* = 3.83(1) log_10_[s]; for **[Dy{N**(**Si**^**i**^**Pr**_**3**_)_**2**_**}**(Cp*)**]**^**+**^^21^, *U*_eff_ = 2191(33) K, *A* = –11.8(1) log_10_[s], *R* = –5.2(2) log_10_[s^–1^ K^–*n*^], *n* = 3^.^1(1) a*n*d *Q* = 1.06(4) log_10_[s].

Magnetic data were collected on microcrystalline samples of **1-Dy** and **2-Dy** using a superconducting quantum interference device (SQUID). The zero-field-cooled (ZFC) and field-cooled (FC) magnetic susceptibility data diverge at 30 K for **1-Dy** and 40 K for **2-Dy** (Fig. 2a and Supplementary Information Figures S64 and S74), which we attribute to the onset of magnetic blocking^33^. We probed the magnetic reversal dynamics of **1-Dy** and **2-Dy** at low temperatures by DC waveform^34^ and DC decay methods^35^, and at higher temperatures under zero DC field by alternating current (AC) measurements (Fig. 2e and Supplementary Information Figures S65–S73 and S75–S85). The Arrhenius law [*τ*^–1^ = 10^–*A*^*exp*(–*U*_eff_/*k*_B_*T*)] is followed for magnetisation reversal rates above 90 K for **1-Dy** and 85 K for **2-Dy**, with *U*_eff_ = 2466(35) K and *A* = –11.97(13) log_10_(s) for **1-Dy**, and *U*_eff_ = 2743(28) K and *A* = –12.9(1) log_10_(s) for **2-Dy**; this is in accord with Orbach relaxation proceeding by concatenated one-phonon transitions. The experimentally determined *U*_eff_ values of **1-Dy** and **2-Dy** are in good agreement with those predicted by SA-CASSCF-SO (see below), and are larger than those measured for **[Dy(Cp**^**iPr5**^)(Cp*)]^**+**^ (2230(10) K)^17,18^ and **[Dy{N(Si**^**i**^**Pr**_**3**_**)**_**2**_}(Cp*)]^**+**^ (2191(33) K)^21^, and similar to that for **[Dy(amide)(amide-alkene)]**^**+**^ (2652(16) K^20^). The magnetisation reversal rates between 10 and 90 K for **1-Dy** and between 12 and 85 K for **2-Dy** have a power-law dependence (*τ*^–1^ = 10^*R*^*T*^*n*^) that suggests relaxation by a two-phonon Raman scattering processes, with *R* = –8.2 (1) log_10_(s^–1^ K^–*n*^) and *n* = 3.9(1) for **1-Dy** and *R* = –4.73(15) log_10_(s^–1^ K^–*n*^) and *n* = 2.66(8) for **2-Dy**. Below 10 K (**1-Dy**) or 12 K (**2-Dy**) the rates become increasingly less dependent on temperature, which is associated with quantum tunnelling of the magnetisation (QTM; *τ*^–1^ = 10^–*Q*^), giving fitted rate constants of Q = 2.19(6) log_10_(s) (**1-Dy**) and Q = 3.83(1) log_10_(s) (**2-Dy**). Unlike **[Dy(amide)(amide-alkene)]**^**+**^^20^ and **[Dy{N(Si**^**i**^**Pr**_**3**_**)**_**2**_}(Cp*)]^**+**^^21^, we were able to determine *T*_B_ of 7.2 K for **1-Dy** and 39.5 K for **2-Dy**; these values are both lower than those of **[Dy(Cp**^**iPr5**^)(Cp*)]^**+**^ (65 K)^17^ and the current record 72 K for [Dy_2_(Cp^iPr5^)_2_(μ-I)_3_]^19^. Complexes **1-Dy** and **2-Dy** each show a second, much weaker signal, of a faster relaxation process below 20 K, which appear to have Raman-like temperature dependence (Figures S73 and S85); these could either be due to minor impurities or higher-order relaxation modes.

The variable temperature magnetic hysteresis properties of **1-Dy** and **2-Dy** were determined under DC fields at sweep rates of 22 Oe/s (Fig. 2b–d). The large zero-field steps at low temperatures are consistent with fast magnetisation reversal by QTM, as indicated in the AC data^33^. At 2 K we find coercive fields (*H*_C_) of 8.885(1) kOe for **1-Dy** and 17.999(6) kOe for **2-Dy**, which are similar to that for **[Dy(amide)(amide-alkene)]**^**+**^ (9.5 kOe at 2K^20^), but greater than that of **[Dy{N(Si**^**i**^**Pr**_**3**_**)**_**2**_}(Cp*)]^**+**^ (2.8 kOe at 2 K^21^); these are all far lower than [Dy_2_(Cp^iPr5^)_2_(μ-I)_3_], which shows *H*_C_ > 140 kOe below 60 K due to strong intramolecular exchange coupling^19^. We find that, as for **[Dy(amide)(amide-alkene)]**^**+**^^20^ and **[Dy{N(Si**^**i**^**Pr**_**3**_**)**_**2**_}(Cp*)]^**+**^^21^, *H*_C_ decreases relatively slowly with temperature (*ca*. 2 kOe K^–1^) for **1-Dy** and **2-Dy** over a wide temperature range (Fig. 2d). Comparison of the magnetic reversal rates of **1-Dy** and **2-Dy** with **[Dy{N(Si**^**i**^**Pr**_**3**_**)**_**2**_}(Cp*)]^**+**^^21^ (Fig. 2e) shows that this is because they all only switch from Raman to Orbach regimes at high temperatures^36^, which is itself a consequence of the large *U*_eff_ barriers. However, while *H*_C_ drops to <5 Oe at 73 K to define *T*_H_ for **[Dy{N(Si**^**i**^**Pr**_**3**_**)**_**2**_}(Cp*)]^**+**^^21^, *H*_C_ is still > 50 Oe for **1-Dy** and **2-Dy** at this temperature, with soft high-temperature magnetic hysteresis profiles comparable to **[Dy(amide)(amide-alkene)]**^**+**^^20^. Above 80 K, *H*_C_ starts to decrease more rapidly with temperature for **1-Dy** and **2-Dy**, with *T*_H_ = 91 K for the former and *T*_H_ = 92 K for the latter complex (*c.f*. *T*_H_ = 80 K for **[Dy(Cp**^**iPr5**^)(Cp*)]^**+**^^17^ and 100 K for **[Dy(amide)(amide-alkene)]**^**+**^^20^).

### Static electronic structure calculations

**Fig. 3:**
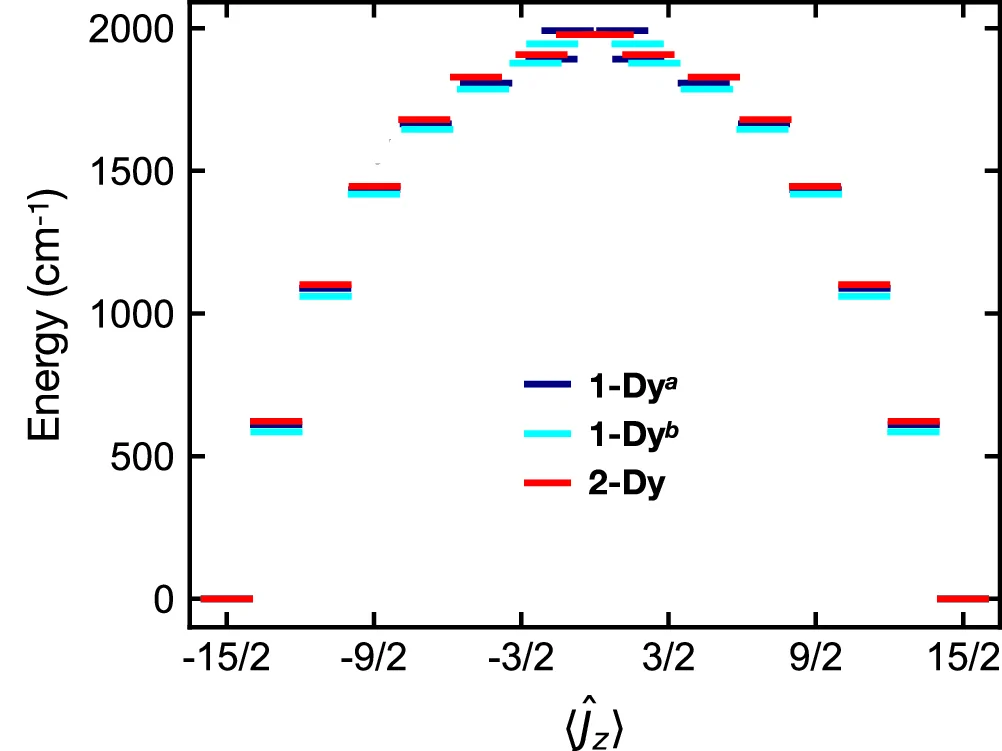
Calculated electronic structure of the two cations of complex 1-Dy (1-Dy^a^ and 1-Dy^b^) and the cation of 2-Dy. Atomic coordinates are from single crystal XRD data, and crystal field states are from SA-CASSCF-SO wavefunction. Dark blue: cation of **1-Dy**^**a**^ with N–Dy–Cp^ttt^_centroid_ = 157.84(11)°; cyan: cation of **1-Dy**^**b**^ with N–Dy–Cp^ttt^_centroid_ = 167.9(1)°; red: cation of **2-Dy** with N–Dy–Cp﻿′′′_centroid_ = 172.26(10)°.

We used OpenMolcas^37^ to perform state-average complete active space self-consistent field spin-orbit (SA-CASSCF-SO) calculations on the cations of **1-Dy** and **2-Dy**, with no optimisation of experimentally-determined atomic coordinates (see Supplementary Information Tables S8–S10). We find that the ground ^6^H_15/2_ multiplets are split by 2865 K for **1-Dy**^**a**^, 2798 K for **1-Dy**^**b**^, and 2845 K for **2-Dy**. These cations all have ground Kramers doublets (KD) with Ising-like anisotropy (*g*_*z*_ > 19.995, *g*_*x*_ and *g*_*y*_ < 10^–4^), which correspond to *m*_*J*_ = ±15/2 functions when quantised along their main magnetic anisotropy axes. The first excited states lie at 878 (**1-Dy**^**a**^) and 842 K (**1-Dy**^**b**^) and 894 K (**2-Dy**) above the ground state, with excited states showing collinear magnetic anisotropy until the fifth KD for **1-Dy**^**a**^ (2395 K) and **1-Dy**^**b**^ (2367 K), and the sixth KD for **2-Dy** (2631 K), which is the barrier over which one-phonon magnetic reversal is expected to proceed (Fig. 3)^38^. These values are greater than those calculated for the related dysprosium cyclopentadienyl-amide complex **[Dy{N(Si**^**i**^**Pr**_**3**_**)**_**2**_}(Cp*)]^**+**^ (CF splitting = 2642 K, first excited state = 770 K, fifth KD = 2220 K^21^), and the low-lying excited states of **1-Dy**^**a**^, **1-Dy**^**b**^ and **2-Dy** show more pure *m*_*J*_ compositions as expected from their nearer-to-linear Cp^R^_centroid_–Dy–N angles. The overall CF splitting for **1-Dy**^**a**^, **1-Dy**^**b**^ and **2-Dy** are all smaller than that calculated for the major component of the dysprosium bis(amide)-alkene complex **[Dy(amide)(amide-alkene)]**^**+**^ (3001 K^20^), which was predicted to relax via the fifth KD (2585 K), which we attribute due to the greater electrostatic potential of two charge-dense σ-donor ligands interacting with the Dy(III) ion rather than one σ- and one π-donor here^39^. However, the first excited state of **[Dy(amide)(amide-alkene)]**^**+**^ (841 K^20^) is lower-lying than predicted for **1-Dy**^**a**^, **1-Dy**^**b**^ and **2-Dy**, and its excited states are less pure, thus we anticipate that under-barrier magnetic relaxation processes should be suppressed in **1-Dy** and **2-Dy** by comparison.

### Spin dynamics calculations

We performed ab initio spin dynamics calculations using our recently developed methods^40–42^ on **2-Dy** and a model of **1-Dy** derived from the packing of **2-Dy**, as the large unit cell of **1-Dy** precludes calculations on this structure (see methodology). In this method, periodic density-functional theory (DFT) calculations are performed to obtain an optimised geometry and phonons. The optimised geometries of **2-Dy** and **[Dy{N(Si**^**i**^**Pr**_**3**_**)**_**2**_}(Cp*)]^**+**^^21^ are in excellent agreement with those determined by single crystal XRD; key bond lengths (Dy–N, Dy–Cp^R^_centroid_) are within 0.03 Å of the crystal structure geometry, and the N–Dy–Cp^R^_centroid_ angles are within 3°. Our model of **1-Dy** using the surrogate packing of **2-Dy** is in good agreement with cation **1-Dy**^**a**^, predicting a 159° N–Dy–Cp^R^_centroid_ angle (Supplementary Information Table S11). The low energy phonon density of states ( < 200 cm^-1^) that is responsible for driving Raman relaxation is very similar for all three systems, and similar to that previously determined for **[Dy(amide)(amide-alkene)]**^**+**^^20^, but distinct from **[Dy(Cp**^**ttt**^**)**_**2**_**]**^**+**^^43^ (Supplementary Information Figures S86–S89).

**Fig. 4:**
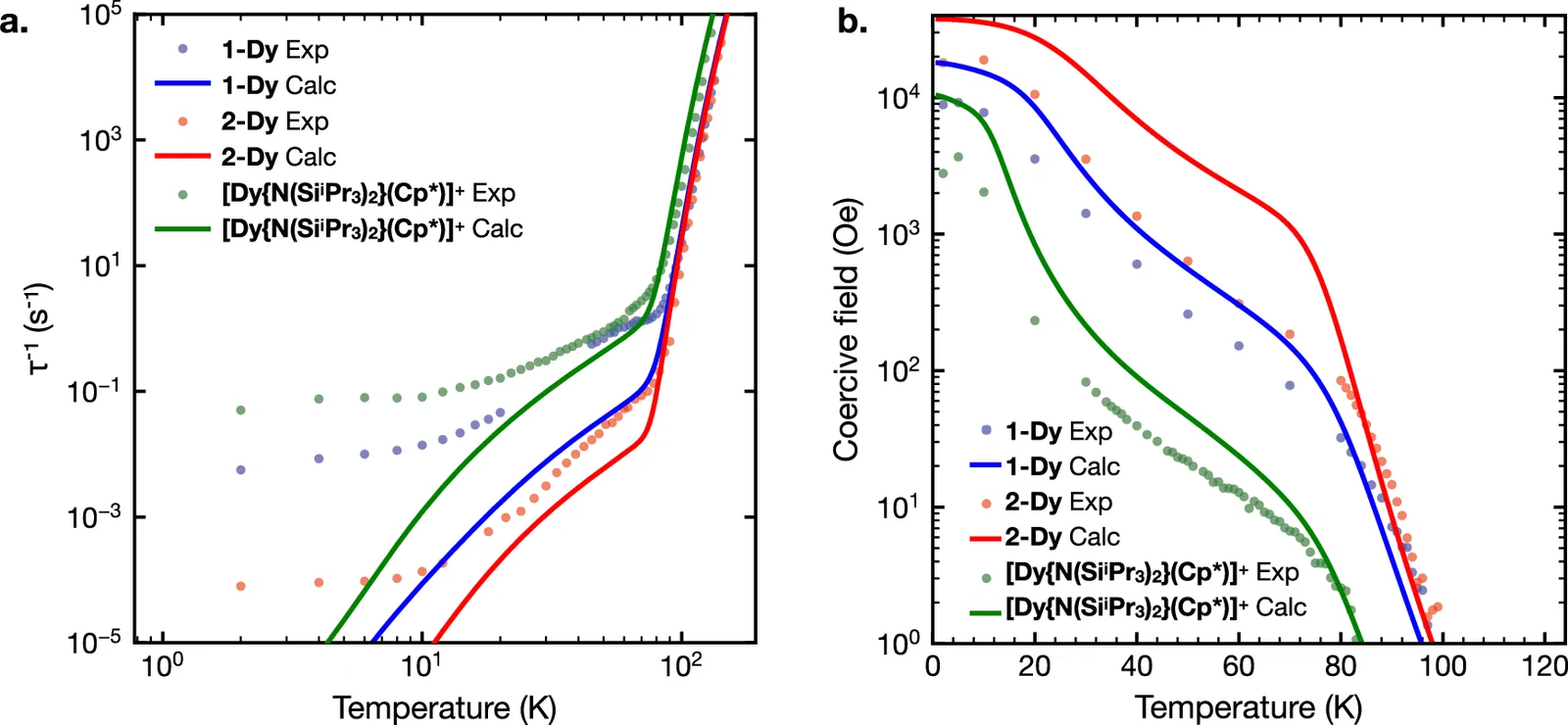
Temperature dependence of experimental and calculated magnetic relaxation rates and coercive fields of complexes 1-Dy, 2-Dy and [Dy{N(Si^i^Pr_3_)_2_}(Cp*)]^+^. **a** Experimental (points) and calculated (lines) magnetic relaxation rates, τ^−1^, vs. temperature of **1-Dy** (blue), **2-Dy** (red) and **[Dy{N**(**Si**^**i**^**Pr**_**3**_)_**2**_**}**(Cp*)**]**^**+**^^21^ (green). **b** Experimental (points) and calculated (lines) coercive field, *H*_C_, vs. temperature of **1-Dy** (blue), **2-Dy** (red) and **[Dy{N**(**Si**^**i**^**Pr**_**3**_)_**2**_**}**(Cp*)**]**^**+**^^21^ (green).

Multiconfigurational SA-CASSCF-SO calculations allow us to simulate the electronic structure of the SMM and, with the calculated phonons, determine spin-phonon coupling^44^. This spin-phonon coupling is then used to simulate the spin-dynamics at zero field via the Raman and Orbach mechanisms using our existing methodologies^42^ and established theory^41^. We find excellent agreement between the calculated and experimental magnetic reversal rates in the one-phonon Orbach region, however we underpredict the two-phonon Raman rates for all three systems (Fig. 4a); note that we do not simulate QTM processes. The discrepancy is worst for **1-Dy**, possibly due to our use of a model which uses the packing of **2-Dy**. Nevertheless, we accurately simulate the relative ordering of the three systems; the calculated Raman reversal rates for **2-Dy** at 40 K are around fifty times slower than those of **[Dy(amide)(amide-alkene)]**^**+**^^20^ and **[Dy{N(Si**^**i**^**Pr**_**3**_**)**_**2**_}(Cp*)]^**+**^^21^, and are *ca*. ten times slower than those of **[Dy(Cp**^**ttt**^**)**_**2**_**]**^**+**^^43^.

We have previously used the one-phonon spectral density (phonon DOS weighted by spin-phonon coupling strength; Supplementary Information Figure S90) to explain Raman relaxation in terms of spin-phonon coupling as a function of phonon energy. However, this metric does not explain the relative behaviour of the present systems: all three Cp^R^-amide complexes have similar spectral densities, but Raman rates differ by orders of magnitude. Furthermore, the spectral density of **[Dy(Cp**^**ttt**^**)**_**2**_**]**^**+**^ is smaller than the other four amide-containing complexes, but the calculated Raman relaxation rate is faster than for **1-Dy** and **2-Dy** (Supplementary Information Figure S91). Finally, the spectral density of **[Dy(amide)(amide-alkene)]**^**+**^^20^ is larger than **[Dy{N(Si**^**i**^**Pr**_**3**_**)**_**2**_}(Cp*)]^**+**^^21^, and yet their Raman rates are very similar. These apparent discrepancies arise because the one-phonon spectral density includes all spin-phonon coupling matrix elements equally, including those that do not contribute to Raman relaxation. In the two-phonon Raman scattering mechanism, only spin-phonon coupling matrix elements that couple the initial and final states (*viz*. the ground state doublet) to the intermediate “virtual state” state *c* (in these complexes, this is the set of the other states in the ^6^H_15/2_ manifold) contribute. Thus, the traditional one-phonon spectral density is not a very helpful metric for explaining differences in Raman relaxation rates.

One way to rectify this would be to compare a “truncated spectral density” only including the relevant matrix elements (Supplementary Information Figure S92). However, this is very similar for all amide-containing systems, with **[Dy(Cp**^**ttt**^**)**_**2**_**]**^**+**^ being distinctly smaller. This suggests that the strength of the spin-phonon coupling which drives Raman relaxation is similar between all four amide-containing complexes. To go one step further, we have recently derived a “Raman spectral density”^42^, which is the temperature-independent part of the Raman rate equation. The temperature-dependent rate integrand is then the Raman spectral density multiplied by the Bose-Einstein phonon occupation factors (Supplementary Information Equation S1 and S1). Across the five systems being compared here, both the Raman spectral density and the rate integrand (at 40 K) are consistent with the calculated Raman relaxation rate, varying by orders of magnitude between the systems (Supplementary Information Figure S93). While all five systems have highly pure $\left|{m}_{J}=\pm \frac{15}{2}\right\rangle$ ground state doublets ( > 99%), we see good correlation with the Raman rate and the impurity of this ground state doublet (i.e., $\left|{m}_{J}\ne \pm \frac{15}{2}\right\rangle$ character, which itself correlates with the N–Dy–Cp^R^_cent_ angles, Fig. 1c) for the four amide systems, while **[Dy(Cp**^**ttt**^**)**_**2**_**]**^**+**^^43^ does not follow this trend (Supplementary Information Figure S94). This suggests that in **2-Dy**, suppression of the Raman rate is due to the highly pure $\left|{m}_{J}=\pm \frac{15}{2}\right\rangle$ ground state doublet, despite the presence of the amide ligand, which induces strong spin-phonon coupling.

To understand the differing contribution of the Cpʹʹʹ and amide ligands, spin-phonon calculations were performed using localized phonon modes of **2-Dy** obtained from partial Hessian vibrational analysis (PHVA)^45^, where phonon modes are localized to the ligands of **2-Dy**. The PHVA phonon DOS of the amide ligand is similar to that of Cpʹʹʹ, but the one-phonon and Raman spectral densities are much larger for the former (Supplementary Information Figures S95–S97). This differing PHVA Raman spectral density confirms the role of the amide ligand in inducing strong spin-phonon coupling, but the similarity of the PHVA DOS suggests that it is the charge density that is more responsible for the strong spin-phonon coupling, rather than its increased flexibility compared to Cpʹʹʹ.

As SMMs are superparamagnets with no magnetic phase transitions, their magnetic hysteresis properties are determined by the underlying molecular spin dynamics^46^. Simulations of the magnetic hysteresis for **1-Dy,** **2-Dy** and **[Dy{N(Si**^**i**^**Pr**_**3**_**)**_**2**_}(Cp*)]^**+**^^21^ (summarised for ease of visualisation as coercive field versus temperature, Fig. 4b), which necessitates calculations of the relaxation rates as a function of magnetic field and orientation^20,42^, overpredict the coercive field in all cases due to our underprediction of the zero-field rates. This is different to our previous results for **[Dy(amide)(amide-alkene)]**^**+**^^20^ and **[Dy(Cp**^**ttt**^**)**_**2**_**]**^**+**^^43^ where we underpredicted the coercive field. This indicates that we do not have a systematic deviation in our methodology, but rather something that is system dependent, and likely arising from the use of DFT for optimisation and calculation of phonon modes, as well as possibly from the omission of dynamic correlation in our spin-phonon coupling calculations with SA-CASSCF-SO. Nonetheless, again we predict the correct experimental ordering and find very good qualitative agreement with the experimental trends.

To conclude, we have shown that judiciously selected π-aromatic and σ-donor ligands can be combined to provide highly axial lanthanide complexes, and in the case of dysprosium analogues this has delivered huge magnetic remanence temperatures. To achieve improved dysprosium SMMs in future, the simplest approach is still to increase CF splitting by reduced metal-ligand bond distances, increased ligand charge densities, and increased inter-ligand angles^7–11^. However, our ab initio calculations herein show the intrinsic dichotomy of this approach, in that the use of charge-dense amides is itself a cause of stronger spin-phonon coupling, that may lead to faster Raman relaxation, rather than the flexibility of the ligand itself. However, the present complexes **1-Dy** and **2-Dy** show that this can be mitigated by increasing the linearity of the complex which increases the purity of the ground state doublet and suppresses Raman relaxation rates. We note that complexes such as [Dy(C_4_R_4_)_2_]^–^^47^, [Dy(Cp^R^)(C_4_R_4_)]^48^ and [Dy(Cp^R^)(OAr)][B(C_6_F_5_)_4_] (Ar = aryl)^49^ have all been targeted^12^. Here we have highlighted how ligand steric complementarity has to be of prime consideration for controlling the lanthanide coordination sphere to deliver more linear complex geometries that effectively suppress under-barrier magnetic relaxation mechanisms.

## Methods

### Synthesis and characterisation

All manipulations were conducted under argon with the strict exclusion of oxygen and water by using Schlenk line and glove box techniques. Glassware was flame-dried under vacuum prior to use. Argon was passed through a column of activated 3 Å molecular sieves and Cu catalyst prior to use. Isolated compounds were dried *in vacuo* on a Schlenk line to the point at which the flask could maintain a constant static pressure less than 5 × 10^-3^ mbar. C_6_H_6_ and C_6_D_6_ were purchased anhydrous, degassed, and stored under argon over a K mirror or activated 3 Å molecular sieves, respectively. *n*-Hexane was refluxed over molten potassium for 3 days, distilled, and stored under argon over a potassium mirror. C_6_H_5_F was refluxed over CaH_2_ for 3 days, distilled, and stored under argon over activated 3 Å molecular sieves. [Ln{N(Si^i^Pr_3_)_2_}(BH_4_)_2_]^25^, KCp^ttt^^26^, KCp′′′^27^ and [CPh_3_][Al{OC(CF_3_)_3_}_4_]^24^ were prepared according to literature procedures.

NMR spectra were recorded at 298 K on a Bruker AVIII HD 400 MHz or 500 MHz spectrometer. Chemical shifts are reported in ppm and coupling constants in Hz. ^1^H and ^13^C{^1^H}_DEPTQ_ NMR spectra recorded in C_6_D_6_ are referenced to the solvent signal^50^. NMR spectra recorded in C_6_H_5_F were locked to an internal sealed capillary of C_6_D_6_, with ^1^H NMR spectra referenced using the highest intensity peak of the lower frequency fluorobenzene multiplet (δ_H_ 6.86) and ^13^C{^1^H}_DEPTQ_ spectra referenced to C_6_D_6_. ^11^B/^11^B{^1^H} (H_3_BO_3_/D_2_O), ^19^F (C_7_H_5_F_3_/CDCl_3_) and ^29^Si{^1^H}_DEPT90_ (SiMe_4_) NMR spectra were referenced to external standards. The *C*(CF_3_)_3_ carbon resonances of the [Al{OC(CF_3_)_3_}_4_]^–^ anion was not observed in the ^13^C{^1^H} NMR spectra of **1-Y** or **2-Y**, likely due to quadrupolar broadening by the 100% abundant *I* = ^5^/_2_^27^Al nuclei and coupling to multiple 100% abundant *I* = ^1^/_2_^19^F nuclei.

Single crystal XRD data were collected on either a Rigaku XtalLAB Synergy-S diffractometer (**1-Ln,** **2-Ln,** **3-Ln**) or a Rigaku FR-X diffractometer (**4-Ln**), both equipped with a HyPix 6000HE photon counting pixel array detector and a mirror-monochromated Cu Kα X-ray source (λ = 1.5418 Å). Intensities were integrated from data recorded on 0.5° frames by ω rotation. Cell parameters were refined from the observed positions of all strong reflections in each data set. A Gaussian grid face-indexed with a beam profile was applied for all structures, with crystal faces determined directly on the mounted crystal using CrysAlisPro^51^. The structures were solved using SHELXT^52^; the datasets were refined by full-matrix least-squares on all unique *F*^2^ values^53^. Anisotropic displacement parameters were used for all non-hydrogen atoms with constrained riding hydrogen geometries, with the exception of borohydride H atoms, which were located in the difference map and refined isotropically; *U*_iso_(H) was set at 1.2 (1.5 for methyl groups) times *U*_eq_ of the parent atom. Crystallographic disorder in the [Al{OC(CF_3_)_3_}_4_]^–^ anions of **1-Ln** were modelled by treating each of the three disordered perfluoroalkoxy groups present in each structure as an equivalent residue, which were then allowed to freely refine over two positions, converging at a final ratio of 0.66: 0.34 for **1-Dy** and 0.77: 0.23 for **1-Y**. To aid the refinement of **1-Y**, one Al···F distance was restrained to be over 3.1 Å, and two disordered O and two disordered C atoms were allowed to occupy the same space. Similarity thermal restraints were applied to the disordered anion, and enhanced rigid bond restraints were used on all atoms. The datasets for **2-Ln,** **3-Ln** and **4-Ln** did not exhibit any significant disorder, though **2-Y** was refined a two-component inversion twin. For **3-Ln** and **4-Ln** the B–H bonds were restrained to be approximately 1.15 Å, and the H···H distances were restrained to be approximately equal. The largest features in the final difference syntheses were close to heavy atoms and were of no chemical significance. CrysAlisPro^51^ was used for control and integration, and SHELX^52,53^ was employed through OLEX2^54^ for structure solution and refinement. ORTEP-3^55^ and POV-Ray^56^ were used for molecular graphics.

Powder XRD data were collected at 100 K using a Rigaku FR-X rotating anode single crystal X-ray diffractometer with a HyPix 6000HE photon counting pixel array detector and a mirror-monochromated Cu Kα X-ray source (λ = 1.5418 Å). Samples of crystalline material finely ground and mixed into a paste with minimal Fomblin were mounted on glass filaments^57^. Data were collected between 3 and 120 °θ with a detector distance of 150 mm and a beam divergence of 1.5 mRad using CrysAlisPro^51^. For data processing, the instrument was calibrated using silver behenate as standard, then the data were reduced and integrated using CrysAlisPro^51^. Le Bail profile analysis was performed using Profex 5.5.0 using default refinement and convergence limits^58^. ATR-IR spectra were recorded as microcrystalline powders using a Agilent Cary FT-IR spectrometer with a diamond-ATR module within an argon-filled glovebox at ambient temperature. Elemental analysis (C, H, N) samples were prepared in an argon-filled glovebox at the Microanalytical service, Department of Chemistry, the University of Manchester^32^.

#### General procedure for the synthesis of 1-Ln and 2-Ln

A solution of **3-Ln** or **4-Ln** (0.2 mmol) in C_6_H_5_F (2 mL) and a solution of [CPh_3_][Al{OC(CF_3_)_3_}_4_] (242.0 mg, 0.2 mmol) in C_6_H_5_F (2 mL) were combined with gentle agitation and left to stand for 2 h at ambient temperature. The volatiles were removed *in vacuo*, and the residues triturated with excess *n*-hexane (3 × 2 mL) to remove the majority of the concomitantly formed HCPh_3_. The resulting solid was redissolved in C_6_H_5_F (1 mL), filtered, and layered with *n*-hexane (*ca*. 5 mL), which upon diffusion at –35 °C gave the title complexes as crystalline solids. The supernatants were decanted and the crystals dried *in vacuo*. No attempt was made to collect any subsequent crop.

#### [Dy{N(Si^i^Pr_3_)_2_}(Cp^ttt^)][Al{OC(CF_3_)_3_}_4_] (1-Dy)

Yield: 163.2 mg, 0.0965 mmol, 48%, pale yellow crystals. Anal. Calcd for C_51_H_71_AlDyF_36_NO_4_Si_2_ (1691.73 g mol^-1^) C, 36.21; H, 4.23; N, 0.83. Found: C, 35.42; H, 3.82; N, 0.76. ^19^F NMR (376.40 MHz, C_6_H_5_F): δ 94.78 (s, OC(CF_3_)_3_). FTIR (ATR, microcrystalline): *ṽ* (cm^–1^) = 2964 (w), 2873 (w), 2785 (w), 2793 (w), 2594 (w), 1466 (w), 1349 (m), 1295 (s), 1275 (s), 1238 (s), 1211 (s), 1162 (s), 1067 (w), 1002 (m), 968 (s), 880 (m), 831 (m), 755 (w), 726 (s), 710 (s), 668 (m).

#### [Y{N(Si^i^Pr_3_)_2_}(Cp^ttt^)][Al{OC(CF_3_)_3_}_4_] (1-Y)

Yield: 120.1 mg, 0.0742 mmol, 37%, colourless crystals. Anal. Calcd for C_51_H_71_AlF_36_NO_4_Si_2_Y (1618.13 g mol^-1^) C, 37.86; H, 4.42; N, 0.87. Found: C, 37.97; H, 4.24; N, 0.82. ^1^H NMR (400.07 MHz, C_6_H_5_F): δ 6.37 (s, 2H, 3,5-CH), 1.12 (s, 18H, 1,2-CCMe_3_), 0.97 (s, 9H, 4-CCMe_3_), 0.87 (d, ^3^*J*_HH_ = 7.5 Hz, 18H, CH(CH_3_)_2_), 0.55 (sept, ^3^*J*_HH_ = 7.3 Hz, 6H, CH(CH_3_)_2_). ^13^C{^1^H}_DEPTQ_ NMR (100.60 MHz, C_6_H_5_F): δ 146.39 (s, 4-CCMe_3_), 145.22 (s, 1,2-CCMe_3_), 121.99 (q, ^1^*J*_FC_ = 292 Hz, OC(CF_3_)_3_), 117.94 (s, 3,5-CH), 34.71 (s, 1,2-CCMe_3_), 33.43 (s, 4-CCMe_3_), 32.64 (s, 1,2-CCMe_3_), 30.78 (s, 4-CCMe_3_), 18.48 (s, CH(CH_3_)_2_), 15.32 (s, CH(CH_3_)_2_). ^19^F NMR (376.40 MHz, C_6_H_5_F): δ 75.08 (s, OC(CF_3_)_3_).). ^29^Si{^1^H}_DEPT90_ NMR (79.48 MHz, C_6_H_5_F): δ –4.86 (s). FTIR (ATR, microcrystalline): *ṽ* (cm^–1^) = 2964 (w), 2873 (w), 2785 (w), 2793 (w), 2594 (w), 1466 (w), 1349 (m), 1295 (s), 1275 (s), 1238 (s), 1211 (s), 1162 (s), 1067 (w), 1002 (m), 968 (s), 880 (m), 831 (m), 755 (w), 726 (s), 710 (s), 668 (m).

#### [Dy{N(Si^i^Pr_3_)_2_}(Cp′′′)][Al{OC(CF_3_)_3_}_4_] (2-Dy)

Yield: 129.8 mg, 0.0746 mmol, 37%, pale yellow crystals. Anal. Calcd for C_48_H_71_AlDyF_36_NO_4_Si_5_ (1739.95 g mol^-1^) C, 33.13; H, 4.11; N, 0.81. Found: C, 32.94; H, 3.91; N, 0.70. ^19^F NMR (376.40 MHz, C_6_H_5_F): δ 90.40 (s, OC(CF_3_)_3_). FTIR (ATR, microcrystalline): *ṽ* (cm^–1^) = 2956 (w), 2874 (w), 2792 (w), 2737 (w), 2591 (w), 1462 (w), 1351 (m), 1296 (s), 1274 (s), 1237 (s), 1210 (s) 1165 (s), 1087 (m), 1024 (m), 1002 (m), 970 (s), 932 (m), 871 (m), 827 (m), 755 (m), 726 (s), 708 (m), 666 (m).

#### [Y{N(Si^i^Pr_3_)_2_}(Cp′′′)][Al{OC(CF_3_)_3_}_4_] (2-Y)

Yield: 144.0 mg, 0.0864 mmol, 43%, pale yellow crystals. Anal. Calcd for C_48_H_71_AlF_36_NO_4_Si_5_Y (1666.35 g mol^-1^) C, 34.60; H, 4.29; N, 0.84. Found: C, 35.10; H, 4.21; N, 0.78. ^1^H NMR (400.07 MHz, C_6_H_5_F): δ 7.42 (s, 2H, 3,5-CH), 1.05 (d, ^3^*J*_HH_ = 7.2 Hz, 36H, CH(CH_3_)_2_), 0.77 (sept, ^3^*J*_HH_ = 7.4 Hz, 6H, CH(CH_3_)_2_), 0.28 (s, 18H, 1,2-CSiMe_3_), 0.21 (s, 9H, 4-CSiMe_3_). ^13^C{^1^H}_DEPTQ_ NMR (100.60 MHz, C_6_H_5_F): δ 145.26 (s, 1,2-CSiMe_3_), 140.60 (s, 3,5-CH), 140.28 (s, 4-CSiMe_3_), 121.99 (q, ^1^*J*_FC_ = 292 Hz, OC(CF_3_)_3_), 18.76 (s, CH(CH_3_)_2_), 14.48 (s, CH(CH_3_)_2_), 0.72 (s, 1,2-CSiMe_3_), -0.77 (s, 4-CSiMe_3_). ^19^F NMR (376.40 MHz, C_6_H_5_F): δ 75.20 (s, OC(CF_3_)_3_). ^29^Si{^1^H}_DEPT90_ NMR (79.48 MHz, C_6_H_5_F): δ –4.85 (s, N(Si^i^Pr_3_)_2_), –7.54 (s, 4-SiMe_3_), –7.73 (s, 1,2-SiMe_3_). FTIR (ATR, microcrystalline): *ṽ* (cm^–1^) = 2956 (w), 2874 (w), 2792 (w), 2737 (w), 2591 (w), 1462 (w), 1351 (m), 1296 (s), 1274 (s), 1237 (s), 1210 (s) 1165 (s), 1087 (m), 1024 (m), 1002 (m), 970 (s), 932 (m), 871 (m), 827 (m), 755 (m), 726 (s), 708 (m), 666 (m).

#### General procedure for the preparation of borohydrides 3-Ln and 4-Ln

A mixture of [Ln{N(Si^i^Pr_3_)_2_}(BH_4_)_2_]_4_ (Ln = Dy: 260.4 mg; Ln = Y, 223.6 mg; 0.5 mmol) and KCp^ttt^ (136.3 mg, 0.5 mmol) or KCp′′′ (160.4 mg, 0.5 mmol) were suspended in C_6_H_6_ (10 mL) and stirred at ambient temperature for 18 h. The crude reaction mixture was evaporated to dryness *in vacuo* and the product extracted into *n*-hexane (3 × 10 mL), and the solution then concentrated to ca. 5 mL. Storage at –35 °C gave crystalline material, which was triturated with cold (–35 °C) *n*-hexane (2 × 1 mL) and dried thoroughly *in vacuo*. An additional crop was obtained on storage of the *n*-hexane triturate at –35 °C.

#### [Dy{N(Si^i^Pr_3_)_2_}(Cp^ttt^)(BH_4_)] (3-Dy)

Yield: 232.0 mg, 0.314 mmol, 62%, colourless crystals. Anal. Calcd for C_35_H_75_BDyNSi_2_ (739.47 g mol^-1^) C, 56.85; H, 10.22; N, 1.89. Found: C, 56.14; H, 10.40; N, 1.98. ^1^H NMR (400.07 MHz, C_6_D_6_): δ 87.5 (broad, FWHM ~ 7,000 Hz), 23.0, 18.0, 17.5, 17.1, 16.0, 3.01 (broad, FWHM ~ 28,000 Hz), –33.25 (broad, FWHM ~ 12,000 Hz). FTIR (ATR, microcrystalline): *ṽ* (cm^–1^) = 2946 (s), 2864 (s), 2699 (m), 2487 (m), 2200 (w), 2132 (w), 1468 (s), 1386 (w), 1363 (m), 1234 (m), 994 (s), 972 (s), 956 (s), 942 (s), 879 (s), 826 (s), 692 (s).

#### [Y{N(Si^i^Pr_3_)_2_}(Cp^ttt^)(BH_4_)] (3-Y)

Yield: 277.3 mg, 0.416 mmol, 83%, colourless crystals. Anal. Calcd for C_35_H_75_BNSi_2_Y (665.88 g mol^-1^) C, 63.13; H, 11.35; N, 2.10. Found: C, 62.72; H, 11.48; N, 2.16. ^1^H{^11^B} NMR (400.07 MHz, C_6_D_6_): δ 6.29 (s, 2H, 3,5-CH), 1.50 (s, 18H 1,2-CCMe_3_), 1.35 (d, ^1^*J*_YH_ = 13.3 Hz, 4H, BH_4_), 1.32 (d, ^3^*J*_HH_ = 7.3 Hz, 18H, CH(CH_3_)_2_), 1.28 (d, ^3^*J*_HH_ = 7.3 Hz, 18H, CH(CH_3_)_2_), 1.26 (s, 9H, 4-CCMe_3_), 0.90 (sept, ^3^*J*_HH_ = 7.3 Hz, 6H, CH(CH_3_)_2_). The BH_4_ resonance in the ^11^B-coupled ^1^H spectrum is a very broad quartet of doublets, obscured by the ^i^Pr and ^t^Bu resonances. ^11^B NMR (128.36 MHz, C_6_D_6_): δ –19.34 (pent, ^1^*J*_BH_ = 83.3 Hz, BH_4_). ^13^C{^1^H}_DEPTQ_ NMR (100.60 MHz, C_6_D_6_): δ 140.86 (d, ^1^*J*_YC_ = 0.6 Hz, 1,2-CCMe_3_), 139.42 (d, ^1^*J*_YC_ = 2.2 Hz, 4-CCMe_3_), 113.35 (d, ^1^*J*_YC_ = 0.9 Hz, 3,5-CH), 34.83 (s, 1,2-CCMe_3_), 34.30 (s, 1,2-CCMe_3_), 33.99 (s, 4-CCMe_3_) 32.27 (s, 4-CCMe_3_), 21.18 (s, CH(CH_3_)_2_), 20.38 (s, CH(CH_3_)_2_), 18.13 (s, CH(CH_3_)_2_). ^29^Si{^1^H}_DEPT90_ NMR (79.48 MHz, C_6_D_6_): δ − 3.99 (s, N(Si^i^Pr_3_)_2_). FTIR (ATR, microcrystalline): *ṽ* (cm^–1^) = 2951 (s), 2865 (s), 2694 (m), 2487 (m), 2202 (w), 2129 (m), 1458 (s), 1393 (m) 1361 (m), 1238 (s), 1169 (m), 1001 (m), 930 (s), 874 (s), 834 (m), 720(s), 678 (m).

#### [Dy{N(Si^i^Pr_3_)_2_}(Cp′′′)(BH_4_)] (4-Dy)

Yield: 276.9 mg, 0.352 mmol, 70%, colourless crystals. Anal. Calcd for C_32_H_75_BDyNSi_5_ (787.69 g mol^-1^) C, 48.79; H, 9.60; N, 1.78. Found: C, 48.41; H, 9.76; N, 1.86. ^1^H NMR (400.07 MHz, C_6_D_6_): δ 84.1 (broad, FWHM ~ 7,500 Hz), 19.5, 14.1, 13.9, 13.5, 12.4, 4.5 (broad, FWHM ~ 29,000 Hz), –36.7 (broad, FWHM ~ 14,000 Hz). FTIR (ATR, microcrystalline): *ṽ* (cm^–1^) = 2946 (s), 2865 (s), 2689 (m), 2494 (m), 2265 (w), 2216 (w), 2139 (w), 1461 (m), 1391 (w), 1244 (s), 1194 (m), 1143 (w), 1087 (s), 994 (s), 933(s), 881 (m), 853 (s), 826 (s), 751 (s), 722 (s), 687 (s).

#### [Y{N(Si^i^Pr_3_)_2_}(Cp′′′)(BH_4_)] (4-Y)

Yield: 235.9 mg, 0.330 mmol, 66%, colourless crystals. Anal. Calcd for C_32_H_75_BNSi_5_Y (714.10 g mol^-1^) C, 53.82; H, 10.59; N, 1.96. Found: C, 52.71; H, 10.96; N, 2.10. ^1^H{^11^B} NMR (400.07 MHz, C_6_D_6_): δ 6.97 (s, 2H, 3,5-CH), 1.27 (d, ^3^*J*_HH_ = 7.4 Hz, 18H, CH(CH_3_)_2_), 1.26 (d, ^3^*J*_HH_ = 7.4 Hz, 18H, CH(CH_3_)_2_), 1.16 (d, ^1^*J*_YH_ = 13.2 Hz, 4H, BH_4_), 0.94 (sept, ^3^*J*_HH_ = 7.4 Hz, 6H, CH(CH_3_)_2_), 0.45 (s, 18H, 1,2-SiMe_3_), 0.25 (s, 9H, 4-SiMe_3_). The BH_4_ resonance in the ^11^B-coupled ^1^H spectrum is a very broad quartet of doublets, obscured by the ^i^Pr resonances. ^11^B NMR (128.36 MHz, C_6_D_6_): δ –19.04 (pent, ^1^*J*_BH_ = 85.2 Hz, BH_4_). ^13^C{^1^H}_DEPTQ_ NMR (100.60 MHz, C_6_D_6_): δ 139.09 (d, ^1^*J*_YC_ = 0.9 Hz, 1,2-CSiMe_3_), 135.35 (s, 3,5-CH), 130.22 (d, ^1^*J*_YC_ = 2.8 Hz, 4-CSiMe_3_), 21.01 (s, CH(CH_3_)_2_), 20.24 (s, CH(CH_3_)_2_), 17.29 (s, CH(CH_3_)_2_), 1.97 (s, 1,2-CSiMe_3_), 0.87 (s, 4-CSiMe_3_). ^29^Si{^1^H}_DEPT90_ NMR (79.48 MHz, C_6_D_6_): δ –3.81 (s, N(Si^i^Pr_3_)_2_), –8.13 (s, 4-SiMe_3_), –8.44 (s, 1,2-SiMe_3_). FTIR (ATR, microcrystalline): *ṽ* (cm^–1^) = 2946 (s), 2865 (s), 2689 (m), 2494 (m), 2265 (w), 2216 (w), 2139 (w), 1461 (m), 1391 (w), 1244 (s), 1194 (m), 1143 (w), 1087 (s), 994 (s), 933(s), 881 (m), 853 (s), 826 (s), 751 (s), 722 (s), 687 (s).

### Magnetometry

Magnetic measurements were performed on a Quantum Design MPMS3 superconducting quantum interference device (SQUID) magnetometer. Crystalline samples of **1-Dy** (27.7 mg) and **2-Dy** (24.2 mg) were ground to a microcrystalline powder and placed into a borosilicate NMR tube along with eicosane (22.8 mg and 24.1 mg respectively). The eicosane was melted by gentle heating and dispersed throughout the sample to immobilise the crystallites. After cooling, the tube was placed under vacuum (*ca*. 5 × 10^-3 ^mbar) and flame-sealed to give an ampoule with a length of *ca*. 3 cm. The ampoule was mounted in the centre of a drinking straw using friction by wrapping it with Kapton tape, and the straw was fixed to the end of the sample rod. The measurements were corrected for the diamagnetism of the straw, borosilicate tube and eicosane using calibrated blanks, for the shape of the sample using Quantum Design Geometry Simulator (correction factor 1.006 for **1-Dy** and 1.033 for **2-Dy**), and for the intrinsic diamagnetism of the sample, which was estimated as the molecular weight multiplied by ‒0.5 × 10^–6^ cm^3^ K mol^–1^.

Zero-field cooled / field cooled (ZFC/FC) magnetic susceptibility was measured under a 0.1 T field at 0.5 K min^–1^ between 1.8 and 300 K. Equilibrium susceptibility measurements were performed using vibrating sample magnetometry (VSM) mode with 5 mm vibrational amplitude and 2 s averaging time. Hysteresis measurements were performed between ± 7 T at low temperature, and ± 3 T at higher temperatures on the approach to *T*_*H*_, using VSM mode with a 5 mm vibration amplitude and 2 s averaging time. All field-swept measurements were performed in continuous sweep mode with a sweep rate of 22 Oe s^–1^ across the entire field range. The field was corrected using a Pd reference sample measured under the same conditions and interpolated using B-spline fitting functions^28^.

DC decay measurements of **2-Dy** were performed at temperatures between 2 and 21 K. The sample was initially saturated at 7 T before being the field was swept to 22 Oe (calibrated with a standard palladium sample to give an actual field of 0 Oe)^35^ at 700 Oe/s. The moment was measured throughout the whole process using VSM mode with 5 mm amplitude and averaging time of 2 s. The data was fitted to a stretched exponential function $M\left(t\right)={M}_{{{{\rm{eq}}}}}+\,\left({M}_{0}-{M}_{{{{\rm{eq}}}}}\right)\exp \left[-{\left(\frac{t}{{\tau }^{*}}\right)}^{\beta }\right]$. During the fitting process, *M*_eq_ was fixed to zero and *M*_0_ fixed to the first stable point at target field.

Alternating current (AC) susceptibility measurements were recorded at temperatures between 2 and 130 K (**1-Dy**), and 2 and 142 K (**2-Dy)**, using 8 frequencies per decade between 0.1 and 1000 Hz with a 5 Oe oscillating field. Due to instrumental limitations, 750 and 1000 Hz frequencies were measured with a 2 Oe oscillating field. Averages were performed for 2 s or for 10 cycles, whichever was longer.

Waveform measurements^34^ were performed to obtain low frequency in-phase and out-of-phase susceptibilities between 2 and 70 K (**1-Dy**), and 24 and 90 K (**2-Dy**). The magnet was reset before the sample was cooled in zero field and the moment measured as a function of time as a square wave dc field was applied. The magnitude of the field was ± 8 Oe, the sweep rate 700 Oe s^–1^, with frequencies in the range 100 μHz to 100 mHz. Measurements were performed using VSM mode with a vibrational amplitude of 0.5 mm and averaging time of 0.5 s. A fixed range of 1 V was used to avoid unevenly spaced datapoints due to autoranging delays. In- and out-of-phase susceptibilities were extracted from the Fourier transform of the magnetic field and sample moment using CC-FIT2^18,35^.

Susceptibilities extracted from analysis of waveform data and AC measurements were fitted to a Generalised Debye model $\chi \left(\omega \right)={\chi }_{S}+\left({\chi }_{T}-{\chi }_{S}\right)\frac{1}{1+{\left(i\omega \tau \right)}^{1-\alpha }}$ or a Double Generalised Debye model$\,\chi \left(\omega \right)={\chi }_{{total}}+\frac{\varDelta {\chi }_{1}}{1+{\left(i\omega {\tau }_{1}\right)}^{1-{\alpha }_{1}}}+\frac{\varDelta {\chi }_{2}}{1+{\left(i\omega {\tau }_{2}\right)}^{1-{\alpha }_{2}}}$ in CC-FIT2 to extract relaxation rates and distributions^18,35^ (Tables S5 and S6). Here, ${\tau }^{-1}$ is the relaxation rate, ω is the angular frequency of the oscillating field, α parameterizes the width of the distribution^18^, ${\chi }_{T}$ and ${\chi }_{S}$ are the isothermal and adiabatic susceptibilities, ${\chi }_{{total}}={\chi }_{1S}+{\chi }_{2S}$, $\varDelta {\chi }_{1}={\chi }_{1T}-{\chi }_{1S}$ and $\varDelta {\chi }_{2}={\chi }_{2T}-{\chi }_{2S}$.

### Computational

CASSCF-SO calculations were performed with OpenMolcas 24.06^37^ on the cationic components of **1-Dy** (including both cation geometries) and **2-Dy** at the crystal structure geometries. To represent the crystalline environment, a single cation was embedded in a spherical point charge cluster of unit cells with a radius of approximately 40 Å. These point charges are CHELPG charges obtained from gas-phase DFT calculations of each anionic and cationic component. The spherical cluster was surrounded by a spherical conductor which screens unphysical surface charges, to reproduce the Madelung potential^40^. The second order Douglas-Kroll-Hess Hamiltonian was used^59^, and ANO-RCC basis sets of varying quality: VTZP contractions on Dy, VDZP on coordinated N and C, and VDZ elsewhere^60^. The resolution of the identity approximation was used with the Cholesky ‘atomic compact’ auxiliary basis set^61^. State averaged CASSCF calculations were performed with 21 sextet, 224 quartet, and 490 doublet states. Of these, the lowest 21, 128 and 130 states were mixed with spin-orbit coupling.

To calculate the spin-phonon coupling, we followed our established methodology^40–44,62^. First, periodic DFT geometry optimizations were performed using VASP^63–66^ on **2-Dy** (version 6.4.3), **[Dy{N(Si**^**i**^**Pr**_**3**_**)**_**2**_}(Cp*)]^**+**^^21^, and a model of **1-Dy** (version 6.6.0). The large unit cell of **1-Dy** precludes performing periodic DFT calculations on it. Instead, we used the optimized geometry of **2-Dy**, substituting Cp”‘ for Cp^ttt^ and optimised this geometry. The PBE functional was used^67^, with a plane wave basis set with an energy cutoff of 900 eV (determined by convergence testing) and PAW pseudopotentials including 4f-in-core pseudopotential on Dy^68,69^. The electronic structure was sampled at the Γ point. The D3 dispersion correction was used^70^. Atomic positions and cell parameters were optimized, fixing the cell volume to experiment (**2-Dy**), and half that of experiment (**1-Dy** model, accounting for the different packing between **1-Dy** our model). Phonons were calculated by finite displacements using Phonopy^71^.

CASSCF calculations were performed following the same methodology as the calculations at the crystal structure geometry, other than only the high-spin 18 sextet roots (i.e., ^6^H + ^6^F) were calculated. The electronic structure is very similar to that calculated at the crystal structure geometries (Supplementary Information Tables S12-S14). The spin-phonon coupling for each phonon was calculated using our linear vibronic coupling method^44,63^.

Magnetic relaxation rates were calculated at the optimized DFT geometry using Tau2^42^. In addition to rate calculations on **1-Dy,** **2-Dy** and **[Dy{N(Si**^**i**^**Pr**_**3**_**)**_**2**_}(Cp*)]^**+**^^21^, we additionally calculated the relaxation rates of **[Dy(amide)(amide-alkene)]**^**+**^^20^ and **[Dy(Cp**^**ttt**^**)**_**2**_**]**^**+**^^43^, ﻿based on the spin-phonon calculations in our previous studies but using our updated methodology in Tau2. A 2 Oe field was applied along the principal axis. One phonon (Orbach and direct, equation 3 of ref. ^42^) and Raman-I (equation 15 of ref. ^42^) rates were calculated. An anti-Lorentzian lineshape was used, with fixed full-width at half-maximum (FWHM) linewidths between 1-100 cm^−1^. For Raman rates, the phonon lineshape is restricted to the equivalent of 2 standard deviations of the full lineshape and modes whose phonon energy plus the phonon linewidth is less than the first excited Kramers doublet are included in the Raman rate calculation. In Tau2, Raman rates are evaluated via calculation of a temperature-independent “Raman spectral density” (equations 11−14 of ref. ^42^); this is evaluated on an even-spaced grid of phonon energies with a resolution of FWHM / 10. For one phonon rates, all modes are included. Phonons were sampled on Γ centred *q-*meshes from 1×1×1 to 3×3×3. While our calculated phonons have no imaginary modes at the Γ point, meshes other than 1×1×1 have small ( < 10*i* cm^−1^) imaginary modes. Their effect is negligible since (consistent with our previous studies^20,43^) rates calculated on the 1×1×1 mesh are indistinguishable from others, whether imaginary modes are removed or set to their absolute value (Figure S98). Rates showed little dependence on FWHM (Figure S99–S101), so for consistency with our previous studies we chose a FWHM of 10 cm^−1^^20,43^. Raman rates were calculated between the ground state doublet, including 16 spin-orbit states (the ^6^H_15/2_ level) in the sum over c states. For one-phonon rates, transitions within the ^6^H_15/2_ level were included. Hysteresis simulation followed the methodology we developed for **[Dy(amide)(amide-alkene)]**^**+**^^20^. Rates were calculated on a mesh of temperatures (1–120 K), field orientations (50 on a Fibonacci hemisphere) and field magnitudes (2 Oe, and 0.05-7 T every 0.05 T, slightly finer than our previous study).

In the partial Hessian vibronic analysis (PHVA) of **2-Dy**, atoms outside the localized region were assigned near-infinite mass (10^9^ a.u.), driving modes with these atoms towards zero energy, and resulting in periodic phonons localized modes in the region of interest. This results in a large spike at near zero energy in the PHVA phonon DOS (Supplementary Information Figure S95a). When calculating the spin-phonon coupling, contributions from the point charge region were excluded. This PHVA analysis was performed on three regions: the Cpʹʹʹ ligand, the amide ligand (in both cases excluding the central Dy) and the whole [Dy{N(Si^i^Pr_3_)_2_}(Cpʹʹʹ)]^+^ cation, in addition to a calculation using non-localized phonons but only including spin-phonon coupling of the point charge region.

DFT geometry optimizations and vibrational analyses were performed on **3-Y,** **4-Y**, the cations of **1-Y** and **2-Y**, and separately the [Al{OC(CF_3_)_3_}_4_]^–^ anion, for the purposes of assigning the experimental IR spectra (Figures S24, S27, S30 and S33)^72^. Calculations were performed in the Orca 5.0 software package^73^, at the PBE0^74,75^-D4^76,77^ / def2-TZVP^78^ level (including the default effective core potential for yttrium^79^). The default Orca 5.0 integration grids, convergence method, and convergence thresholds (for both SCF and geometry iterations) were used throughout. The SCF energy calculations were expedited through employment of the RIJCOSX approximation^80^ (and associated def2/J auxiliary basis set^81^) and DIIS convergence acceleration^82^. Geometry-optimized structures were verified as minima on the potential energy surface through the absence of imaginary vibrational modes.

## Supplementary information

Supplementary Information

Transparent Peer Review file

## Source data

Source Data

## Data Availability

Research data generated in this study have been deposited on FigShare at https://figshare.com/doi/10.48420/32567592. A preprint of this article was deposited on *ChemRxiv*^83^. All data are available from the corresponding authors upon request. Source Data are provided with this manuscript. Crystallographic data for the structures reported in this Article have been deposited at the Cambridge Crystallographic Data Centre, under deposition numbers CCDC 2558778 (**1-Dy**), 2558779 (**1-Y**), 2558780 (**2-Dy**), 2558781 (**2-Y**), 2558782 (**3-Dy**), 2558783 (**3-Y**), 2558784 (**4-Dy**) and 2558785 (**4-Y**). Copies of the data can be obtained free of charge via https://www.ccdc.cam.ac.uk/structures/. Source data are provided with this paper.
