# Supplementary material for: Axial dysprosium cyclopentadienyl-amide single-molecule magnets with hysteresis up to 92 kelvin
Source: Nat Commun. 2026 Aug 26;17:10212. doi: 10.1038/s41467-026-77104-z (PMC13612813; doi:10.1038/s41467-026-77104-z)
Supplement: Supplementary file 1 [file 41467_2026_77104_MOESM1_ESM.pdf]

Supplementary Information for:

Axial dysprosium cyclopentadienyl-amide single-molecule magnets with hysteresis up to 92 kelvin

Jack Emerson-King[1], Benjamin E. Atkinson[2], William J. A. Blackmore[1], Jacinta Rees[2], Sophie C. Corner[1], Jack Baldwin[1], Nicholas F. Chilton[1,2]* & David P. Mills[1]*

[1]*Department of Chemistry, The University of Manchester, Oxford Road, Manchester, M13 9PL, UK.*

[2]*Research School of Chemistry, The Australian National University, Sullivans Creek Road, Canberra, ACT, 2601, Australia.*

**Email:* nicholas.chilton@anu.edu.au; david.mills@manchester.ac.uk

Contents

1. Single crystal X-ray diffraction S3

2. Powder X-ray diffraction S20

3. IR spectra S24

3.1 [Ln{N(Si^{i}Pr$_3$)$_2$}(Cp^{ttt})][Al{OC(CF$_3$)$_3$}$_4$] (1-Ln) S24

3.2 [Ln{N(Si^{i}Pr$_3$)$_2$}(Cp‴)][Al{OC(CF$_3$)$_3$}$_4$] (2-Ln) S28

3.3 [Ln{N(Si^{i}Pr$_3$)$_2$}(Cp^{ttt})(BH$_4$)] (3-Ln) S32

3.4 [Ln{N(Si^{i}Pr$_3$)$_2$}(Cp‴)(BH$_4$)] (4-Ln) S36

4. NMR spectra S40

4.1 [Dy{N(Si^{i}Pr$_3$)$_2$}(Cp^{ttt})][Al{OC(CF$_3$)$_3$}$_4$] (1-Dy) S40

4.2 [Y{N(Si^{i}Pr$_3$)$_2$}(Cp^{ttt})][Al{OC(CF$_3$)$_3$}$_4$] (1-Y) S41

4.3 [Dy{N(Si^{i}Pr$_3$)$_2$}(Cp‴)][Al{OC(CF$_3$)$_3$}$_4$] (2-Dy) S42

4.4 [Y{N(Si^{i}Pr$_3$)$_2$}(Cp‴)][Al{OC(CF$_3$)$_3$}$_4$] (2-Y) S43

4.5 [Dy{N(Si^{i}Pr$_3$)$_2$}(Cp^{ttt})(BH$_4$)] (3-Dy) S44

4.6 [Y{N(Si^{i}Pr$_3$)$_2$}(Cp^{ttt})(BH$_4$)] (3-Y) S45

4.7 [Dy{N(Si^{i}Pr$_3$)$_2$}(Cp‴)(BH$_4$)] (4-Dy) S47

4.8 [Y{N(Si^{i}Pr$_3$)$_2$}(Cp‴)(BH$_4$)] (4-Y) S47

5. Magnetism data S50

6. Computational data S67

6.1 Static electronic structure calculations S67

6.2 Spin-phonon calculations S68

1. Single crystal X-ray diffraction

Table S1. Crystallographic data for **1-Ln** and **2-Ln**.

	**1-Dy**	**1-Y**	**2-Dy**	**2-Y**
Formula	$C_{51}H_{71}AlDyF_{36}NO_4Si_2$	$C_{51}H_{71}AlF_{36}NO_4Si_2Y$	$C_{48}H_{71}AlDyF_{36}NO_4Si_5$	$C_{48}H_{71}AlF_{36}NO_4Si_5Y$
Fw, g mol^{-1}	1691.74	1618.15	1739.98	1666.39
Crystal size, mm	0.24 × 0.17 × 0.13	0.28 × 0.16 × 0.15	0.58 × 0.26 × 0.25	0.351 × 0.141 × 0.122
Crystal system	monoclinic	monoclinic	orthorhombic	orthorhombic
Space group	$P2_1/n$	$P2_1/n$	$Pna2_1$	$Pna2_1$
Collection temperature, K	100.0(1)	100.0(1)	100.0(2)	100.0(1)
a, Å	17.21620(10)	17.18200(10)	20.19260(10)	20.2203(3)
b, Å	15.97840(10)	15.88710(10)	20.13180(10)	20.1297(3)
c, Å	49.7142(3)	49.6462(4)	17.03840(10)	17.0270(2)
α, °	90	90	90	90
β, °	98.3650(10)	98.2480(10)	90	90
γ, °	90	90	90	90
V, Å^3	13530.25(15)	13411.85(16)	6926.34(6)	6930.48(19)
Z	8	8	4	4
ρ_{calcd}, g cm^{-3}	1.661	1.603	1.669	1.597
μ, mm^{-1}	7.675	3.007	7.995	3.408
No. of reflections made	136210	109709	119047	31762
No. of unique reflns, R_{int}	29190, 0.0565	28352, 0.0540	14716, 0.0746	11276, 0.0550
No. of reflns with $F^2 > 2\sigma(F^2)$	25520	23876	14404	10376
Transmn coeff range	0.374–1.000	0.534–1.000	0.194–1.000	0.630–1.000
R, R_w [a] ($F^2 > 2\sigma(F^2)$)	0.0594, 0.1606	0.0628, 0.2030	0.0429, 0.1121	0.0414, 0.1110
R, R_w [a] (all data)	0.0663, 0.1661	0.0838, 0.2529	0.0434, 0.1124	0.0460, 0.1146
S [a]	1.022	1.146	1.085	1.033
Parameters, Restraints	2151, 5823	2132, 5547	887, 1	887, 1
Max., min. diff map, e Å^{-3}	1.140, –1.031	1.420, –1.841	1.230, –2.334	0.878, –0.925

[a] Conventional $R = \Sigma||F_o| - |F_c||/\Sigma|F_o|$; $R_w = [\Sigma w(F_o^2 - F_c^2)^2/\Sigma w(F_o^2)^2]^{1/2}$; $S = [\Sigma w(F_o^2 - F_c^2)^2/\text{no. data} - \text{no. params})]^{1/2}$ for all data.

Table S2. Crystallographic data for **3-Ln** and **4-Ln**.

	**3-Dy**	**3-Y**	**4-Dy**	**4-Y**
Formula	$C_{35}H_{75}BDyNSi_2$	$C_{35}H_{75}BNSi_2Y$	$C_{32}H_{75}BDyNSi_5$	$C_{32}H_{75}BNSi_5Y$
Fw, g mol^{-1}	739.45	665.86	787.69	714.10
Crystal size, mm	0.13 × 0.11 × 0.07	0.243 × 0.195 × 0.135	0.431 × 0.246 × 0.18	0.48 × 0.306 × 0.184
Crystal system	monoclinic	monoclinic	monoclinic	monoclinic
Space group	$C2/c$	$P2_1/n$	$P2_1/c$	$P2_1/c$
Collection temperature, K	100.0(1)	100.0(1)	100.0(2)	100.0(1)
a, Å	19.3432(3)	11.53410(10)	16.40592(11)	16.39870(13)
b, Å	8.68430(10)	14.57100(10)	20.04242(17)	20.06647(14)
c, Å	45.6513(5)	23.0010(2)	13.03305(9)	13.02654(10)
α, °	90	90	90	90
β, °	97.2210(10)	95.7750(10)	100.8976(7)	100.8460(7)
γ, °	90	90	90	90
V, Å^3	7607.78(17)	3846.01(5)	4208.17(5)	4209.99(6)
Z	8	4	4	4
ρ_{calcd}, g cm^{-3}	1.291	1.150	1.243	1.127
μ, mm^{-1}	11.257	2.876	10.997	3.448
No. of reflections made	53625	31992	41546	36993
No. of unique reflns, R_{int}	8224, 0.1170	8249, 0.0346	8764, 0.0317	8807, 0.0235
No. of reflns with $F^2 > 2\sigma(F^2)$	6513	7726	8218	8537
Transmn coeff range	0.716–1.000	0.478–1.000	0.269–0.817	0.537–1.000
R, R_w ^{a} ($F^2 > 2\sigma(F^2)$)	0.0418, 0.0918	0.0354, 0.0926	0.0259, 0.0711	0.0263, 0.0695
R, R_w ^{a} (all data)	0.0575, 0.0983	0.0373, 0.0939	0.0283, 0.0727	0.0272, 0.0701
S ^{a}	1.067	1.047	1.069	1.048
Parameters, Restraints	394, 19	394, 19	394, 19	394, 19
Max., min. diff map, e Å^{-3}	0.623, –0.827	1.054, –0.770	0.886, –0.558	0.503, –0.549

[a] Conventional $R = \Sigma||F_o| - |F_c||/\Sigma|F_o|$; $R_w = [\Sigma w(F_o^2 - F_c^2)^2/\Sigma w(F_o^2)^2]^{1/2}$; $S = [\Sigma w(F_o^2 - F_c^2)^2/\text{no. data} - \text{no. params})]^{1/2}$ for all data.

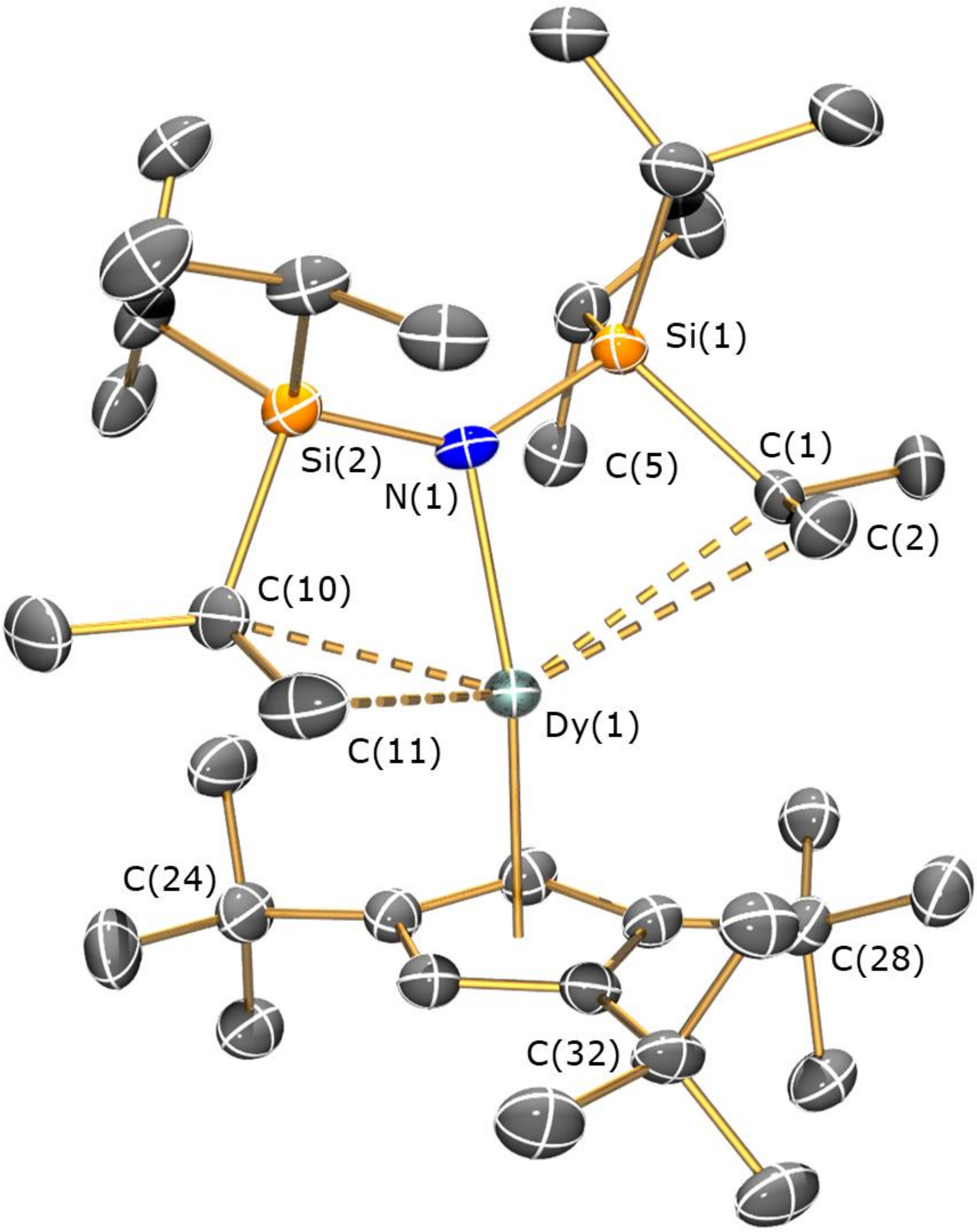

Figure S1. Single-crystal structure of **1-Dy**[a] (Z' = 2, cation A) collected at 100 K. Displacement ellipsoids set at 50% probability level. Hydrogen atoms, other cation, and $[Al\{OC(CF_3)_3\}_4]^-$ counter-anion omitted for clarity. ^{i}Pr carbon atoms within 3.3 Å of the Dy atom indicated with dashed bonding. Selected bond distances and angles tabulated in Table S3 below.

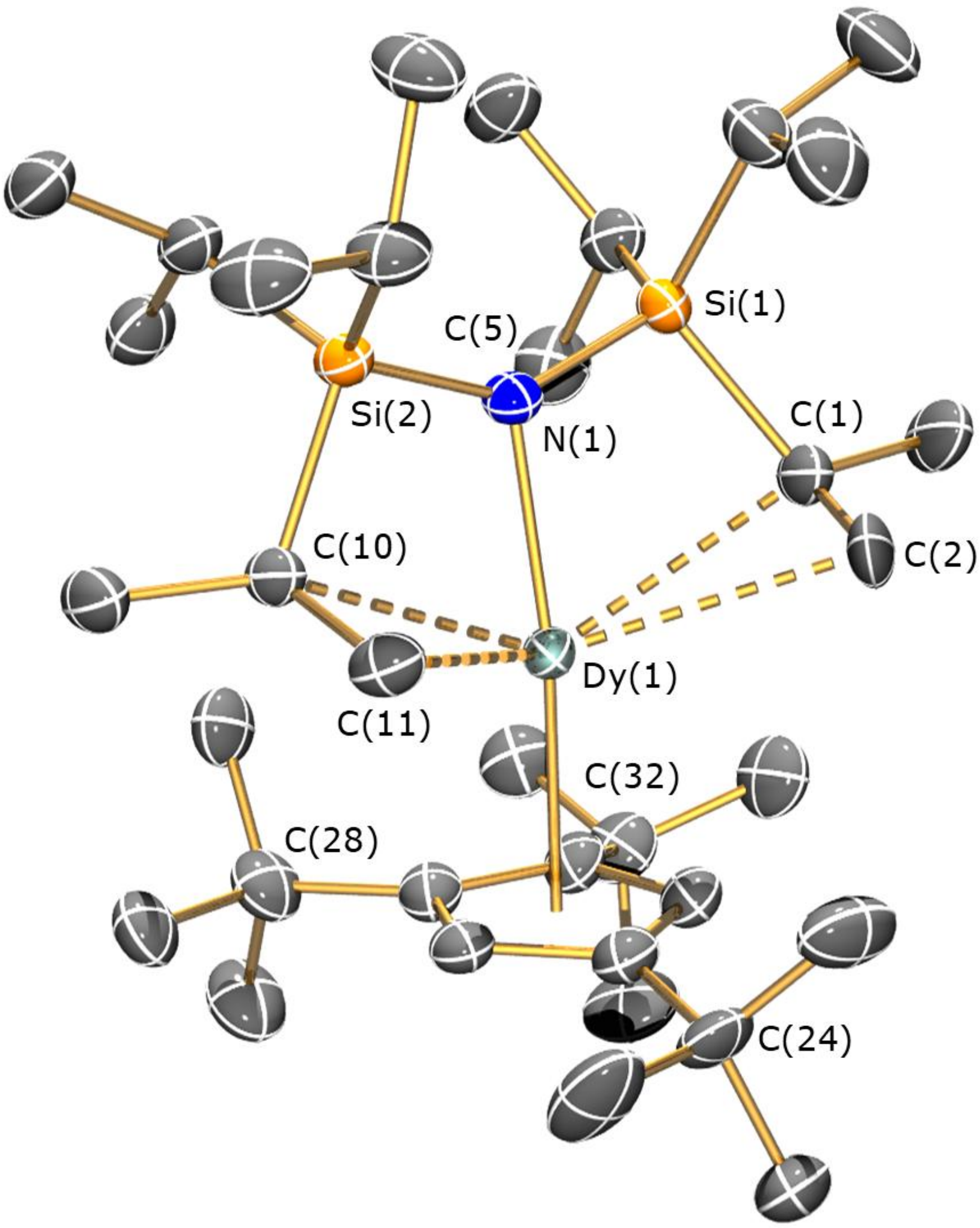

Figure S2. Single-crystal structure of **1-Dy**[b] (Z' = 2, cation B) collected at 100 K. Displacement ellipsoids set at 50% probability level. Hydrogen atoms, other cation, and $[Al\{OC(CF_3)_3\}_4]^-$ counter-anion omitted for clarity. ^{i}Pr carbon atoms within 3.3 Å of the Dy atom indicated with dashed bonding. Selected bond distances and angles tabulated in Table S3 below.

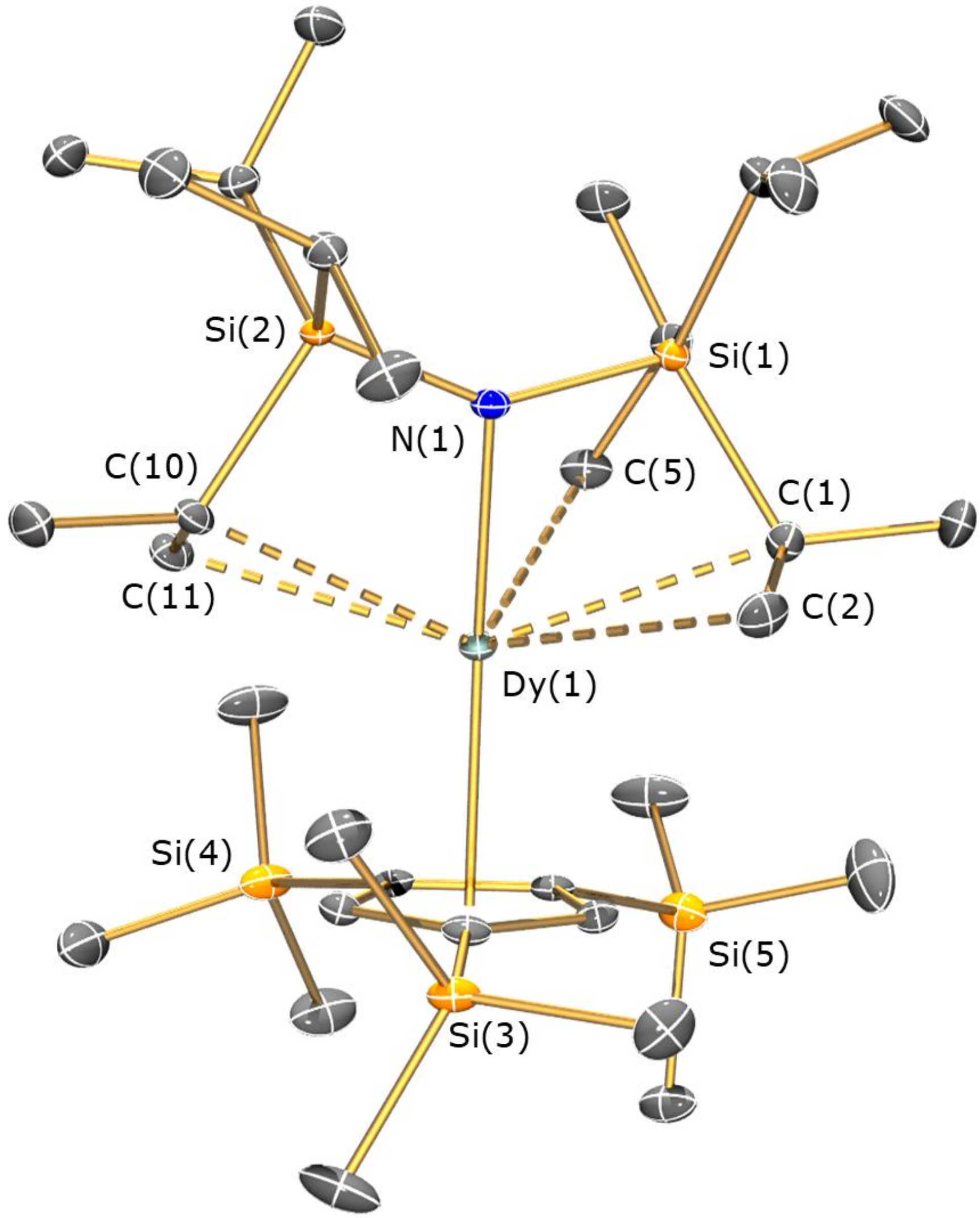

Figure S3. Single-crystal structure of **2-Dy** collected at 100 K. Displacement ellipsoids set at 50% probability level. Hydrogen atoms and $[Al\{OC(CF_3)_3\}_4]^-$ counter-anion. ^{i}Pr carbon atoms within 3.3 Å of the Dy atom indicated with dashed bonding. Selected bond distances and angles tabulated in Table S3 below.

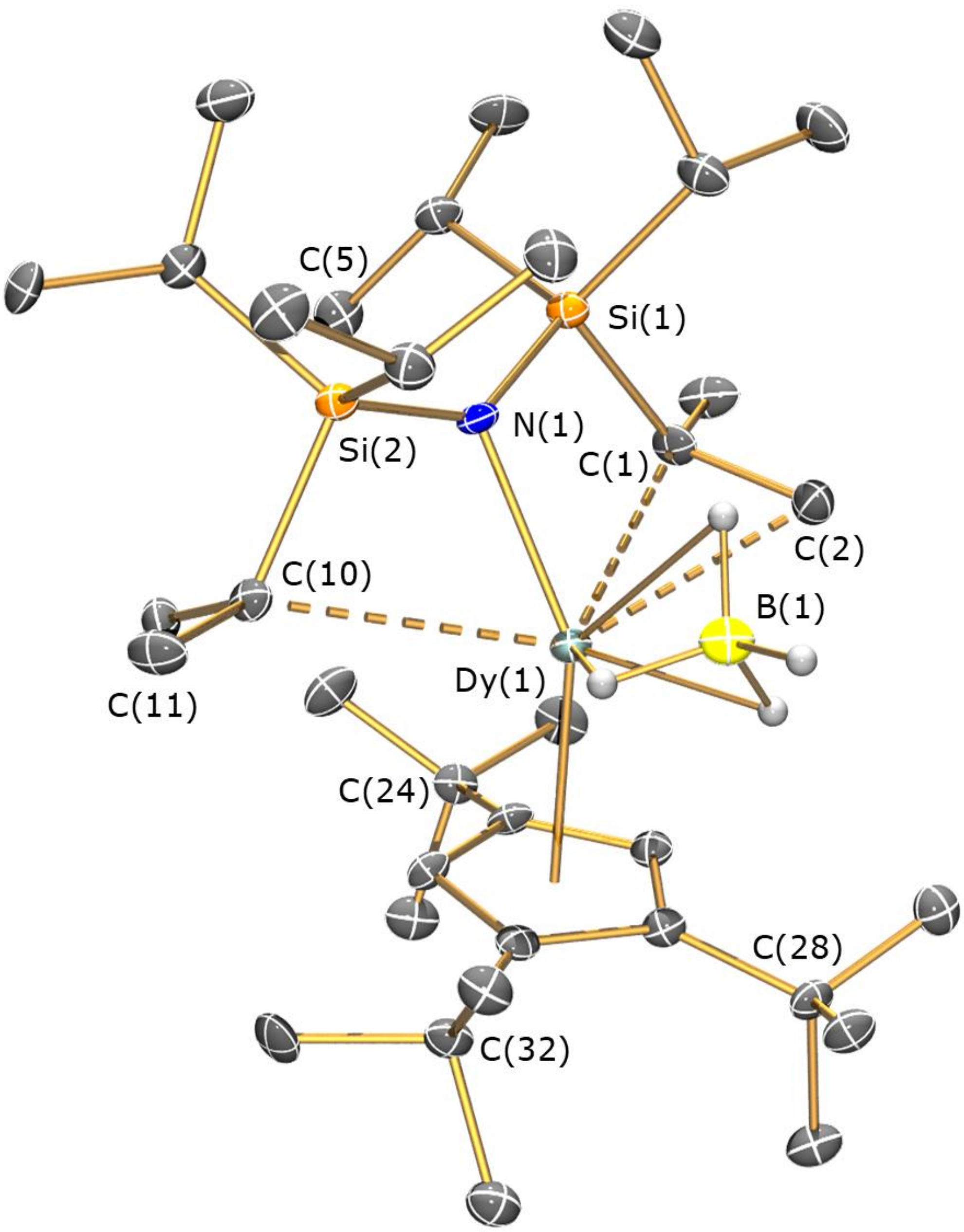

Figure S4. Single-crystal structure of **3-Dy** collected at 100 K. Displacement ellipsoids set at 50% probability level. Non-borohydride hydrogen atoms omitted for clarity. ^{i}Pr carbon atoms within 3.3 Å of the Dy atom indicated with dashed bonding. Selected bond distances and angles tabulated in Table S3 below.

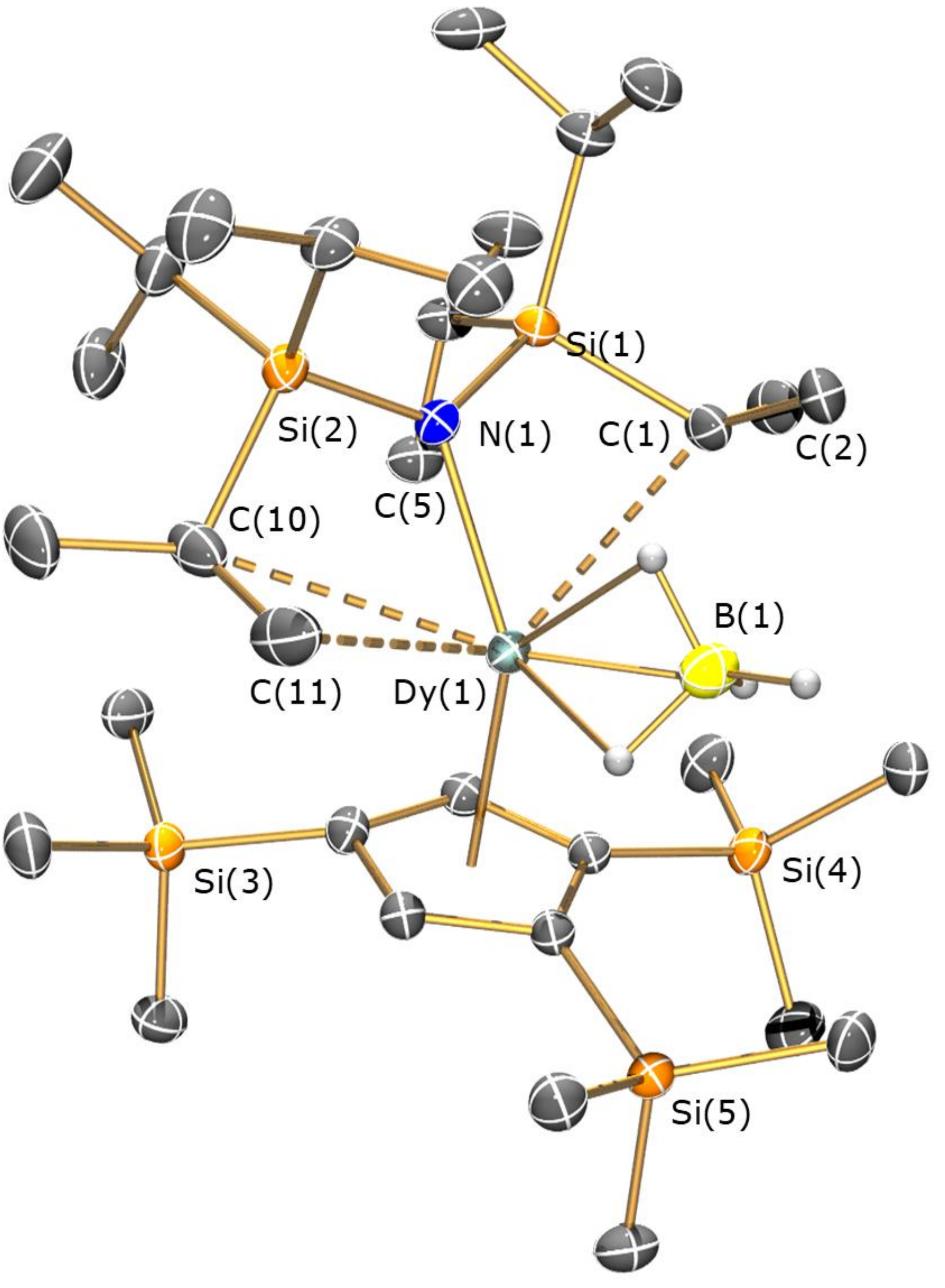

Figure S5. Single-crystal structure of **4-Dy** collected at 100 K. Displacement ellipsoids set at 50% probability level. Non-borohydride hydrogen atoms omitted for clarity. ^{i}Pr carbon atoms within 3.3 Å of the Dy atom indicated with dashed bonding. Selected bond distances and angles tabulated in Table S3 below.

Table S3. Crystallographically determined distances and angles for Dy complexes.

	1-Dy[a]	1-Dy[b]	2-Dy	3-Dy	4-Dy
Dy(1)–Cp_{cent} / Å	2.345(2)	2.339(2)	2.333(2)	2.4339(17)	2.3958(10)
Dy(1)–N(1) / Å	2.185(3)	2.209(3)	2.204(3)	2.259(3)	2.2486(18)
Dy(1)···B(1) / Å	-	-	-	2.500(5)	2.484(3)
N(1)–Dy(1)–Cp_{cent} / °	157.84(11)	167.90(10)	172.26(10)	147.79(10)	136.39(5)
B(1)···Dy(1)–Cp_{cent} / °	-	-	-	113.78(11)	113.55(7)
B(1)···Dy(1)–N(1) / °	-	-	-	96.78(13)	109.30(8)
Si(1)–N(1)–Si(2) / °	138.2(2)	141.6(2)	143.9(2)	138.2(2)	128.35(11)
Dy(1)···Si(1) / Å	3.2141(11)	3.2410(13)	3.2777(11)	3.3207(12)	3.3925(7)
Dy(1)···Si(2) / Å	3.2415(12)	3.1923(11)	3.0811(11)	3.3205(13)	3.3751(7)
Dy(1)···C(1) / Å	2.877(6)	2.832(4)	2.877(4)	2.941(4)	3.225(3)
Dy(1)···H(1a) / Å	2.3589(4)	2.3047(8)	2.3484(4)	2.4607(4)	2.8944(3)
Dy(1)···C(2) / Å	2.843(4)	2.867(5)	2.893(5)	2.970(4)	3.025(2)
Dy(1)···H(2a) / Å	2.3151(8)	2.3213(9)	2.3332(4)	2.4203(4)	2.3609(3)
Dy(1)···C(5) / Å	3.911(5) [c]	4.071(7) [c]	3.045(4)	4.766(4) [c]	4.169(2) [c]
Dy(1)···H(5a) / Å	3.213(1) [c]	3.5642(4) [c]	2.6069(4)	4.2514(6) [c]	3.5078(3) [c]
Dy(1)···H(5b) / Å	3.9188(8) [c]	3.8853(7) [c]	2.7056(4)	4.6962(5) [c]	4.1382(3) [c]
Dy(1)···C(10) / Å	2.906(3)	2.815(5)	2.874(5)	3.183(4)	3.247(2)
Dy(1)···H(10a) / Å	2.3184(4)	2.3048(7)	2.3837(3)	2.4299(3)	2.5316(3)
Dy(1)···C(11) / Å	3.209(5)	2.903(5)	3.072(5)	3.760(4) [c]	3.816(3) [c]
Dy(1)···H(11a) / Å	2.6458(6)	2.3561(3)	2.4425(4)	3.3544(3) [c]	3.4564(4) [c]
Cp–C(24) / Å	1.522(6)	1.524(7)	-	1.520(6)	-
Cp–C(28) / Å	1.535(7)	1.541(7)	-	1.539(6)	-
Cp–C(32) / Å	1.541(6)	1.550(7)	-	1.536(6)	-
Cp–Si(3) / Å	-	-	1.888(4)	-	1.873(2)
Cp–Si(4) / Å	-	-	1.892(4)	-	1.883(2)
Cp–Si(5) / Å	-	-	1.889(5)	-	1.879(2)
C(24)–Cp_{plane} / ° [d]	8.7(4)	8.7(4)	-	14.8(3)	-
C(28)–Cp_{plane} / ° [d]	14.4(4)	8.2(5)	-	12.6(3)	-
C(32)–Cp_{plane} / ° [d]	6.2(4)	16.5(4)	-	10.8(4)	-
Si(3)–Cp_{plane} / ° [d]	-	-	6.0(3)	-	22.3(2)
Si(4)–Cp_{plane} / ° [d]	-	-	9.5(3)	-	10.8(2)
Si(5)–Cp_{plane} / ° [d]	-	-	14.9(3)	-	13.2(2)
C(1)···Dy(1)–Cp_{cent} / °	112.92(10)	113.77(9)	114.27(10)	109.35(8)	108.60(5)
C(2)···Dy(1)–Cp_{cent} / °	106.57(13)	104.37(11)	103.29(10)	105.89(9)	109.41(6)
C(10)···Dy(1)–Cp_{cent} / °	118.14(11)	115.75(11)	111.80(10)	103.76(8)	114.45(4)
C(11)···Dy(1)–Cp_{cent} / °	121.45(10)	108.24(12)	102.00(9)	90.41(7)	126.78(4)
C(1)···Dy(1)···C(10) / °	128.76(13)	131.43(12)	127.25(12)	120.28(11)	120.25(6)
C(2)···Dy(1)···C(11) / °	104.27(15)	106.13(15)	144.46(12)	138.77(10)	122.88(6)

[a] Z' = 2, cation A. [b] Z' = 2, cation B. [c] Not a Dy-HC agostic contact. [d] Deflection from Cp plane of the Cp-C/Si bond.

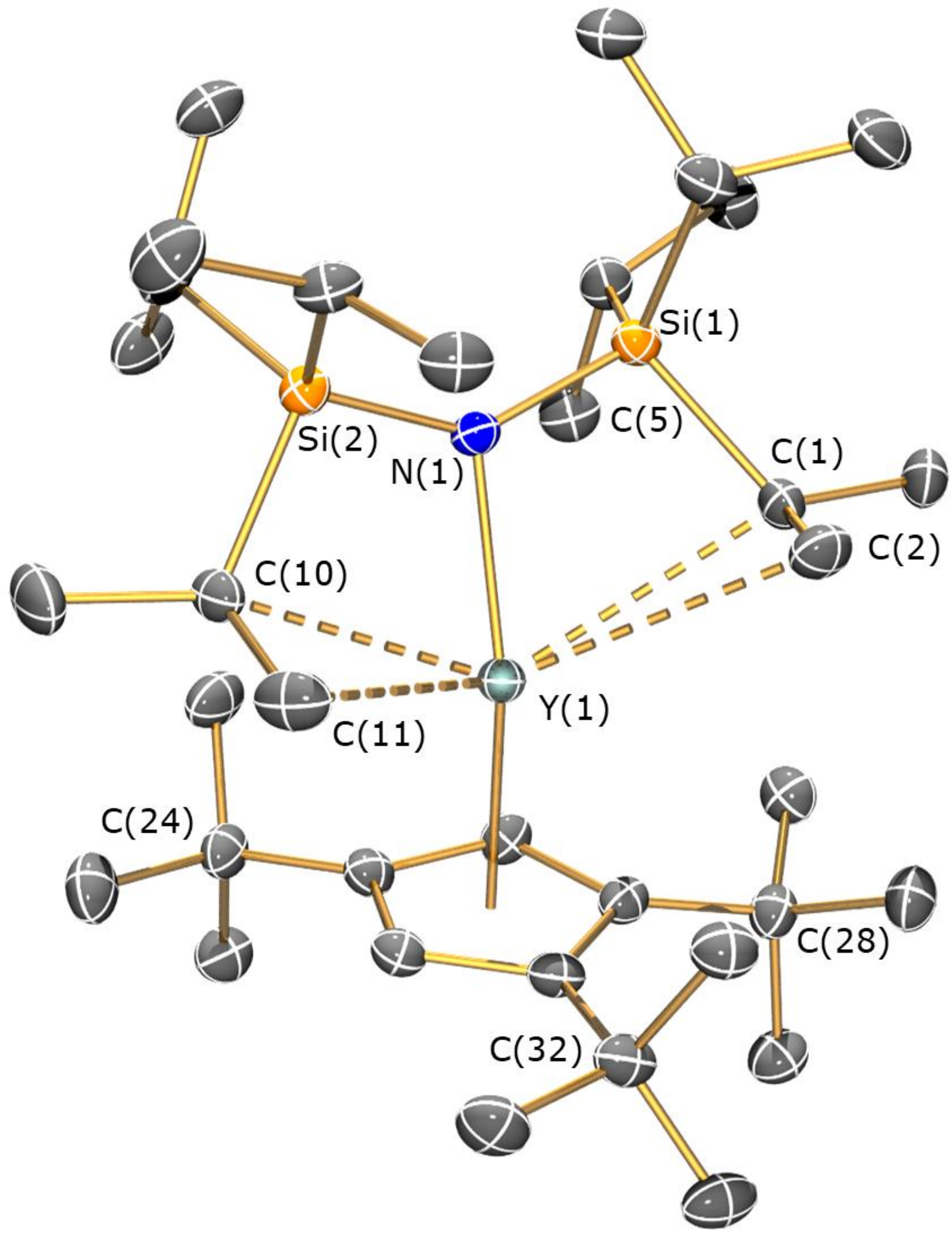

Figure S6. Single-crystal structure of **1-Y**[a] (Z' = 2, cation A) collected at 100 K. Displacement ellipsoids set at 50% probability level. Hydrogen atoms, other cation, and $[Al\{OC(CF_3)_3\}_4]^-$ counter-anion omitted for clarity. ^{i}Pr carbon atoms within 3.3 Å of the Y atom indicated with dashed bonding. Selected bond distances and angles tabulated in Table S4 below.

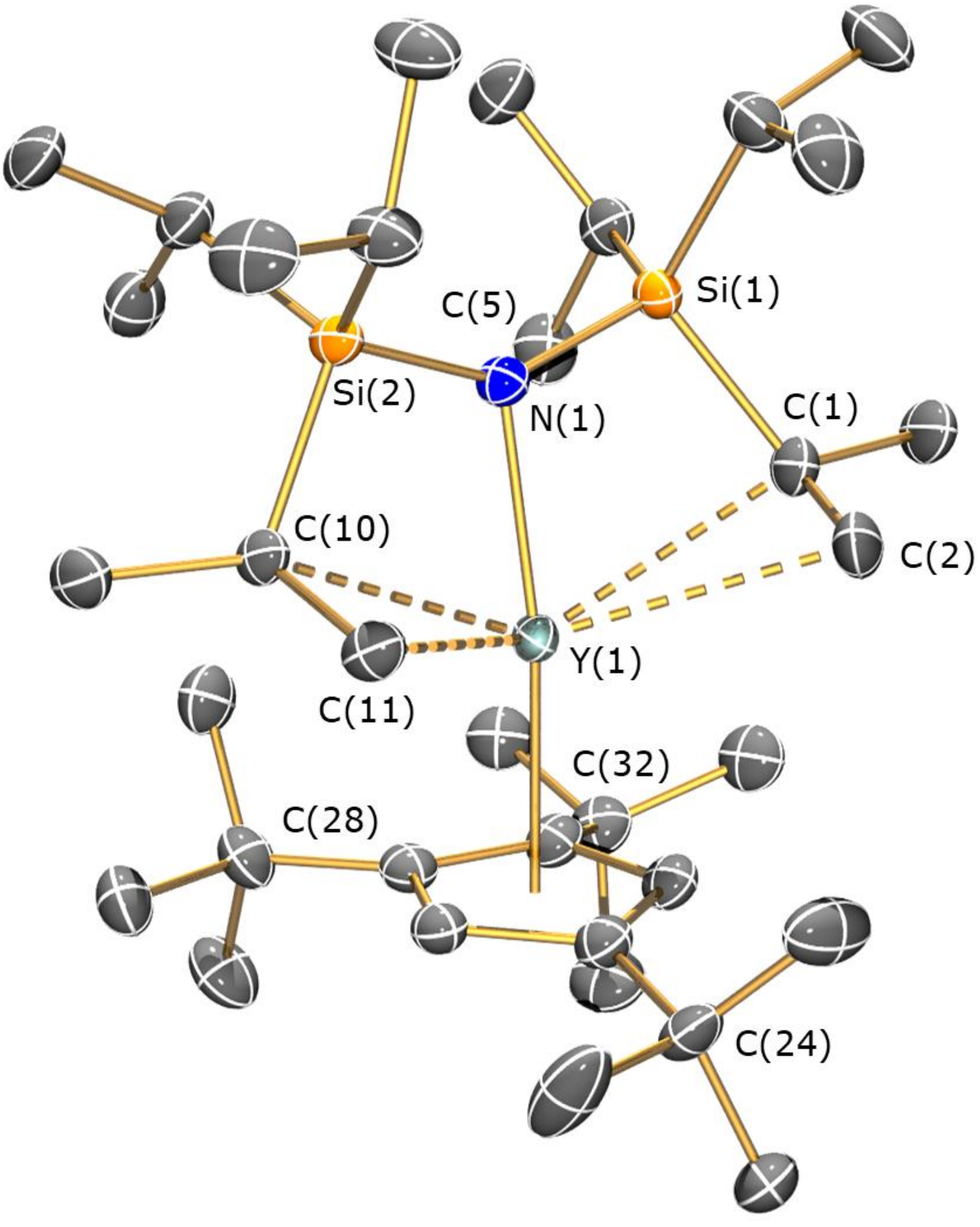

Figure S7. Single-crystal structure of **1-Y^b** (Z' = 2, cation B) collected at 100 K. Displacement ellipsoids set at 50% probability level. Hydrogen atoms, other cation, and $[Al\{OC(CF_3)_3\}_4]^-$ counter-anion omitted for clarity. ^{i}Pr carbon atoms within 3.3 Å of the Y atom indicated with dashed bonding. Selected bond distances and angles tabulated in Table S4 below.

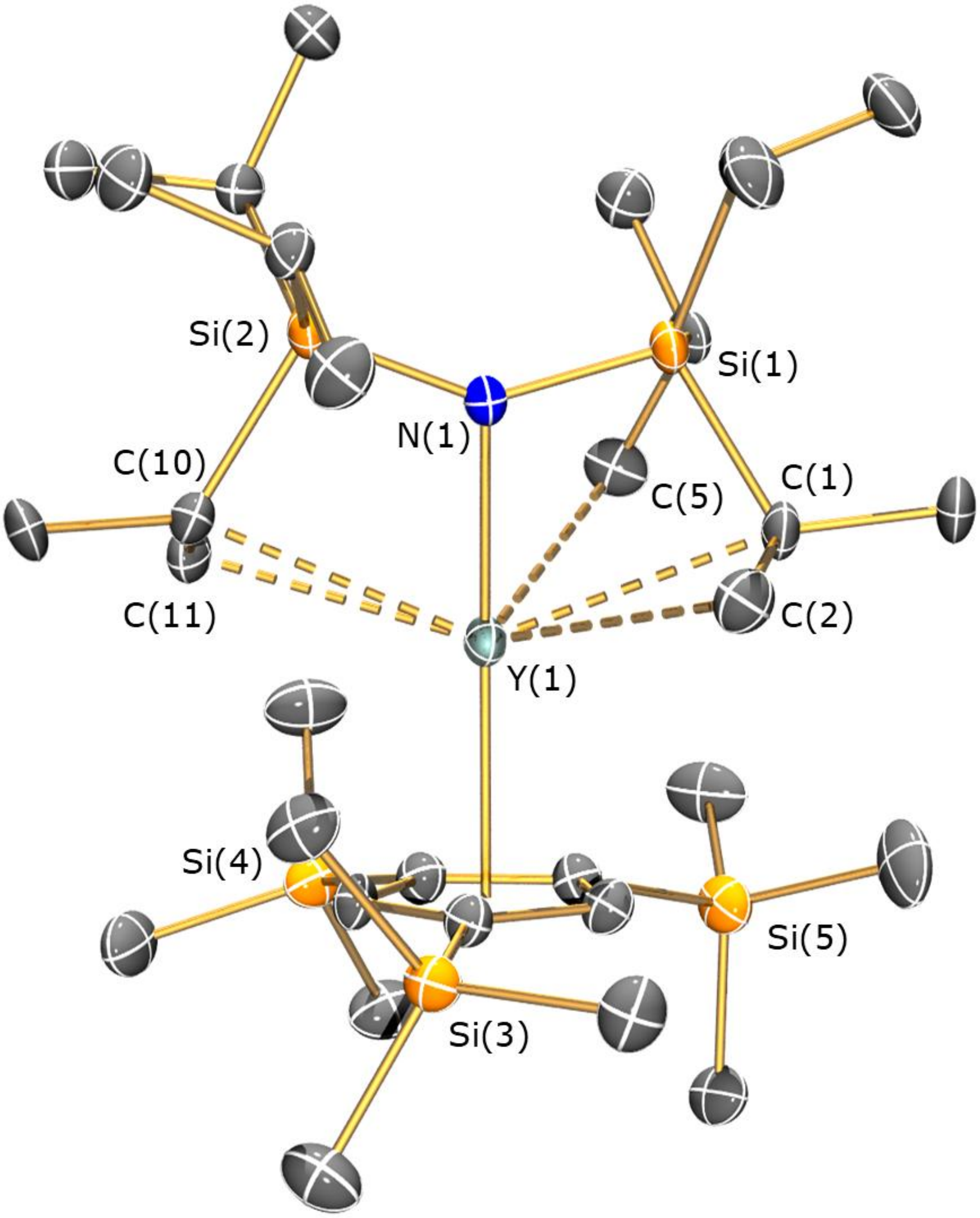

Figure S8. Single-crystal structure of **2-Y** collected at 100 K. Displacement ellipsoids set at 50% probability level. Hydrogen atoms and $[Al\{OC(CF_3)_3\}_4]^-$ counter-anion omitted for clarity. ^{i}Pr carbon atoms within 3.3 Å of the Y atom indicated with dashed bonding. Selected bond distances and angles tabulated in Table S4 below.

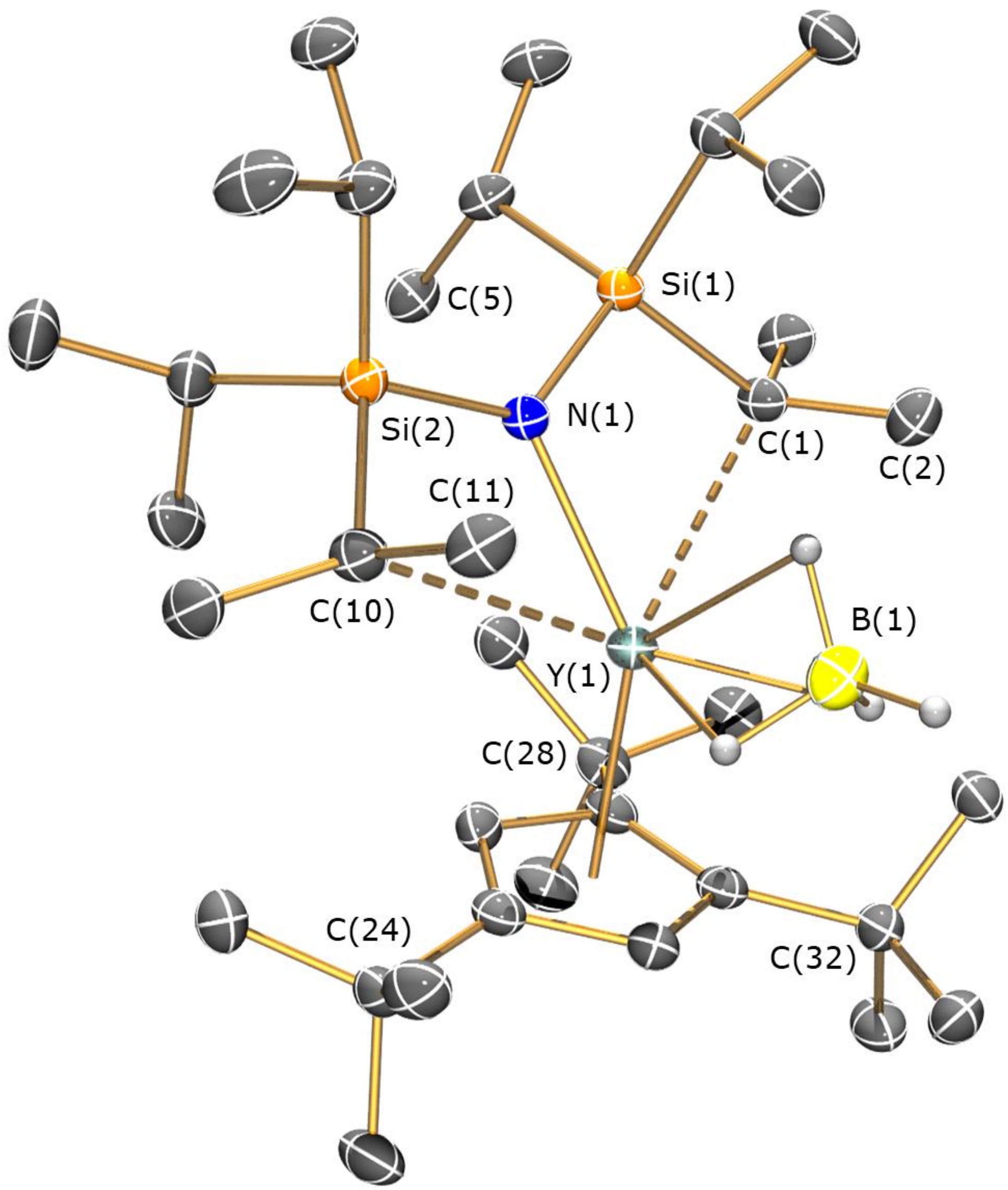

Figure S9. Single-crystal structure of **3-Y** collected at 100 K. Displacement ellipsoids set at 50% probability level. Non-borohydride hydrogen atoms omitted for clarity. ^{i}Pr carbon atoms within 3.3 Å of the Y atom indicated with dashed bonding. Selected bond distances and angles tabulated in Table S4 below.

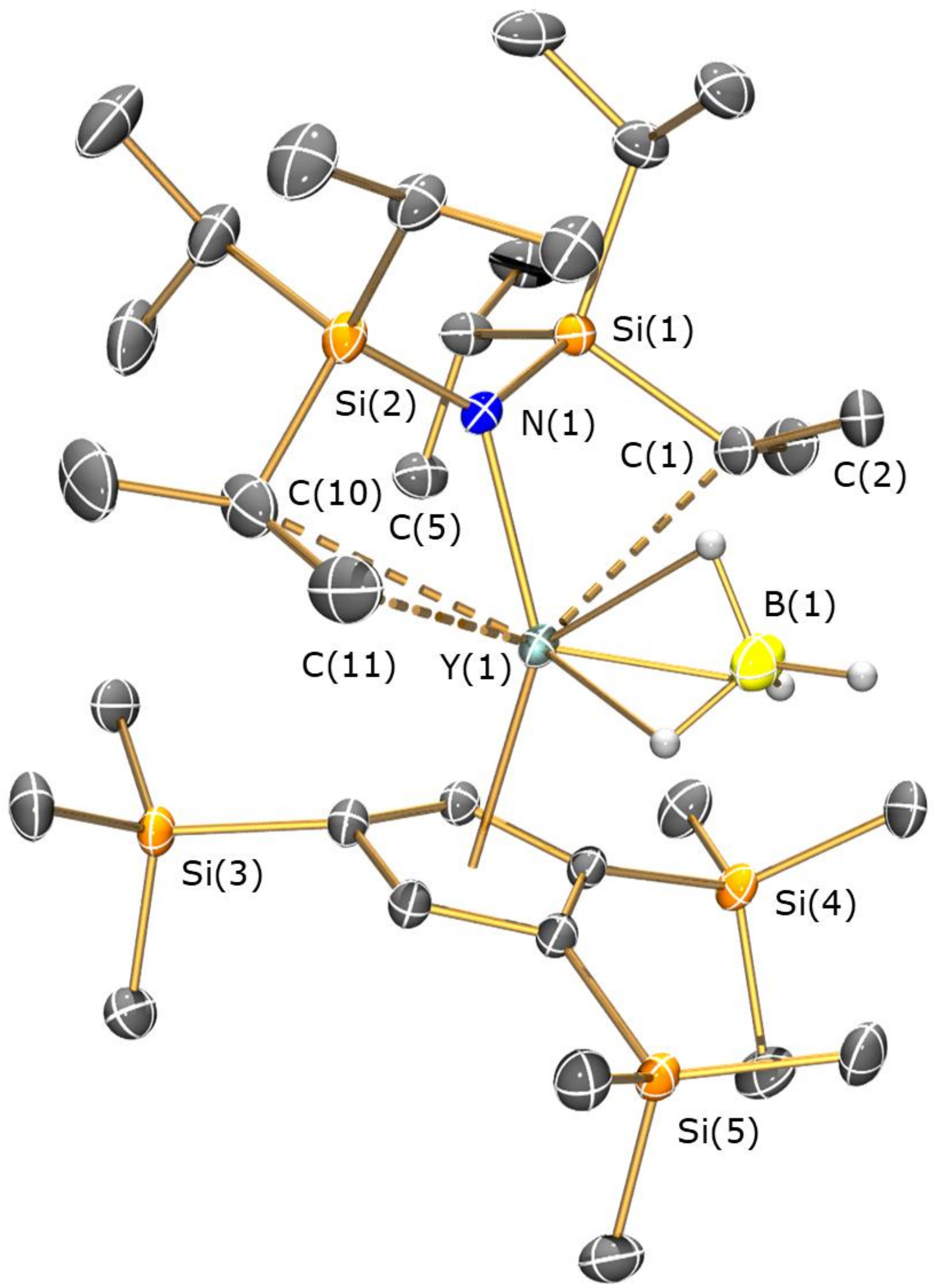

Figure S10. Single-crystal structure of **4-Y** collected at 100 K. Displacement ellipsoids set at 50% probability level. Non-borohydride hydrogen atoms omitted for clarity. ^{i}Pr carbon atoms within 3.3 Å of the Y atom indicated with dashed bonding. Selected bond distances and angles tabulated in Table S4 below.

Table S4. Crystallographically determined distances and angles for Y complexes.

	1-Y[a]	1-Y[b]	2-Y	3-Y	4-Y
Y(1)–Cp_{cent} / Å	2.3322(15)	2.3272(16)	2.319(2)	2.3964(9)	2.3836(7)
Y(1)–N(1) / Å	2.198(3)	2.216(3)	2.206(4)	2.2622(14)	2.2546(13)
Y(1)···B(1) / Å	-	-	-	2.495(3)	2.477(2)
N(1)–Y(1)–Cp_{cent} / °	156.65(10)	166.27(9)	172.06(12)	139.60(5)	135.39(4)
B(1)···Y(1)–Cp_{cent} / °	-	-	-	109.84(6)	114.38(5)
B(1)···Y(1)–N(1) / °	-	-	-	109.79(7)	109.37(6)
Si(1)–N(1)–Si(2) / °	138.64(18)	142.05(18)	143.7(3)	125.98(8)	128.20(8)
Y(1)···Si(1) / Å	3.2075(9)	3.1895(9)	3.2747(14)	3.3471(6)	3.3908(6)
Y(1)···Si(2) / Å	3.2456(10)	3.2354(13)	3.0861(15)	3.4872(5)	3.3848(6)
Y(1)···C(1) / Å	2.815(3)	2.807(4)	2.882(5)	3.166(2)	3.224(2)
Y(1)···H(1a) / Å	2.3405(4)	2.2968(7)	2.3503(5)	2.5630(3)	2.8994(3)
Y(1)···C(2) / Å	2.851(4)	2.876(3)	2.886(5)	3.509(2) [c]	3.007(2)
Y(1)···H(2a) / Å	2.3494(7)	2.3127(3)	2.2969(5)	2.9858(4) [c]	2.3759(3)
Y(1)···C(5) / Å	3.945(4) [c]	4.090(5) [c]	3.034(6)	4.361(2) [c]	4.199(2) [c]
Y(1)···H(5a) / Å	4.2153(9) [c]	3.6816(4) [c]	2.5558(5)	3.6339(3) [c]	3.5426(3) [c]
Y(1)···H(5b) / Å	4.5091(7) [c]	3.7959(7) [c]	2.7358(4)	4.4582(3) [c]	4.1670(3) [c]
Y(1)···C(10) / Å	2.954(3)	2.816(4)	2.872(5)	3.361(2)	3.260(2)
Y(1)···H(10a) / Å	2.3165(4)	2.2944(7)	2.4969(4)	2.6723(2)	2.5453(3)
Y(1)···C(11) / Å	3.200(5)	2.840(4)	3.091(6)	3.734(2) [c]	3.818(2) [c]
Y(1)···H(11a) / Å	2.6080(6)	2.2760(9)	2.3694(5)	3.3177(3) [c]	3.4615(4) [c]
Cp–C(24) / Å	1.524(5)	1.518(6)	-	1.526(3)	-
Cp–C(28) / Å	1.538(5)	1.536(5)	-	1.538(3)	-
Cp–C(32) / Å	1.544(5)	1.545(6)	-	1.541(3)	-
Cp–Si(3) / Å	-	-	1.889(6)	-	1.872(2)
Cp–Si(4) / Å	-	-	1.887(6)	-	1.883(2)
Cp–Si(5) / Å	-	-	1.892(6)	-	1.877(2)
C(24)–Cp_{plane} / ° [d]	9.0(3)	8.8(3)	-	12.0(2)	-
C(28)–Cp_{plane} / ° [d]	15.1(3)	7.7(4)	-	15.7(2)	-
C(32)–Cp_{plane} / ° [d]	7.0(3)	17.5(3)	-	9.2(2)	-
Si(3)–Cp_{plane} / ° [d]	-	-	6.7(4)	-	22.81(11)
Si(4)–Cp_{plane} / ° [d]	-	-	9.9(5)	-	11.02(11)
Si(5)–Cp_{plane} / ° [d]	-	-	15.3(3)	-	12.68(11)
C(1)···Y(1)–Cp_{cent} / °	113.17(8)	114.48(9)	114.33(13)	115.81(4)	108.49(4)
C(2)···Y(1)–Cp_{cent} / °	107.04(9)	108.47(9)	103.59(13)	119.37(4)	109.66(4)
C(10)···Y(1)–Cp_{cent} / °	117.71(8)	113.75(8)	111.78(13)	114.70(4)	114.26(3)
C(11)···Y(1)–Cp_{cent} / °	121.32(7)	104.65(8)	102.02(13)	124.76(4)	126.85(3)
C(1)···Y(1)···C(10) / °	128.85(9)	131.77(10)	126.90(17)	118.95(5)	119.89(5)
C(2)···Y(1)···C(11) / °	104.40(12)	106.67(12)	143.91(17)	113.32(5)	122.75(5)

[a] Z' = 2, cation A. [b] Z' = 2, cation B. [c] Not a Y-HC agostic contact. [d] Deflection from Cp plane of the Cp-C/Si bond.

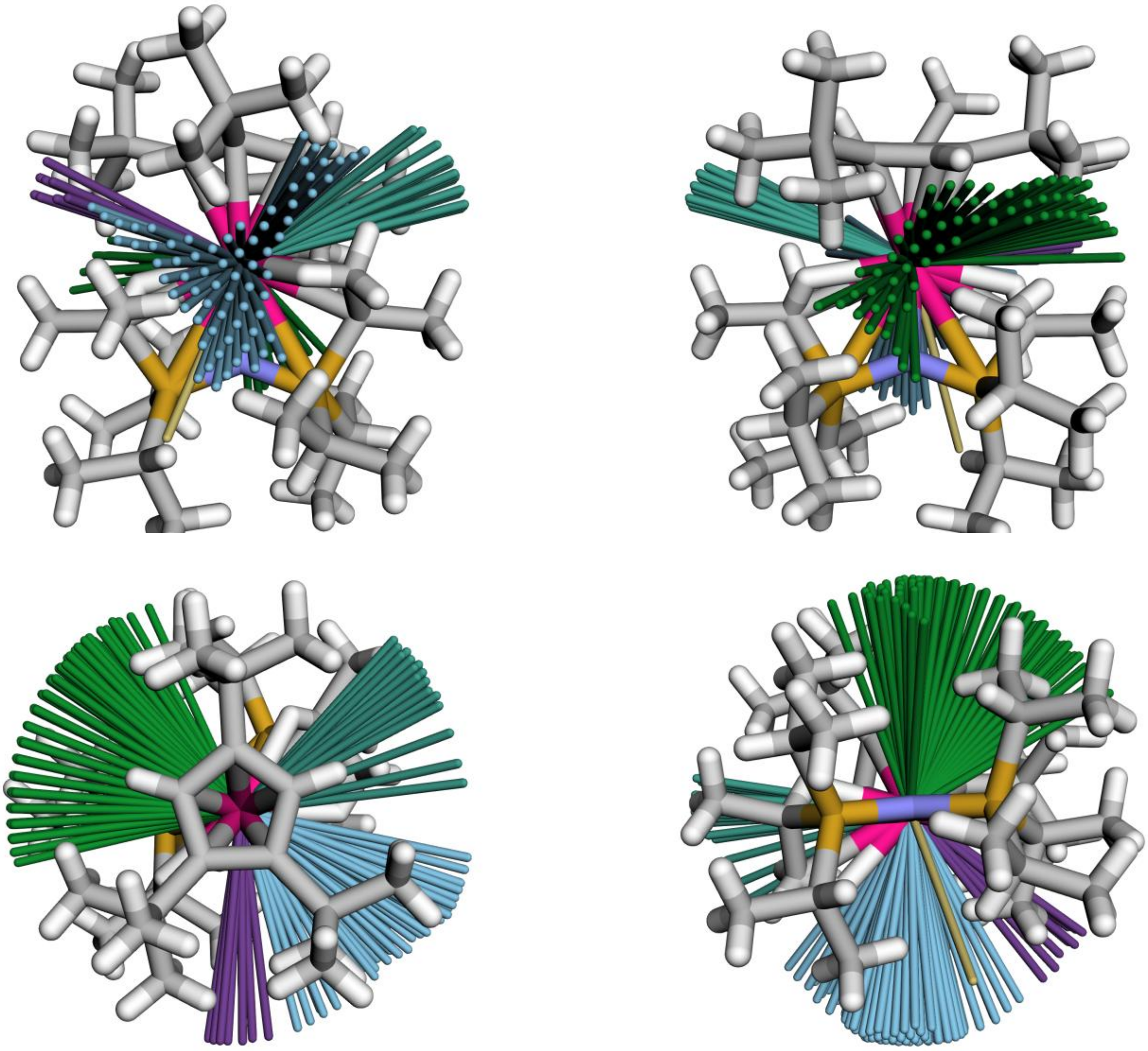

Figure S11. Orthogonal views of the accessible sites in the cation of the crystallographically determined structure of **1-Dy**[a] (Z' = 2, cation A) as measured by the *AtomAccess* solid angle metric. Dy: pink, N: blue, Si: orange, C: grey, H: white. Total: 6.192%; largest clusters: light blue: 2.748%, green 2.477%, Cyan 0.619%, Purple 0.310%, gold: 0.039%.

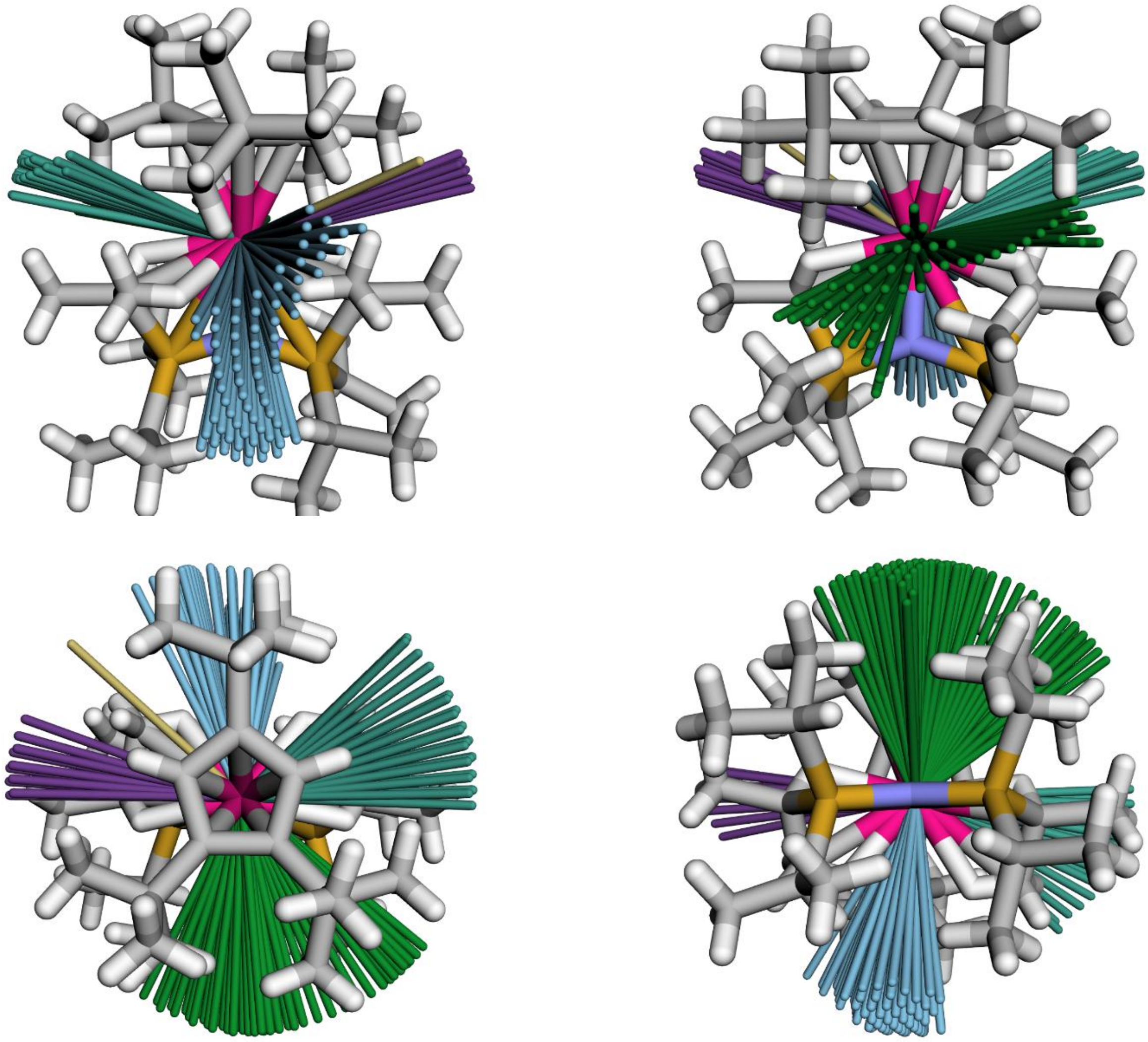

Figure S12. Orthogonal views of the accessible sites in the cation of the crystallographically determined structure of **1-Dy** (Z' = 2, cation B) as measured by the *AtomAccess* solid angle metric. Dy: pink, N: blue, Si: orange, C: grey, H: white. Total: 6.192%; largest clusters: light blue: 2.825%, green 2.090%, Cyan 0.851%, Purple 0.387%, gold: 0.039%.

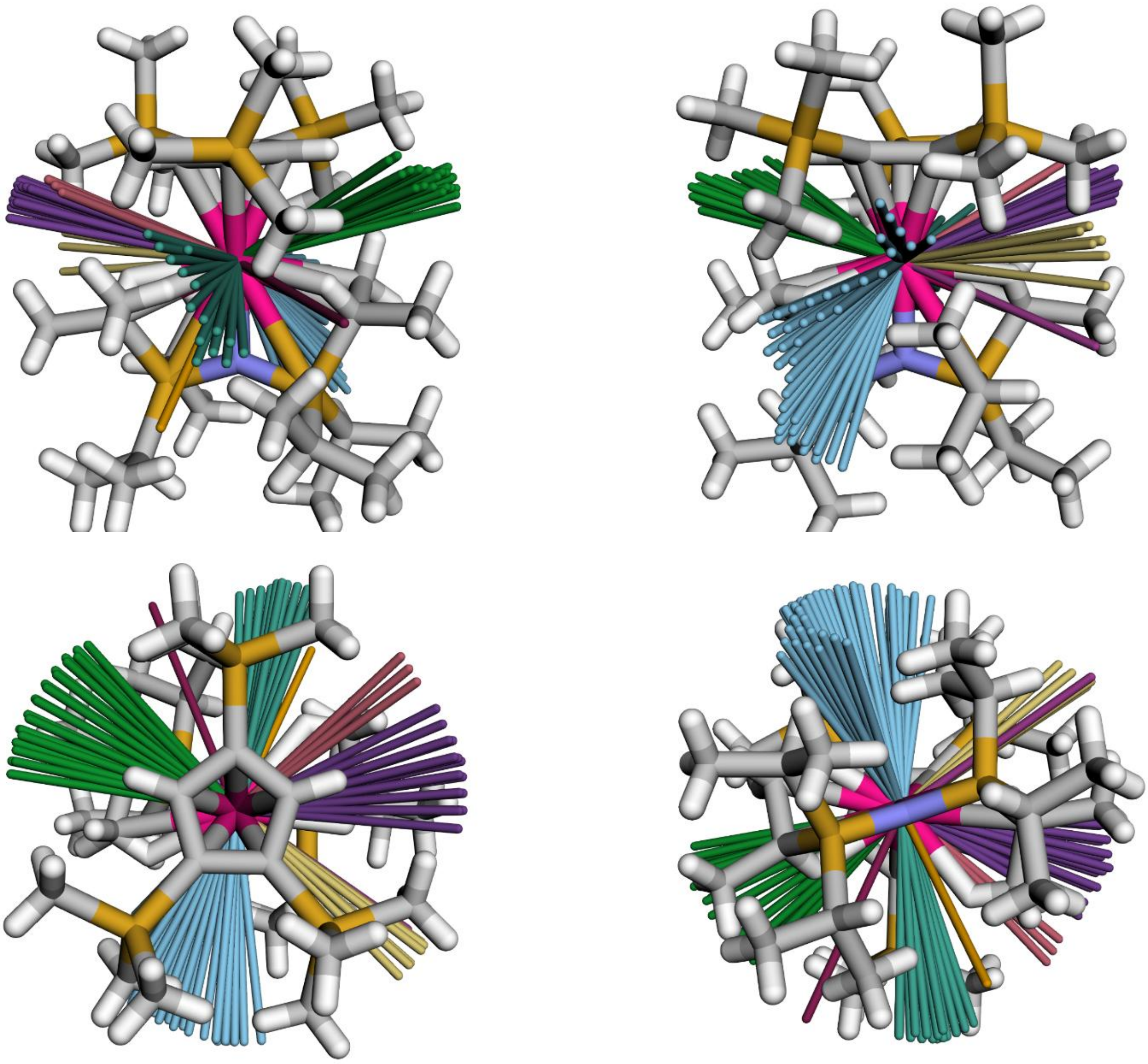

Figure S13. Orthogonal views of the accessible sites in the cation of the crystallographically determined structure of **2-Dy** as measured by the *AtomAccess* solid angle metric. Dy: pink, N: blue, Si: orange, C: grey, H: white. Total: 3.947%; largest clusters: light blue 1.580%, Green 0.813%, Cyan 0.580%, Purple 0.542%, Gold: 0.232%, Red: 0.116%, Lilac: 0.039%, Brown: 0.039%, orange: 0.039%.

2. Powder X-ray diffraction

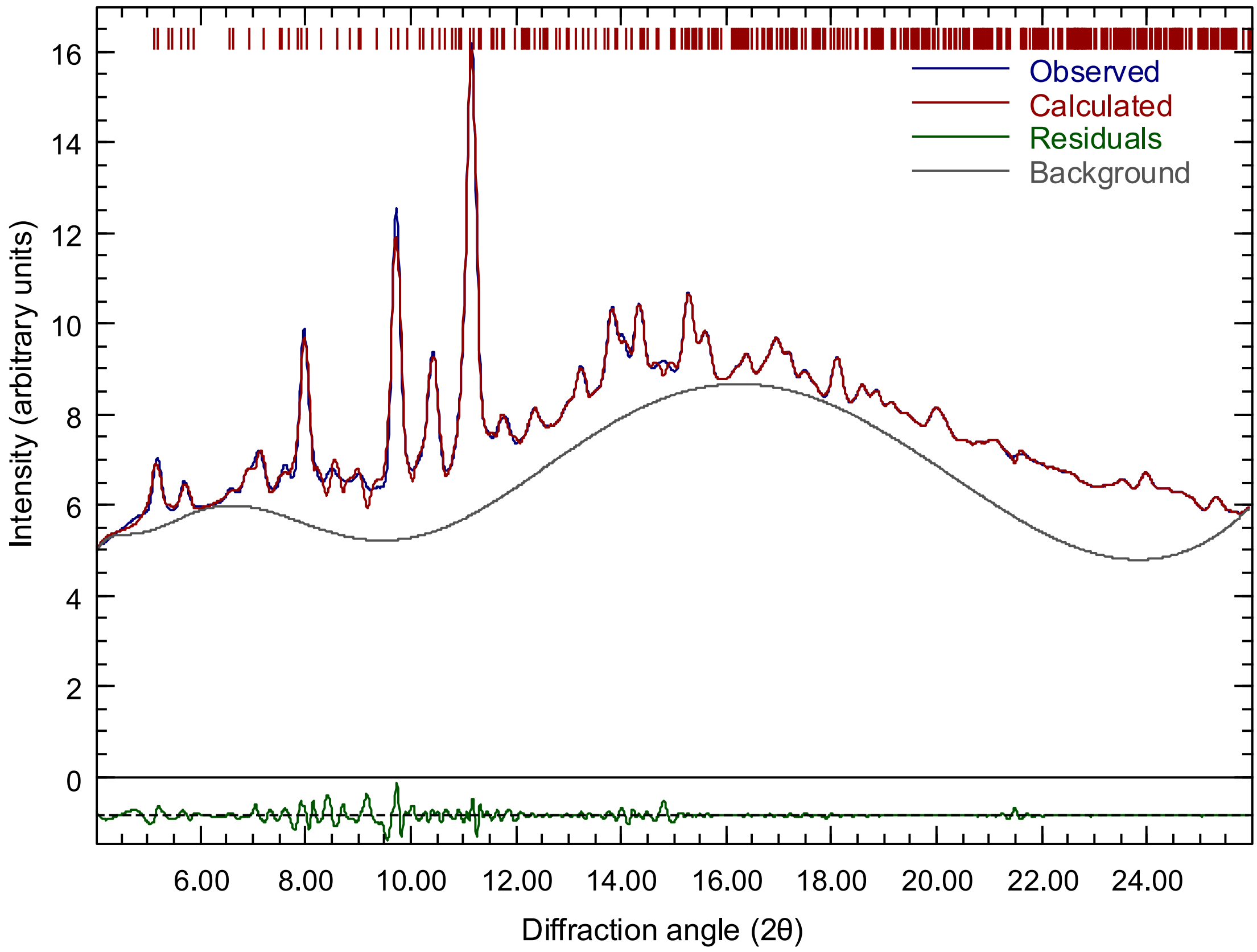

Figure S14. Powder XRD pattern of **1-Dy** in the range used for Le Bail profile fitting. Rwp = 1.30%, Rexp = 32.61%, χ^2 = 0.002, GoF = 0.040.

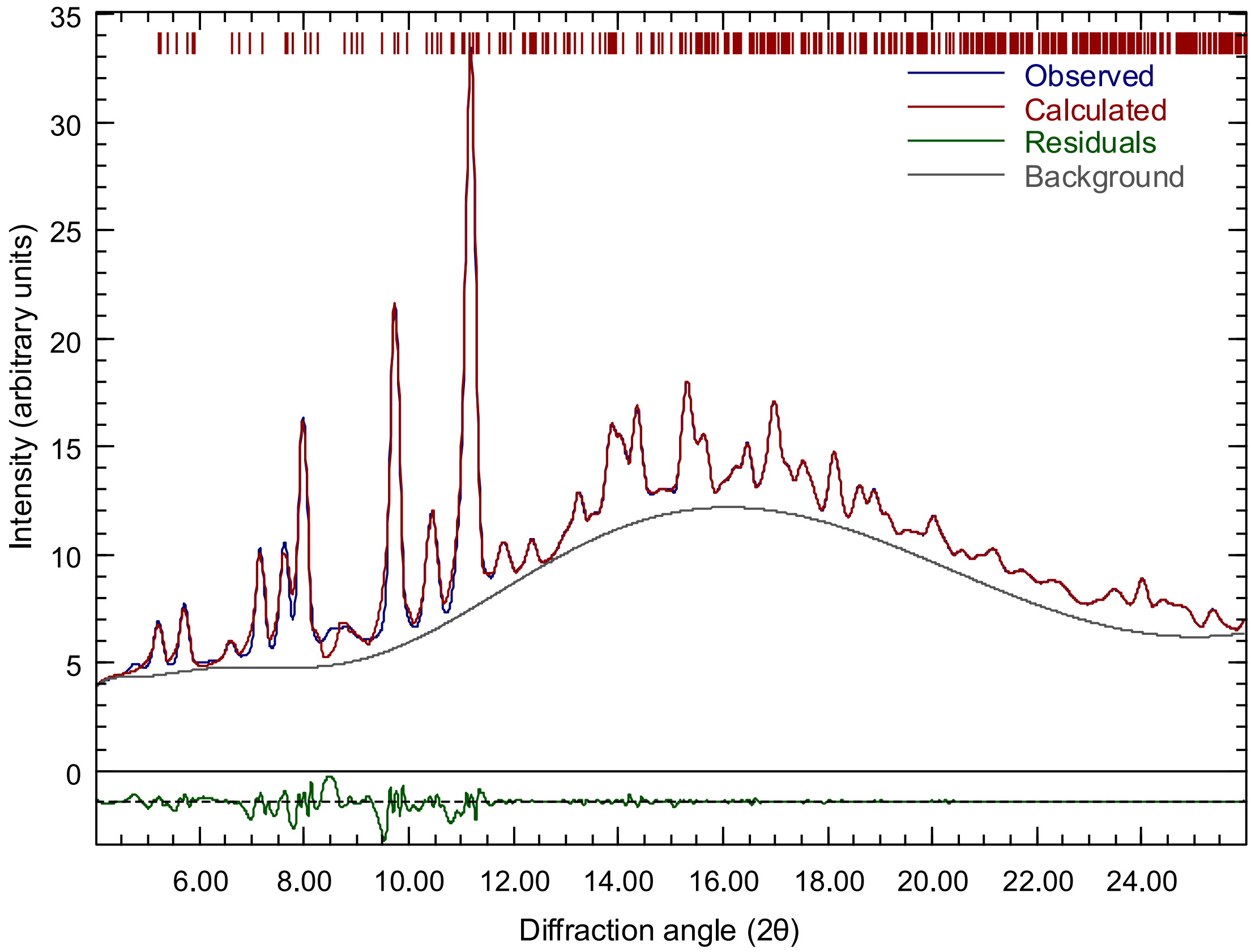

Figure S15. Powder XRD pattern of **1-Y** in the range used for Le Bail profile fitting. Rwp = 1.04%, Rexp = 0.39%, χ^2 = 7.111, GoF = 2.667.

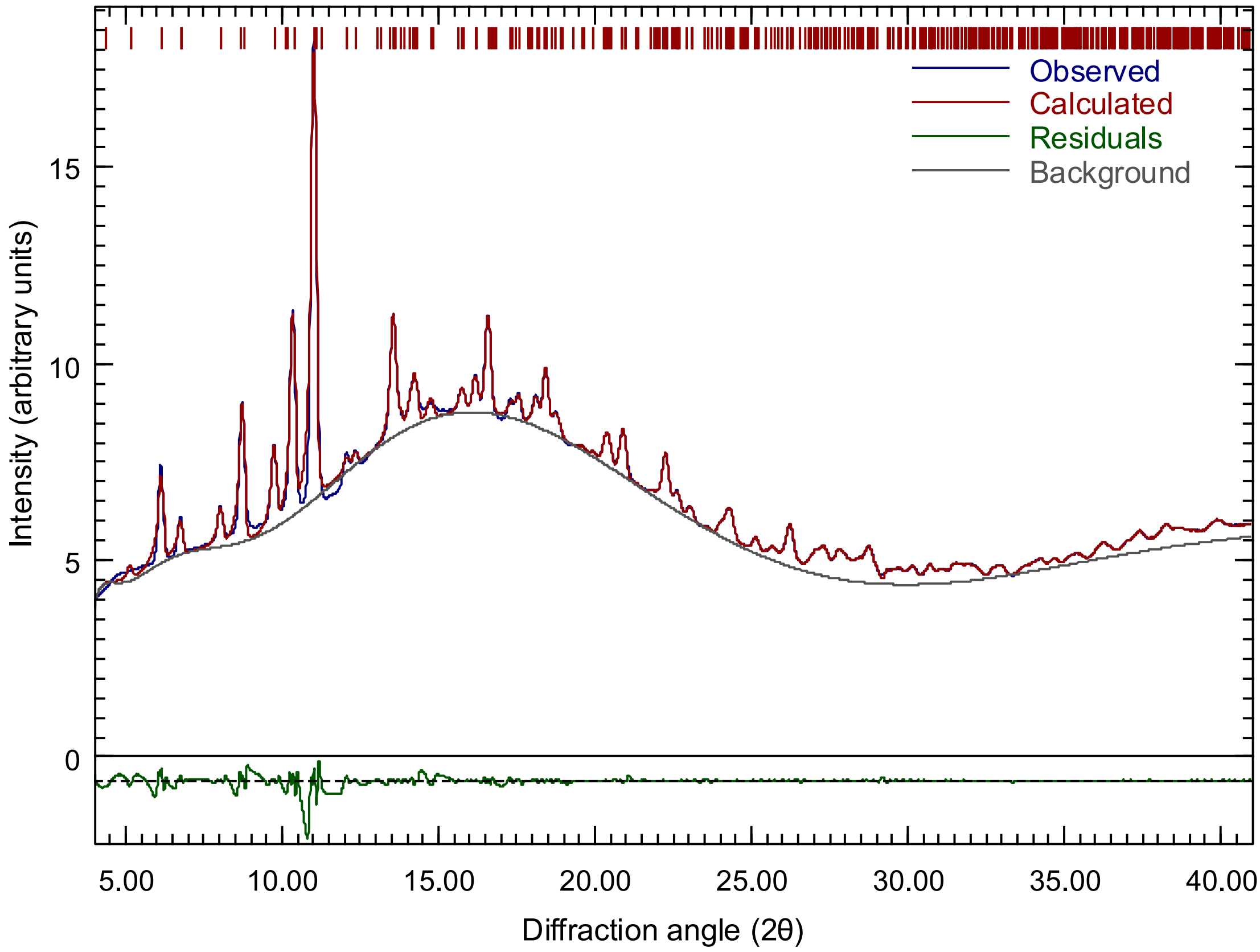

Figure S16. Powder XRD pattern of **2-Dy** in the range used for Le Bail profile fitting. wRp = 0.93%, Rexp = 0.47%, χ^2 = 3.915, GoF = 1.979.

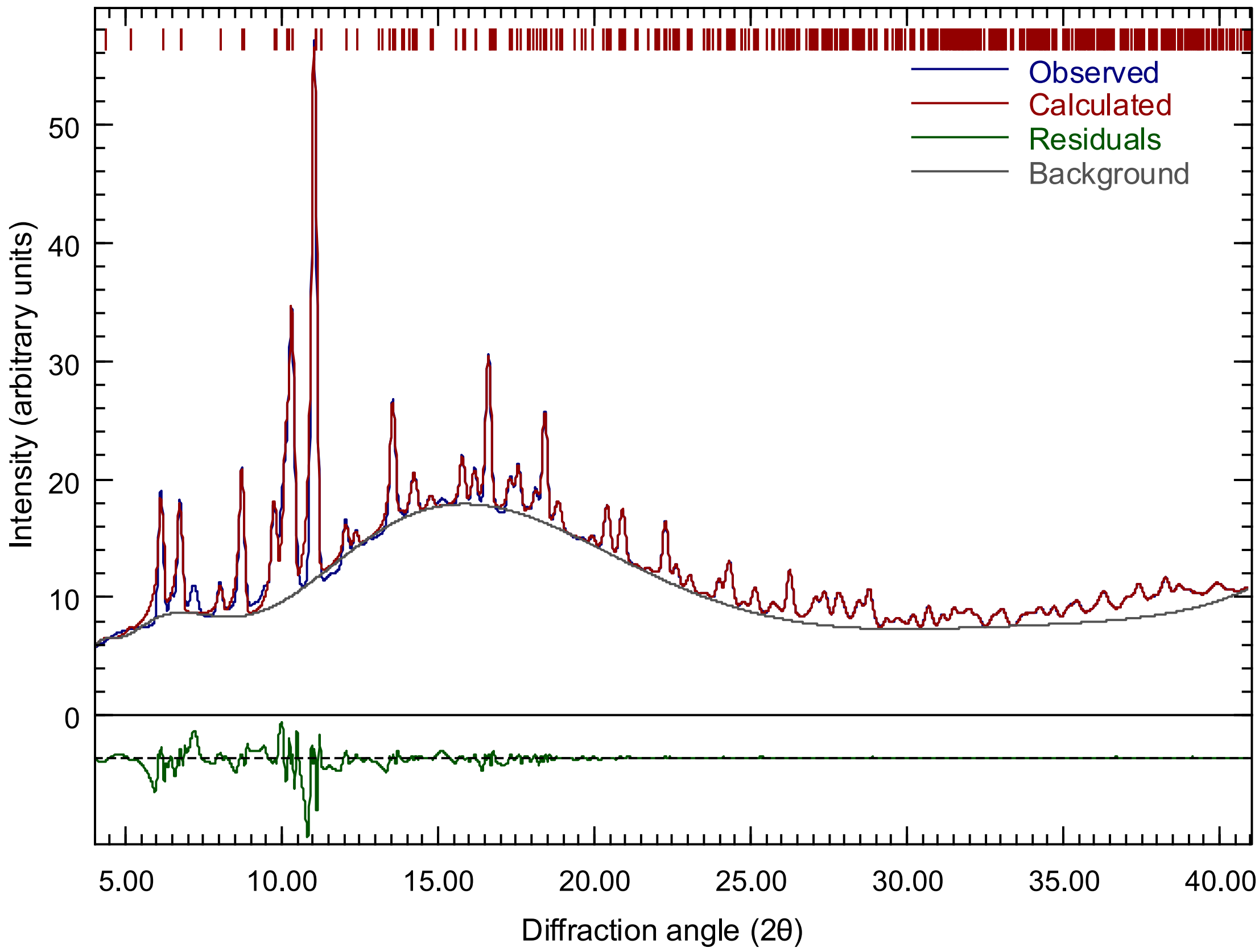

Figure S17. Powder XRD pattern of **2-Y** in the range used for Le Bail profile fitting. Rwp = 1.48%, Rexp = 0.40%, χ^2 = 13.690, GoF = 3.700.

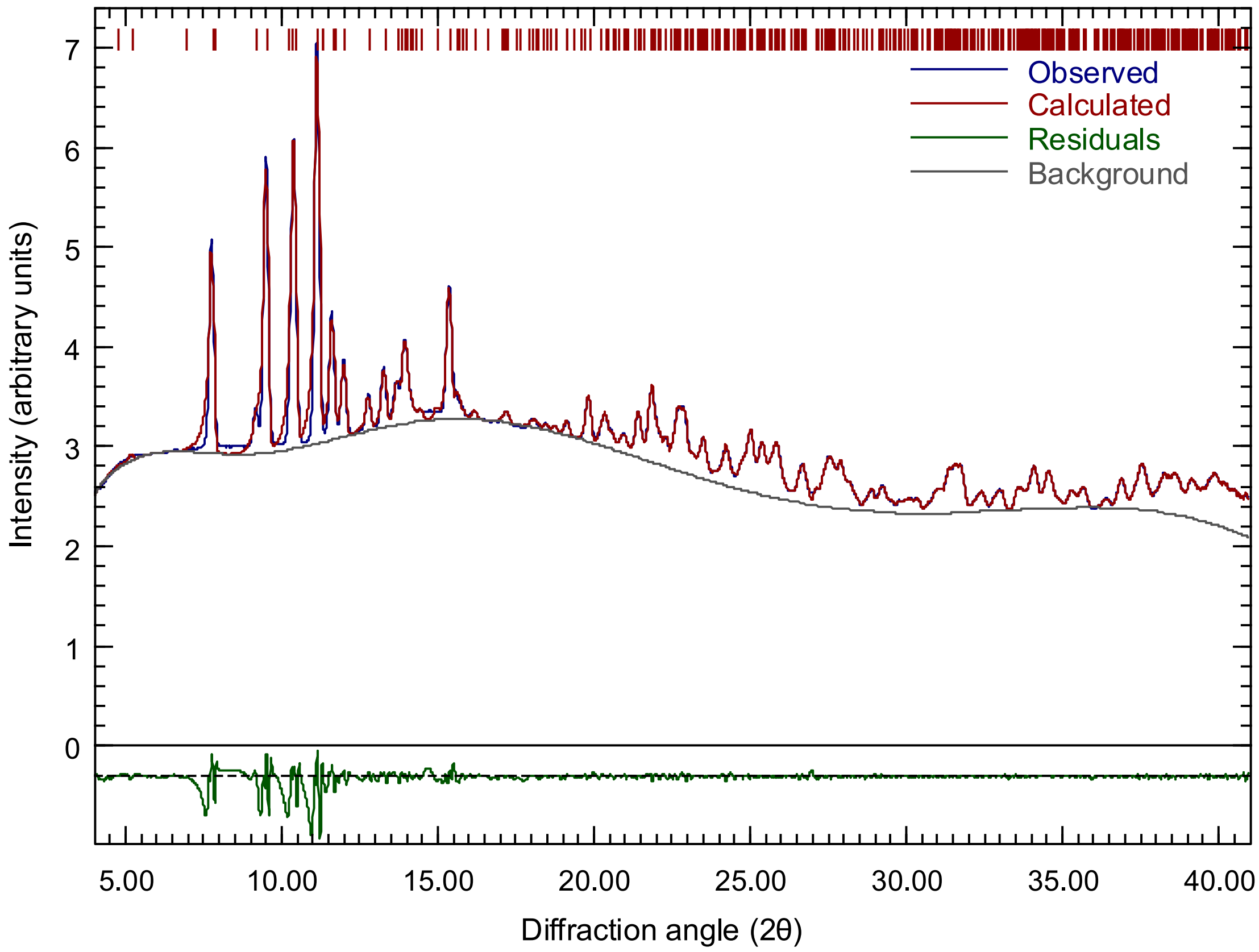

Figure S18. Powder XRD pattern of **3-Dy** in the range used for Le Bail profile fitting. Rwp = 1.39%, Rexp = 0.67%, χ^2 = 4.304, GoF = 2.075.

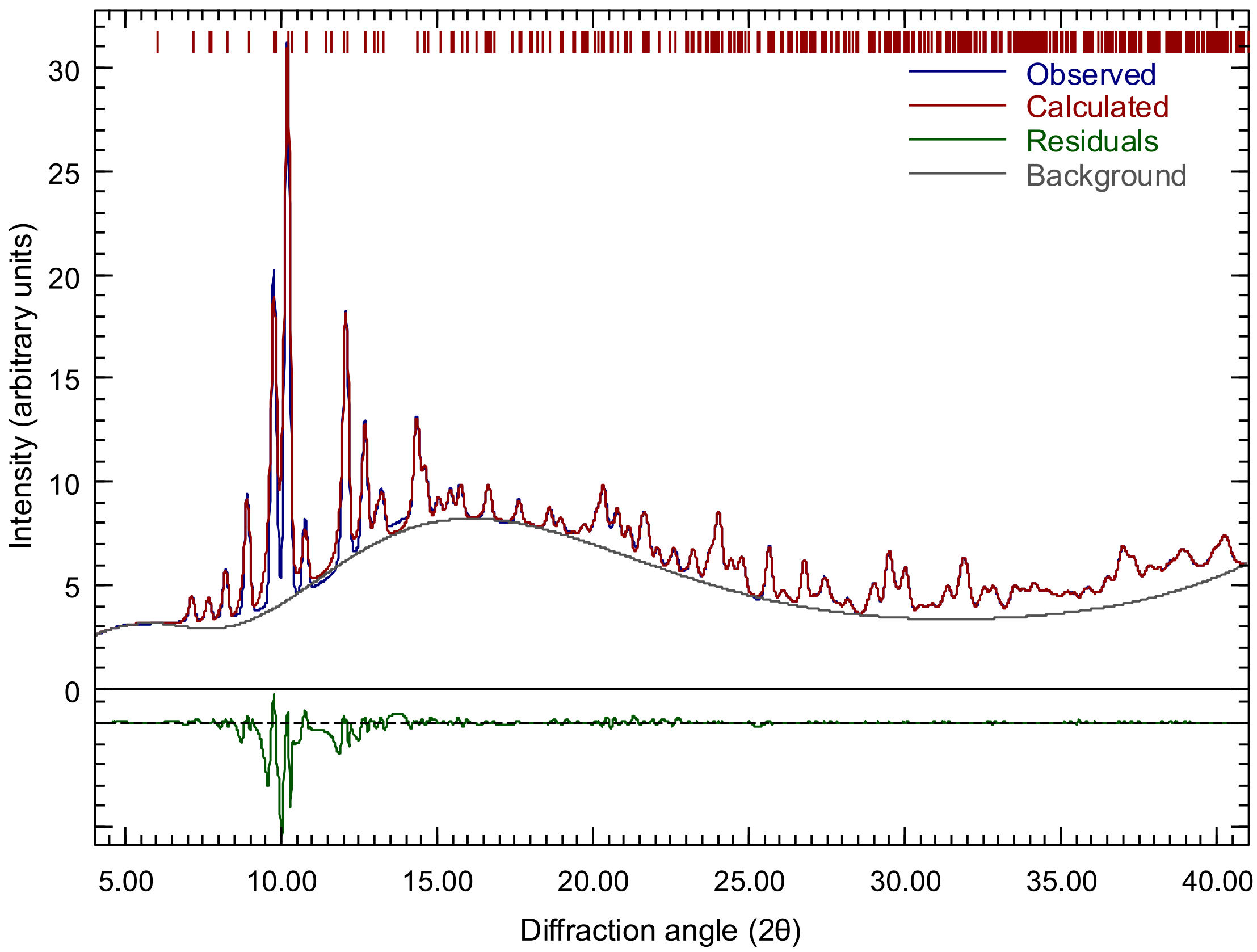

Figure S19. Powder XRD pattern of **3-Y** in the range used for Le Bail profile fitting. Rwp = 1.43%, Rexp = 0.45%, χ^2 = 10.098, GoF = 3.178.

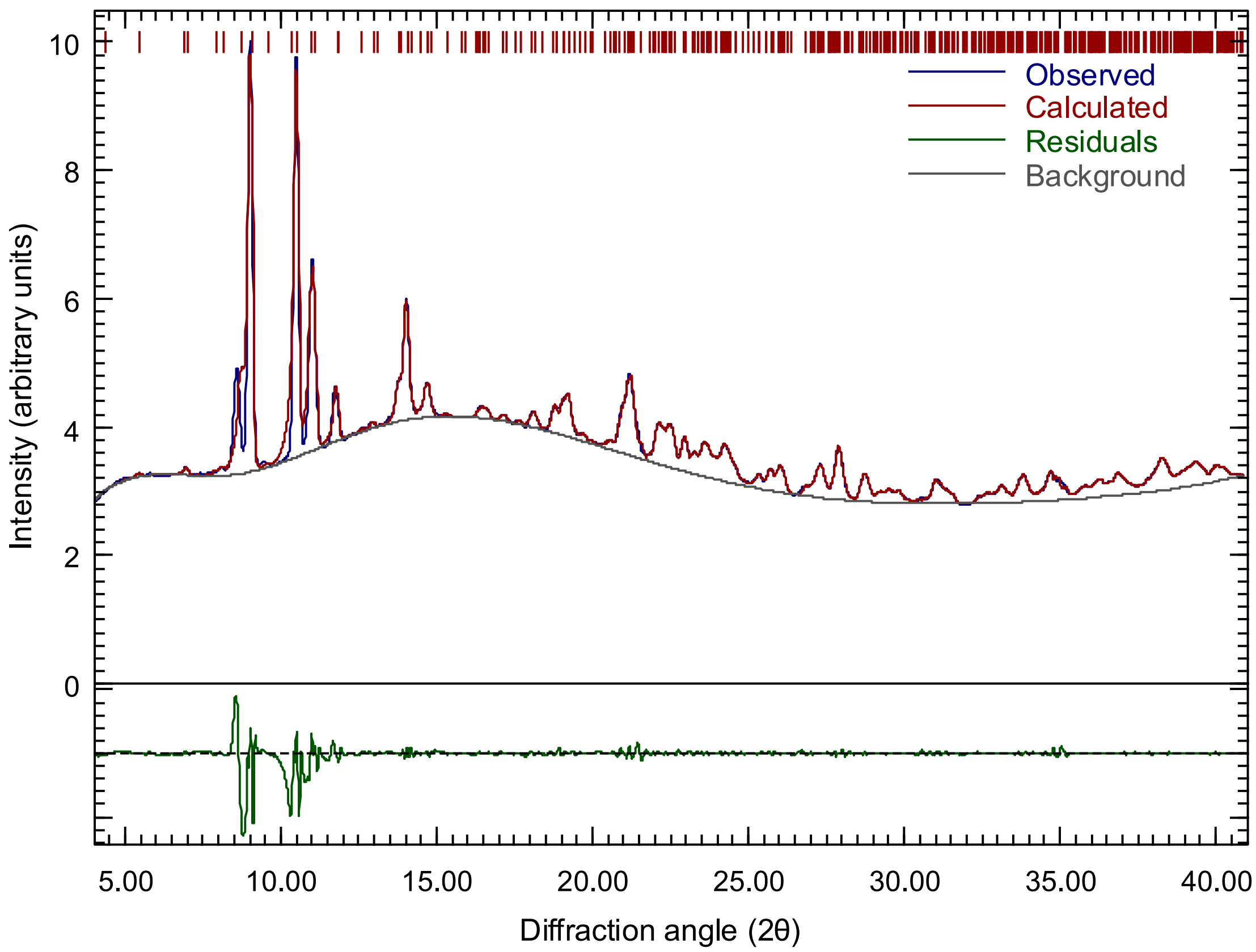

Figure S20. Powder XRD pattern of **4-Dy** in the range used for Le Bail profile fitting. Rwp = 1.32%, Rexp = 0.62%, χ^2 = 4.533, GoF = 2.129.

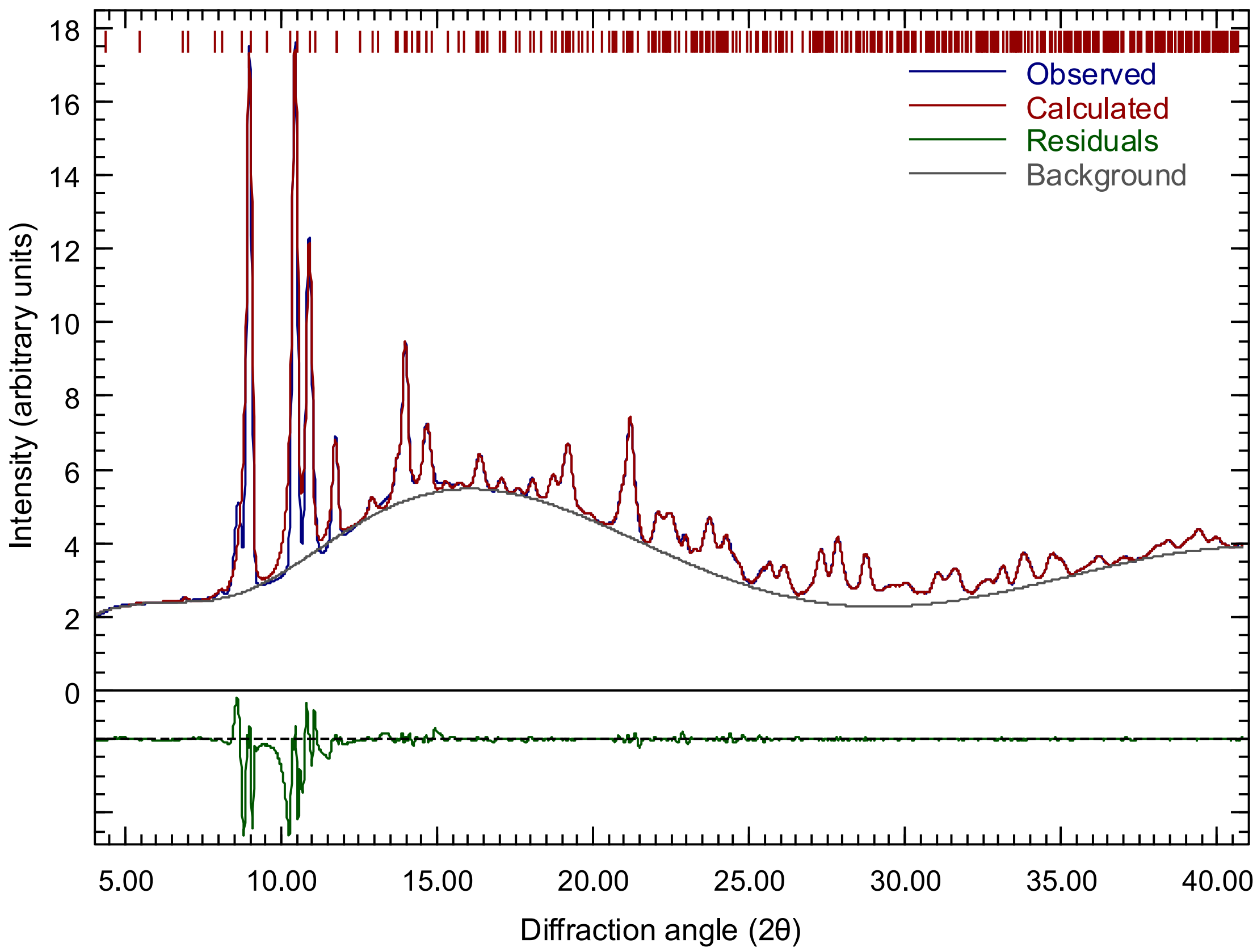

Figure S21. Powder XRD pattern of **4-Y** in the range used for Le Bail profile fitting. Rwp = 1.27%, Rexp = 0.51%, χ^2 = 6.201, GoF = 2.490.

3. IR spectra

3.1 [Ln{N(Si^{i}Pr$_3$)$_2$}(Cp^{ttt})][Al{OC(CF$_3$)$_3$}$_4$] (1-Ln)

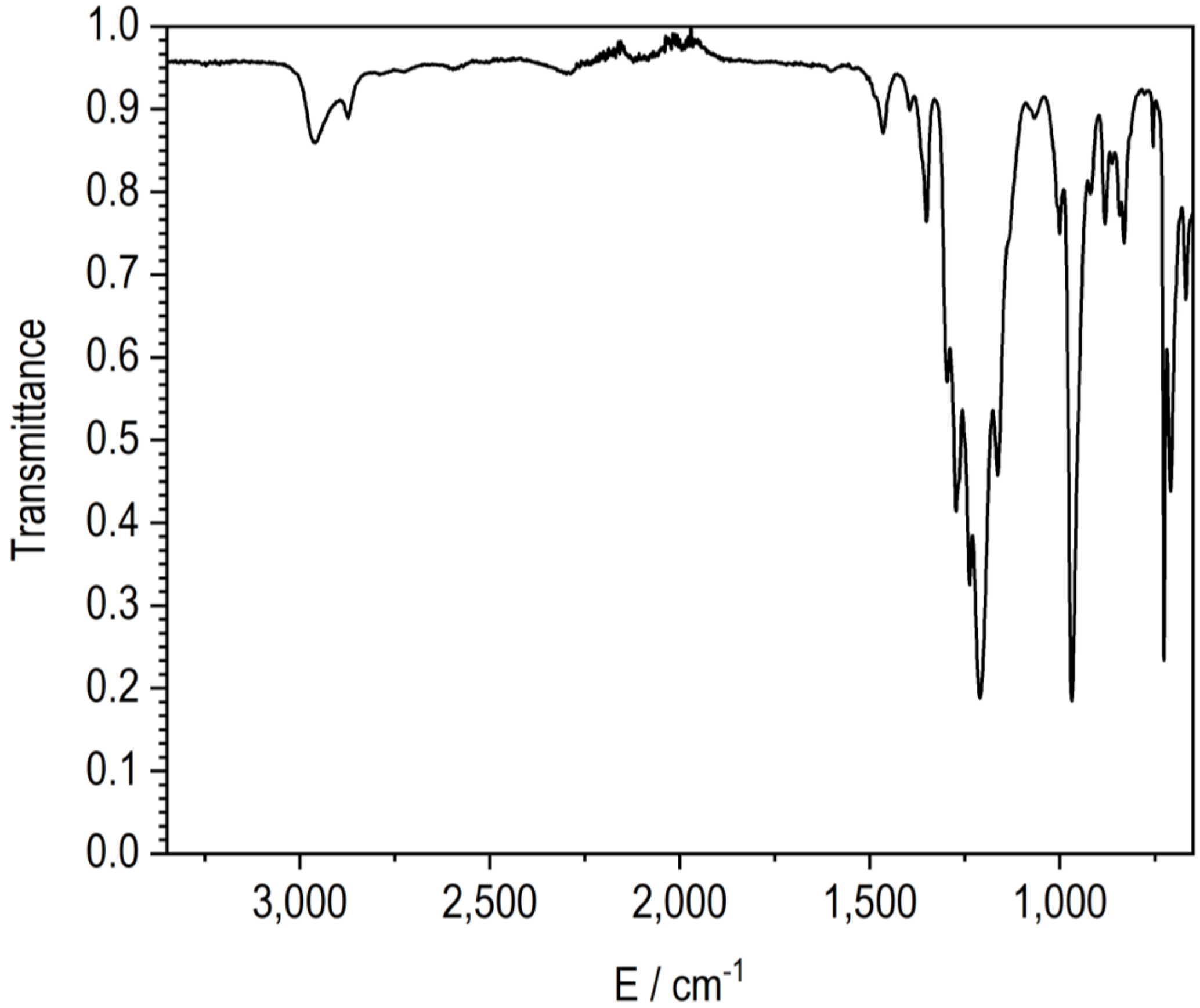

Figure S22. FT-IR (ATR, microcrystalline solid) spectrum of **1-Dy**.

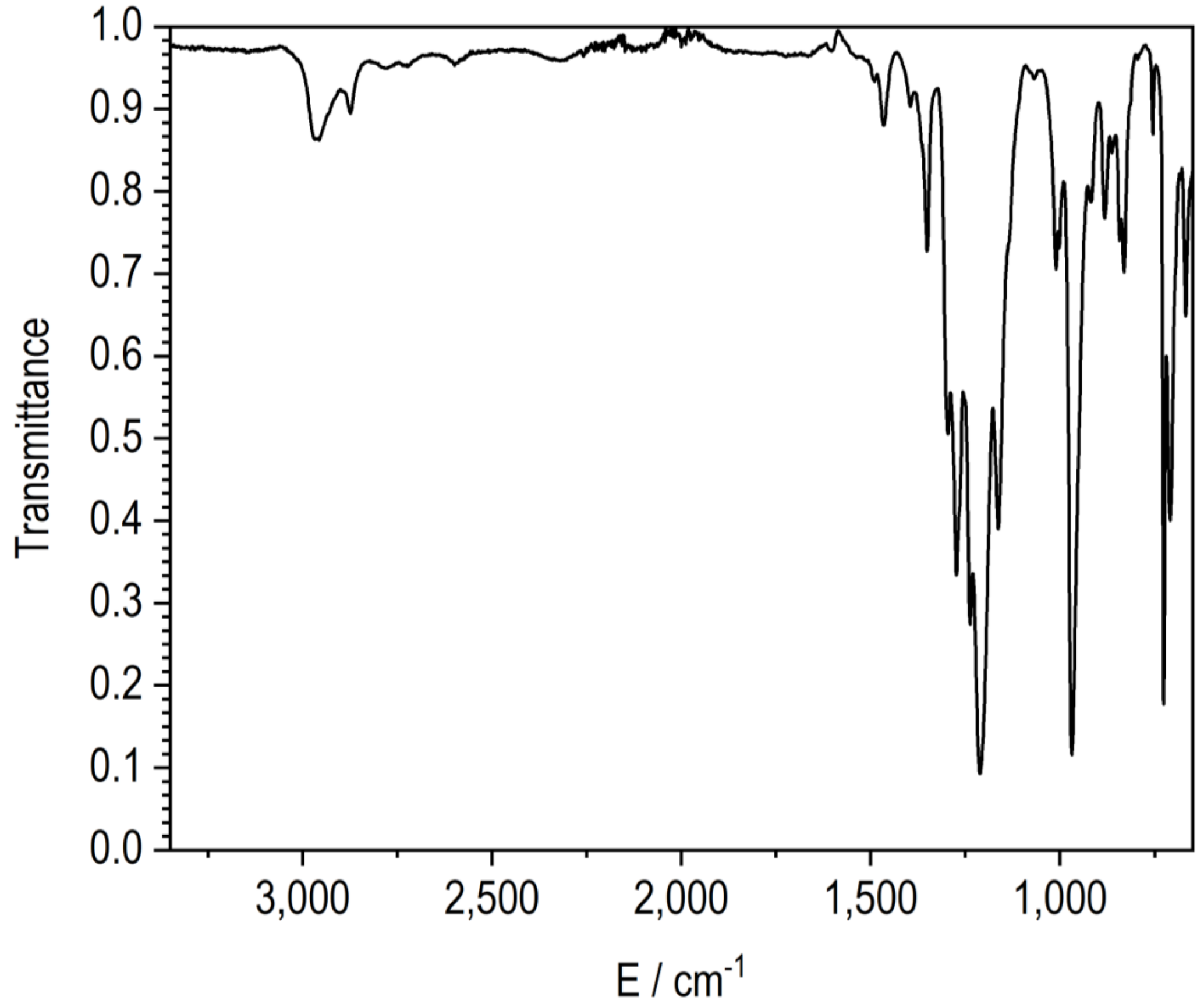

Figure S23. FT-IR (ATR, microcrystalline solid) spectrum of **1-Y**.

a.

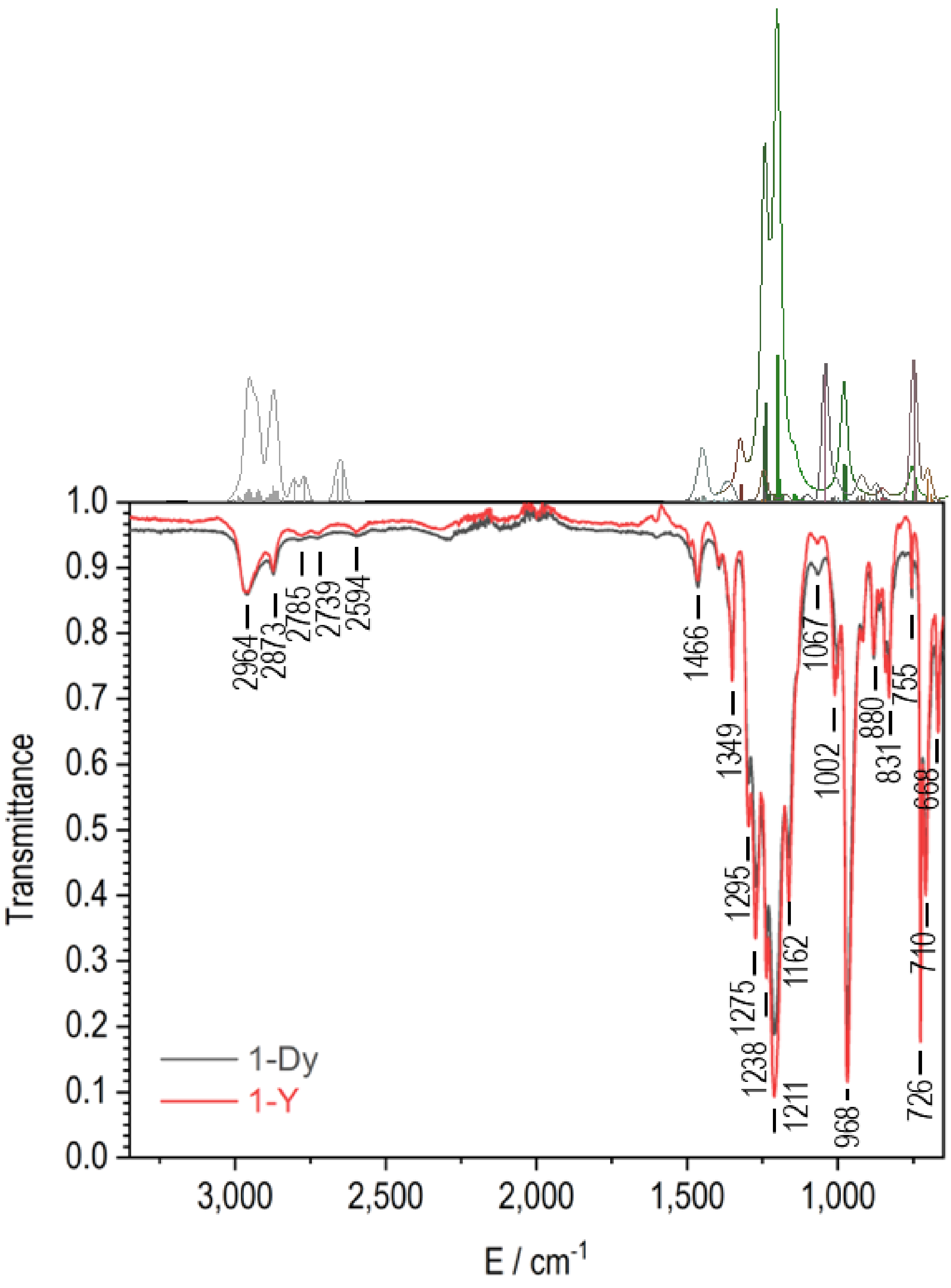

Figure S24. a. Overlay of the FT-IR (ATR, microcrystalline solid) spectrum of **1-Dy, 1-Y,** and (above) the superimposed DFT-calculated vibrational modes of the independently calculated cation and anion of **1-Y**; empirical energy scale factor (0.97 for the anion, 0.94 for the cations) and Lorentzian convolution (fwhm = 30 cm^{-1}) applied, coloured as a weighted average of the atoms involved in the vibration (C = black, N = blue, O = red, F = green, Al = magenta, Si = orange, Y = teal). **b.** (Overleaf) Selected DFT-calculated vibrational modes of the cation and anion of **1-Y**; atomic displacement vectors shown with red arrows scaled by the magnitude of the displacement.

b.

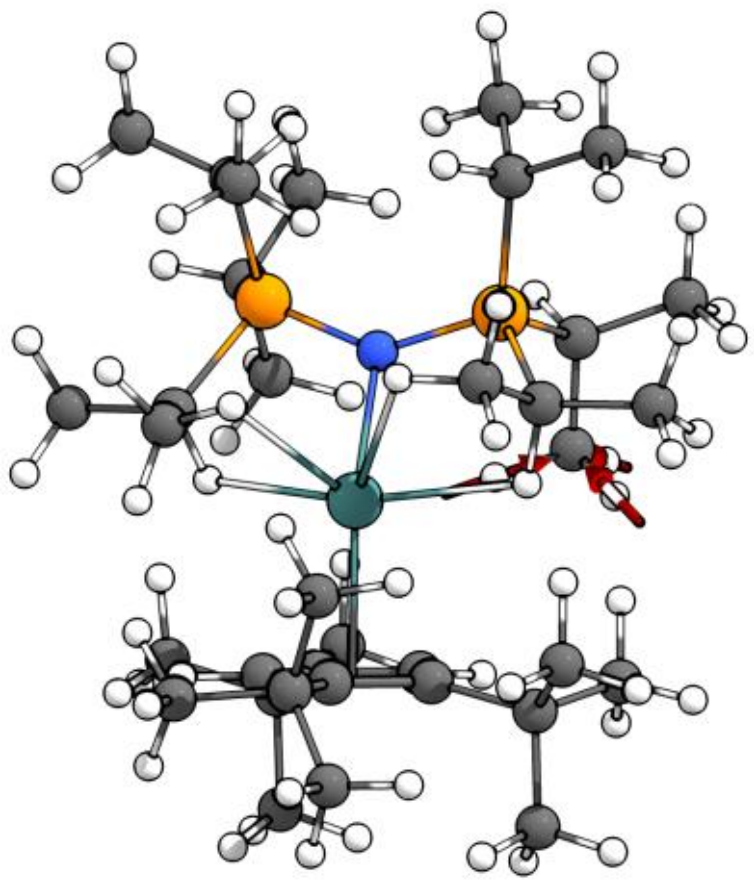

**mode 268**	**E / cm^{-1}**
DFT (raw)	3026.68
DFT (scaled)	2845.08
observed band	2873

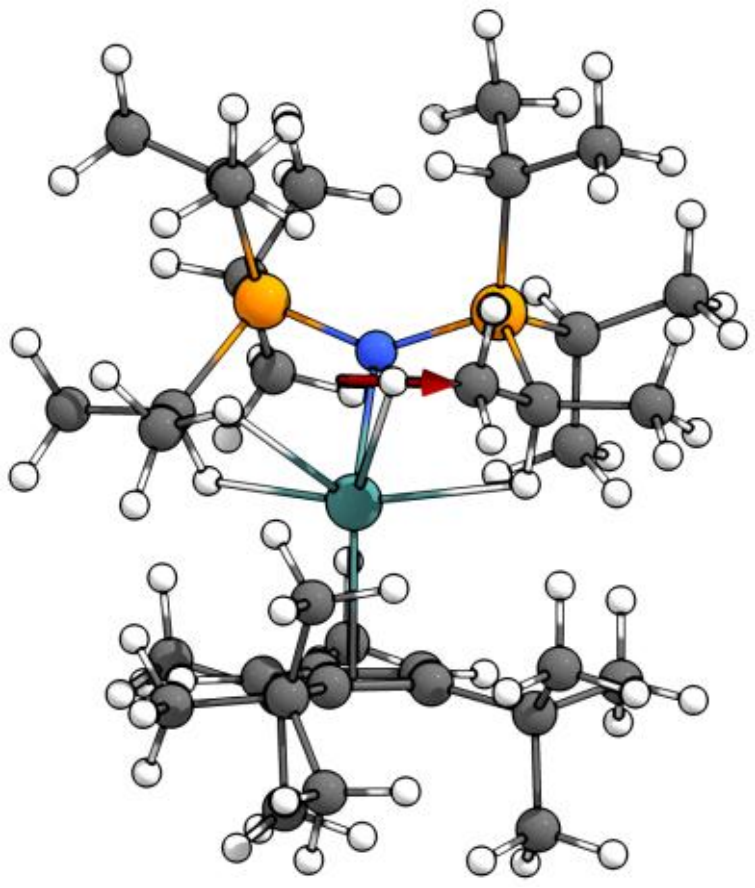

**mode 263**	**E / cm^{-1}**
DFT (raw)	2965.61
DFT (scaled)	2787.67
observed band	2785

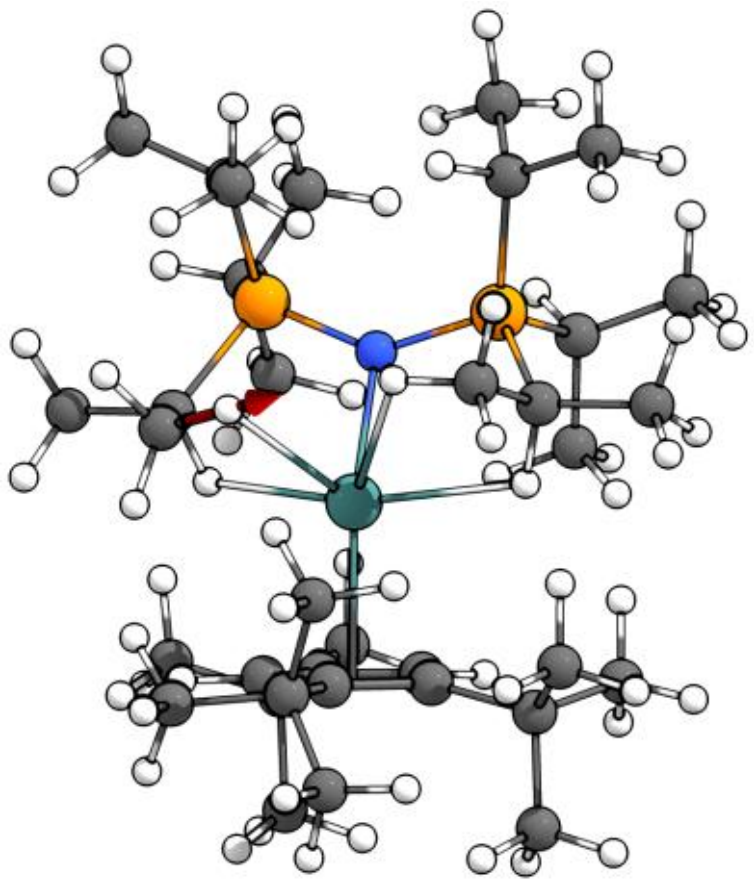

**mode 262**	**E / cm^{-1}**
DFT (raw)	2928.43
DFT (scaled)	2752.72
observed band	2739

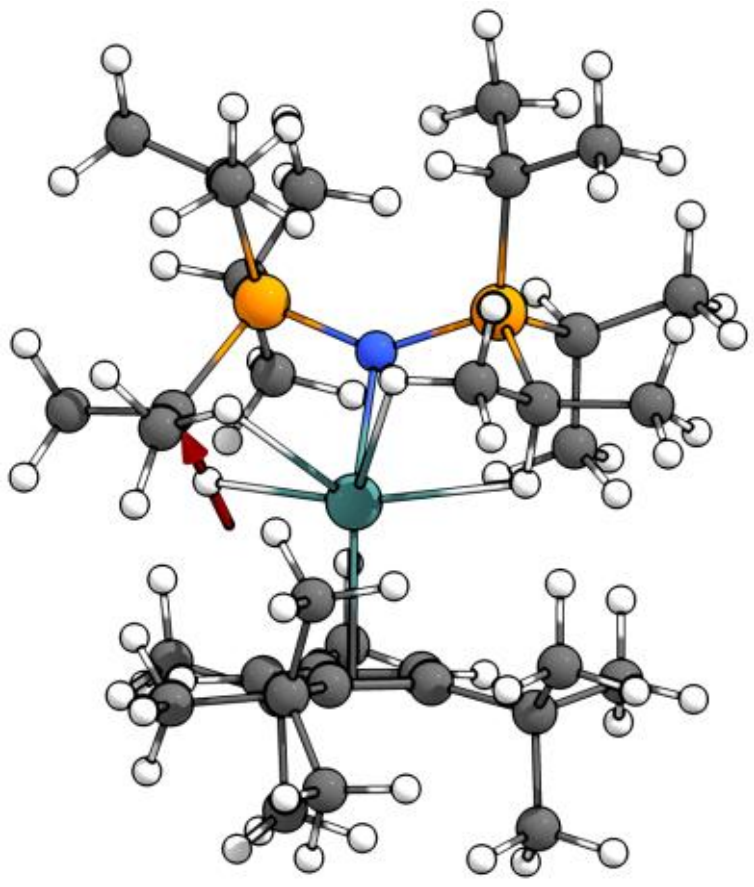

**mode 261**	**E / cm^{-1}**
DFT (raw)	2811.34
DFT (scaled)	2642.66
observed band	2594

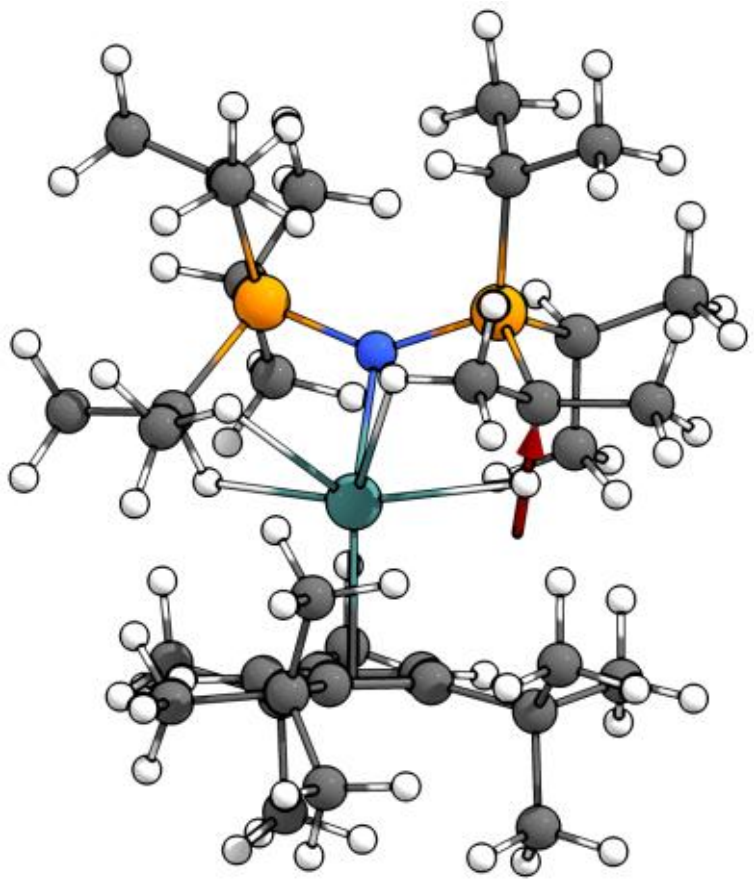

**mode 260**	**E / cm^{-1}**
DFT (raw)	2792.14
DFT (scaled)	2624.61
observed band	2594

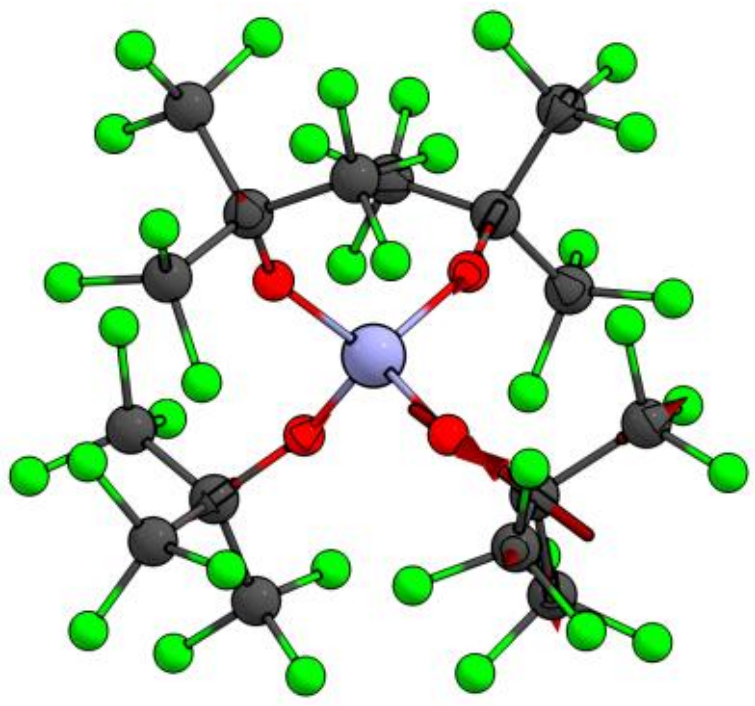

**mode 170**	**E / cm^{-1}**
DFT (raw)	1388.18
DFT (scaled)	1346.54
observed band	1349

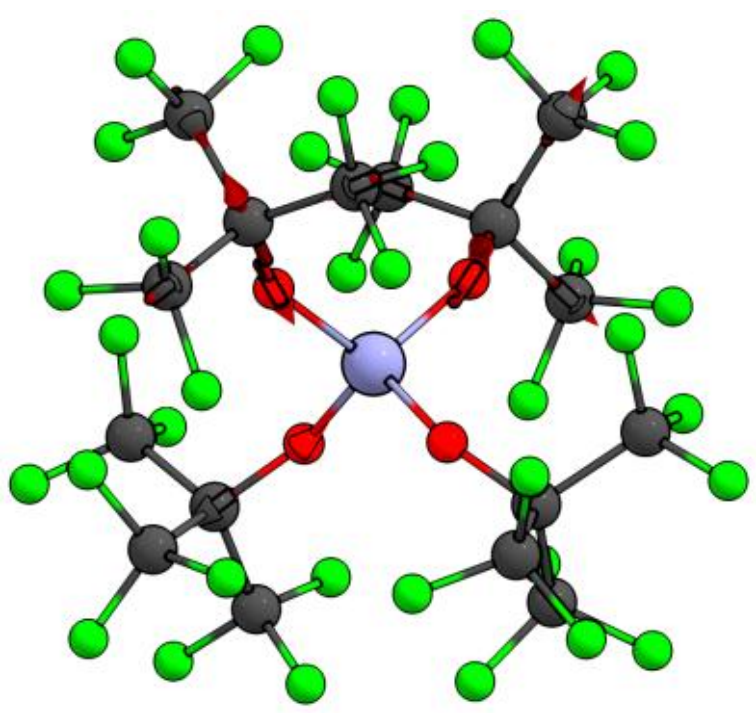

**mode 169**	**E / cm^{-1}**
DFT (raw)	1386.95
DFT (scaled)	1345.34
observed band	1349

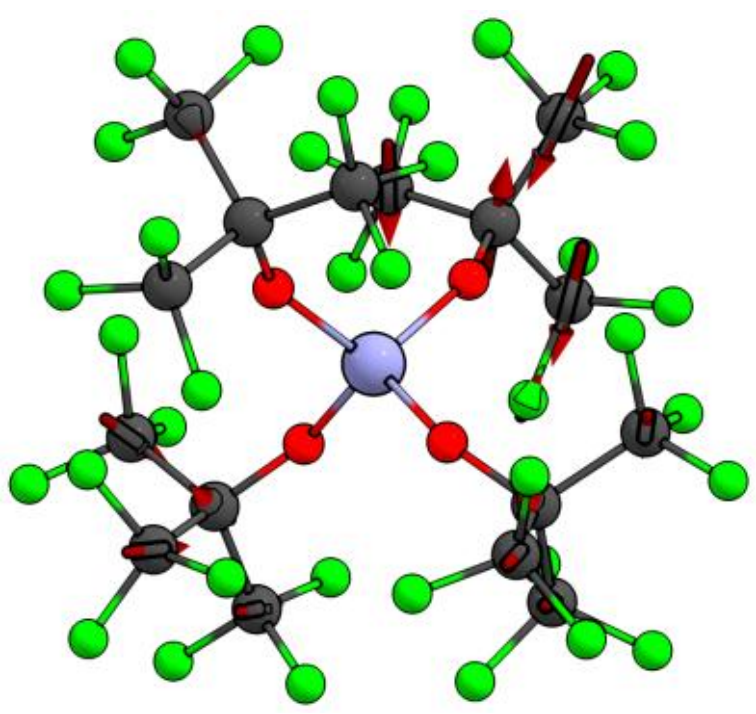

**mode 167**	**E / cm^{-1}**
DFT (raw)	1295.32
DFT (scaled)	1256.46
observed band	1275

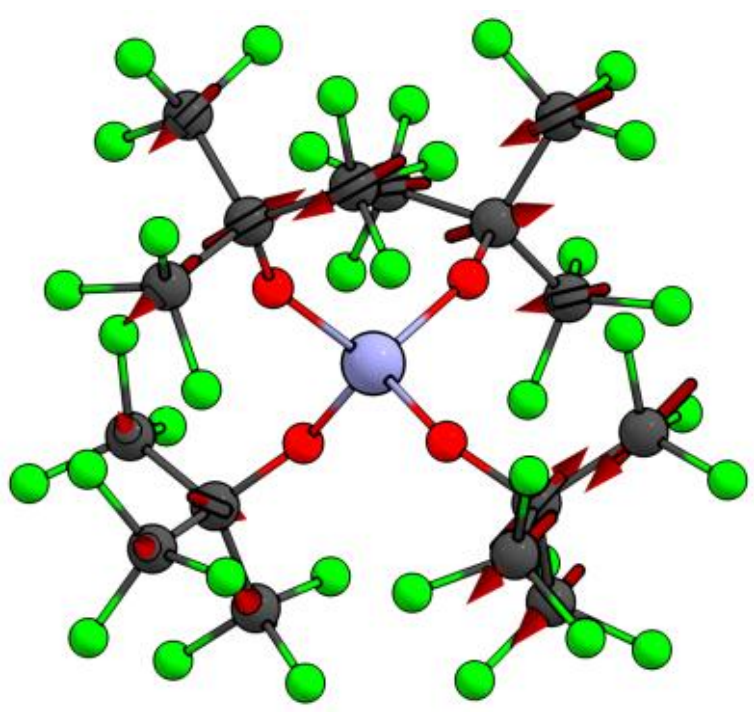

**mode 164**	**E / cm^{-1}**
DFT (raw)	1291.42
DFT (scaled)	1252.68
observed band	1275

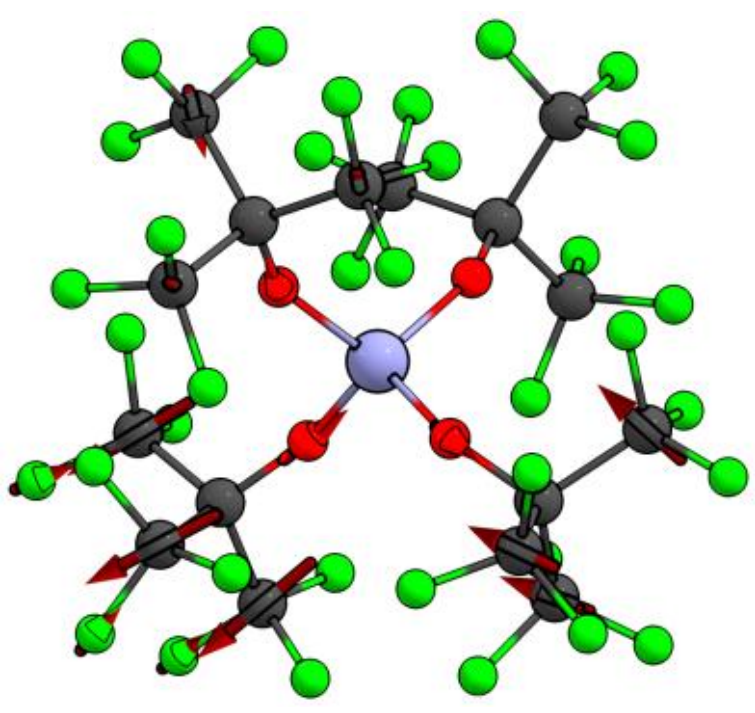

**mode 153**	**E / cm^{-1}**
DFT (raw)	1247.50
DFT (scaled)	1210.08
observed band	1211

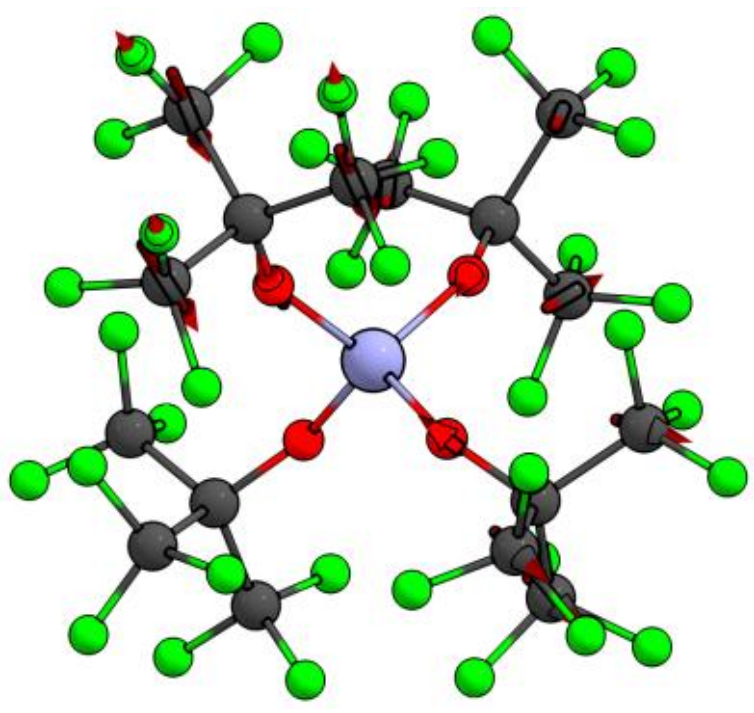

**mode 152**	**E / cm^{-1}**
DFT (raw)	1247.23
DFT (scaled)	1209.81
observed band	1211

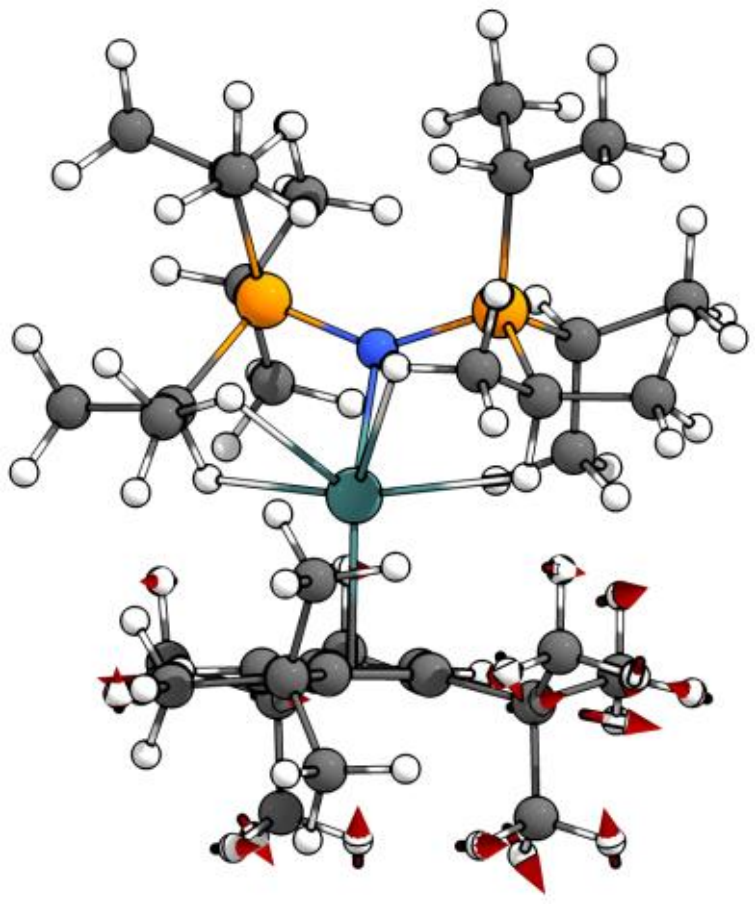

**mode 184**	**E / cm^{-1}**
DFT (raw)	1271.80
DFT (scaled)	1195.49
observed band	*obscured*

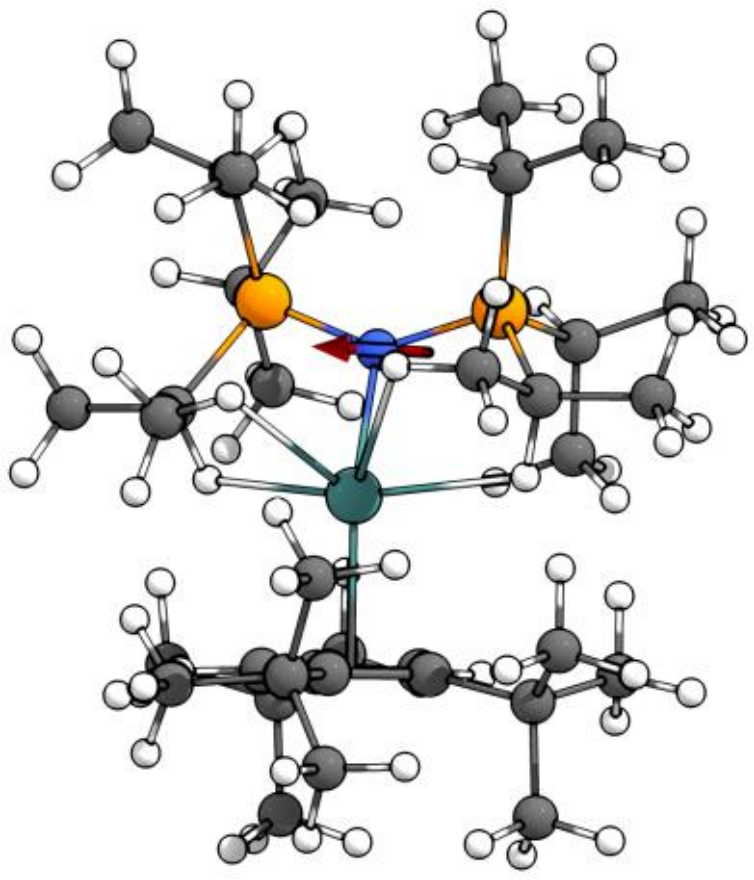

**mode 155**	**E / cm^{-1}**
DFT (raw)	1047.30
DFT (scaled)	984.46
observed band	1002

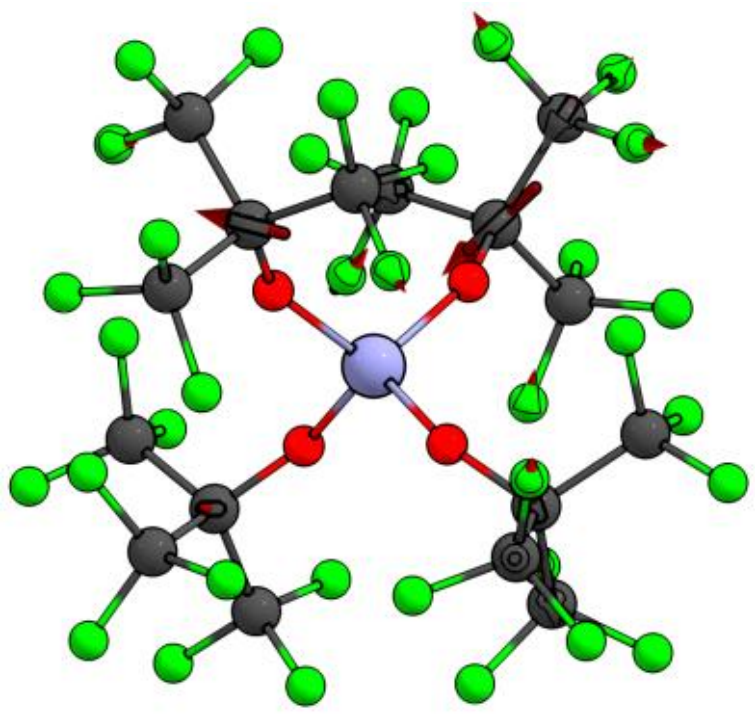

**mode 131**	**E / cm^{-1}**
DFT (raw)	994.47
DFT (scaled)	964.64
observed band	968

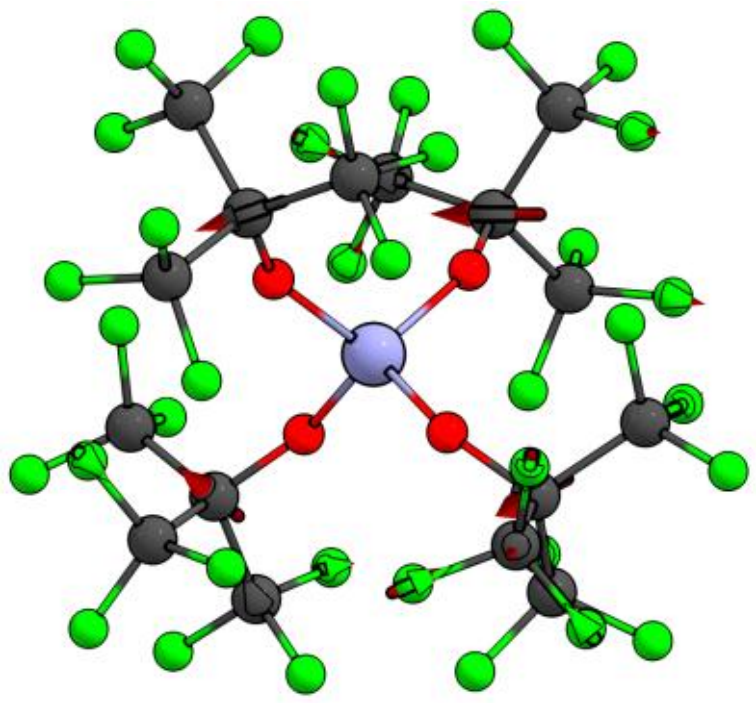

**mode 130**	**E / cm^{-1}**
DFT (raw)	994.04
DFT (scaled)	964.22
observed band	968

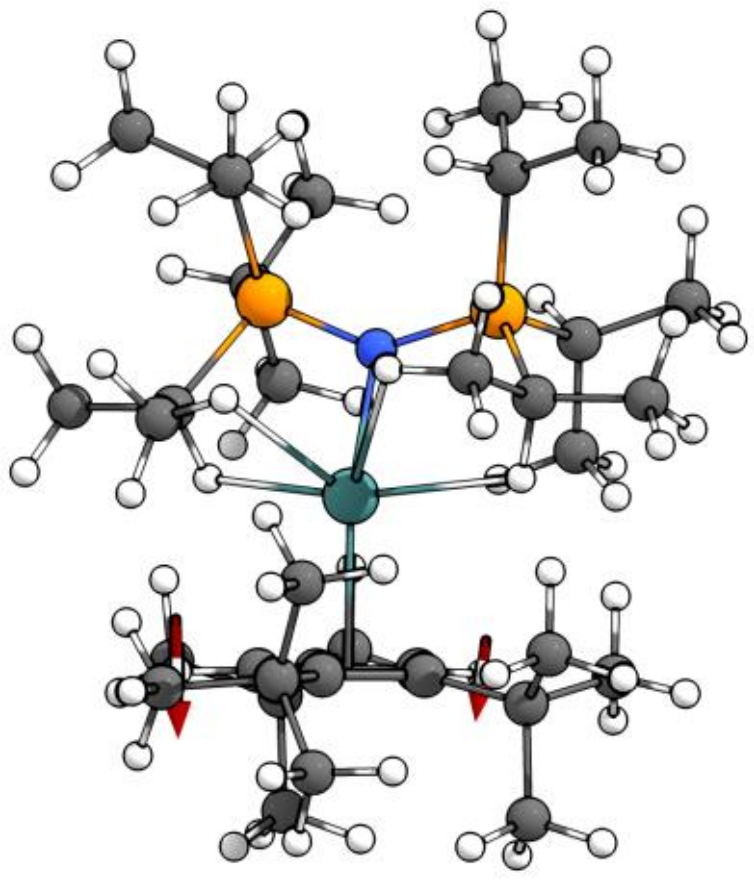

**mode 114**	**E / cm^{-1}**
DFT (raw)	862.93
DFT (scaled)	811.15
observed band	831

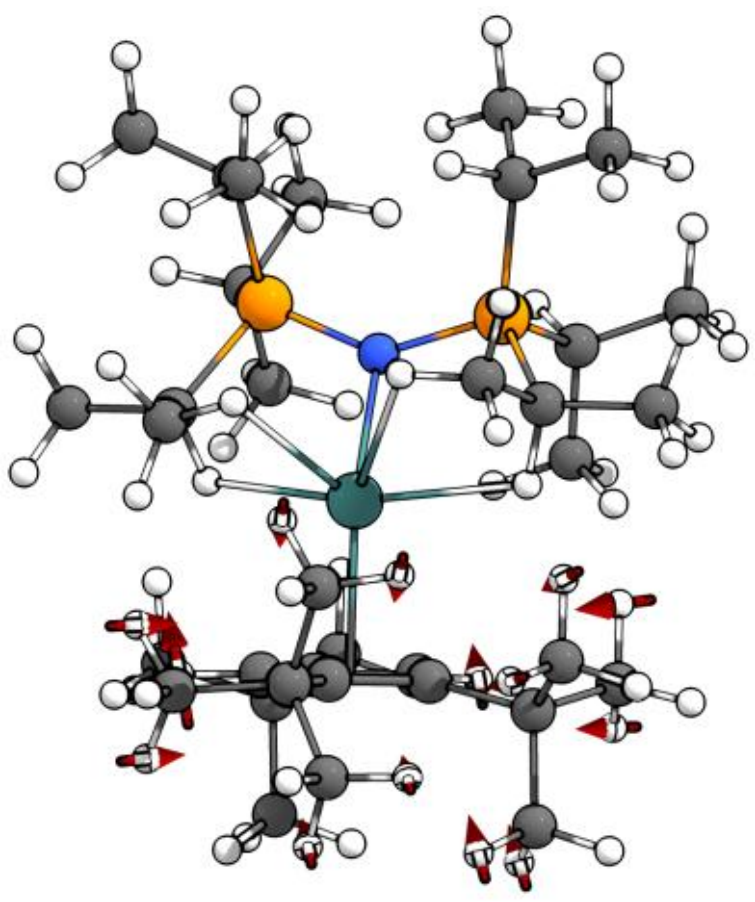

**mode 113**	**E / cm^{-1}**
DFT (raw)	856.45
DFT (scaled)	805.06
observed band	831

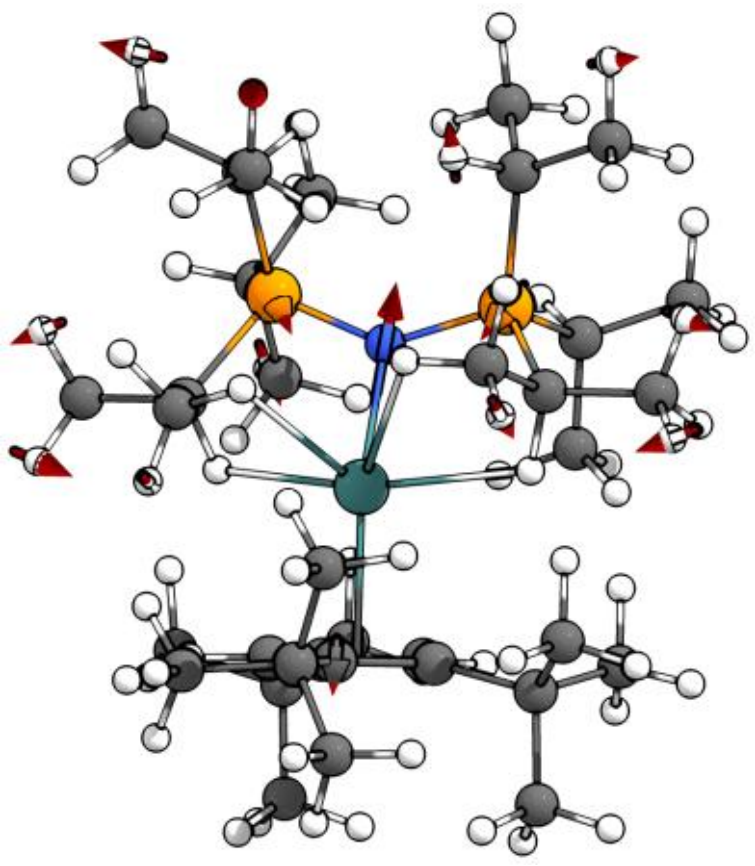

**mode 109**	**E / cm^{-1}**
DFT (raw)	728.77
DFT (scaled)	685.04
observed band	668

3.2 [Ln{N(Si^{i}Pr$_3$)$_2$}(Cp′′′)][Al{OC(CF$_3$)$_3$}$_4$] (2-Ln)

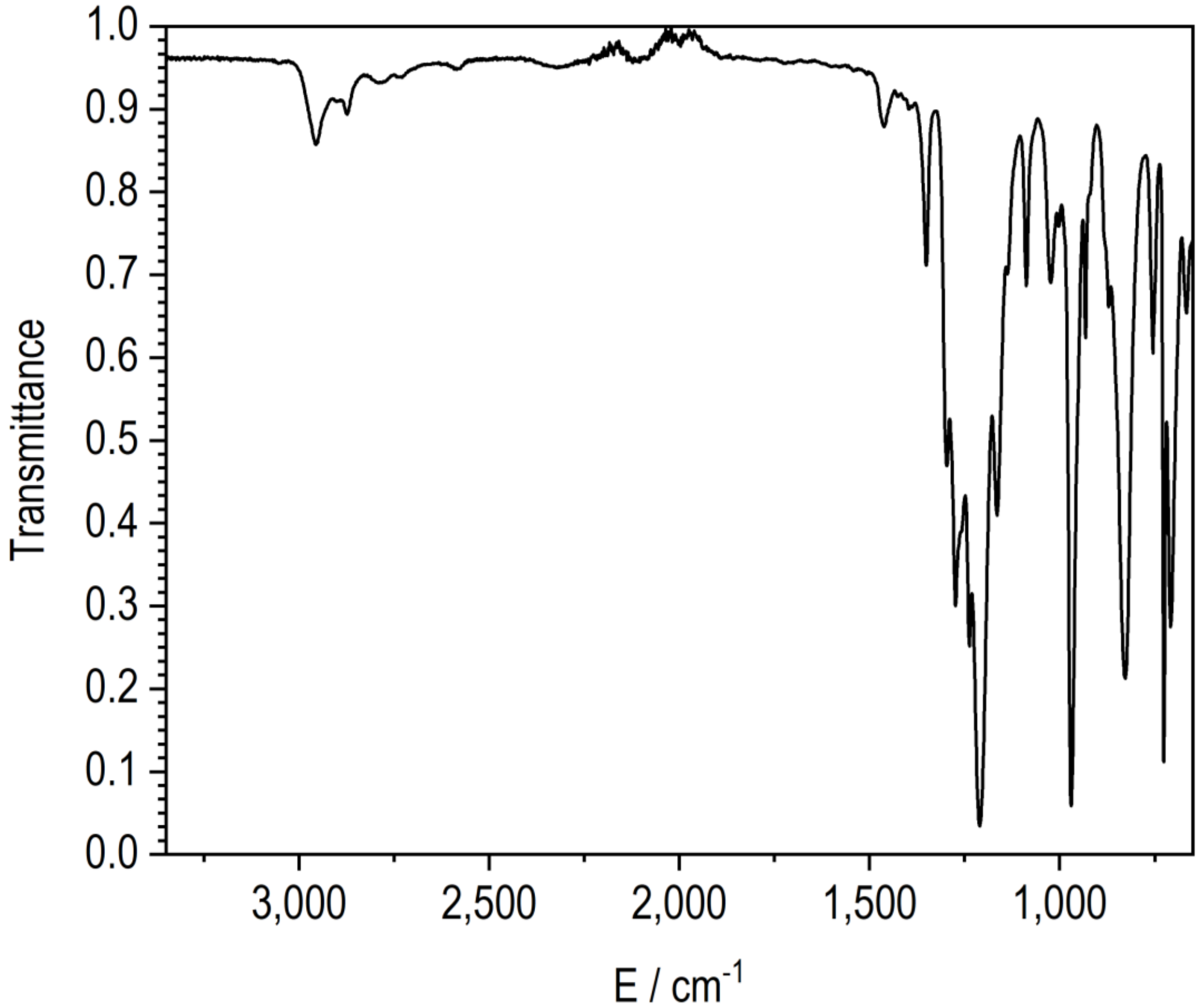

Figure S25. FT-IR (ATR, microcrystalline solid) spectrum of **2-Dy**.

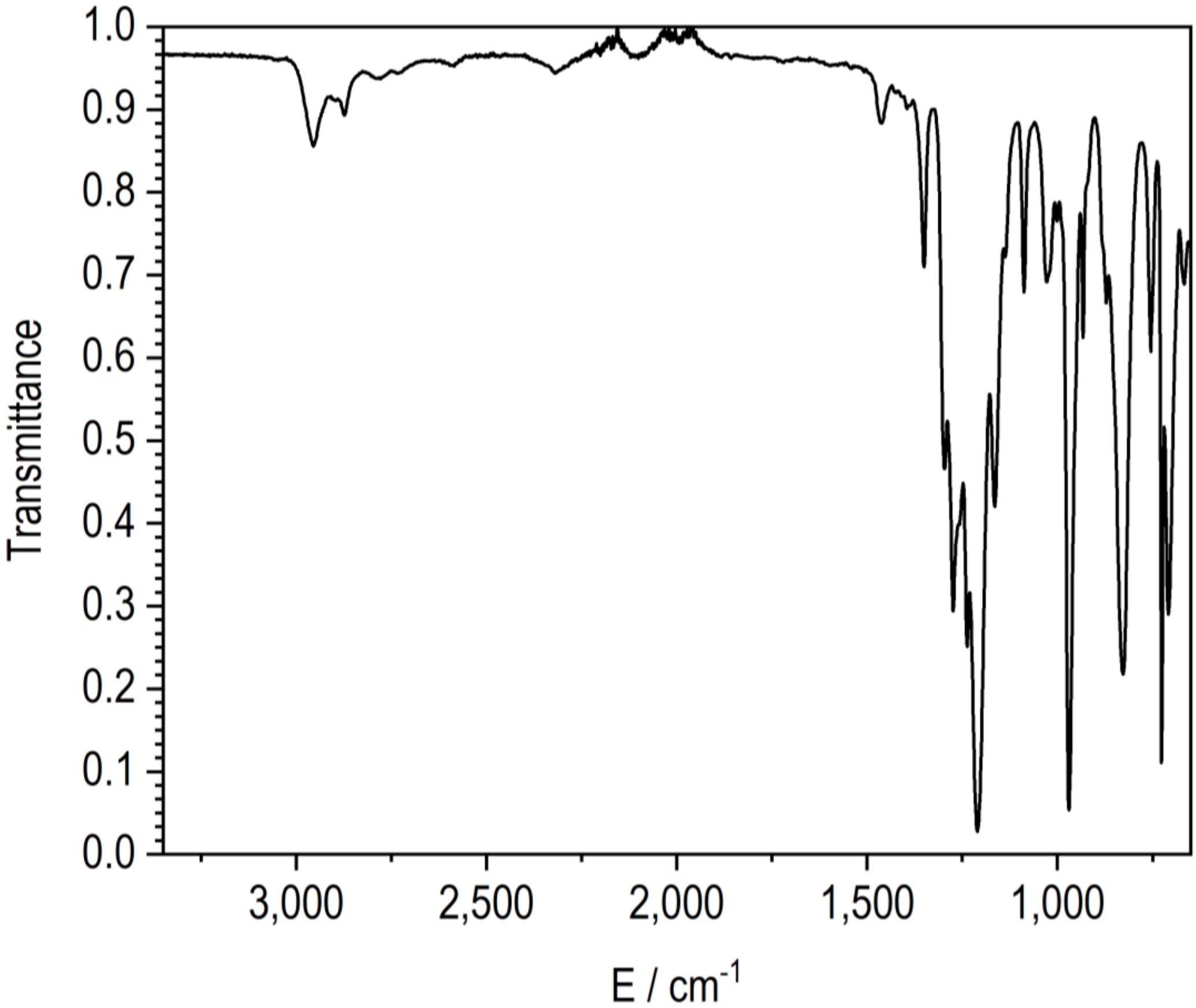

Figure S26. FT-IR (ATR, microcrystalline solid) spectrum of **2-Y**.

a.

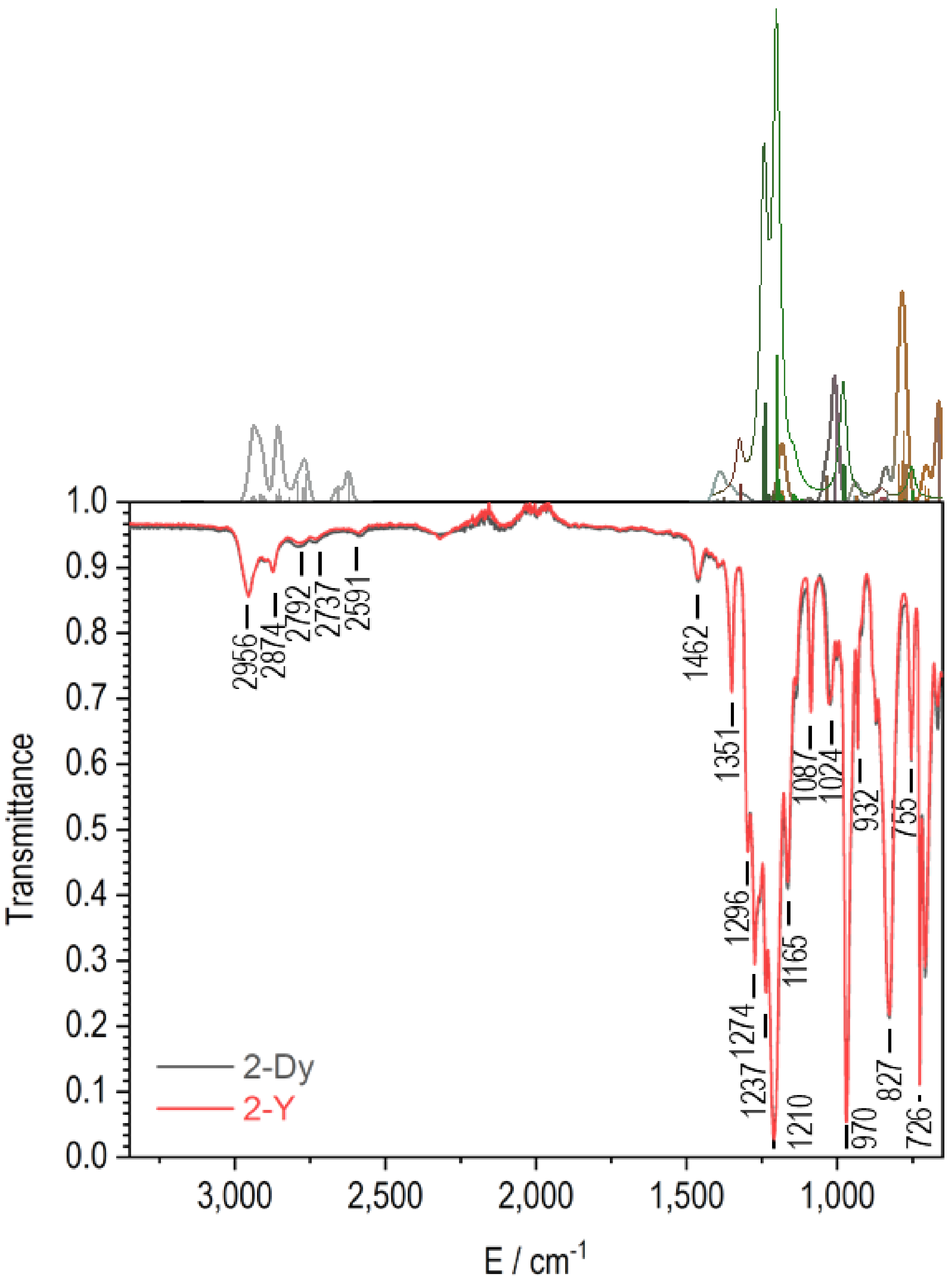

Figure S27. a. Overlay of the FT-IR (ATR, microcrystalline solid) spectrum of **2-Dy, 2-Y,** and (above) the superimposed DFT-calculated vibrational modes of the independently calculated cation and anion of **2-Y**; empirical energy scale factor (0.97 for the anion, 0.94 for the cation) and Lorentzian convolution (fwhm = 30 cm^{-1}) applied, coloured as a weighted average of the atoms involved in the vibration (C = black, N = blue, O = red, F = green, Al = magenta, Si = orange, Y = teal). **b.** (Overleaf) Selected DFT-calculated vibrational modes of the cation and anion of **2-Y**; atomic displacement vectors shown with red arrows scaled by the magnitude of the displacement.

b.

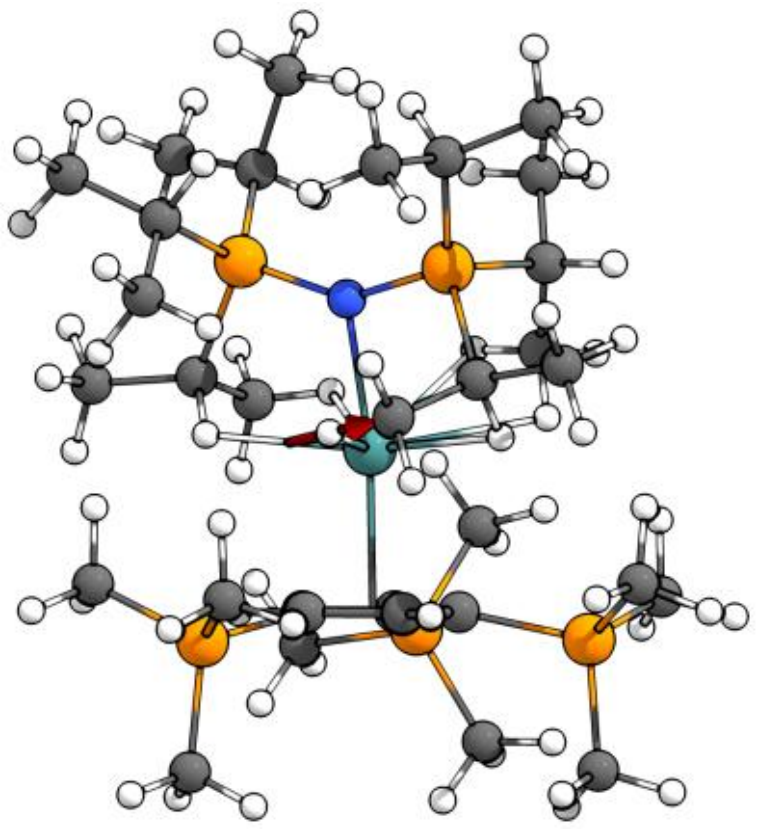

**mode 264**	**E / cm^{-1}**
DFT (raw)	2972.72
DFT (scaled)	2794.36
observed band	2792

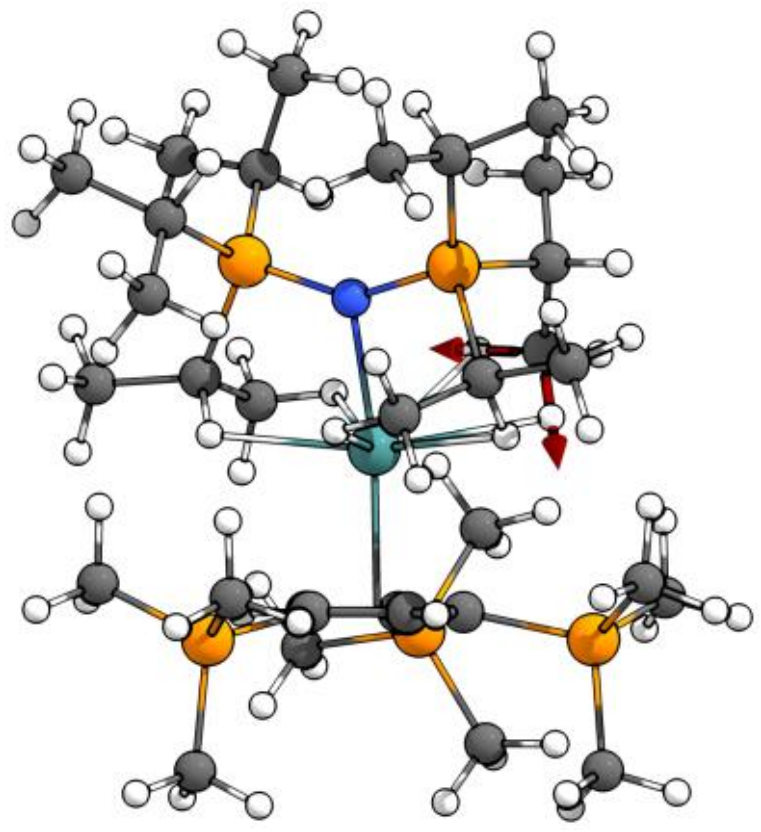

**mode 263**	**E / cm^{-1}**
DFT (raw)	2953.13
DFT (scaled)	2775.94
observed band	2737

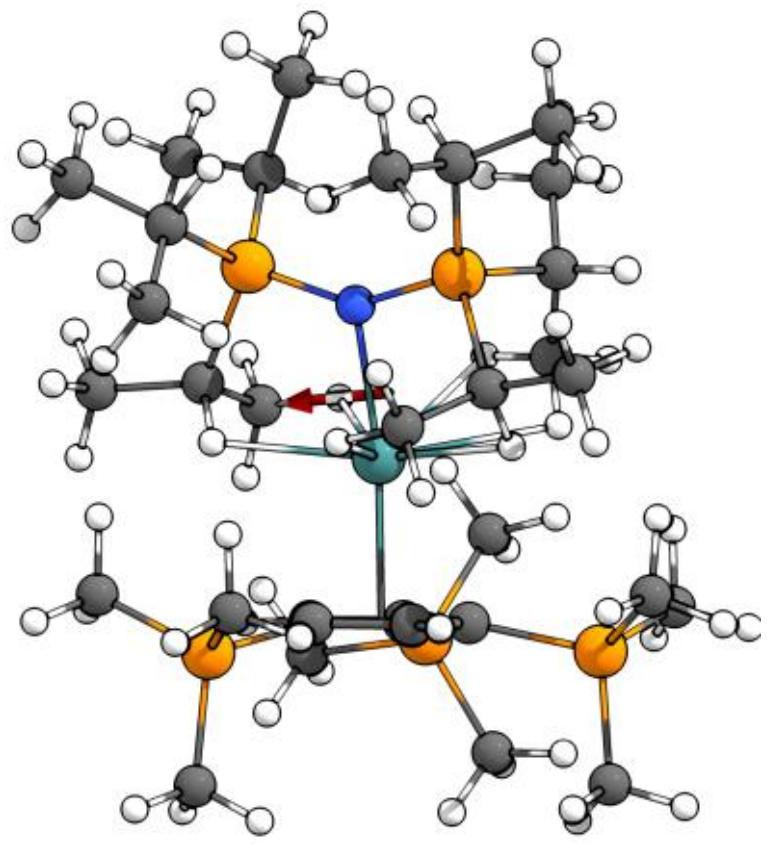

**mode 262**	**E / cm^{-1}**
DFT (raw)	2942.13
DFT (scaled)	2765.60
observed band	2792

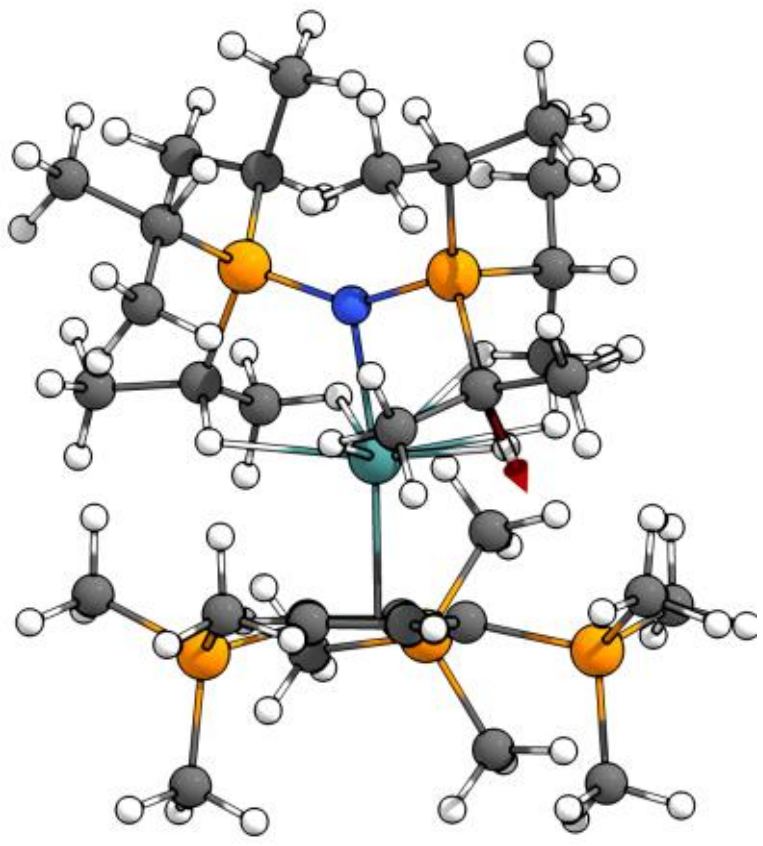

**mode 261**	**E / cm^{-1}**
DFT (raw)	2831.35
DFT (scaled)	2661.47
observed band	2591

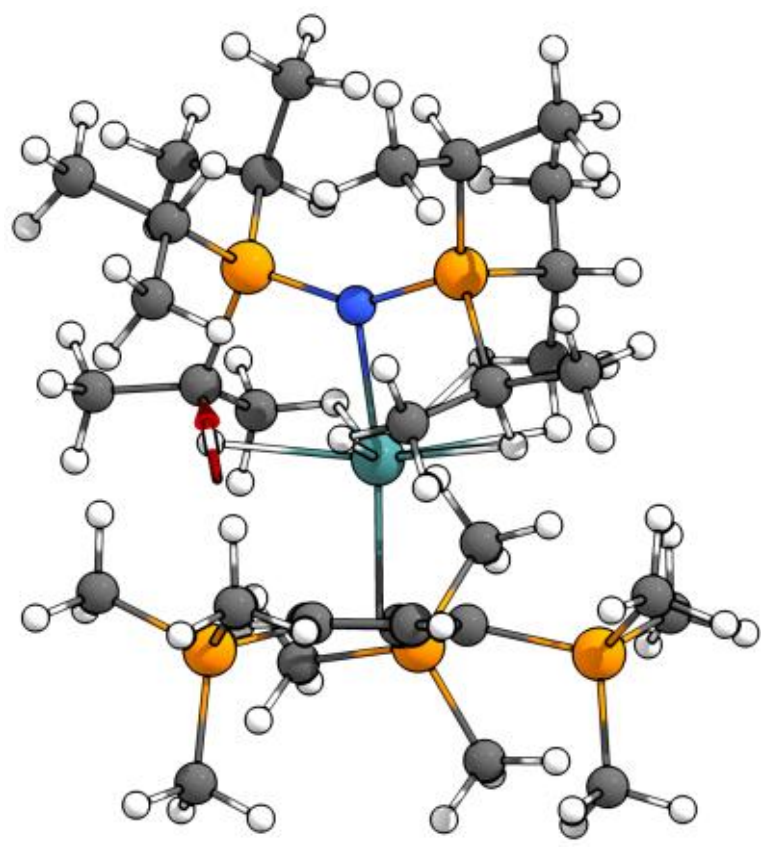

**mode 260**	**E / cm^{-1}**
DFT (raw)	2793.71
DFT (scaled)	2626.09
observed band	2591

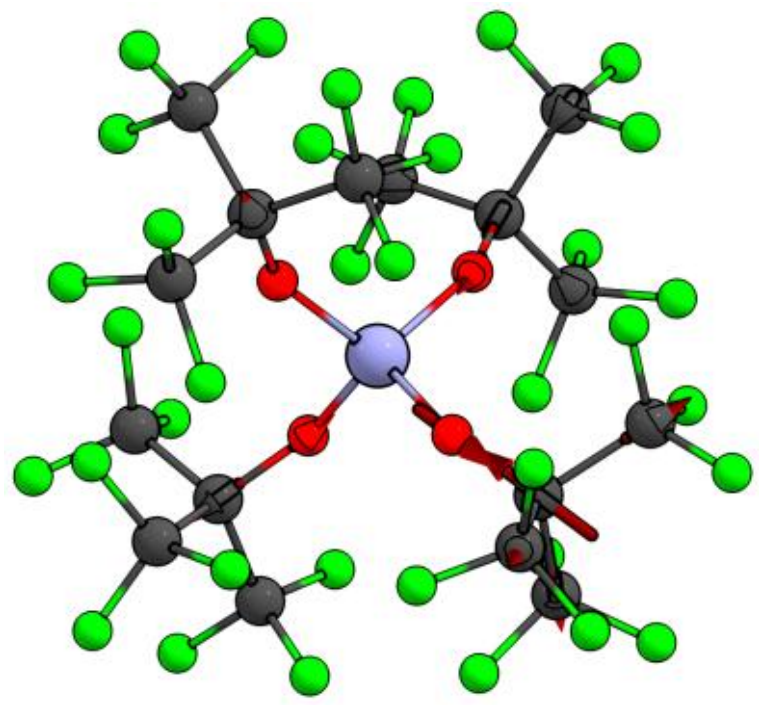

**mode 170**	**E / cm^{-1}**
DFT (raw)	1388.18
DFT (scaled)	1346.54
observed band	1351

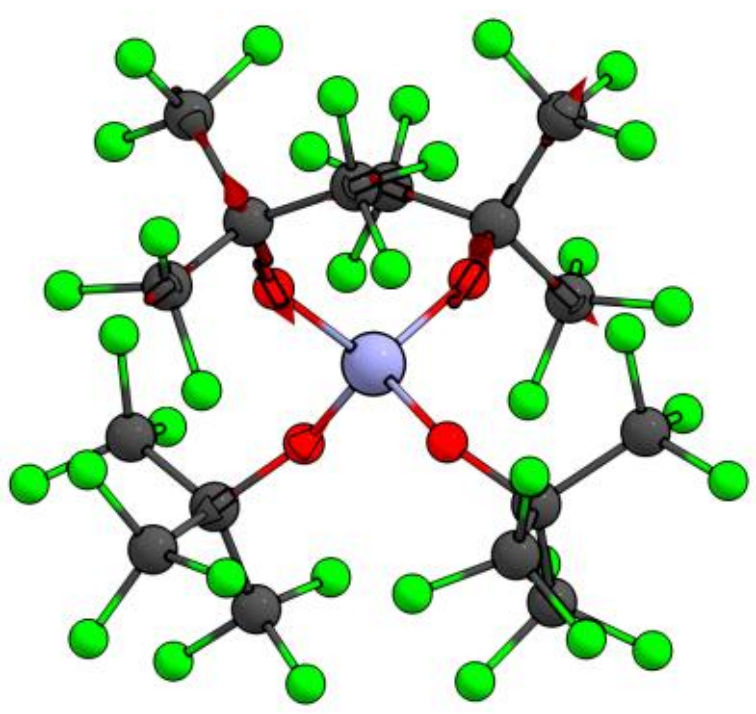

**mode 169**	**E / cm^{-1}**
DFT (raw)	1386.95
DFT (scaled)	1345.34
observed band	1351

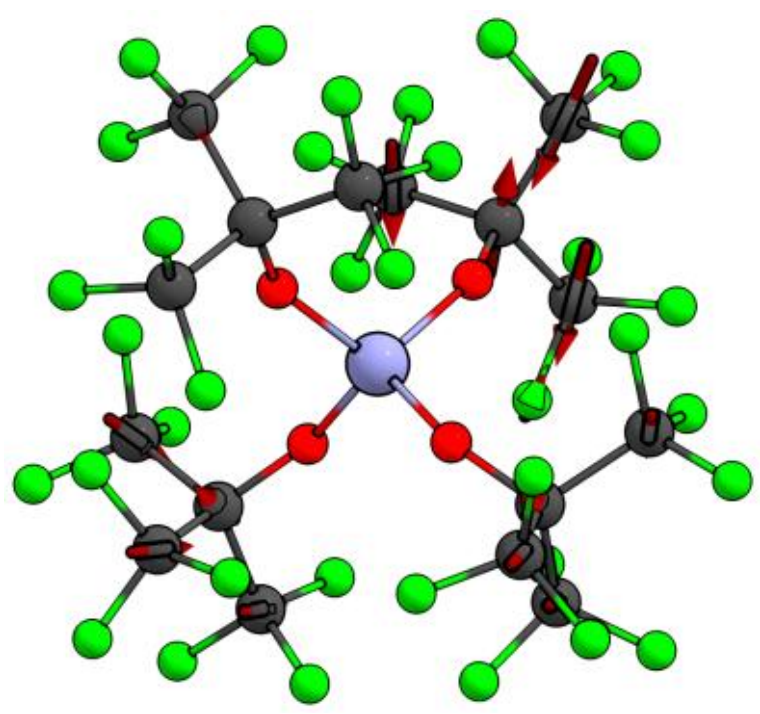

**mode 167**	**E / cm^{-1}**
DFT (raw)	1295.32
DFT (scaled)	1256.46
observed band	1274

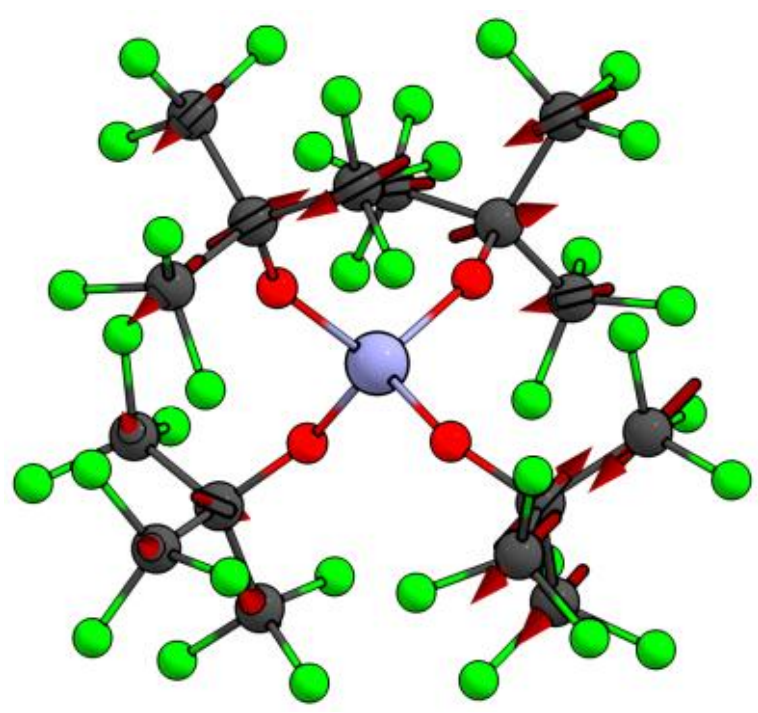

**mode 164**	**E / cm^{-1}**
DFT (raw)	1291.42
DFT (scaled)	1252.68
observed band	1274

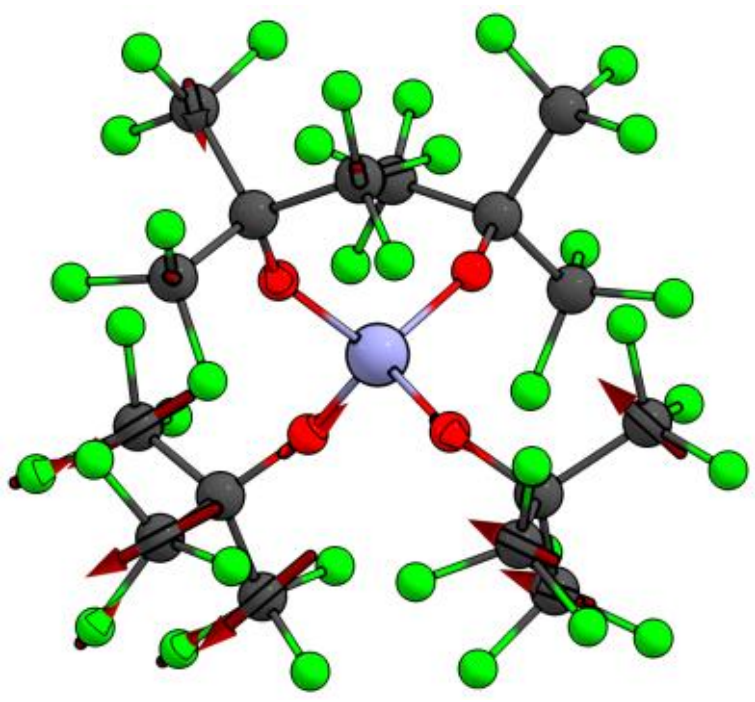

mode 153	E / cm^{-1}
DFT (raw)	1247.50
DFT (scaled)	1210.08
observed band	1210

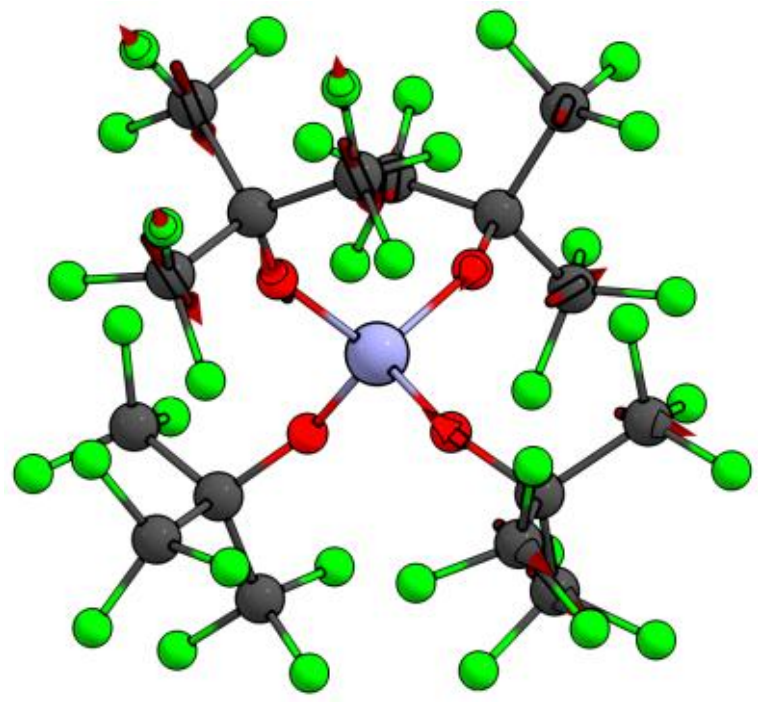

mode 152	E / cm^{-1}
DFT (raw)	1247.23
DFT (scaled)	1209.81
observed band	1210

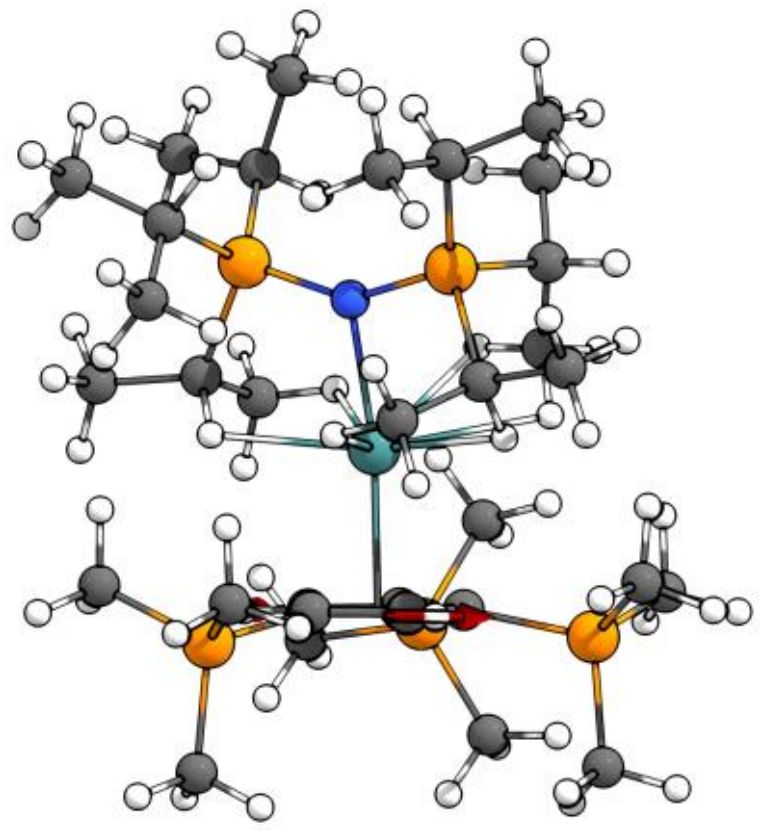

mode 170	E / cm^{-1}
DFT (raw)	1113.79
DFT (scaled)	1046.96
observed band	1087

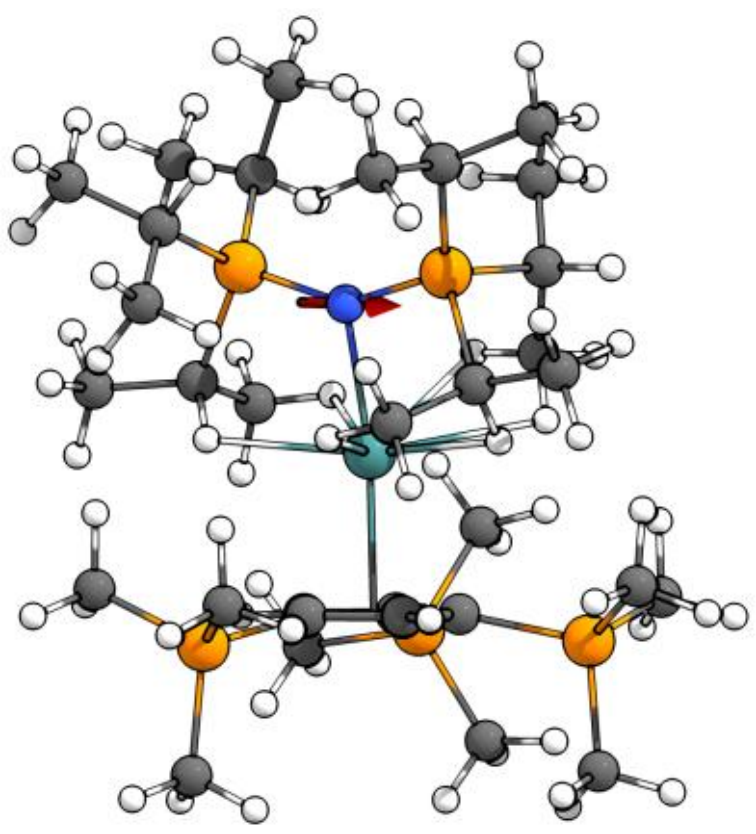

mode 165	E / cm^{-1}
DFT (raw)	1083.67
DFT (scaled)	1018.65
observed band	1024

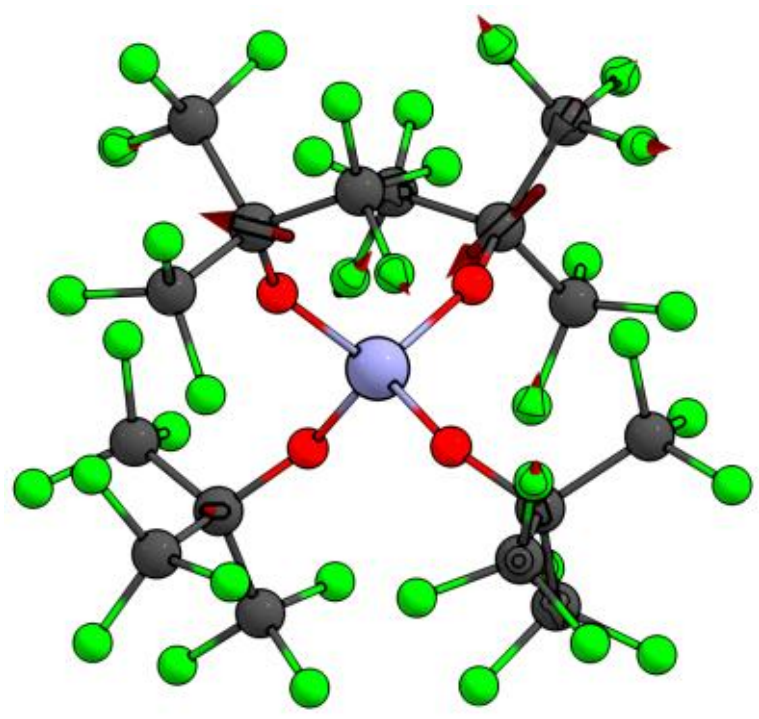

mode 131	E / cm^{-1}
DFT (raw)	994.47
DFT (scaled)	964.64
observed band	970

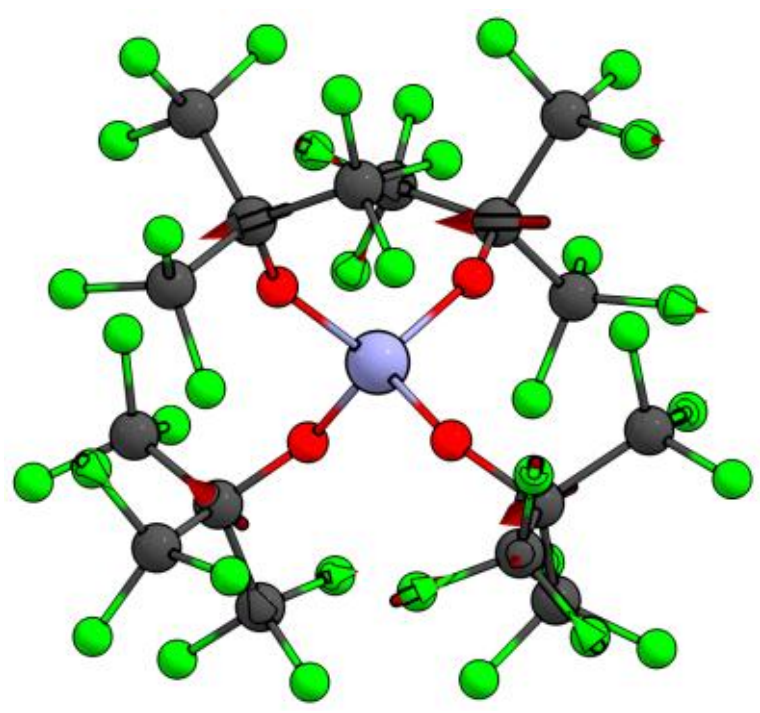

mode 130	E / cm^{-1}
DFT (raw)	994.04
DFT (scaled)	964.22
observed band	970

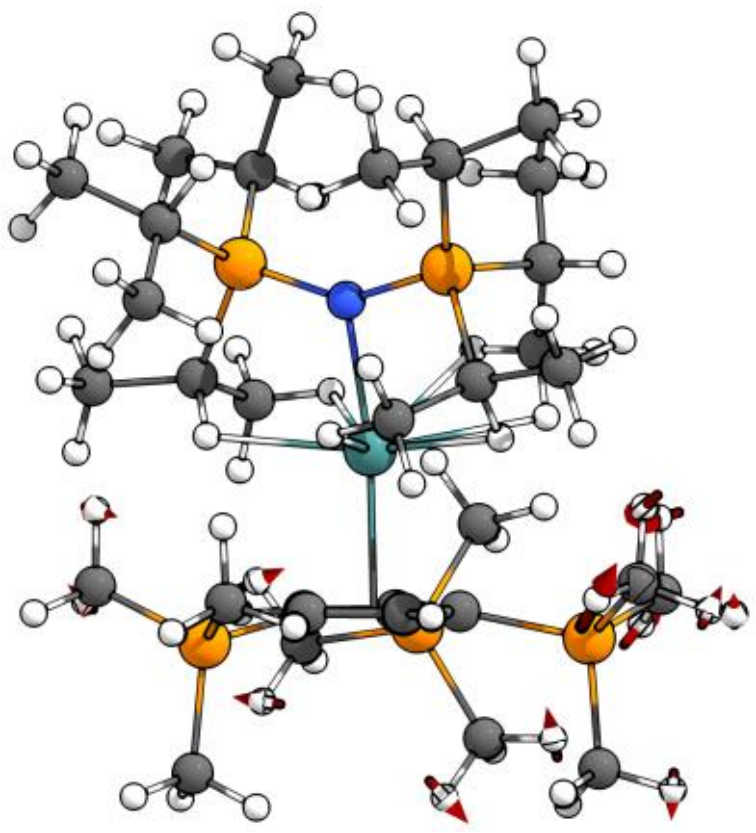

mode 133	E / cm^{-1}
DFT (raw)	852.04
DFT (scaled)	800.92
observed band	827

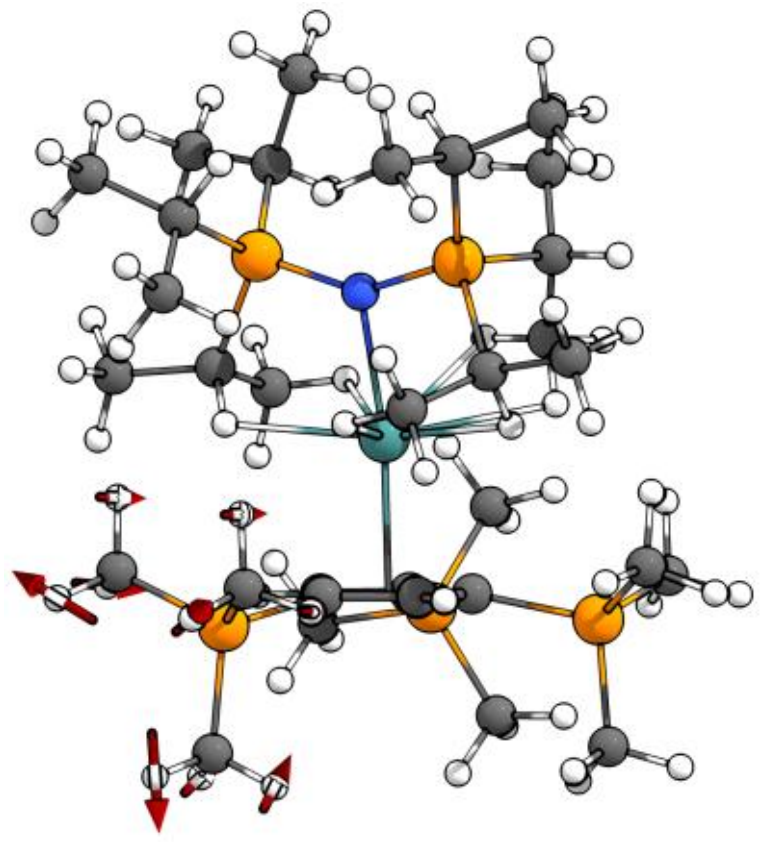

mode 129	E / cm^{-1}
DFT (raw)	843.38
DFT (scaled)	792.78
observed band	827

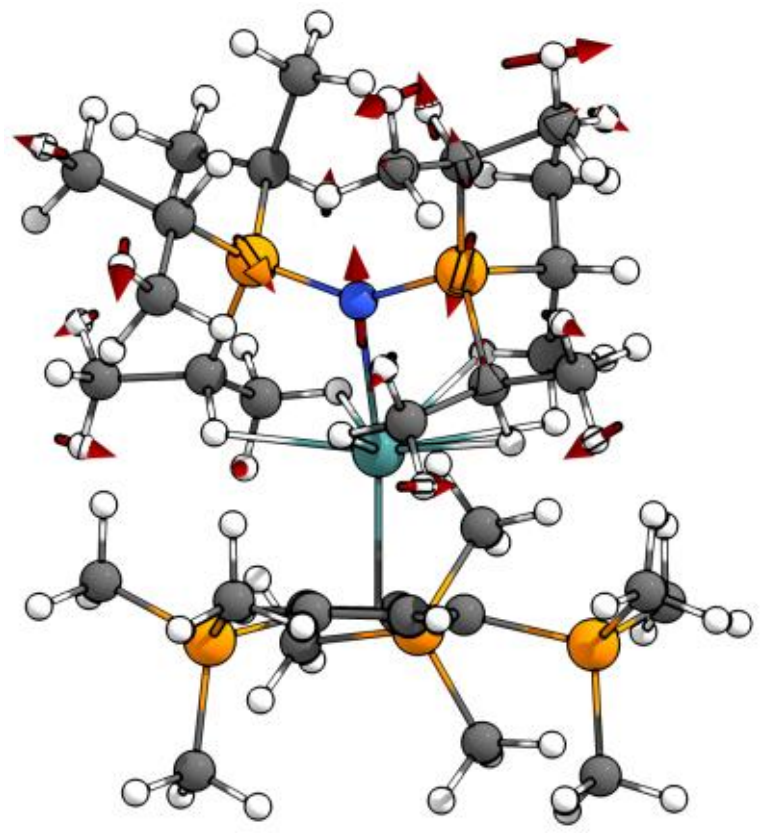

mode 121	E / cm^{-1}
DFT (raw)	719.65
DFT (scaled)	676.47
observed band	666

3.3 [Ln{N(Si^{i}Pr$_3$)$_2$}(Cp^{ttt})(BH$_4$)] (3-Ln)

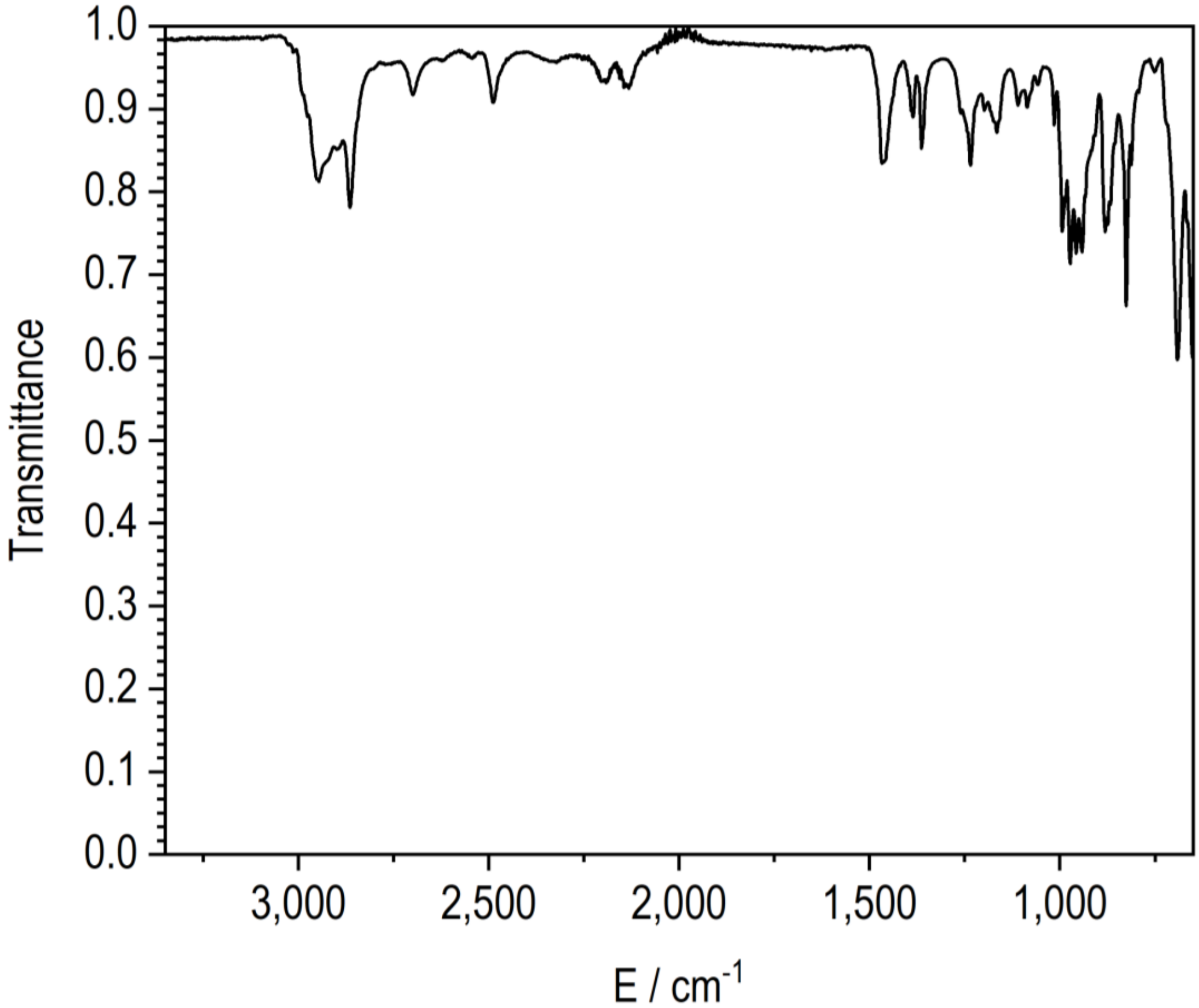

Figure S28. FT-IR (ATR, microcrystalline solid) spectrum of **3-Dy**.

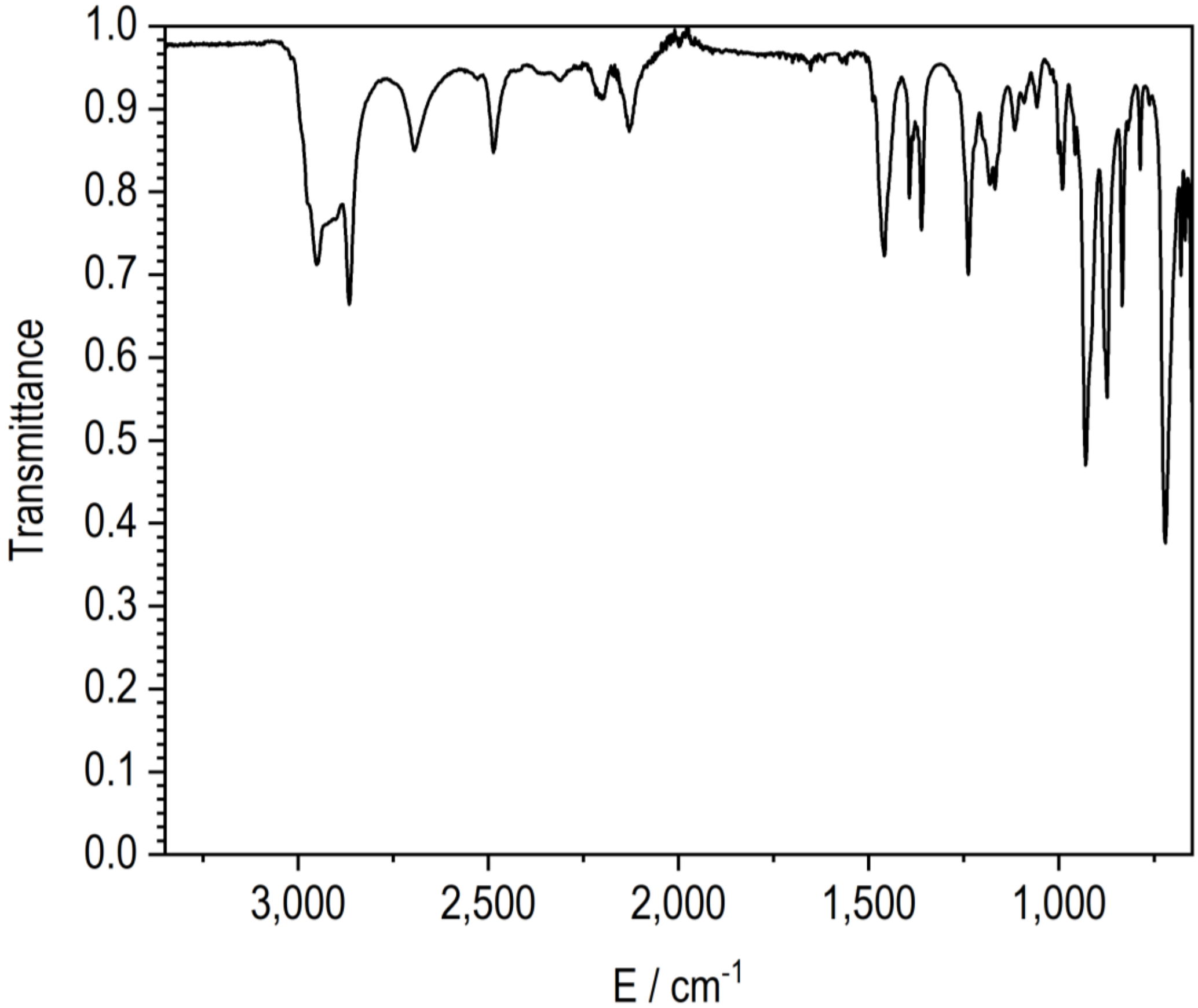

Figure S29. FT-IR (ATR, microcrystalline solid) spectrum of **3-Y**.

a.

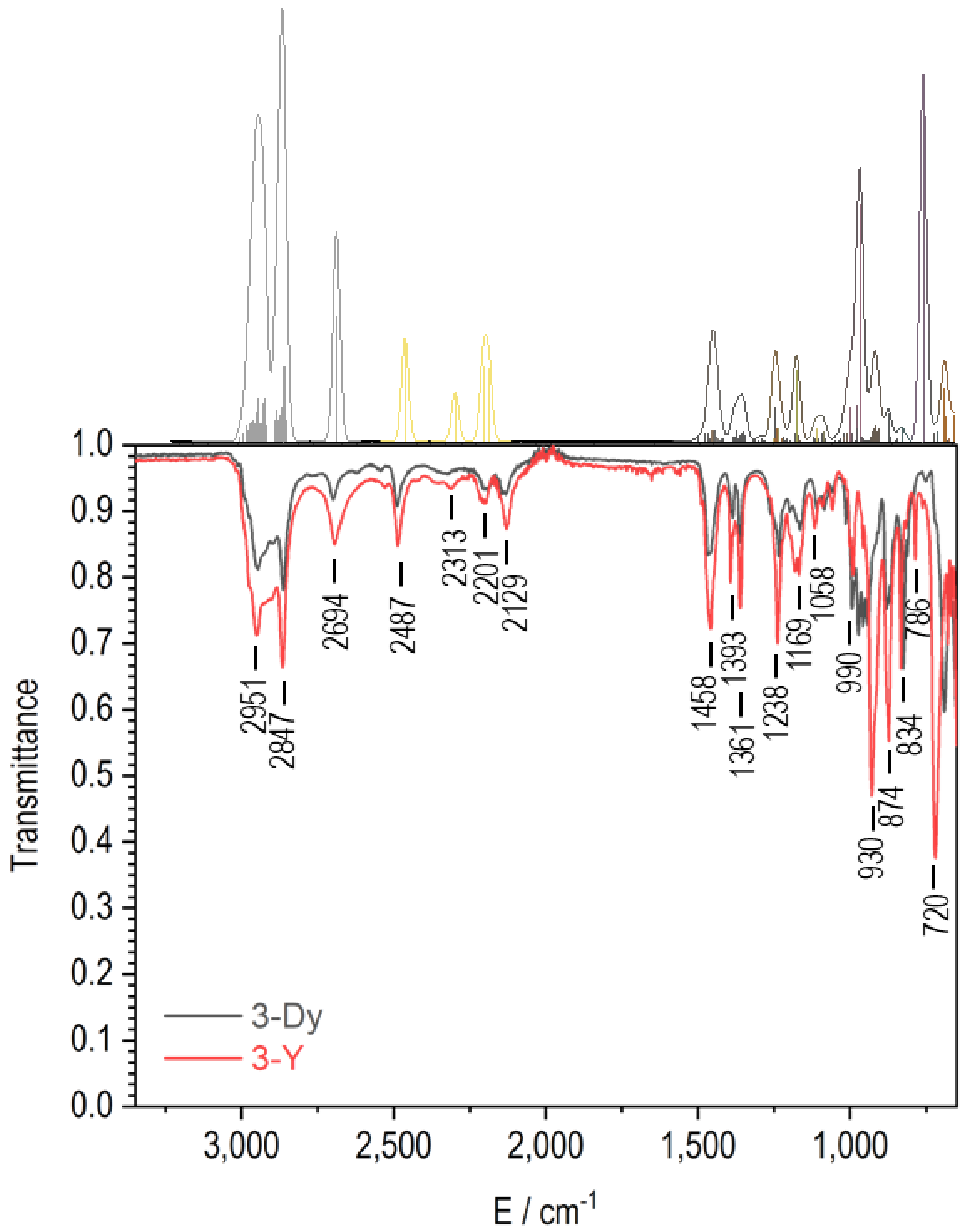

Figure S30. a. Overlay of the FT-IR (ATR, microcrystalline solid) spectrum of **3-Dy**, **3-Y**, and (above) the DFT-calculated vibrational modes of **3-Y**; empirical energy scale factor (0.94) and Lorentzian convolution (fwhm = 30 cm^{-1}) applied, coloured as a weighted average of the atoms involved in the vibration (B = yellow, C = black, N = blue, Si = orange, Y = teal). **b.** (Overleaf) Selected DFT-calculated vibrational modes of **3-Y**; atomic displacement vectors shown with red arrows scaled by the magnitude of the displacement.

b.

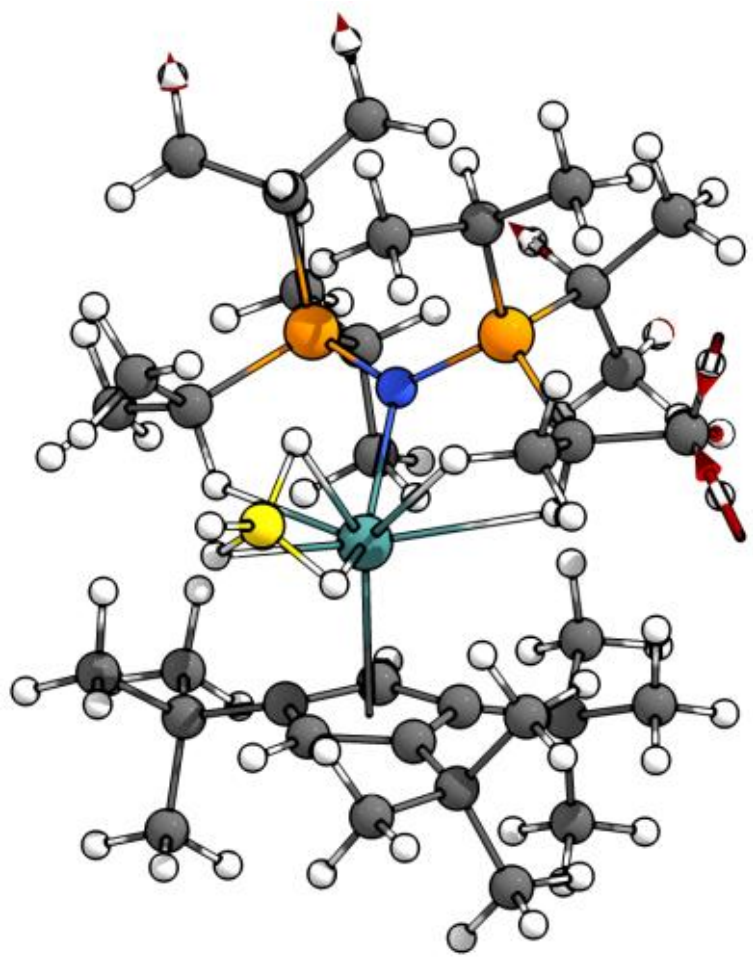

**mode 286**	**E / cm^{-1}**
DFT (raw)	3033.04
DFT (scaled)	3028.83
observed band	2951

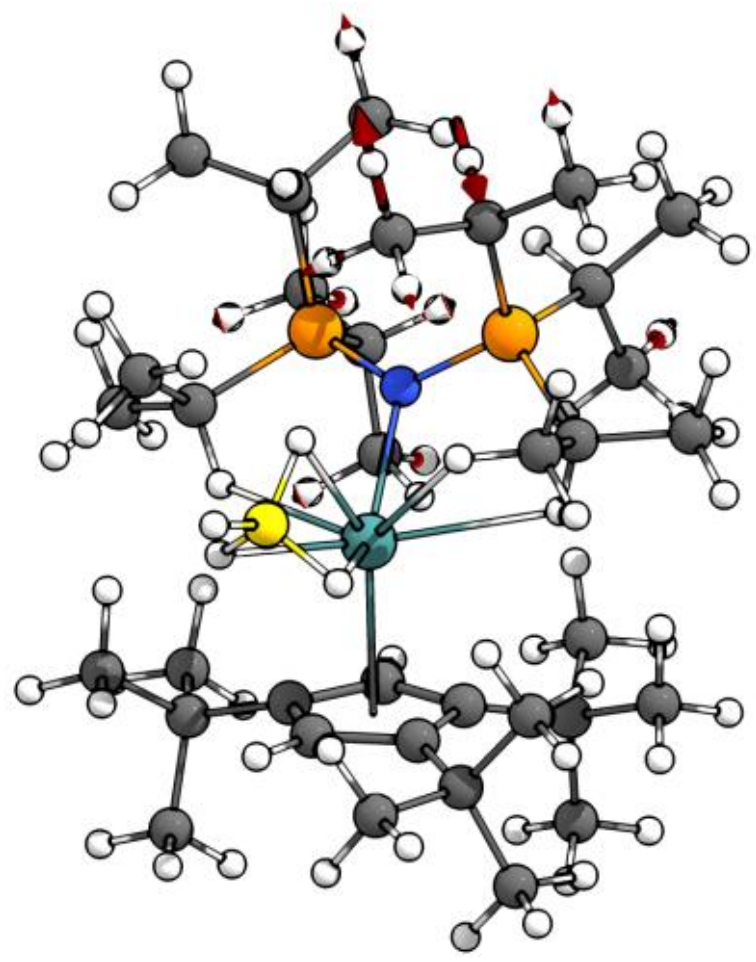

**mode 280**	**E / cm^{-1}**
DFT (raw)	3028.83
DFT (scaled)	2847.10
observed band	2865

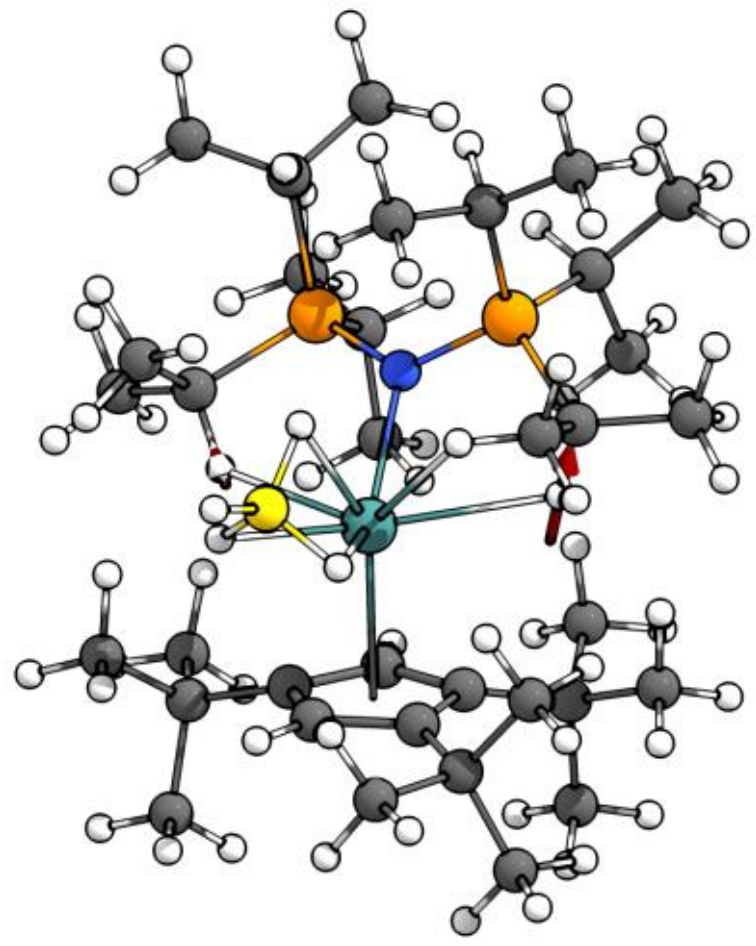

**mode 276**	**E / cm^{-1}**
DFT (raw)	2842.70
DFT (scaled)	2672.14
observed band	2694

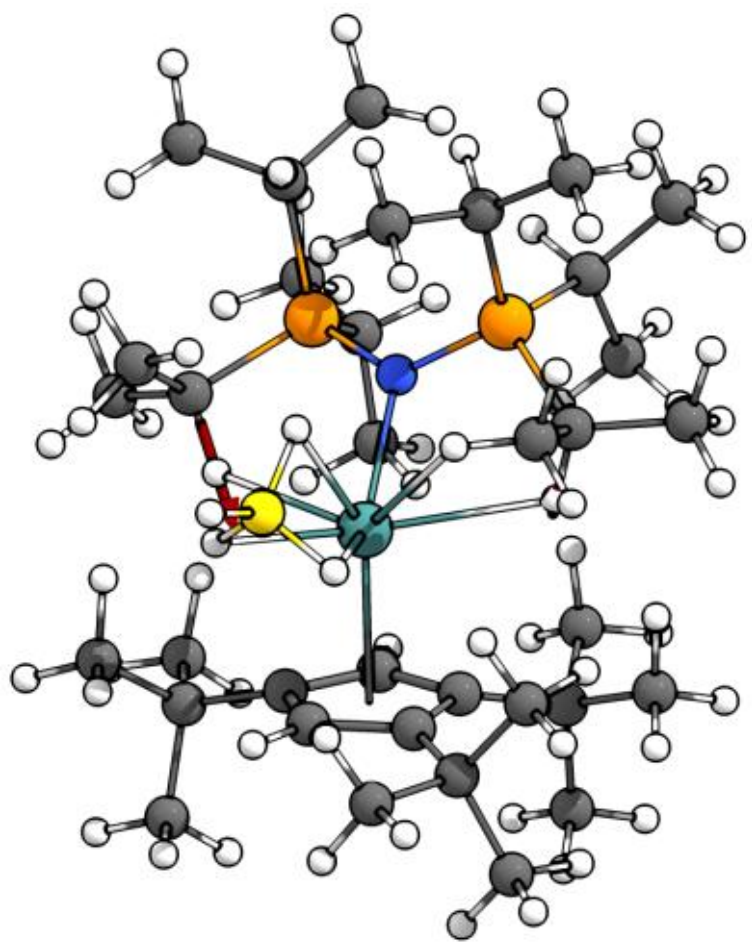

**mode 275**	**E / cm^{-1}**
DFT (raw)	2837.61
DFT (scaled)	2667.35
observed band	2694

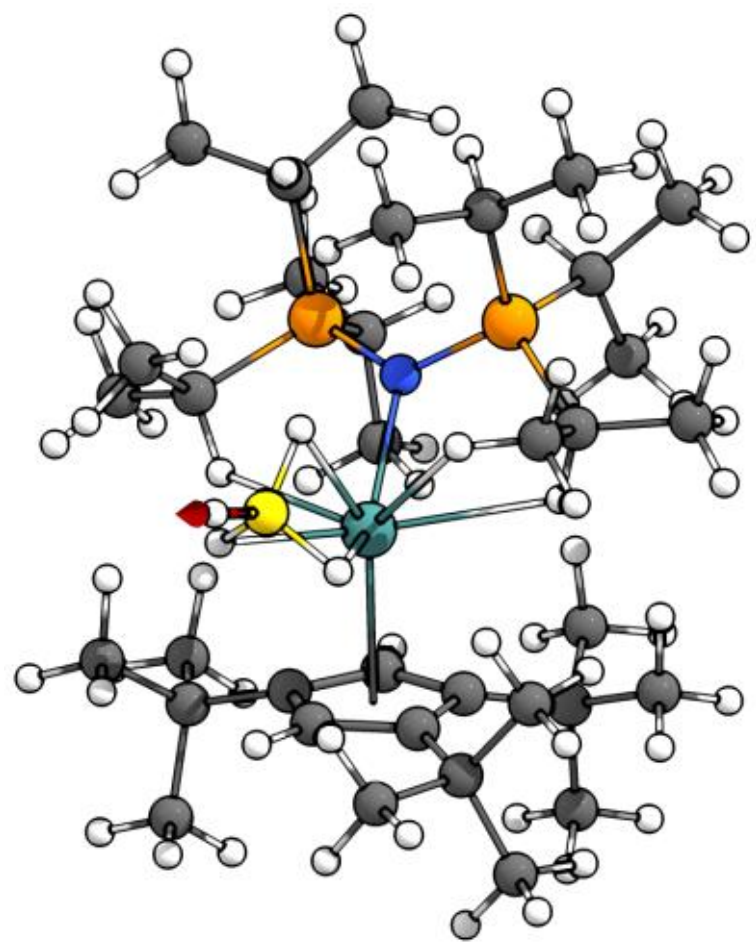

**mode 274**	**E / cm^{-1}**
DFT (raw)	2595.05
DFT (scaled)	2439.34
observed band	2487

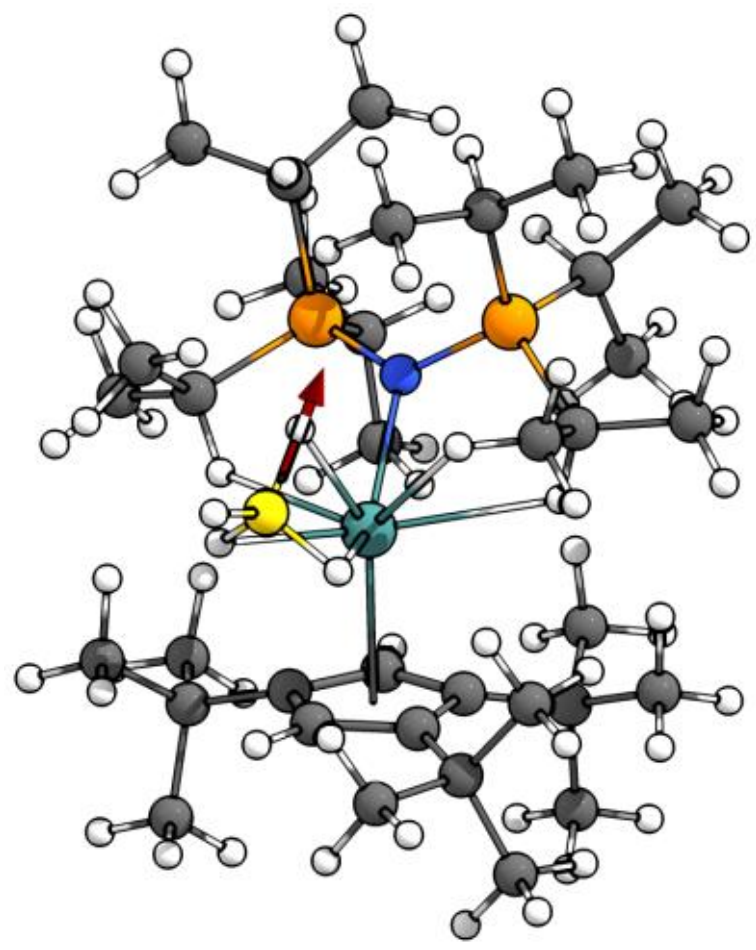

**mode 273**	**E / cm^{-1}**
DFT (raw)	2415.47
DFT (scaled)	2270.54
observed band	2313

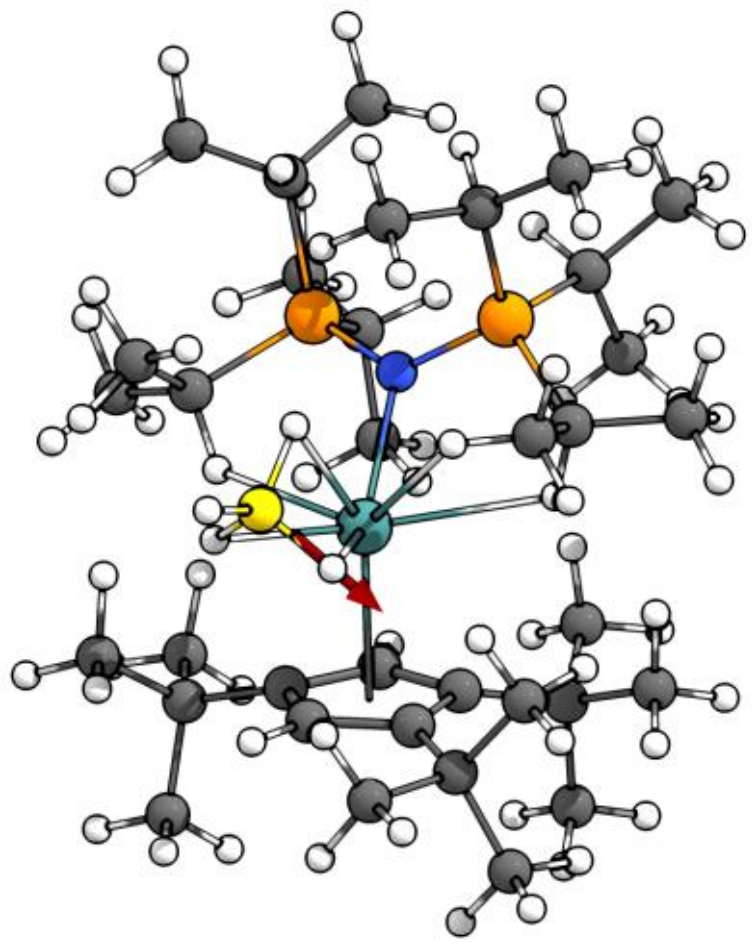

**mode 272**	**E / cm^{-1}**
DFT (raw)	2315.49
DFT (scaled)	2176.56
observed band	2201

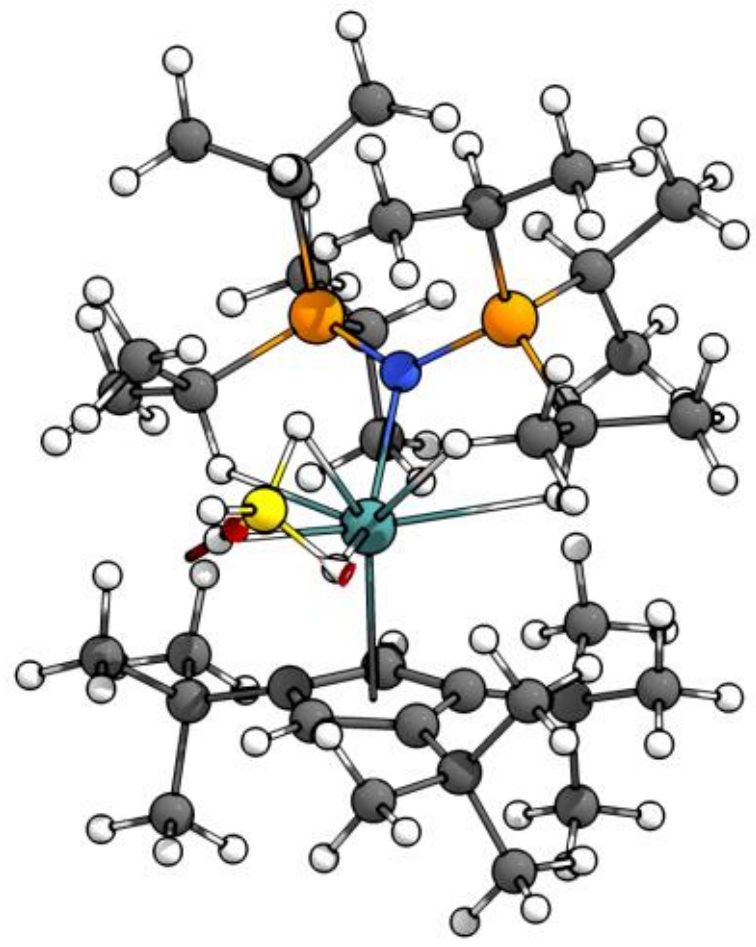

**mode 271**	**E / cm^{-1}**
DFT (raw)	2295.21
DFT (scaled)	2157.50
observed band	2129

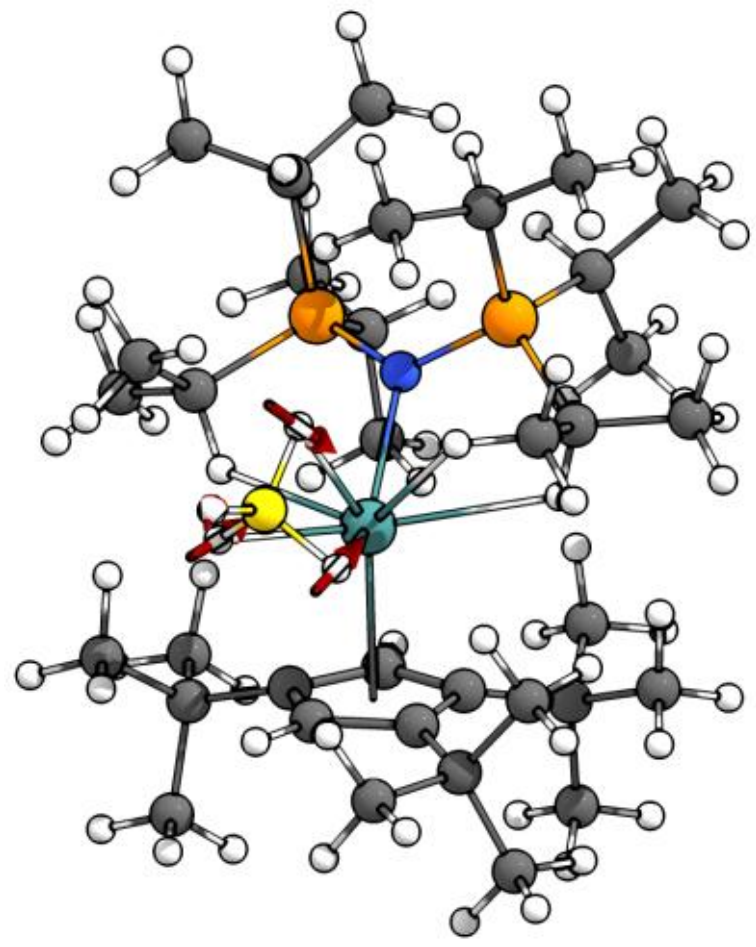

**mode 179**	**E / cm^{-1}**
DFT (raw)	1193.91
DFT (scaled)	1122.28
observed band	1116

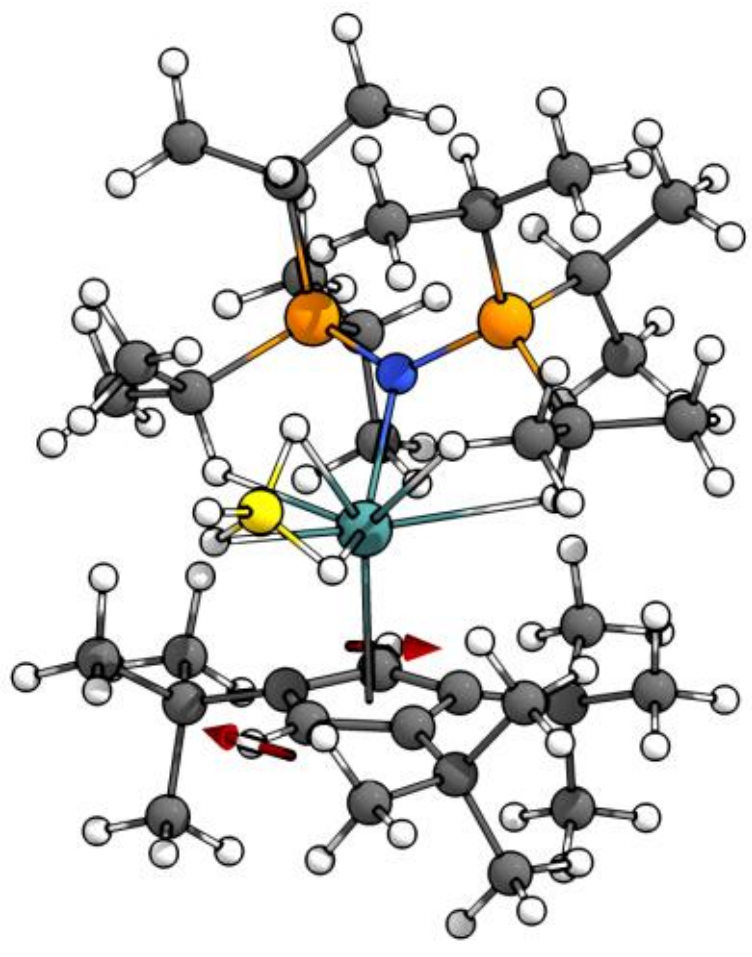

**mode 175**	**E / cm^{-1}**
DFT (raw)	1188.29
DFT (scaled)	1116.99
observed band	1116

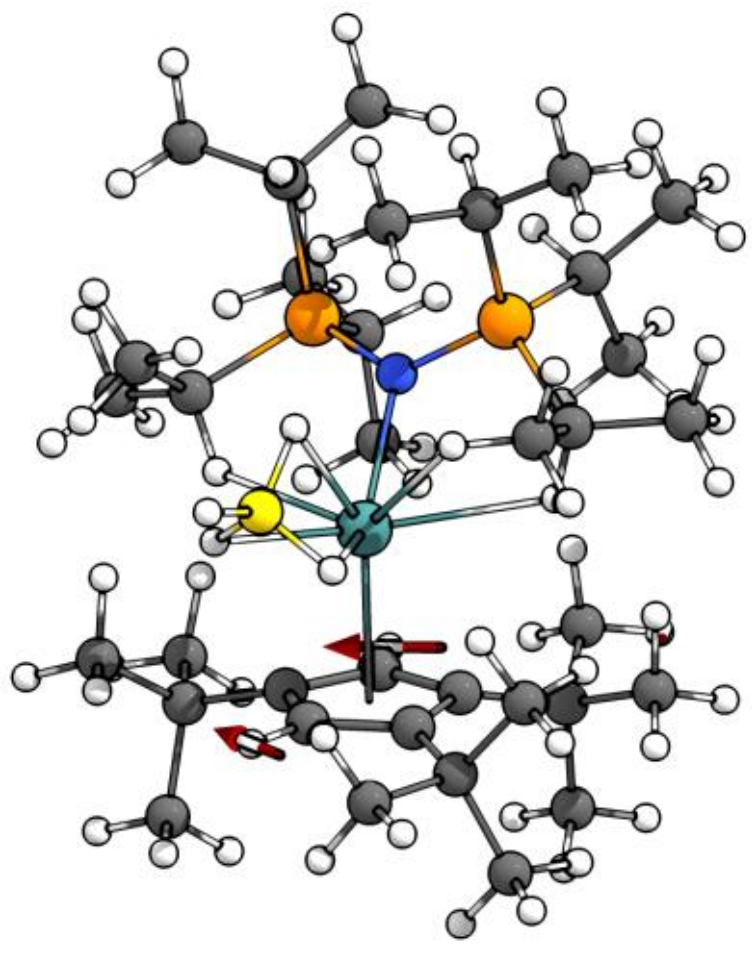

**mode 171**	**E / cm^{-1}**
DFT (raw)	1133.29
DFT (scaled)	1065.29
observed band	1059

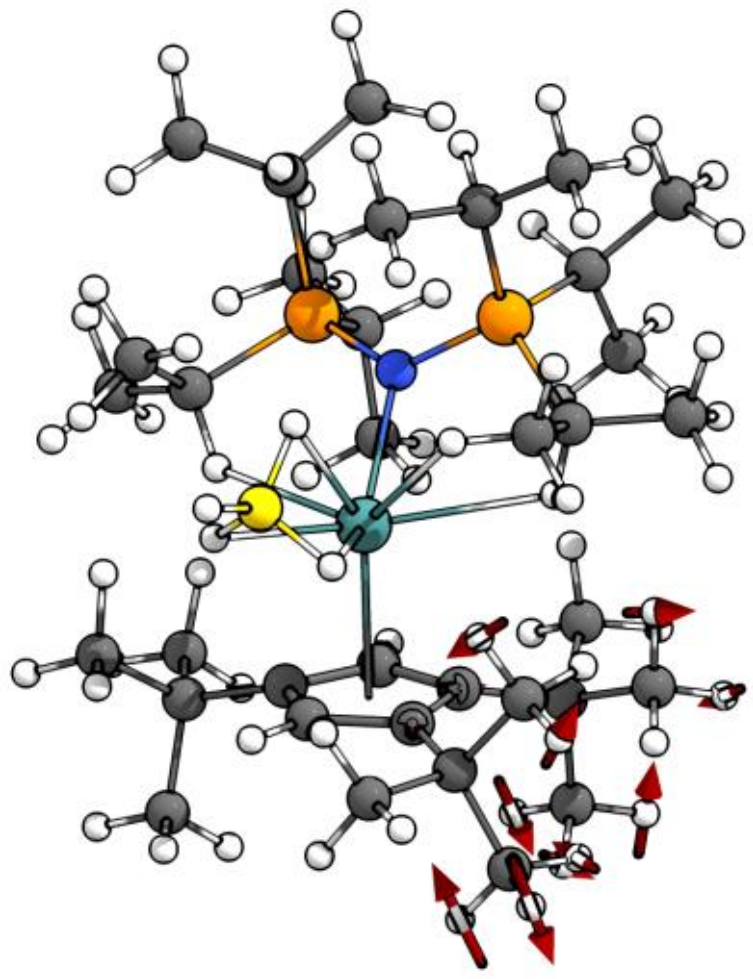

**mode 156**	**E / cm^{-1}**
DFT (raw)	1026.94
DFT (scaled)	965.32
observed band	991

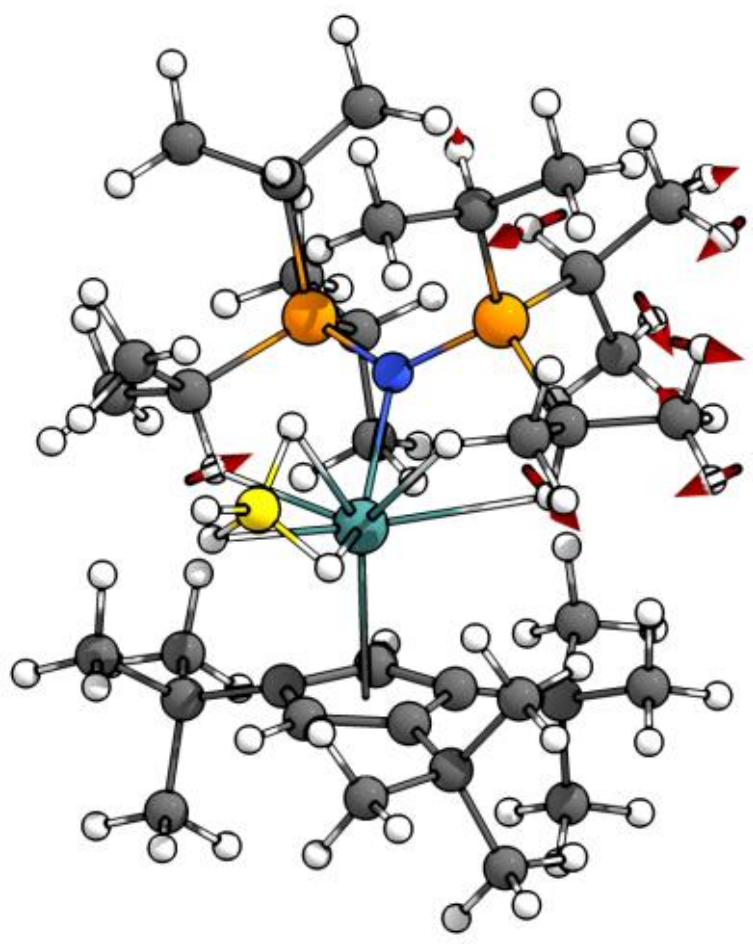

**mode 152**	**E / cm^{-1}**
DFT (raw)	1002.06
DFT (scaled)	941.94
observed band	930

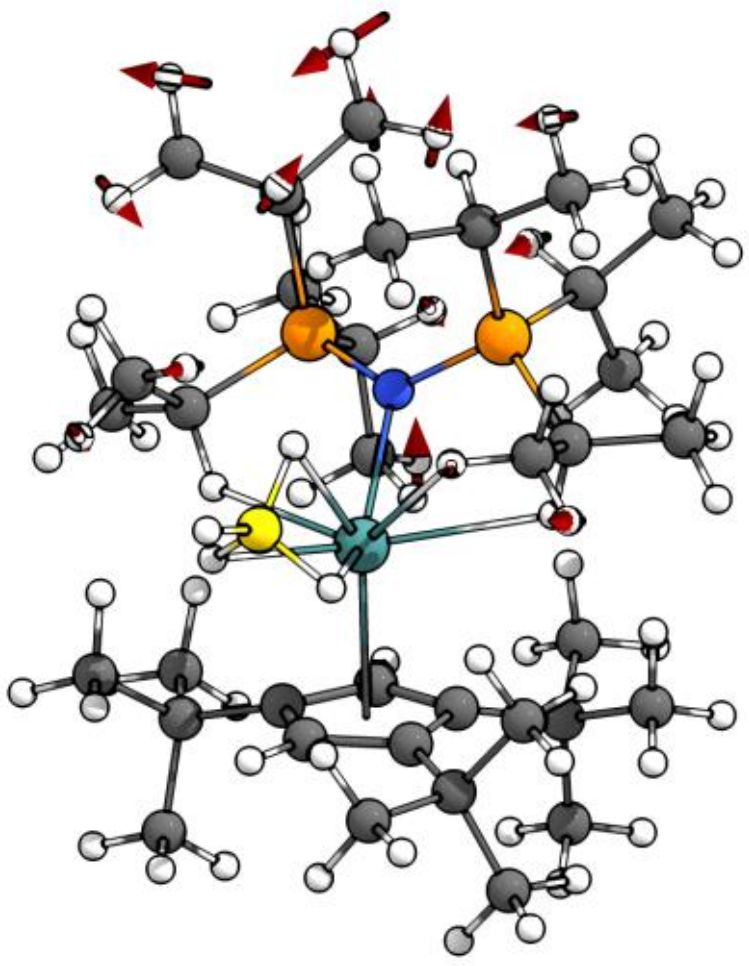

**mode 146**	**E / cm^{-1}**
DFT (raw)	980.61
DFT (scaled)	921.77
observed band	930

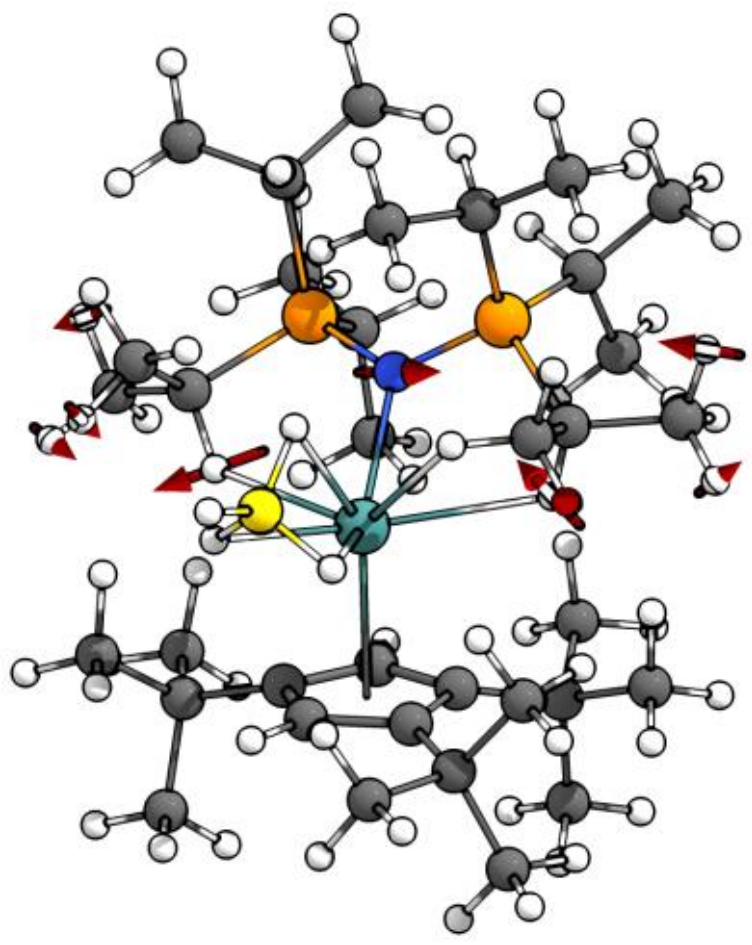

**mode 142**	**E / cm^{-1}**
DFT (raw)	965.64
DFT (scaled)	907.70
observed band	874

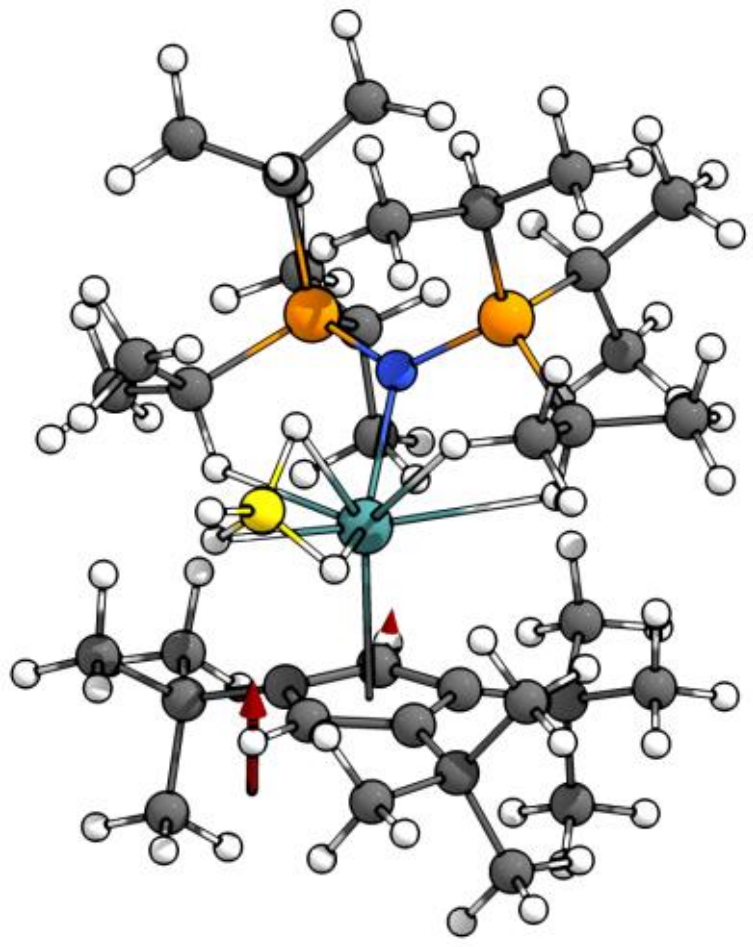

**mode 120**	**E / cm^{-1}**
DFT (raw)	865.94
DFT (scaled)	813.98
observed band	834

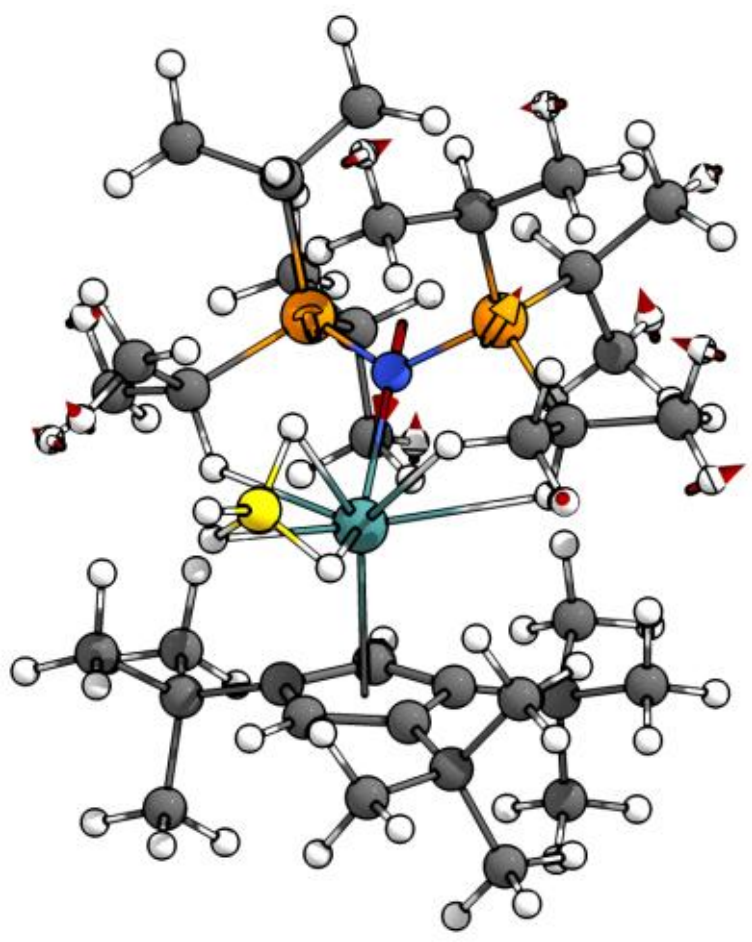

**mode 115**	**E / cm^{-1}**
DFT (raw)	740.61
DFT (scaled)	696.17
observed band	720

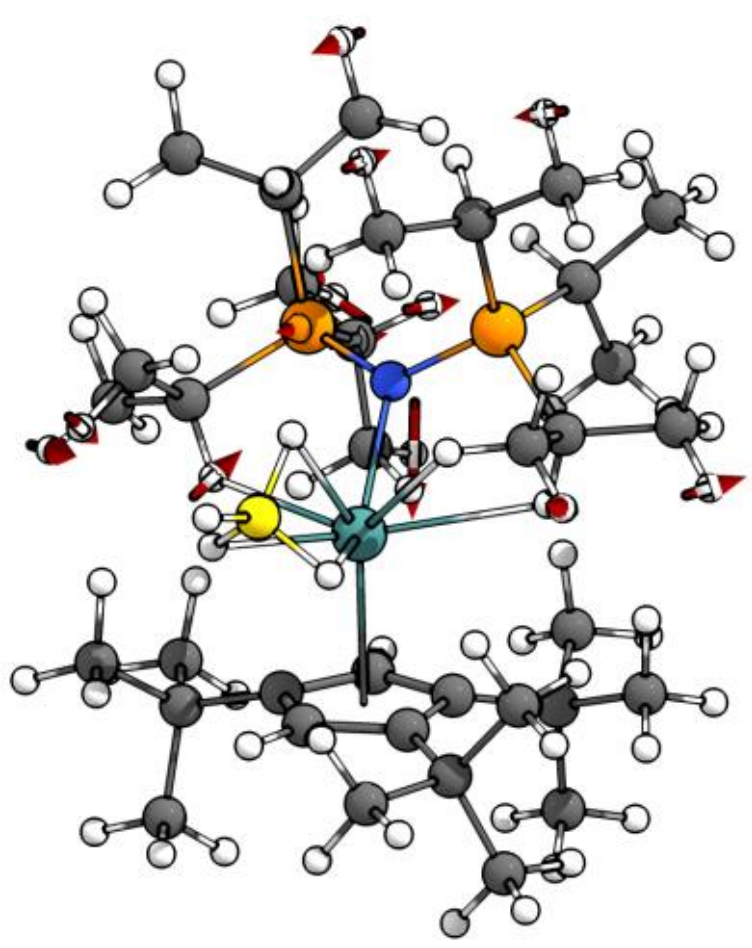

**mode 112**	**E / cm^{-1}**
DFT (raw)	665.47
DFT (scaled)	625.54
observed band	686

3.4 [Ln{N(Si^{i}Pr$_3$)$_2$}(Cp′′′)(BH$_4$)] (4-Ln)

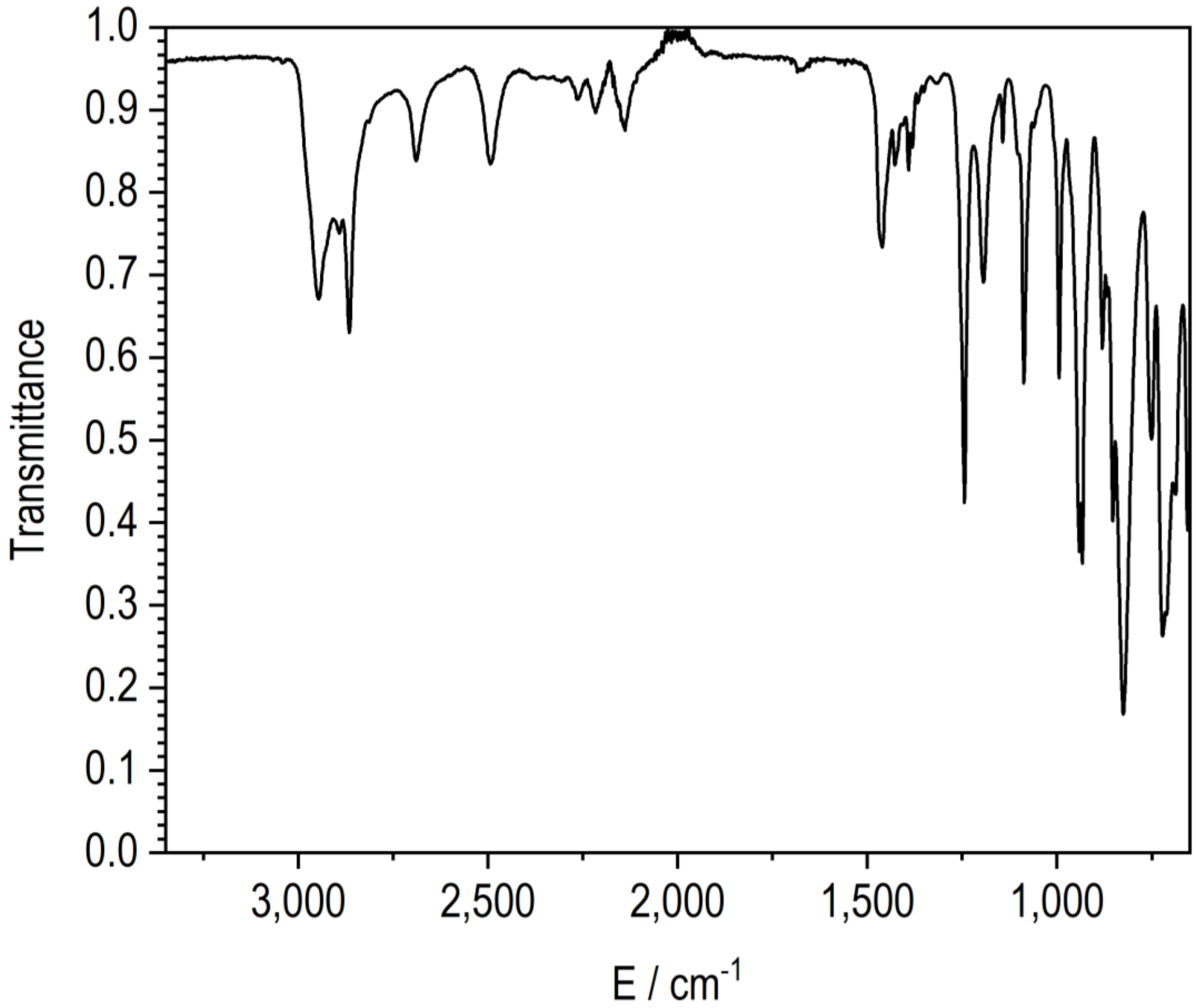

Figure S31. FT-IR (ATR, microcrystalline solid) spectrum of **4-Dy**.

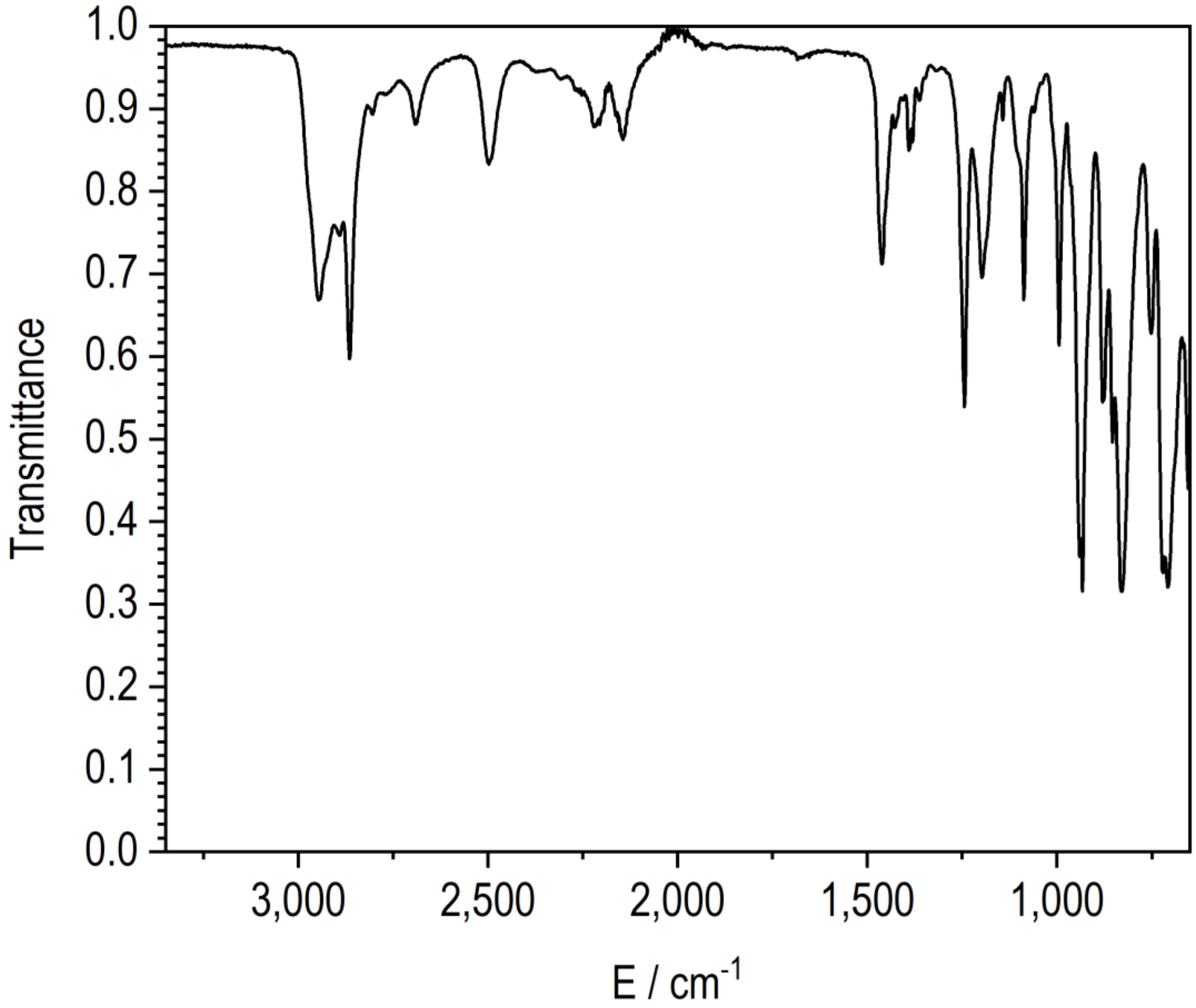

Figure S32. FT-IR (ATR, microcrystalline solid) spectrum of **4-Y**.

a.

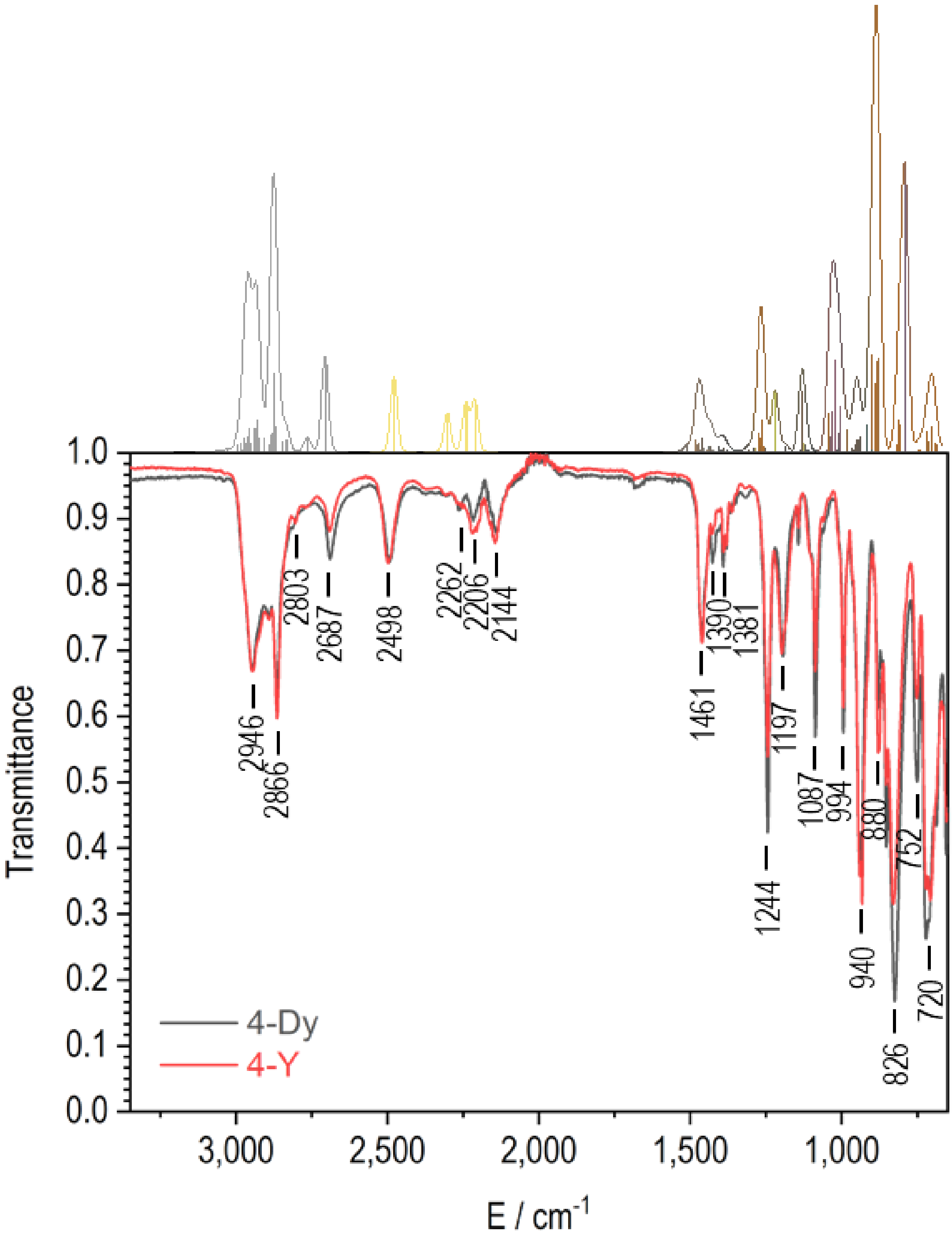

Figure S33. a. Overlay of the FT-IR (ATR, microcrystalline solid) spectrum of **4-Dy**, **4-Y**, and (above) the DFT-calculated vibrational modes of **4-Y**; empirical energy scale factor (0.94) and Lorentzian convolution (fwhm = 30 cm^{-1}) applied, coloured as a weighted average of the atoms involved in the vibration (B = yellow, C = black, N = blue, Si = orange, Y = teal). **b.** (Overleaf) Selected DFT-calculated vibrational modes of **4-Y**; atomic displacement vectors shown with red arrows scaled by the magnitude of the displacement.

b.

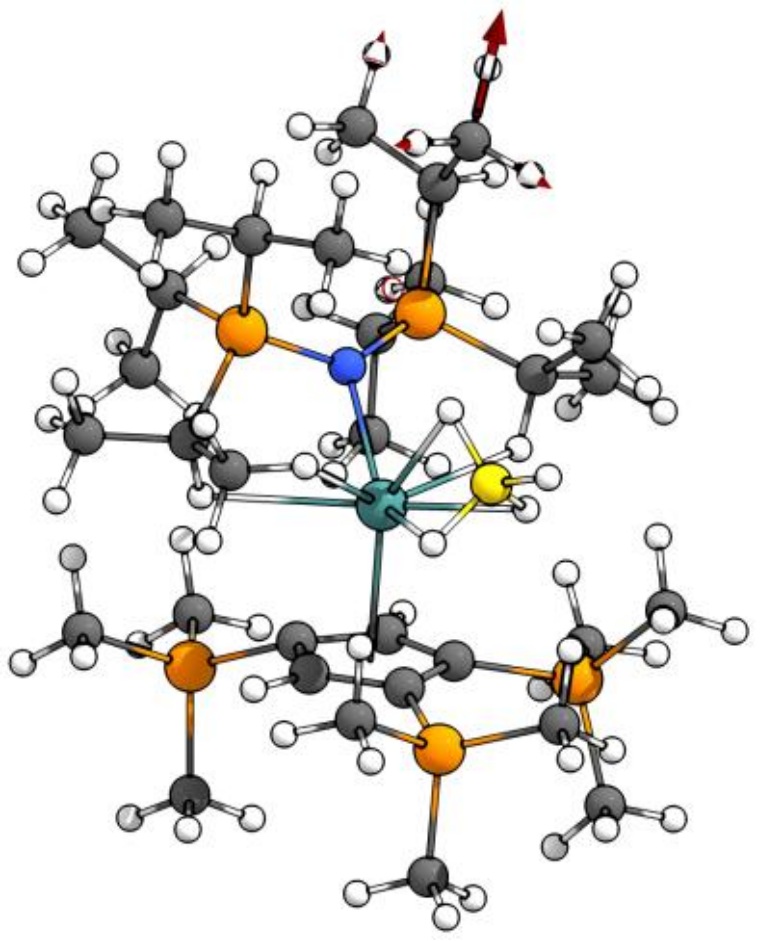

mode 289	E / cm^{-1}
DFT (raw)	3033.69
DFT (scaled)	2851.67
observed band	2865

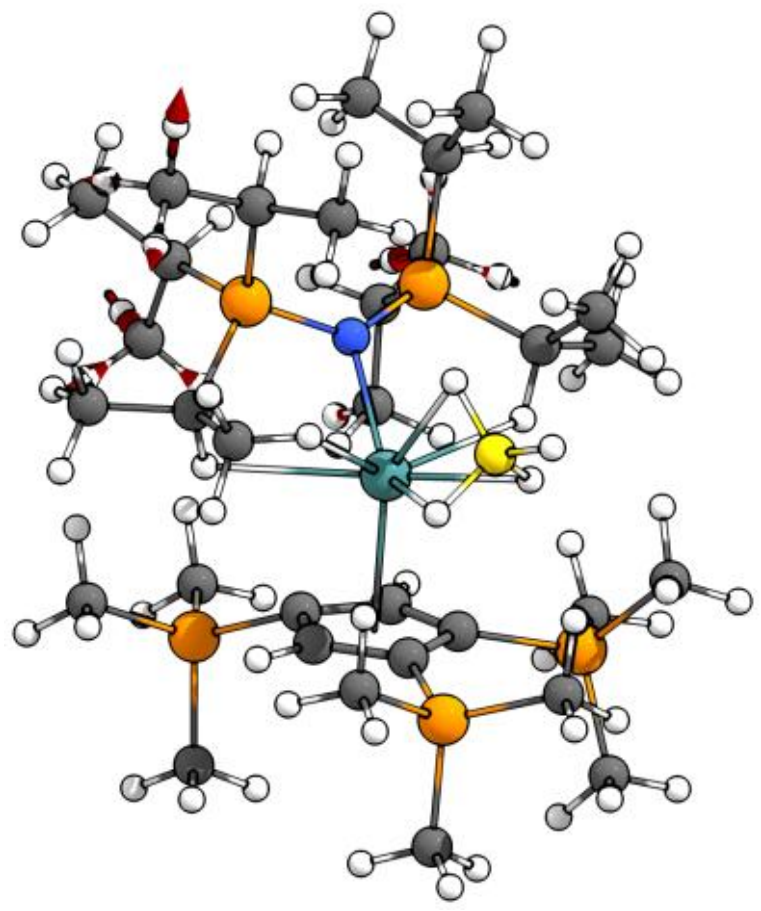

mode 286	E / cm^{-1}
DFT (raw)	3031.38
DFT (scaled)	2849.50
observed band	2865

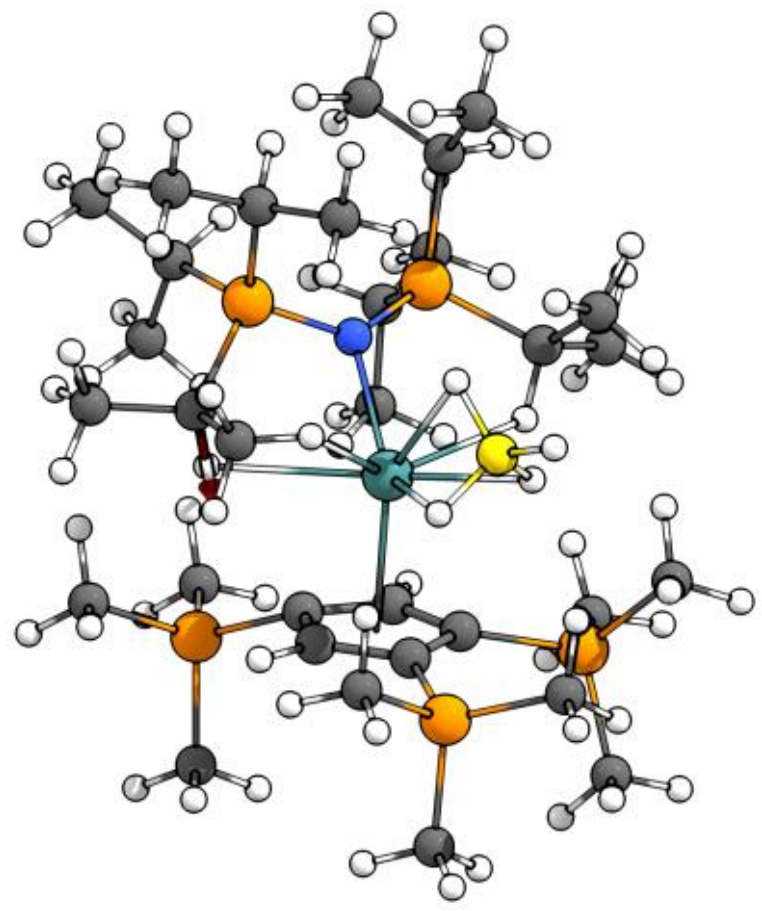

mode 276	E / cm^{-1}
DFT (raw)	2910.67
DFT (scaled)	2736.03
observed band	2803

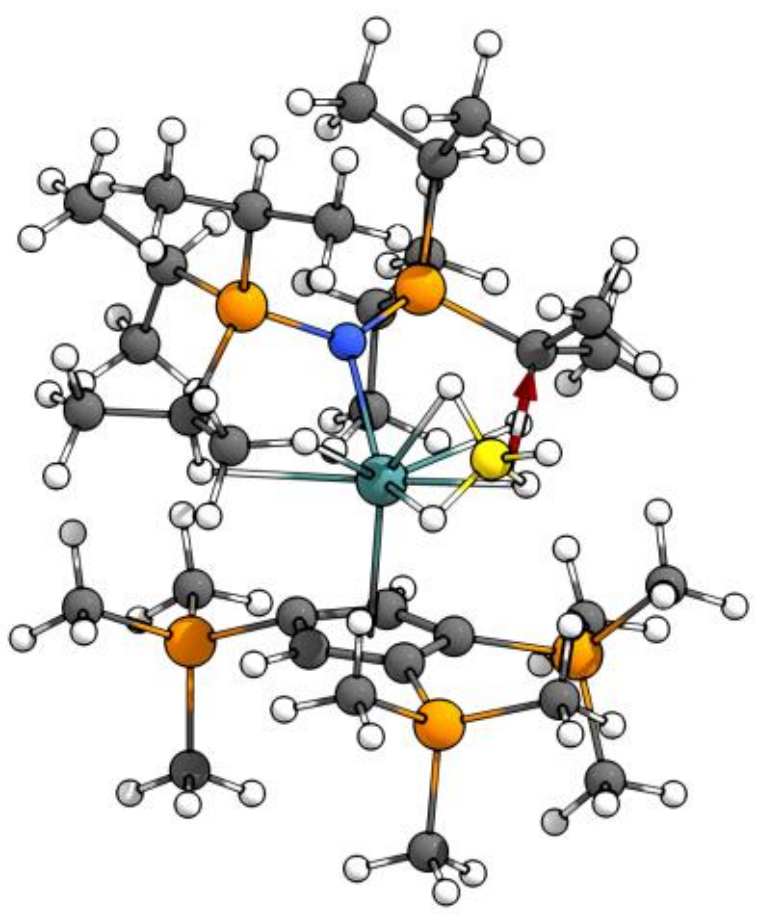

mode 275	E / cm^{-1}
DFT (raw)	2846.65
DFT (scaled)	2675.85
observed band	2689

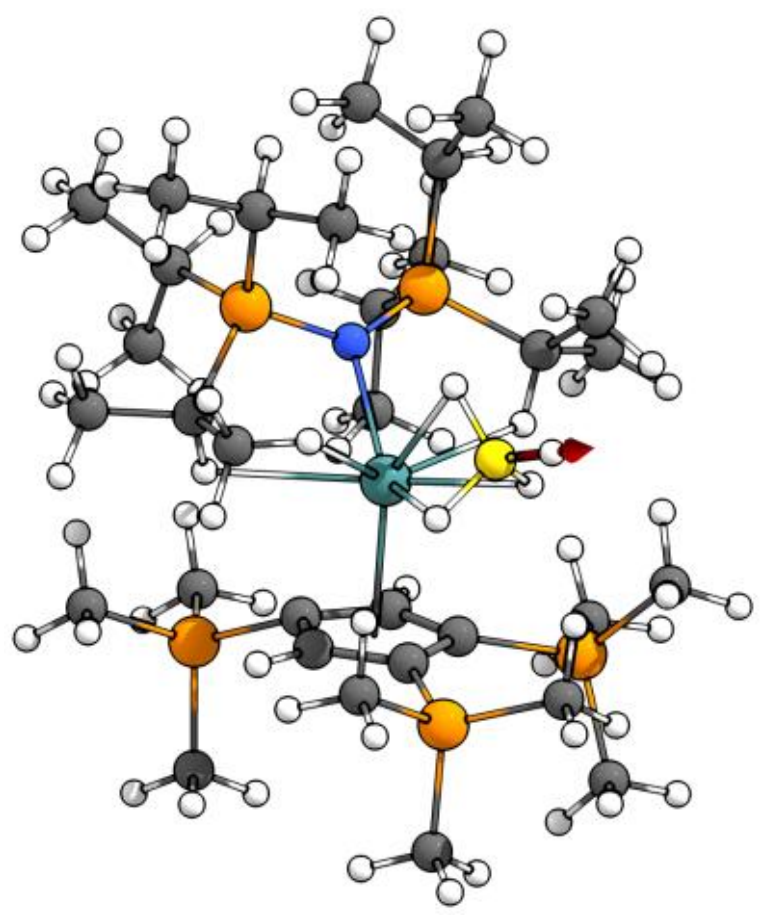

mode 274	E / cm^{-1}
DFT (raw)	2596.21
DFT (scaled)	2440.44
observed band	2494

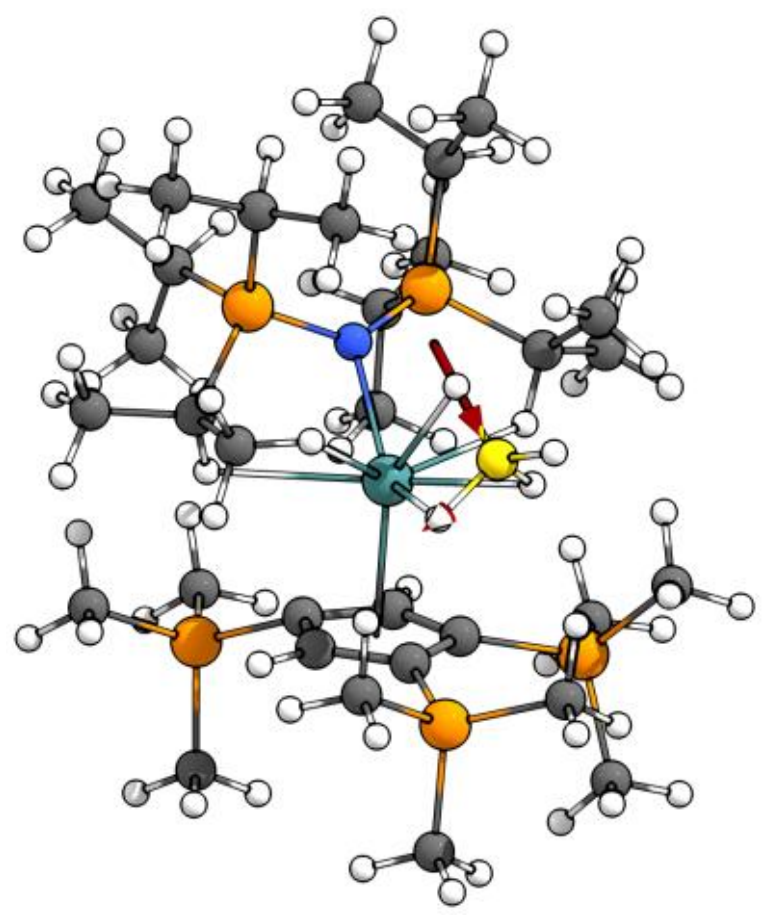

mode 273	E / cm^{-1}
DFT (raw)	2402.64
DFT (scaled)	2258.48
observed band	2262

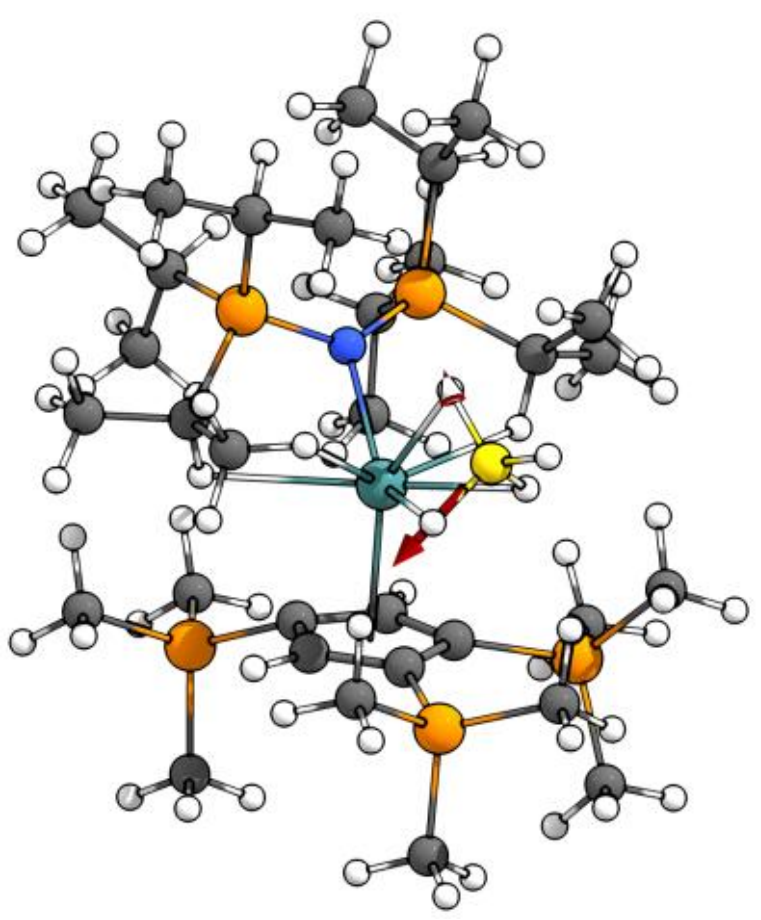

mode 272	E / cm^{-1}
DFT (raw)	2335.36
DFT (scaled)	2195.24
observed band	2206

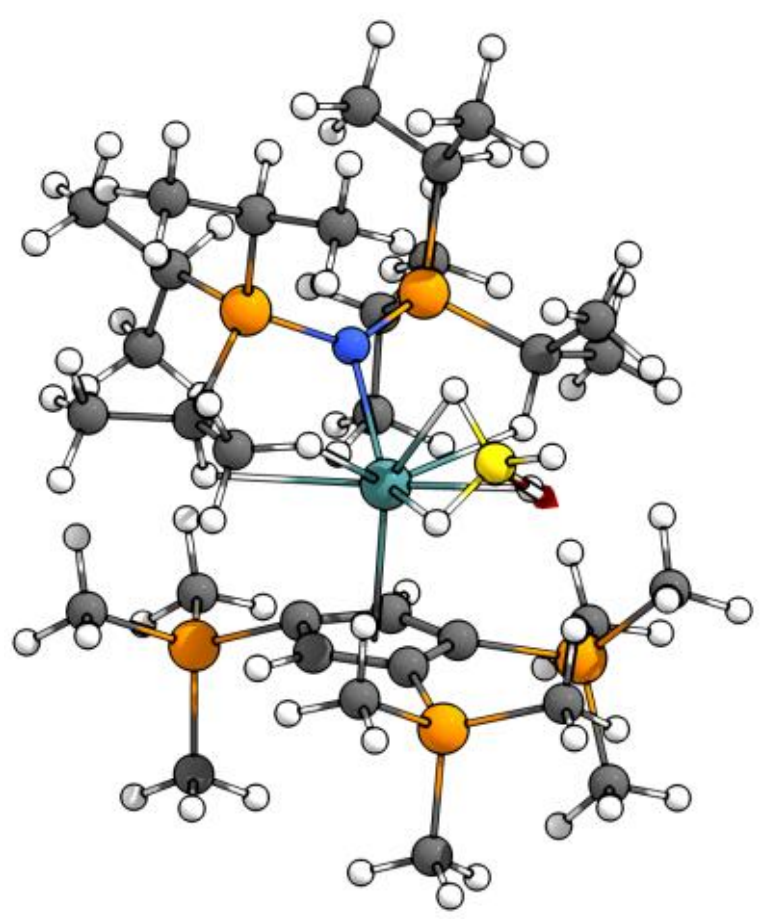

mode 271	E / cm^{-1}
DFT (raw)	2302.90
DFT (scaled)	2164.73
observed band	2144

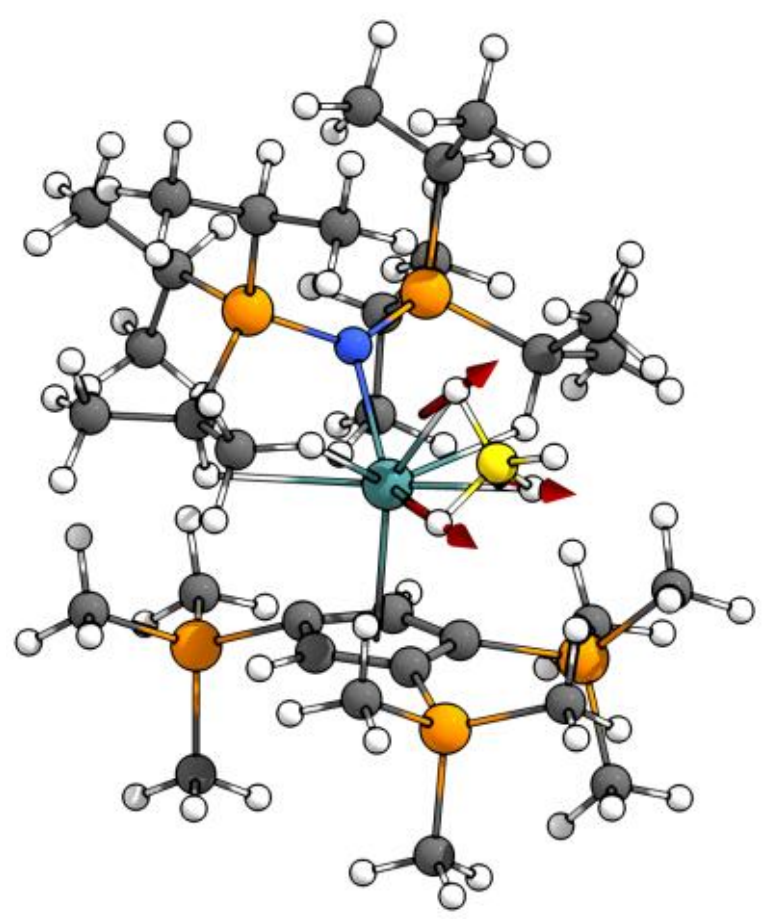

mode 188	E / cm^{-1}
DFT (raw)	1213.26
DFT (scaled)	1140.46
observed band	1197

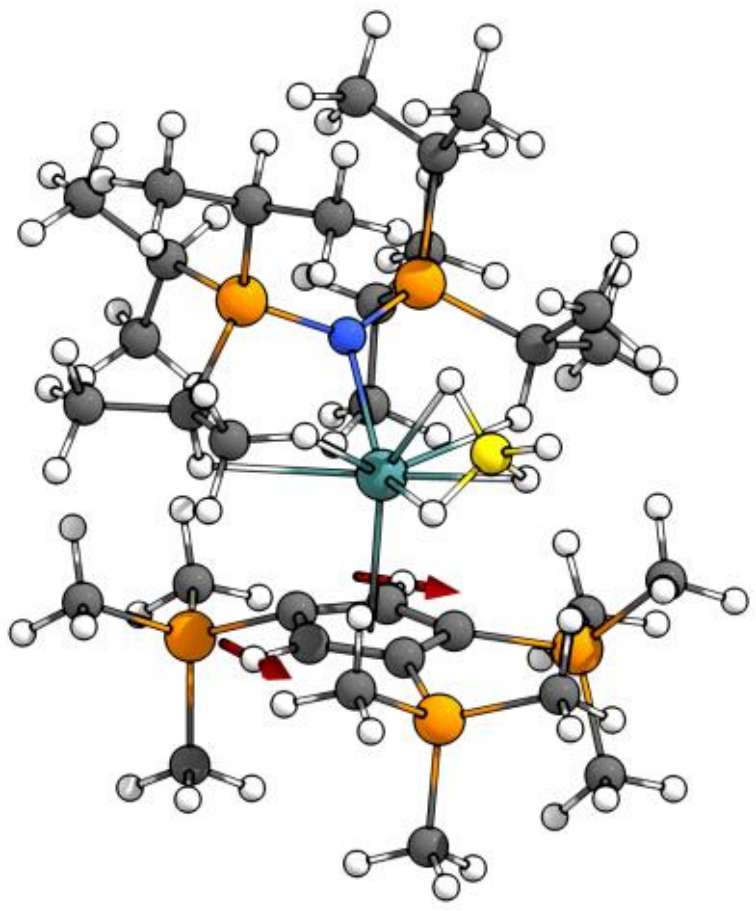

**mode 178**	**E / cm^{-1}**
DFT (raw)	1115.74
DFT (scaled)	1048.80
observed band	1087

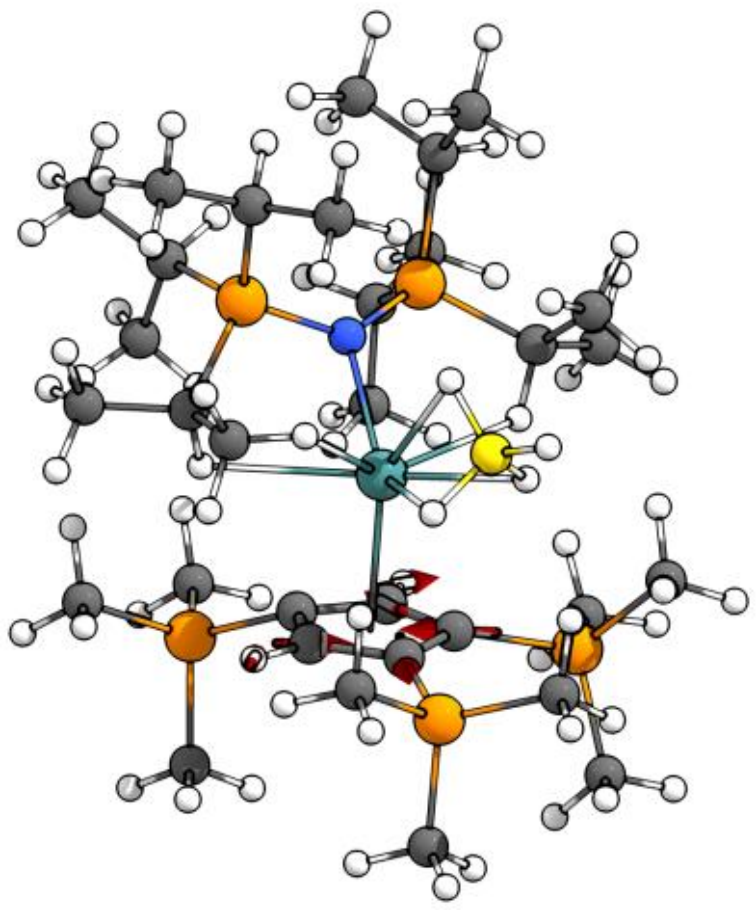

**mode 170**	**E / cm^{-1}**
DFT (raw)	1020.06
DFT (scaled)	958.86
observed band	994

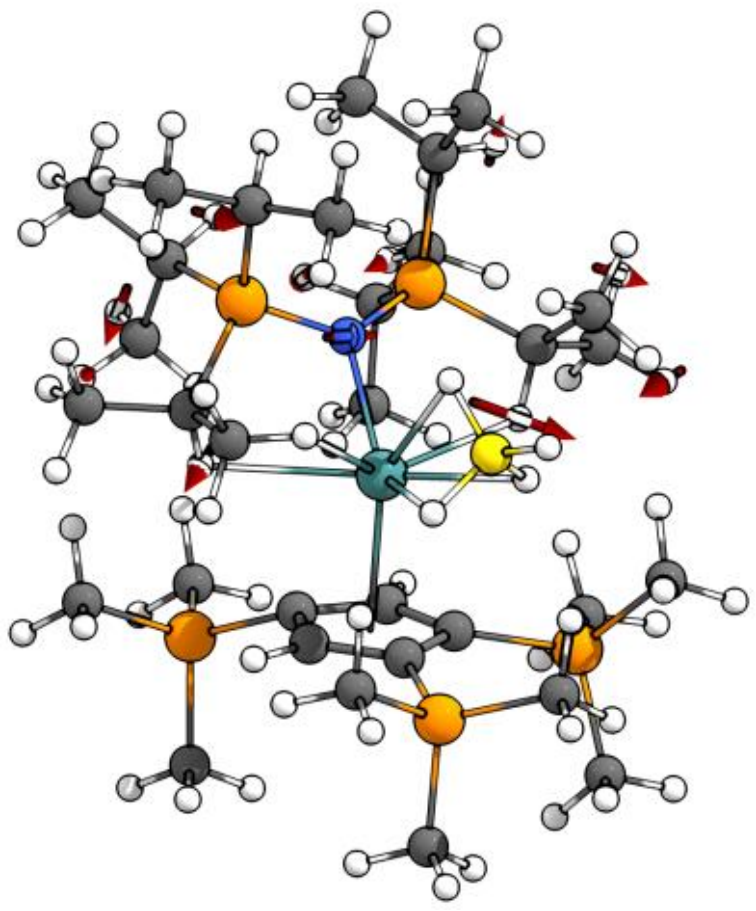

**mode 166**	**E / cm^{-1}**
DFT (raw)	999.68
DFT (scaled)	939.70
observed band	940

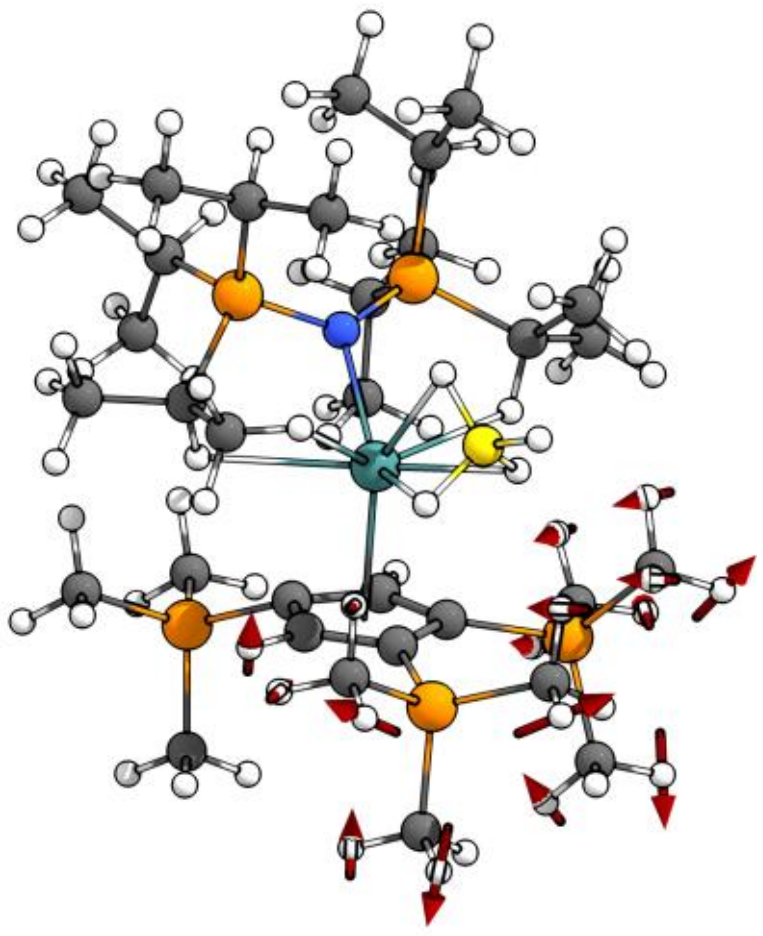

**mode 143**	**E / cm^{-1}**
DFT (raw)	863.16
DFT (scaled)	811.37
observed band	826

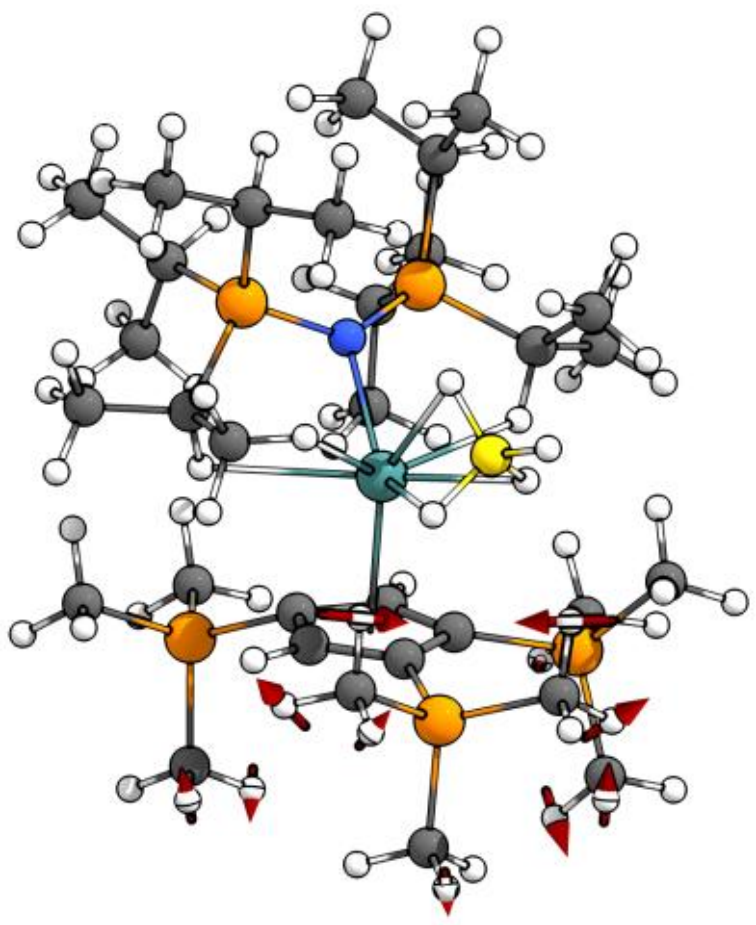

**mode 140**	**E / cm^{-1}**
DFT (raw)	850.65
DFT (scaled)	799.61
observed band	826

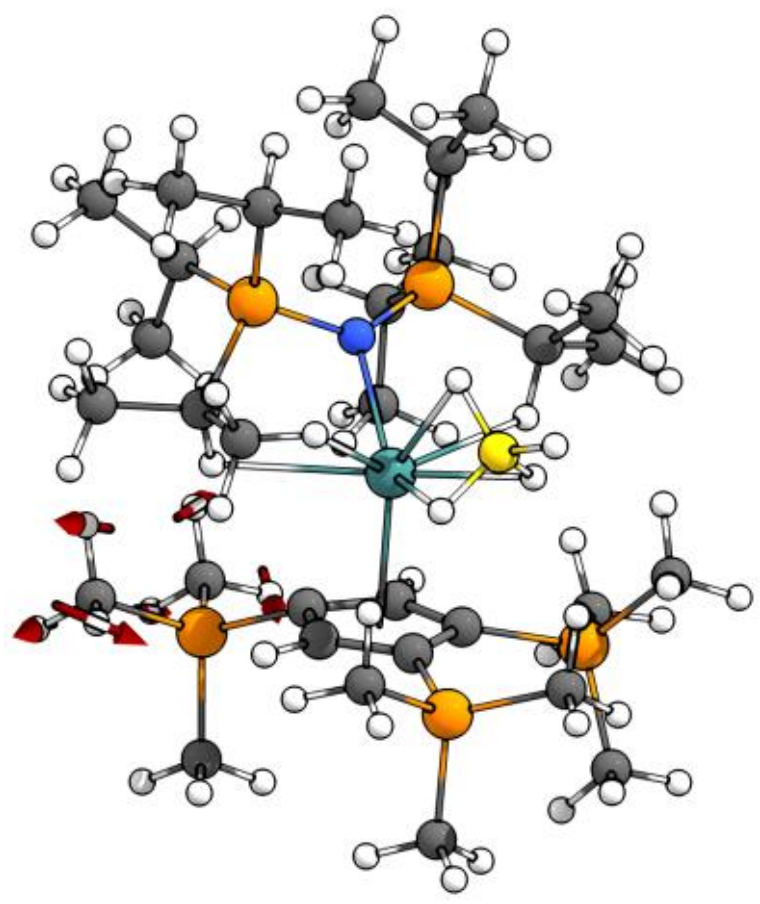

**mode 136**	**E / cm^{-1}**
DFT (raw)	844.21
DFT (scaled)	793.56
observed band	826

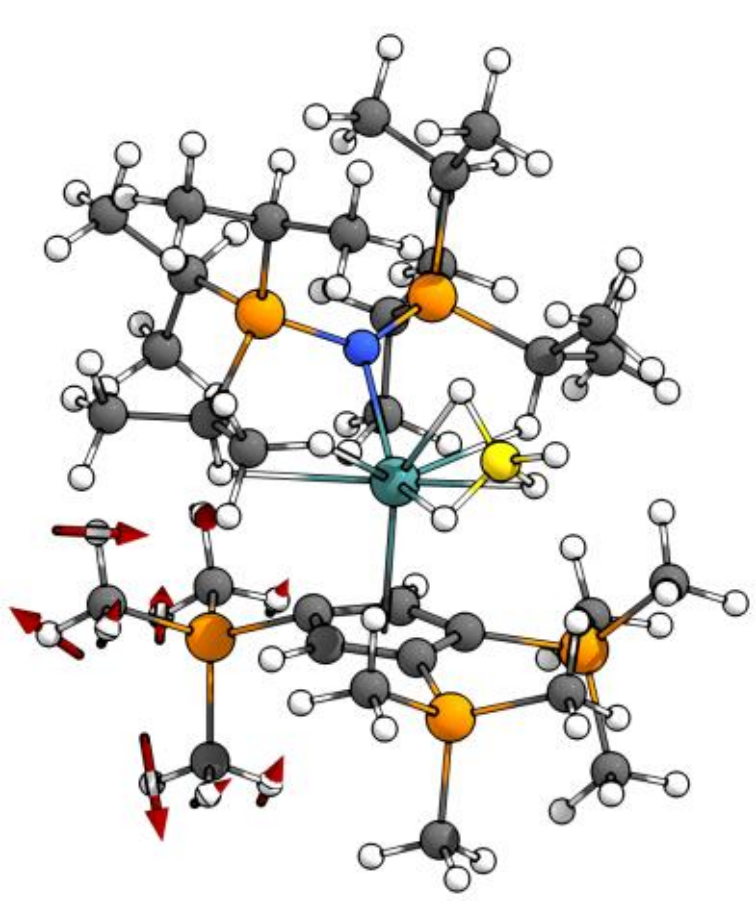

**mode 135**	**E / cm^{-1}**
DFT (raw)	843.72
DFT (scaled)	793.10
observed band	826

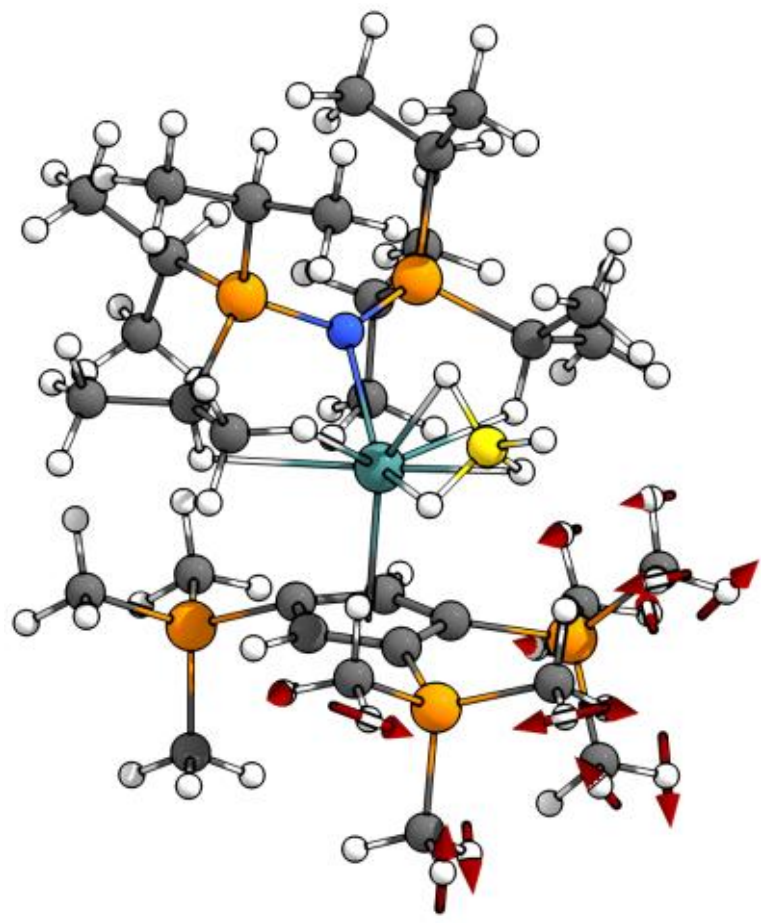

**mode 134**	**E / cm^{-1}**
DFT (raw)	837.07
DFT (scaled)	786.85
observed band	826

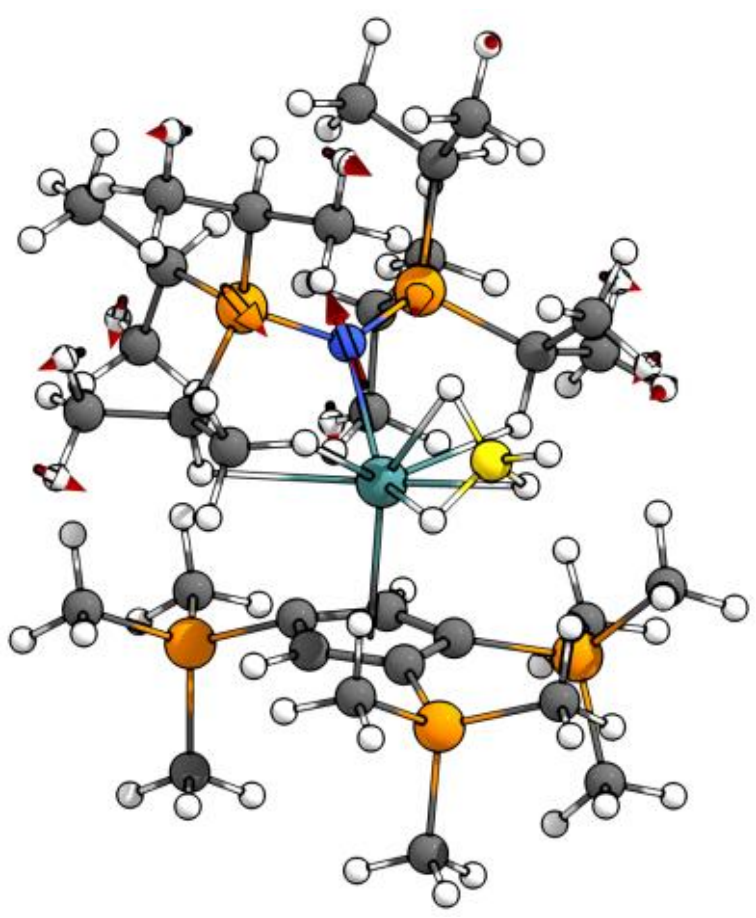

**mode 127**	**E / cm^{-1}**
DFT (raw)	741.88
DFT (scaled)	697.37
observed band	720

4. NMR spectra

4.1 [Dy{N(Si^{i}Pr$_3$)$_2$}(Cp^{ttt})][Al{OC(CF$_3$)$_3$}$_4$] (1-Dy)

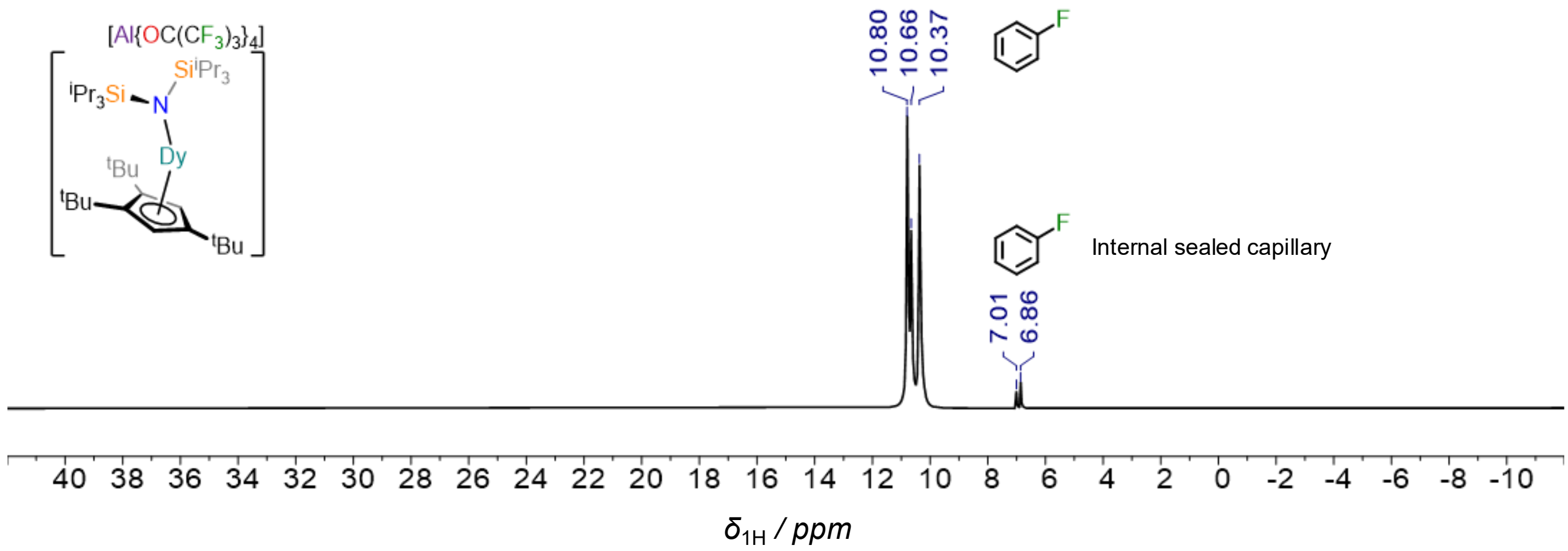

Figure S34. ^{1}H NMR spectrum (500.19 MHz, C_6H_5F) of a sample containing **1-Dy** at 298 K, with an internal sealed capillary (1:1 C_6D_6:C_6H_5F) for reference.

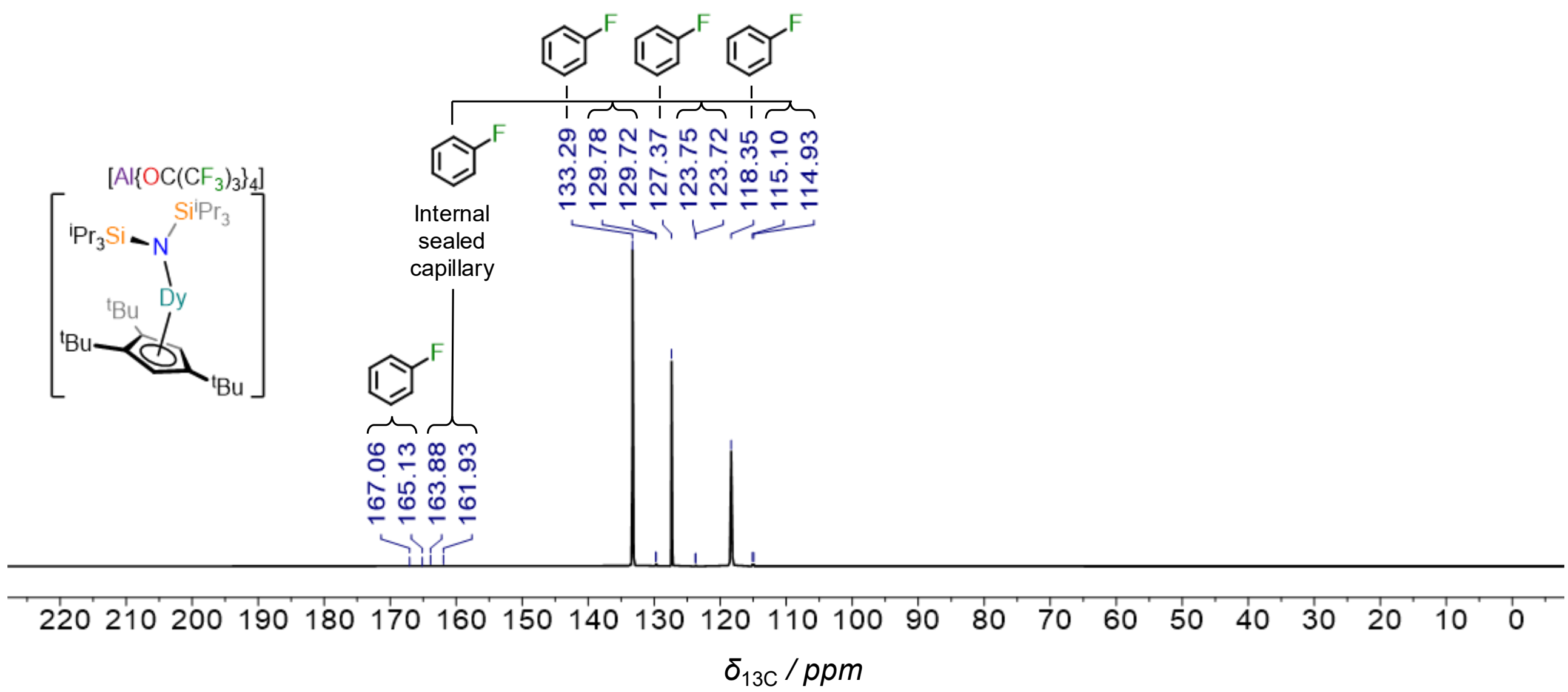

Figure S35. ^{13}C{^{1}H}$_{DEPTQ}$ NMR spectrum (125.79 MHz, C_6H_5F) of a sample containing **1-Dy** at 298 K, with an internal sealed capillary (1:1 C_6D_6:C_6H_5F) for reference.

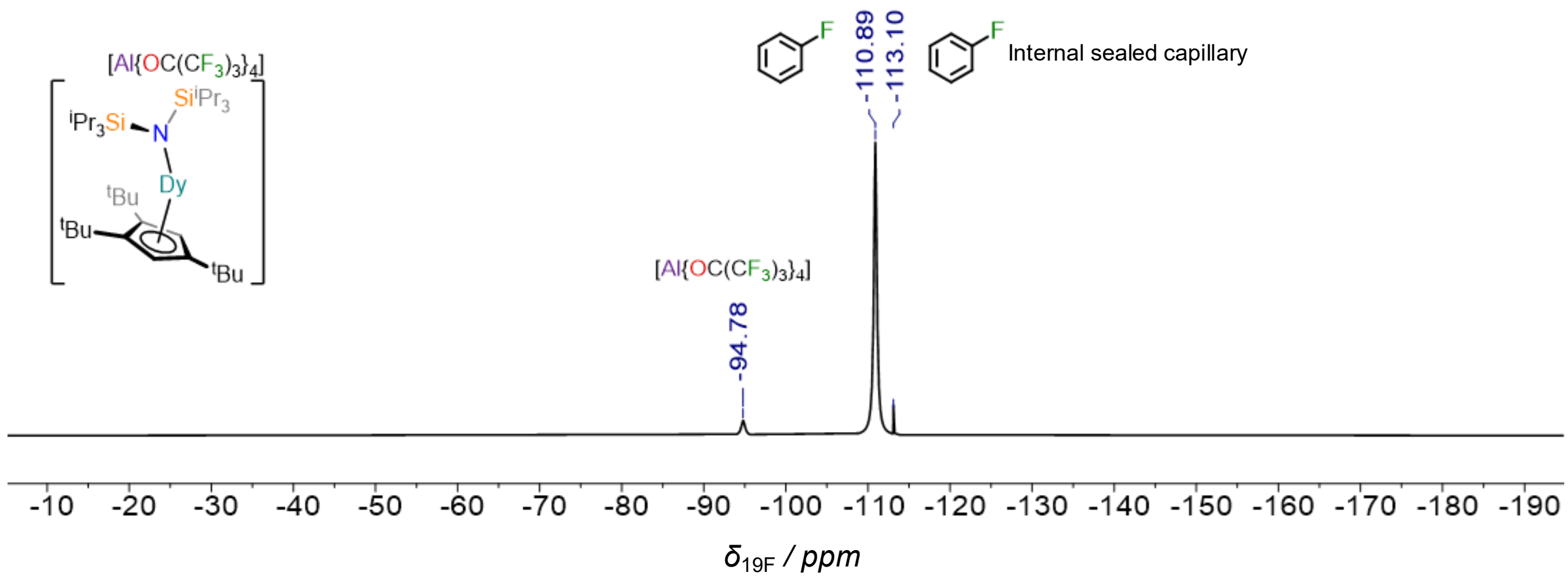

Figure S36. ^{19}F NMR spectrum (470.60 MHz, C_6H_5F) of **1-Dy** at 298 K, with an internal sealed capillary (1:1 C_6D_6:C_6H_5F) for reference.

4.2 [Y{N(Si^{i}Pr$_3$)$_2$}(Cp^{ttt})][Al{OC(CF$_3$)$_3$}$_4$] (1-Y)

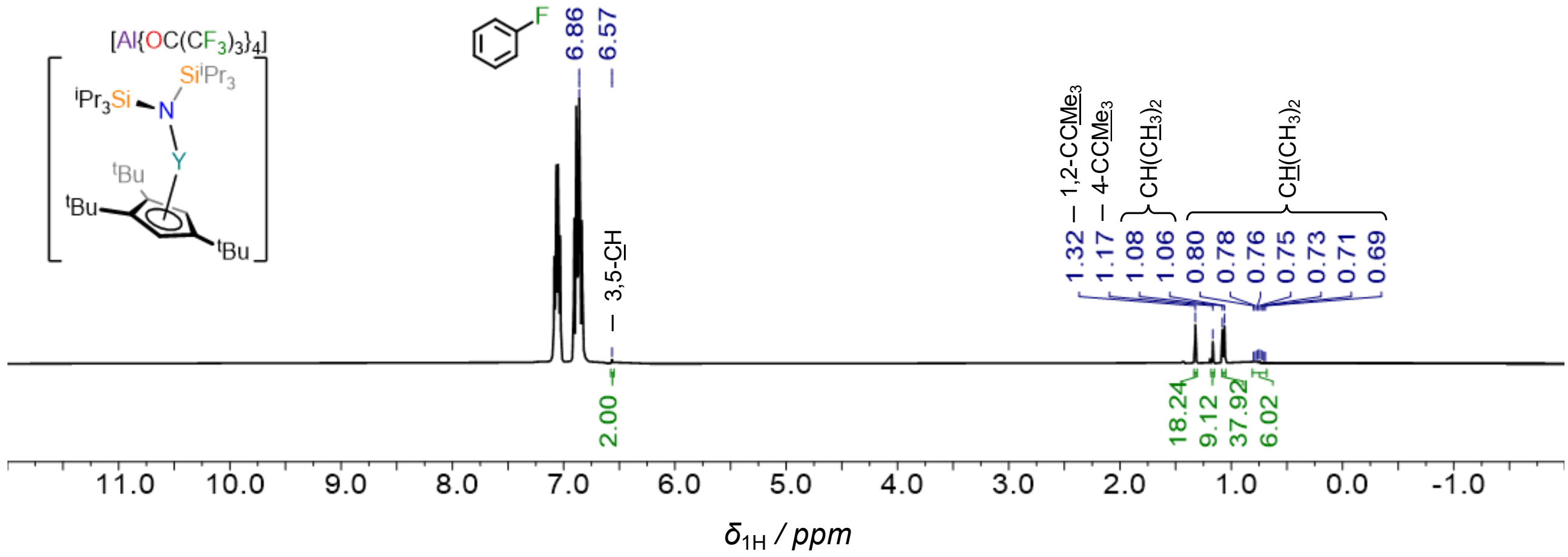

Figure S37. ^{1}H NMR spectrum (400.07 MHz, C_6H_5F) of **1-Y** at 298 K.

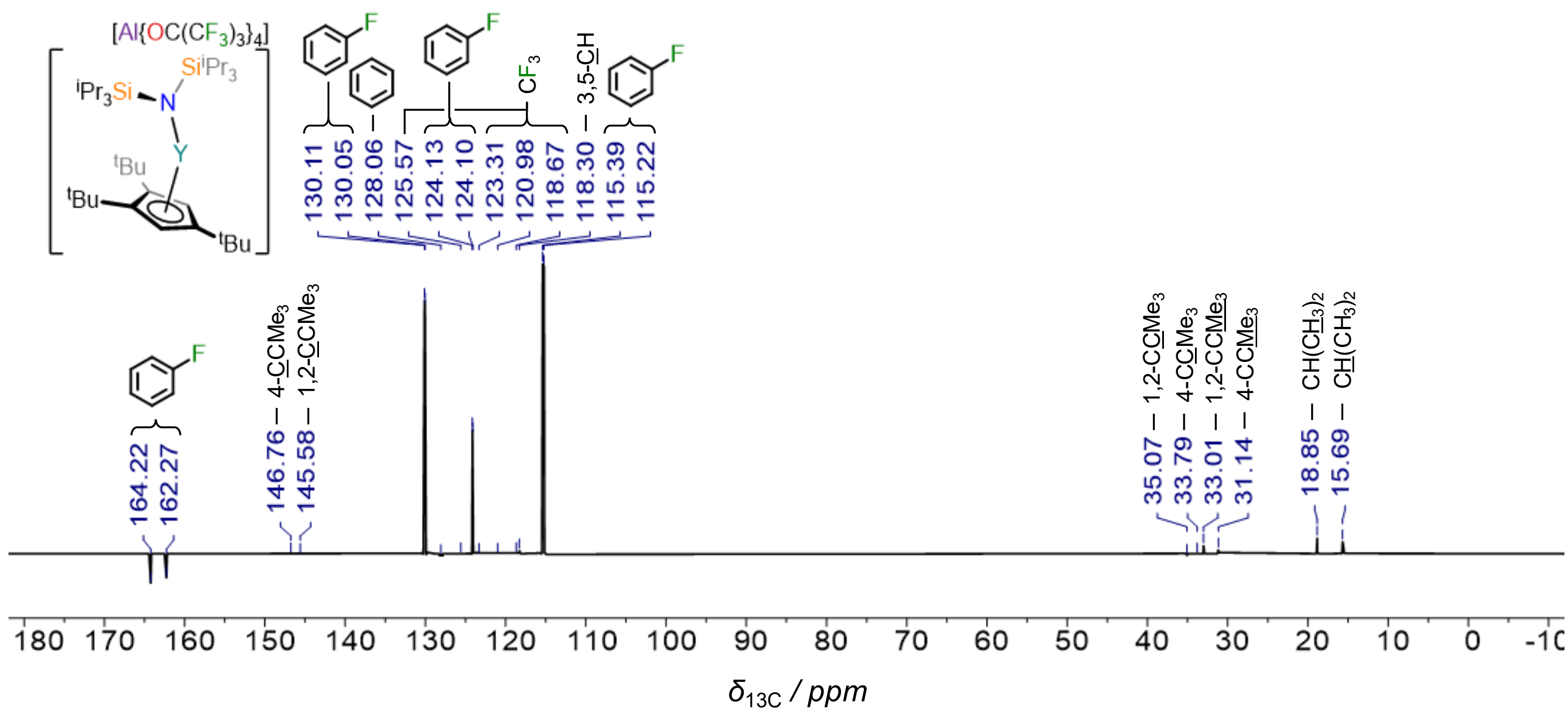

Figure S38. $^{13}C\{^1H\}_{DEPTQ}$ NMR spectrum (100.60 MHz, C_6H_5F) of **1-Y** at 298 K.

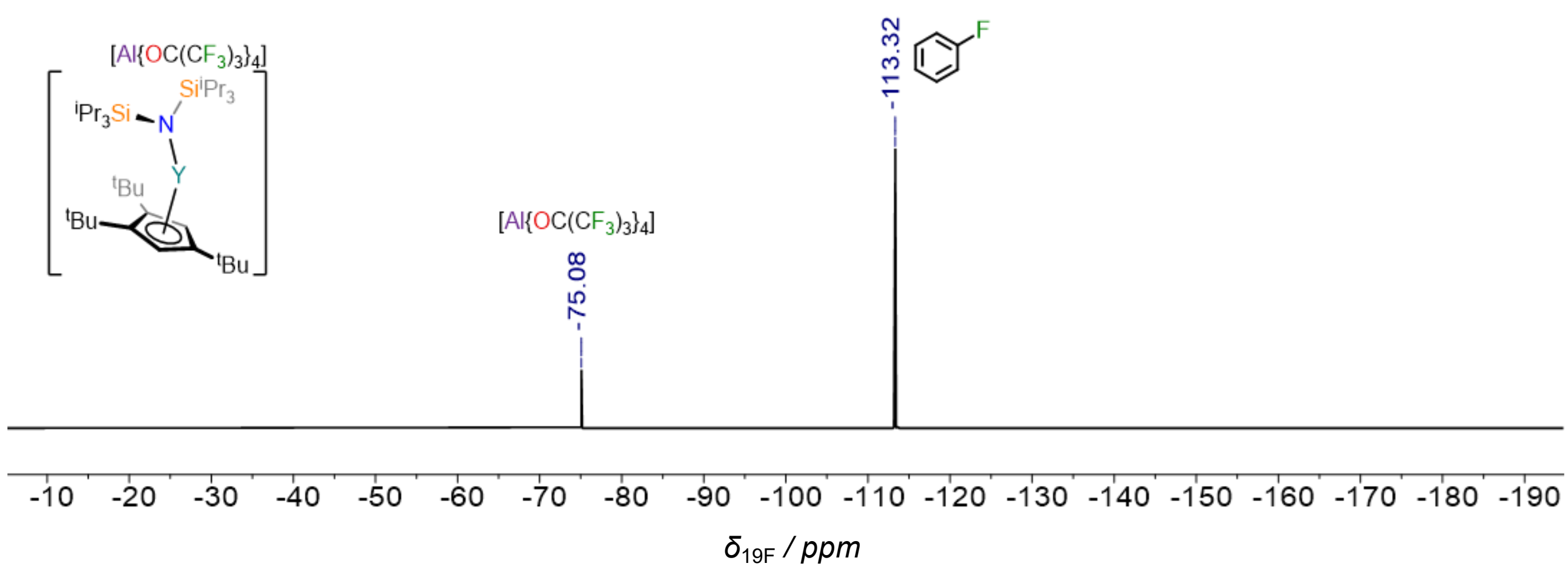

Figure S39. ^{19}F NMR spectrum (376.40 MHz, C_6H_5F) of **1-Y** at 298 K.

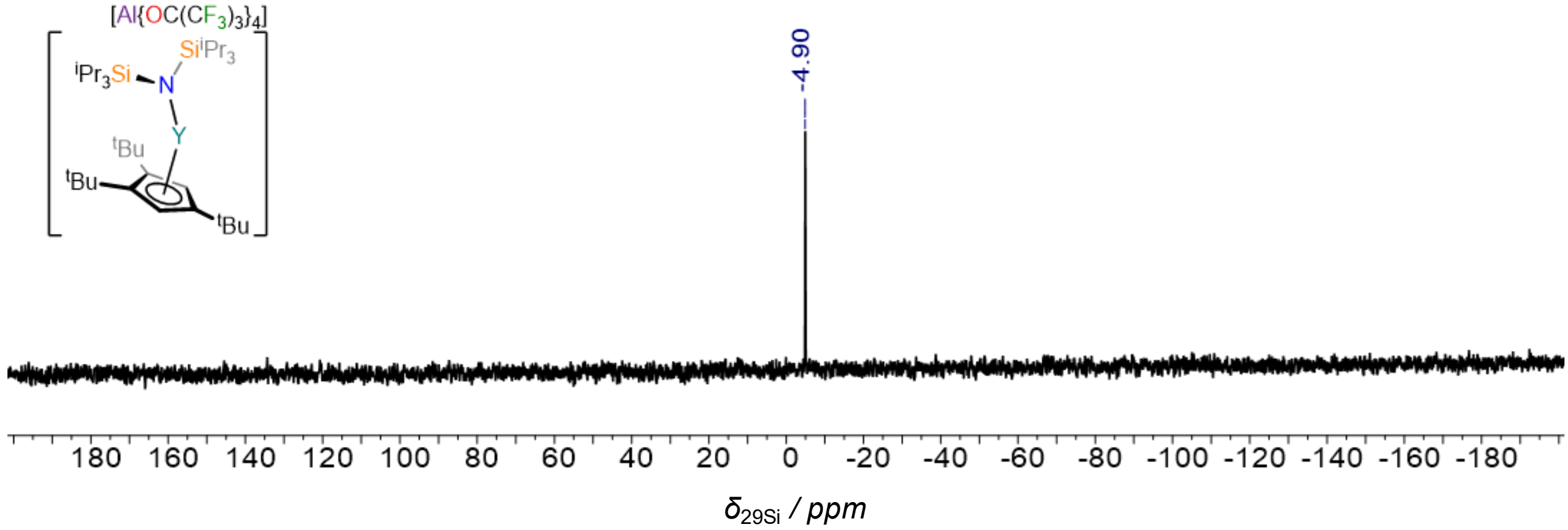

Figure S40. ^{29}Si{^{1}H}$_{\text{DEPT90}}$ NMR spectrum (79.48 MHz, C_6H_5F) of **1-Y** at 298 K.

4.3 [Dy{N(Si^{i}Pr$_3$)$_2$}(Cp′′′)][Al{OC(CF_3)$_3$}$_4$] (2-Dy)

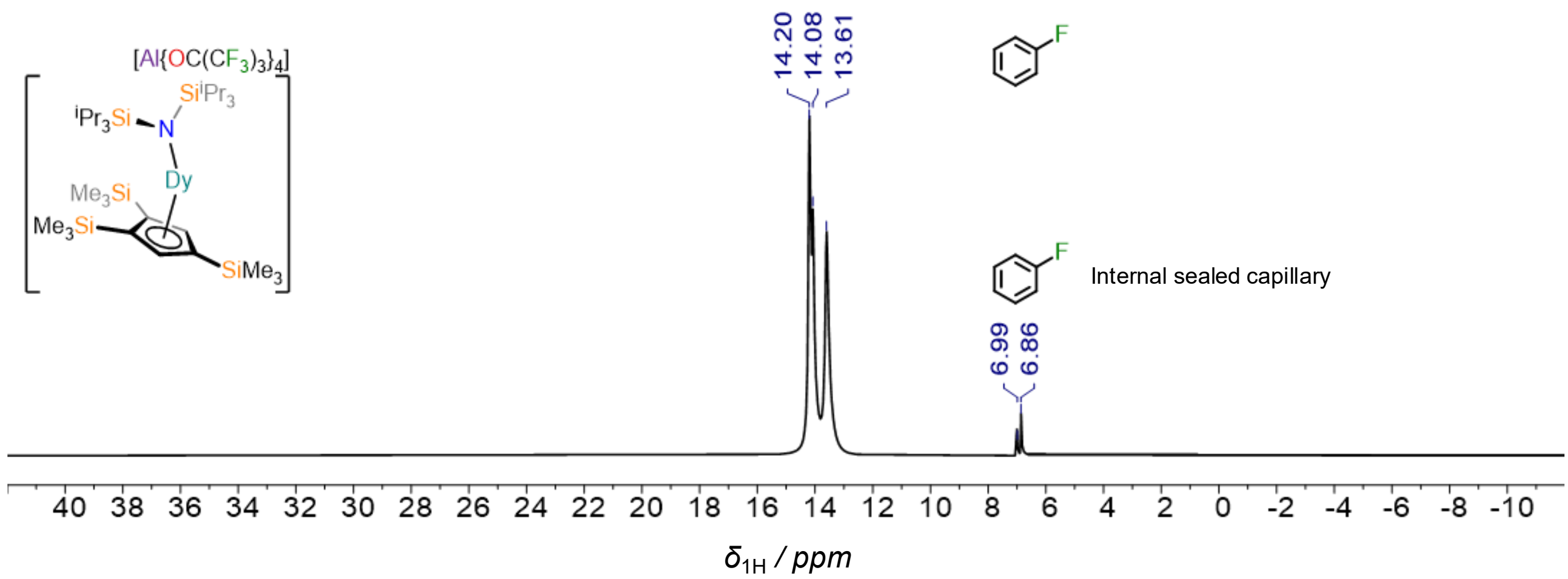

Figure S41. ^{1}H NMR spectrum (500.19 MHz, C_6H_5F) of a sample containing **2-Dy** at 298 K, with an internal sealed capillary (1:1 C_6D_6:C_6H_5F) for reference.

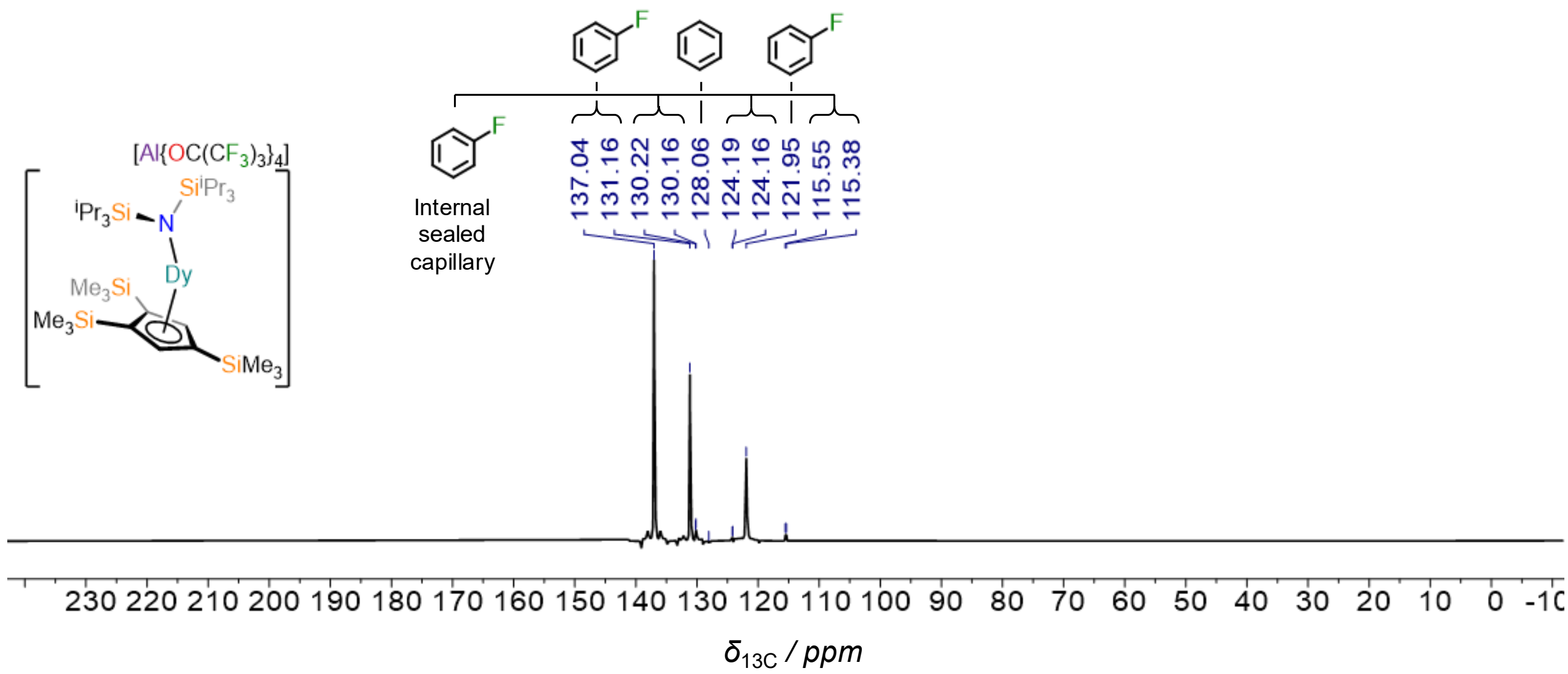

Figure S42. ^{13}C{^{1}H}$_{\text{DEPTQ}}$ NMR spectrum (125.79 MHz, C_6H_5F) of a sample containing **2-Dy** at 298 K, with an internal sealed capillary (1:1 C_6D_6:C_6H_5F) for reference.

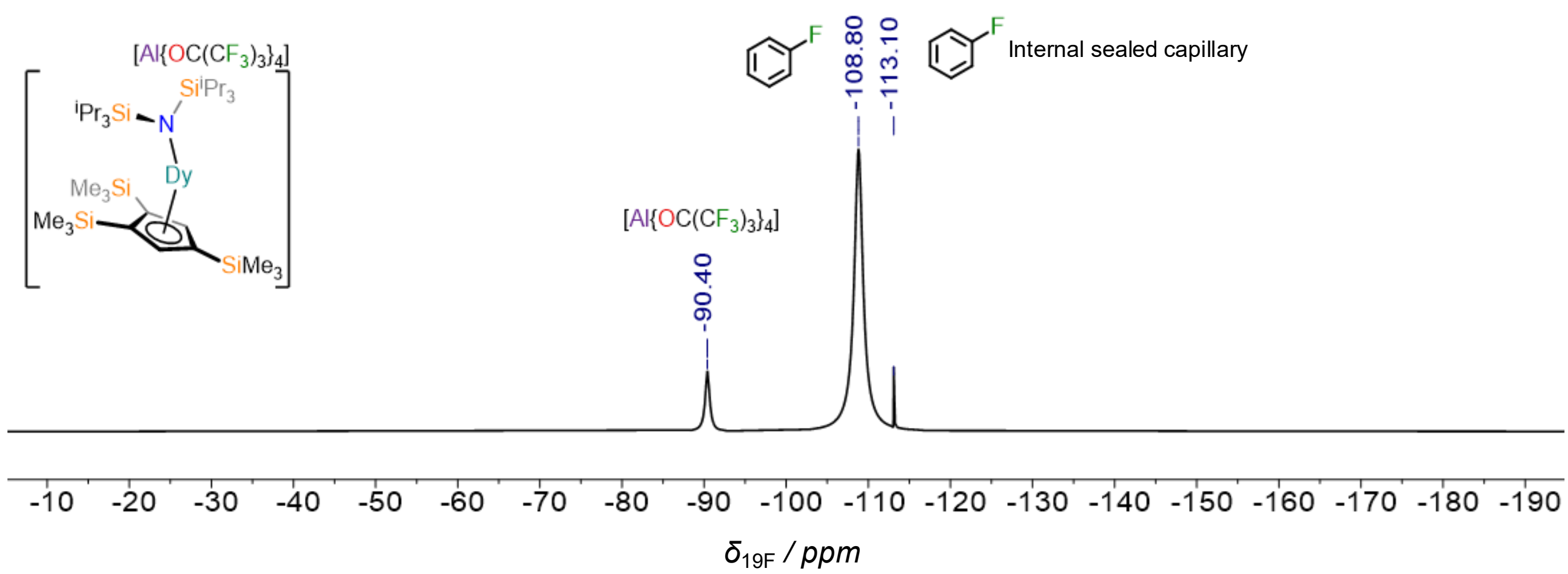

Figure S43. ^{19}F NMR spectrum (470.60 MHz, C_6H_5F) of **2-Dy** at 298 K, with an internal sealed capillary (1:1 C_6D_6:C_6H_5F) for reference.

4.4 [Y{N(Si^{i}Pr$_3$)$_2$}(Cp′′′)][Al{OC(CF$_3$)$_3$}$_4$] (2-Y)

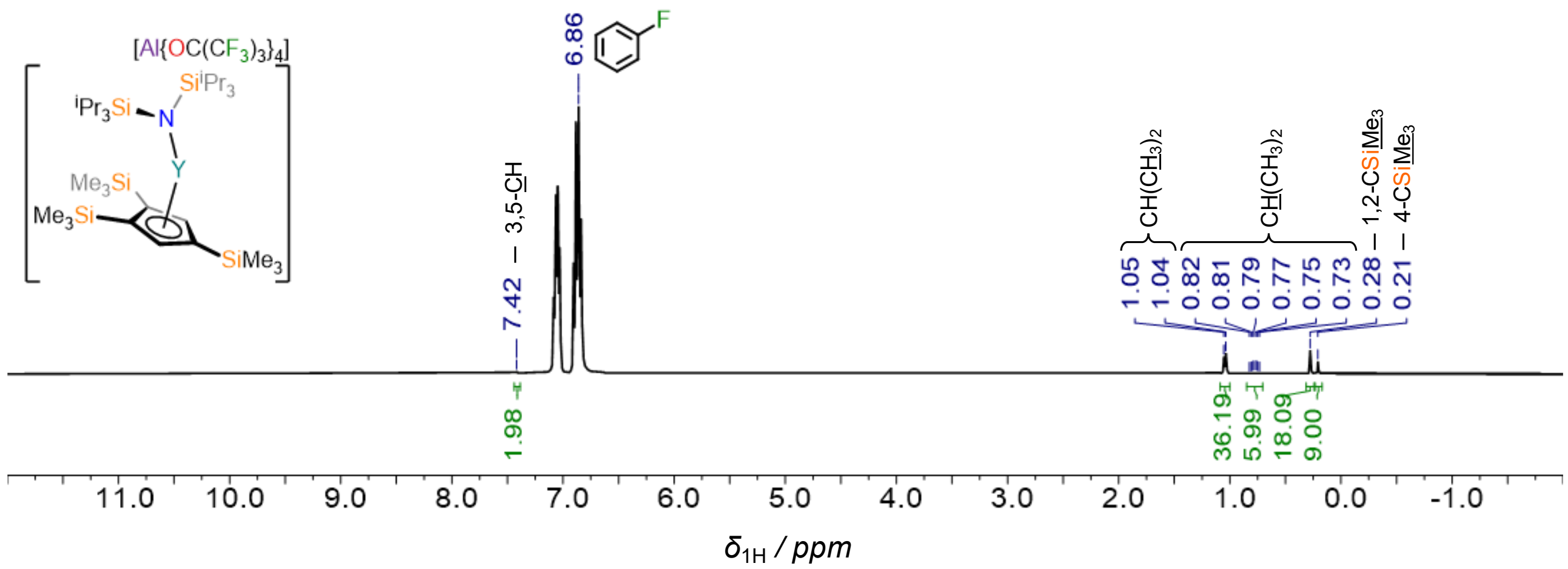

Figure S44. ^{1}H NMR spectrum (400.07 MHz, C_6H_5F) of **2-Y** at 298 K.

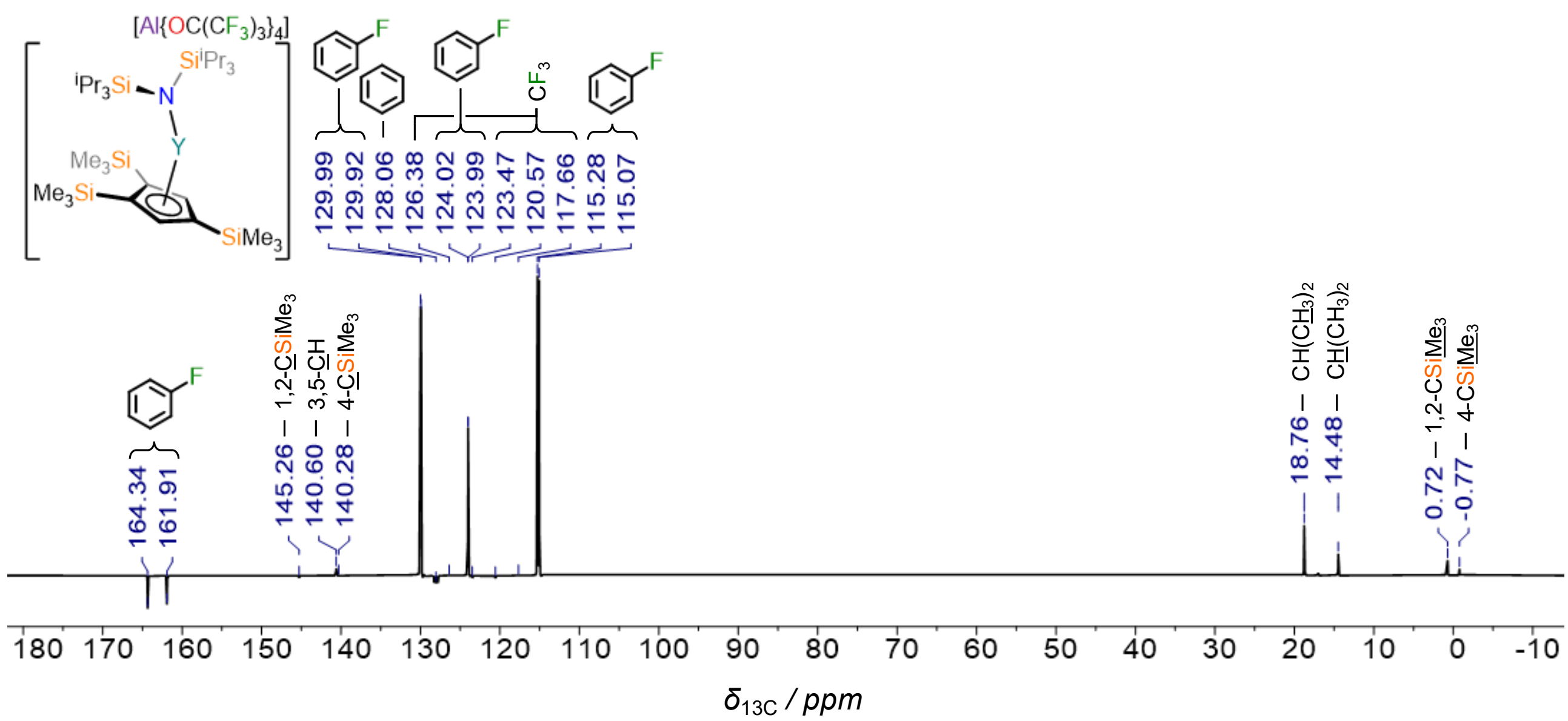

Figure S45. ^{13}C{^{1}H}$_{DEPTQ}$ NMR spectrum (100.60 MHz, C_6H_5F) of **2-Y** at 298 K.

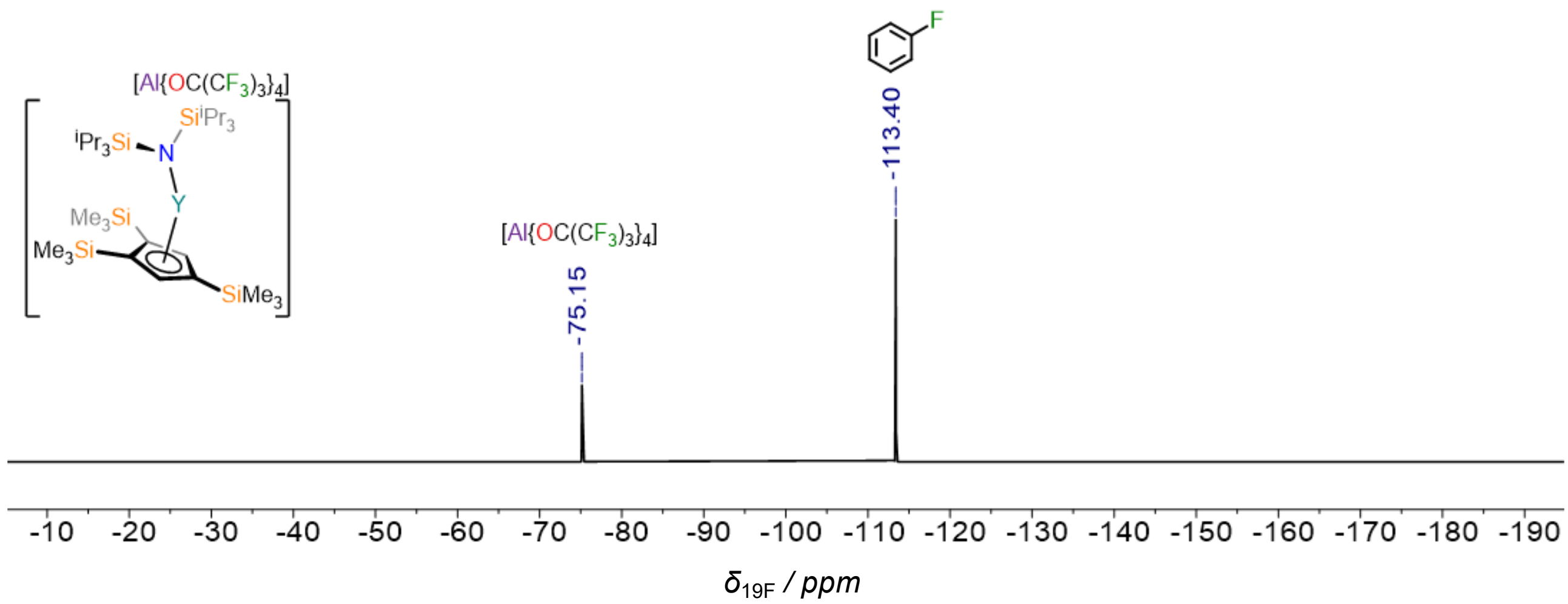

Figure S46. ^{19}F NMR spectrum (376.40 MHz, C_6H_5F) of **2-Y** at 298 K.

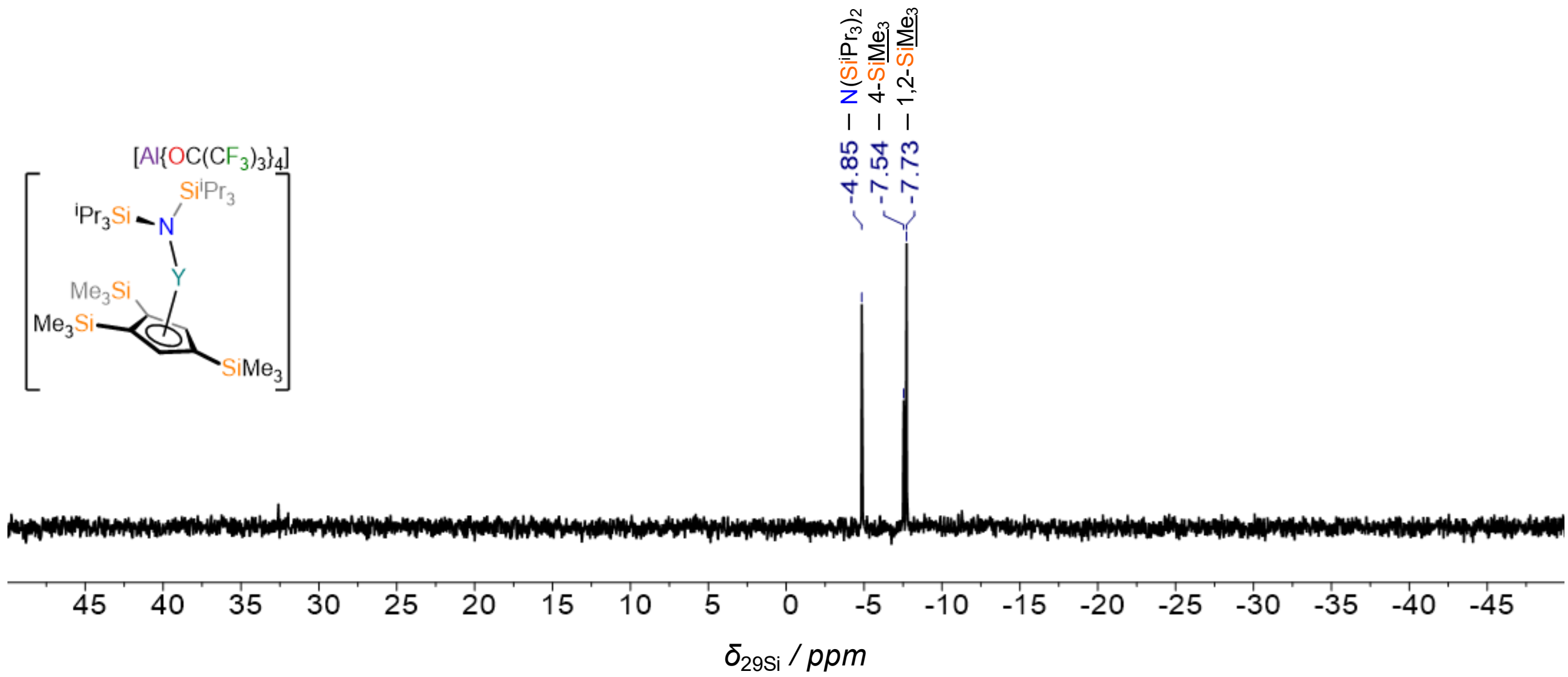

Figure S47. ^{29}Si{^{1}H}$_{DEPT90}$ NMR spectrum (79.48 MHz, C_6H_5F) of **2-Y** at 298 K.

4.5 [Dy{N(Si^{i}Pr$_3$)$_2$}(Cp^{ttt})(BH$_4$)] (3-Dy)

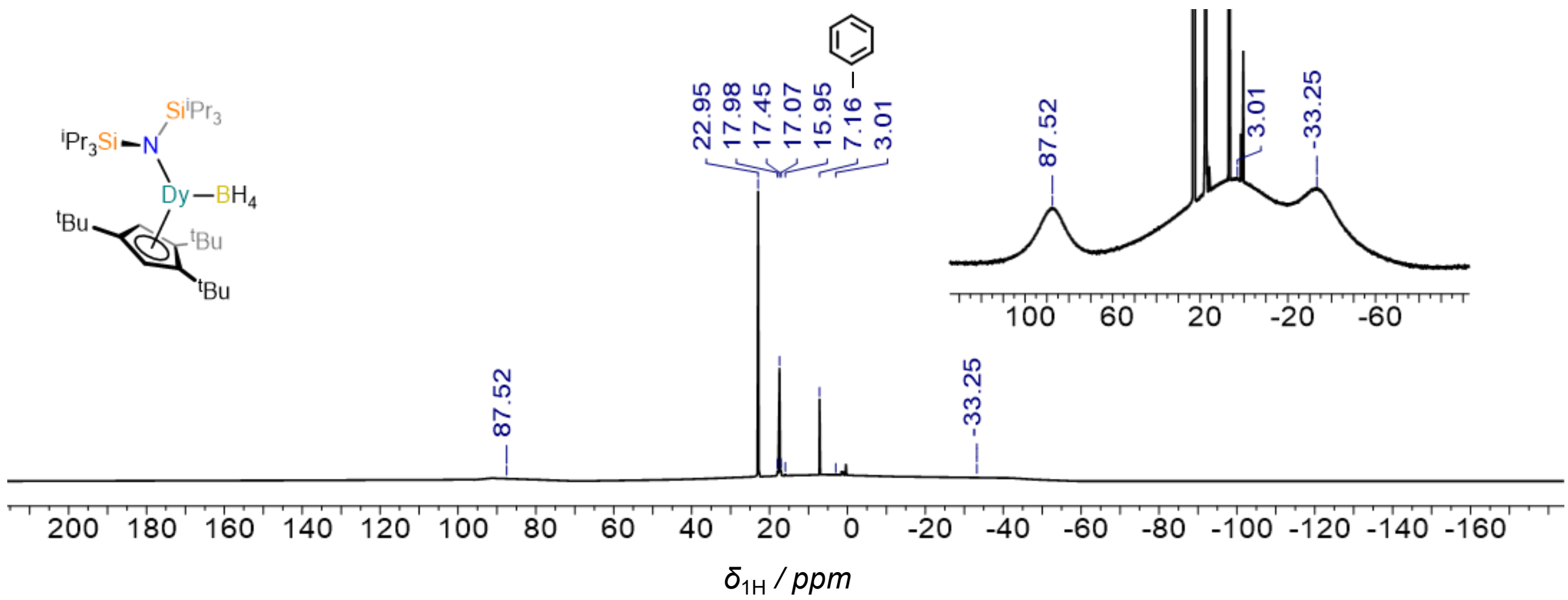

Figure S48. ^{1}H NMR spectrum (400.07 MHz, C_6D_6) of **3-Dy** at 298 K.

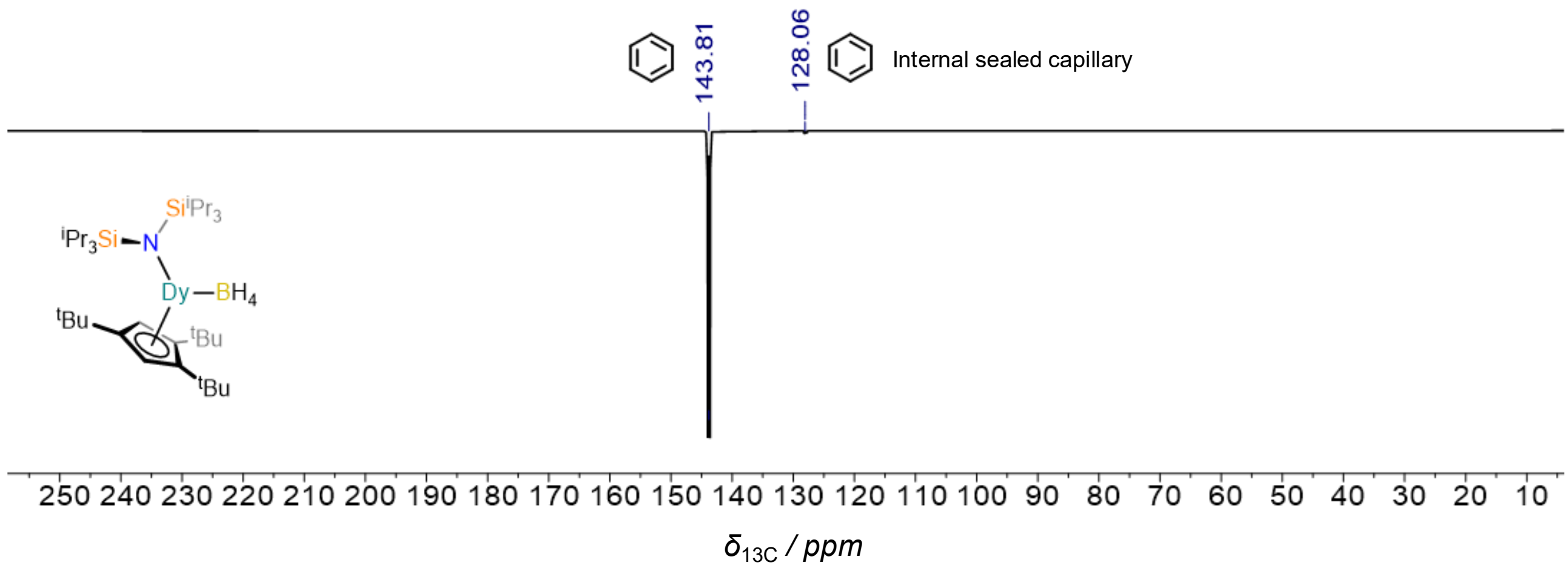

Figure S49. $^{13}C\{^1H\}_{DEPTQ}$ NMR spectrum (100.60 MHz, C_6D_6)) of a sample containing **3-Dy** at 298 K, with an internal sealed capillary (C_6D_6) for reference.

4.6 [Y{N(Si^{i}Pr$_3$)$_2$}(Cp^{ttt})(BH$_4$)] (3-Y)

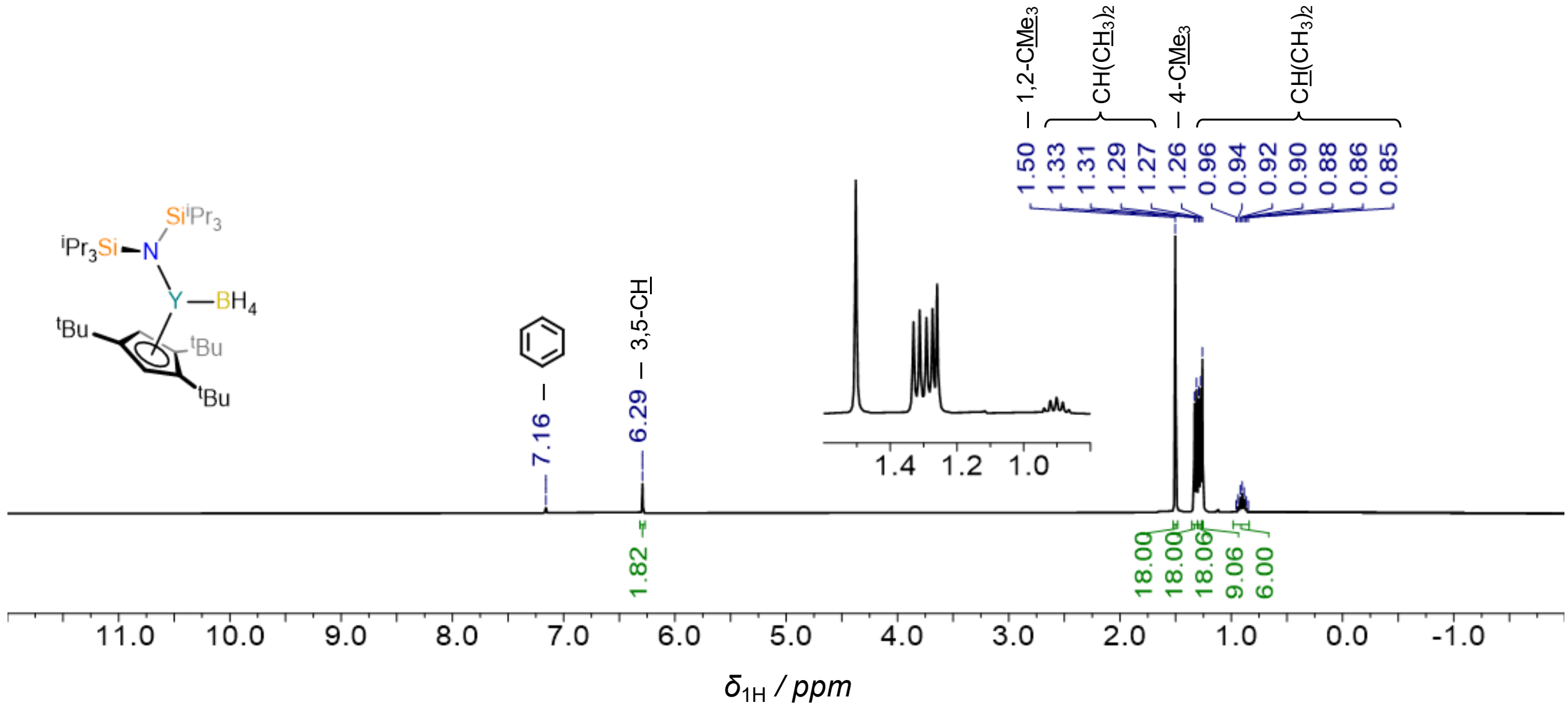

Figure S50. ^1H NMR spectrum (400.07 MHz, C_6D_6) of **3-Y** at 298 K.

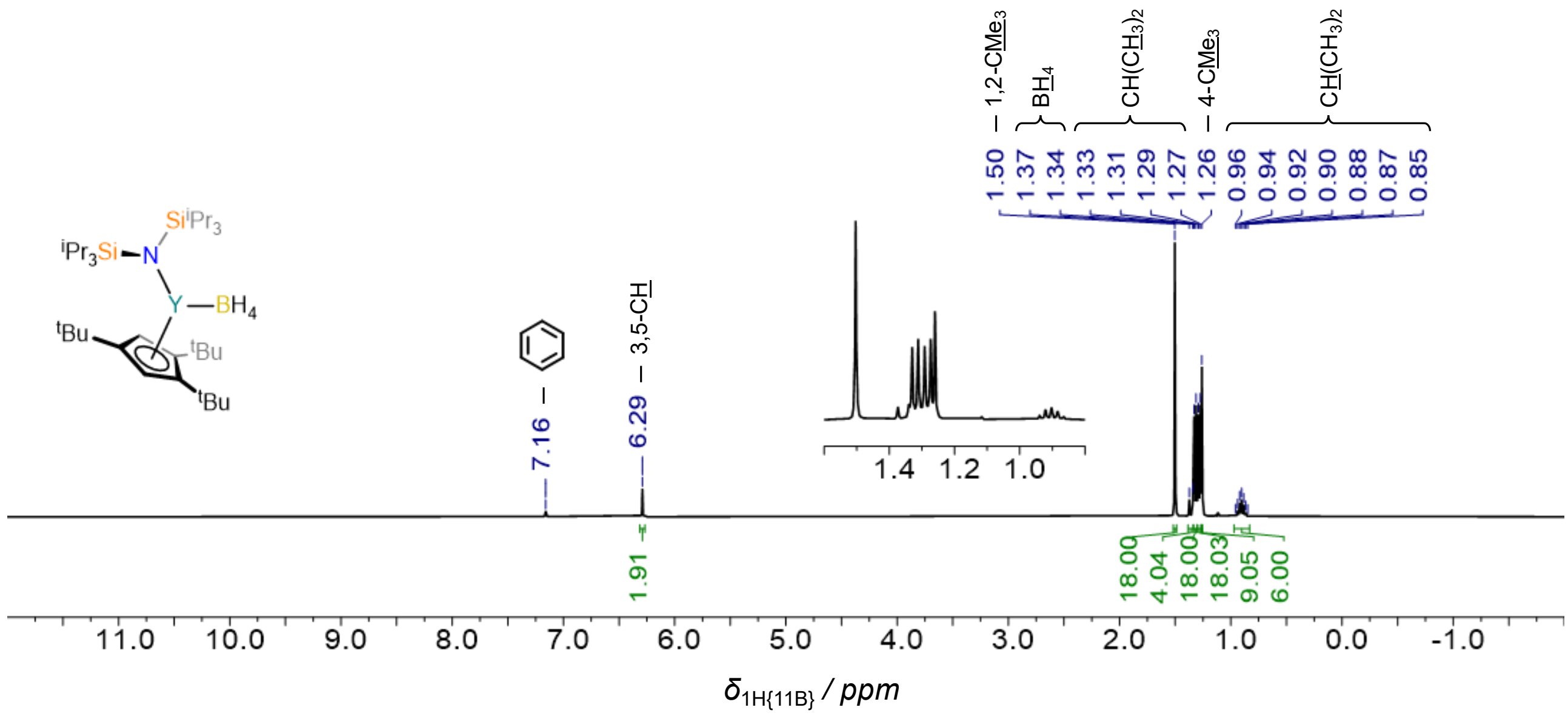

Figure S51. $^1H\{^{11}B\}$ NMR spectrum (400.07 MHz, C_6D_6) of **3-Y** at 298 K.

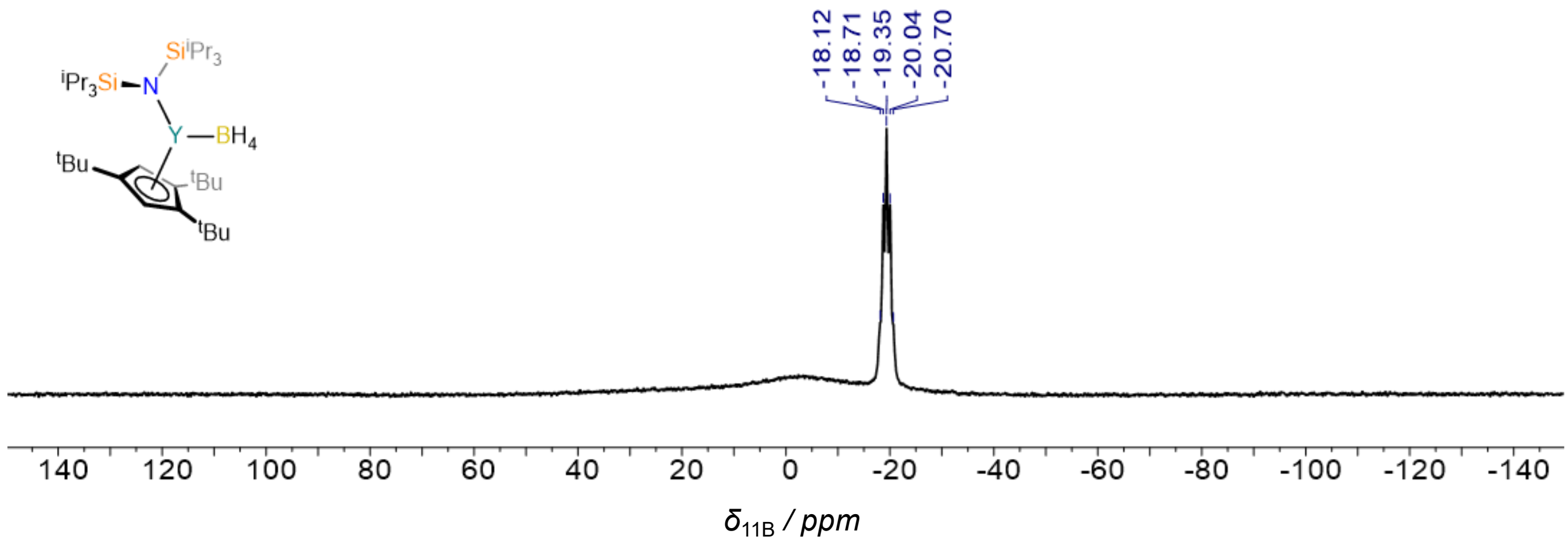

Figure S52. ^{11}B NMR spectrum (128.36 MHz, C_6D_6) of **3-Y** at 298 K.

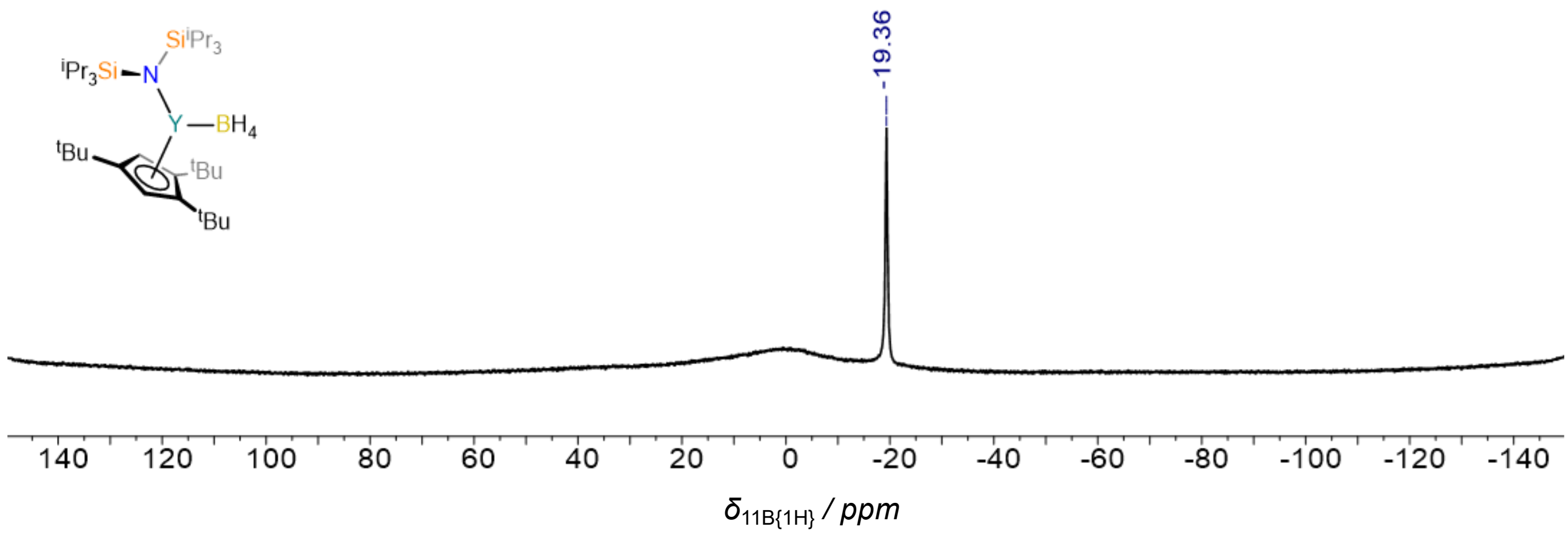

Figure S53. $^{11}B\{^1H\}$ NMR spectrum (128.36 MHz, C_6D_6) of **3-Y** at 298 K.

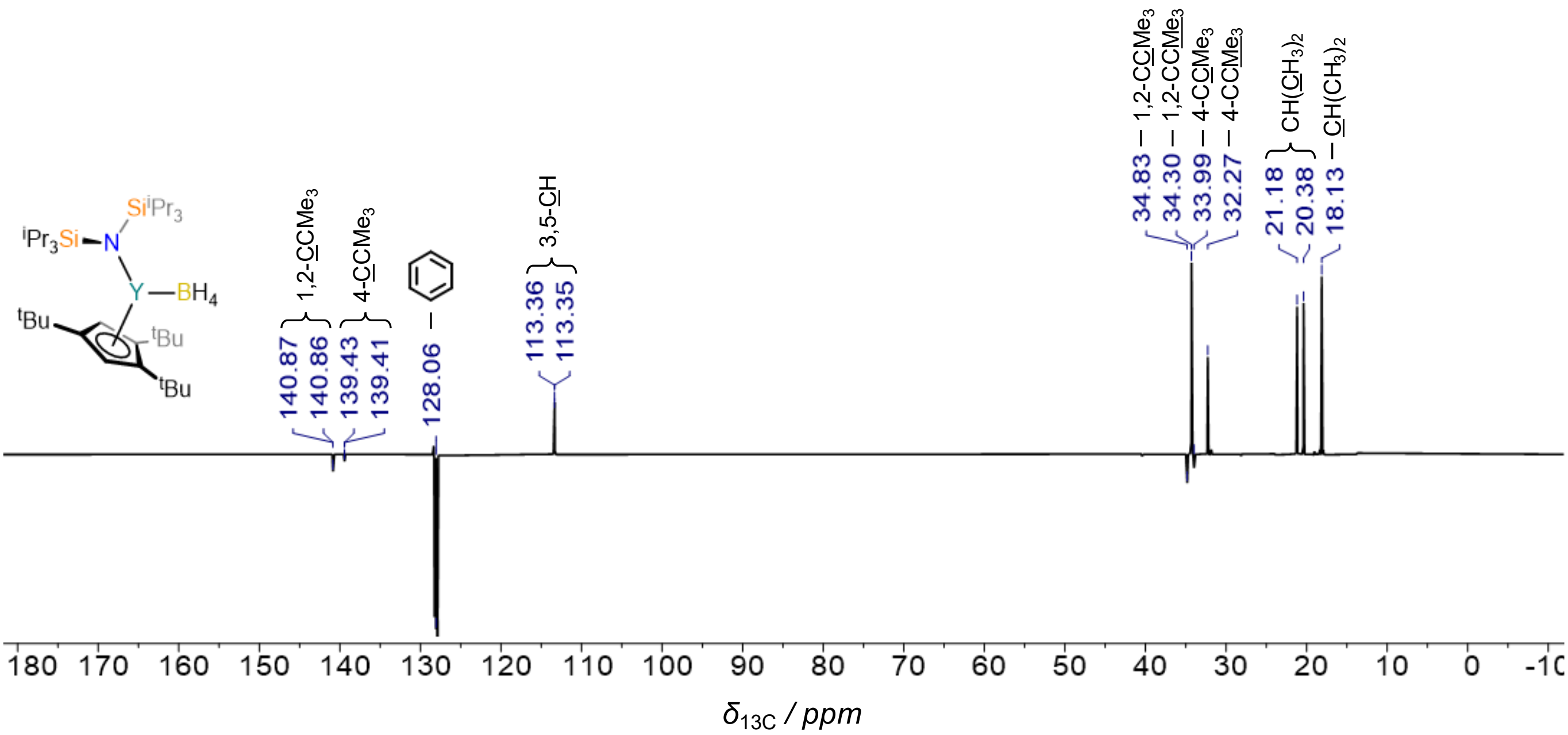

Figure S54. $^{13}C\{^1H\}_{DEPTQ}$ NMR spectrum (100.60 MHz, C_6D_6) of **3-Y** at 298 K.

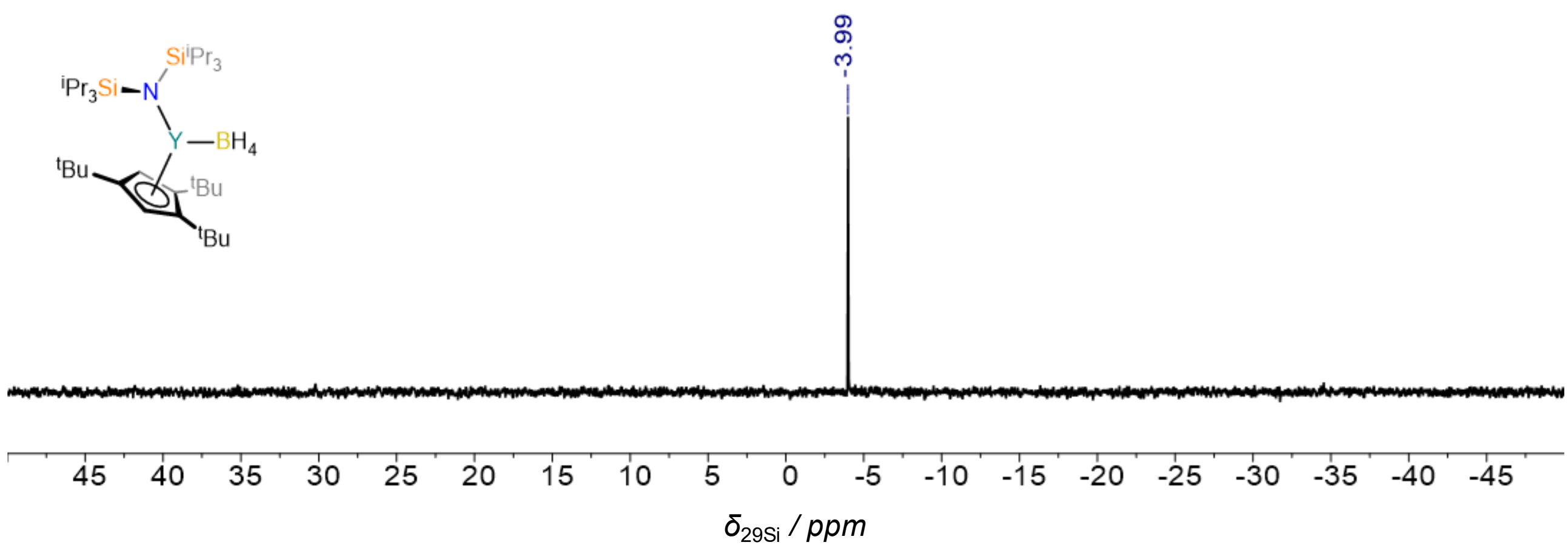

Figure S55. $^{29}Si\{^{1}H\}_{DEPT90}$ NMR spectrum (79.48 MHz, C_6D_6) of **3-Y** at 298 K.

4.7 [Dy{N(Si^{i}Pr$_3$)$_2$}(Cp′′′)(BH$_4$)] (4-Dy)

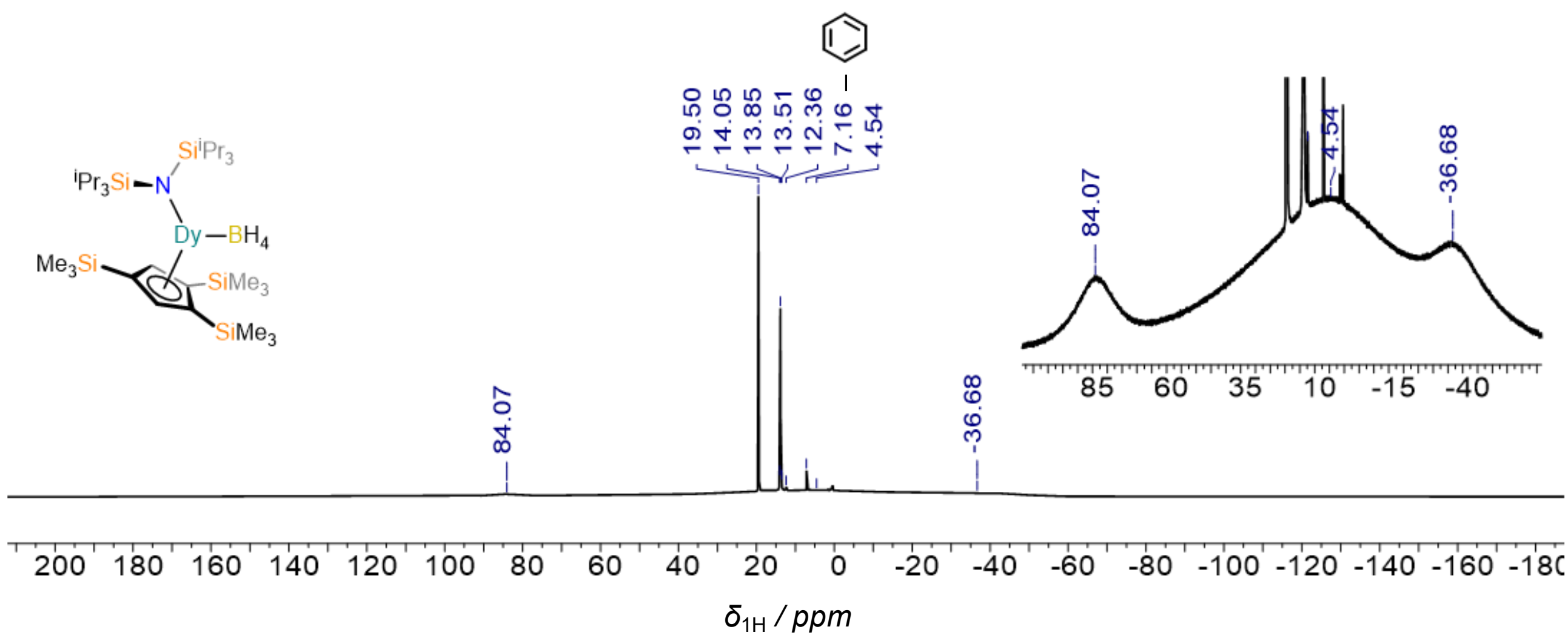

Figure S56. ^{1}H NMR spectrum (400.07 MHz, C_6D_6) of **4-Dy** at 298 K.

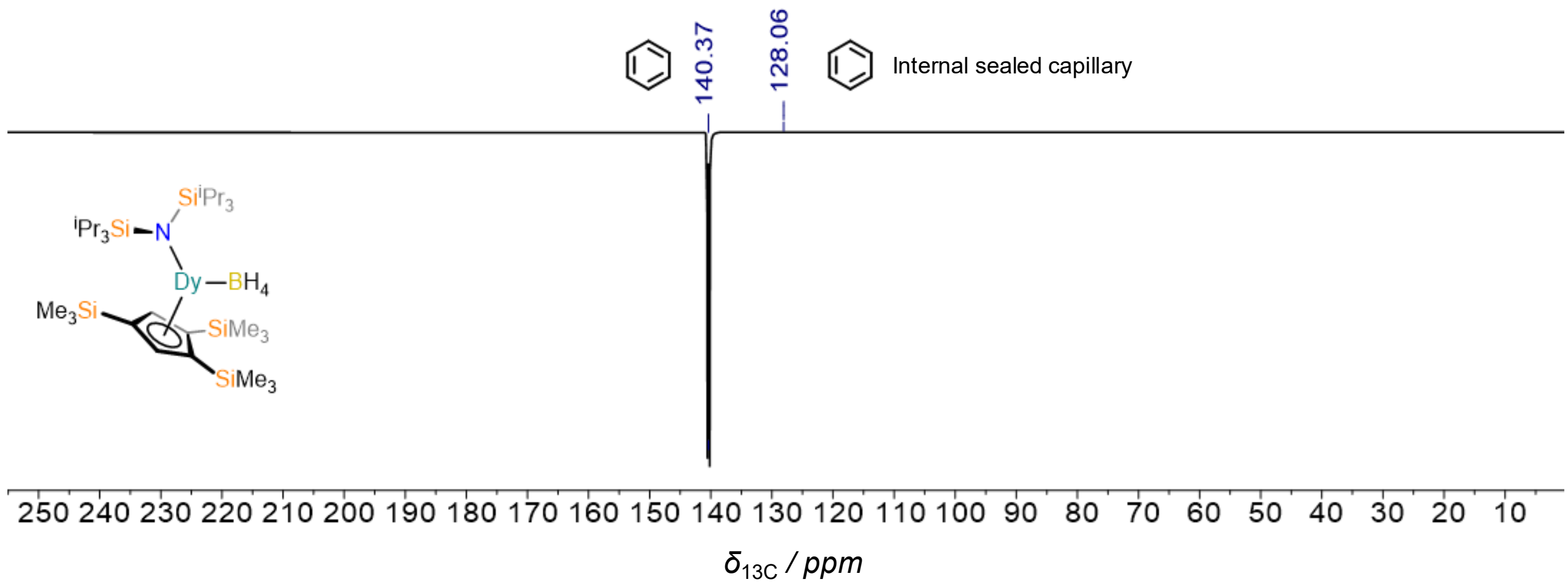

Figure S57. $^{13}C\{^{1}H\}_{DEPTQ}$ NMR spectrum (100.60 MHz, C_6D_6) of a sample containing **4-Dy** at 298 K, with an internal sealed capillary (C_6D_6) for reference.

4.8 [Y{N(Si^{i}Pr$_3$)$_2$}(Cp′′′)(BH$_4$)] (4-Y)

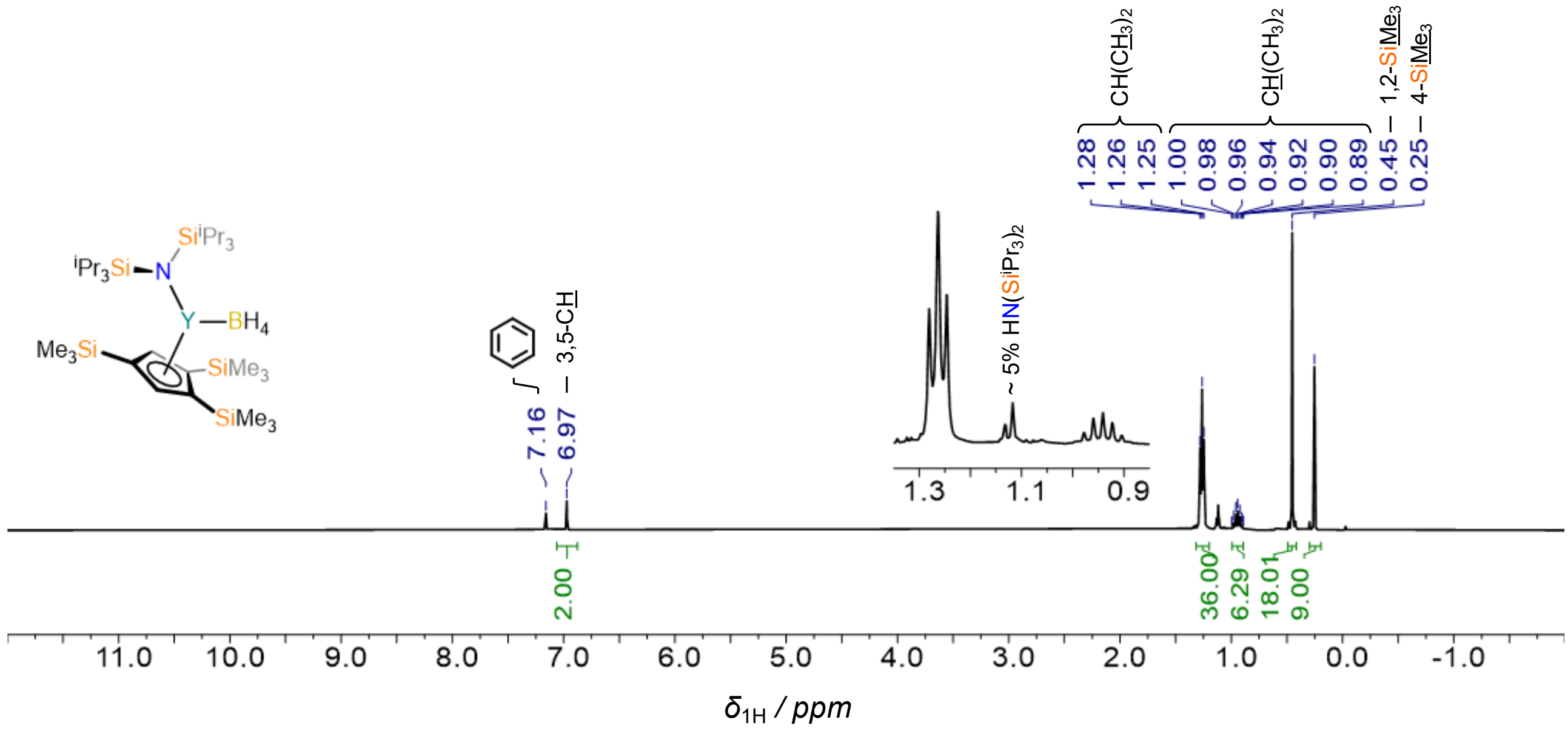

Figure S58. ^{1}H NMR spectrum (400.07 MHz, C_6D_6) of **4-Y** at 298 K.

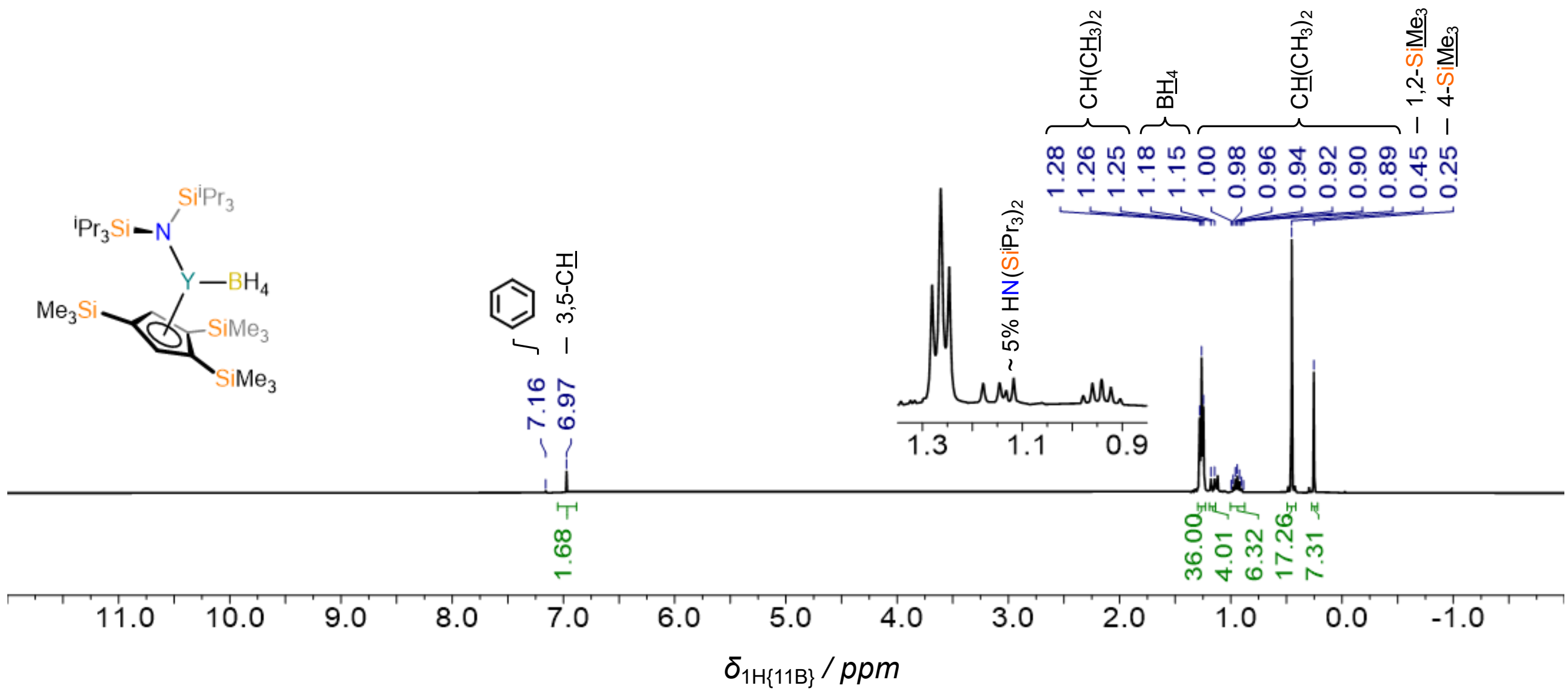

Figure S59. $^{1}H\{^{11}B\}$ NMR spectrum (400.07 MHz, C_6D_6) of **4-Y** at 298 K.

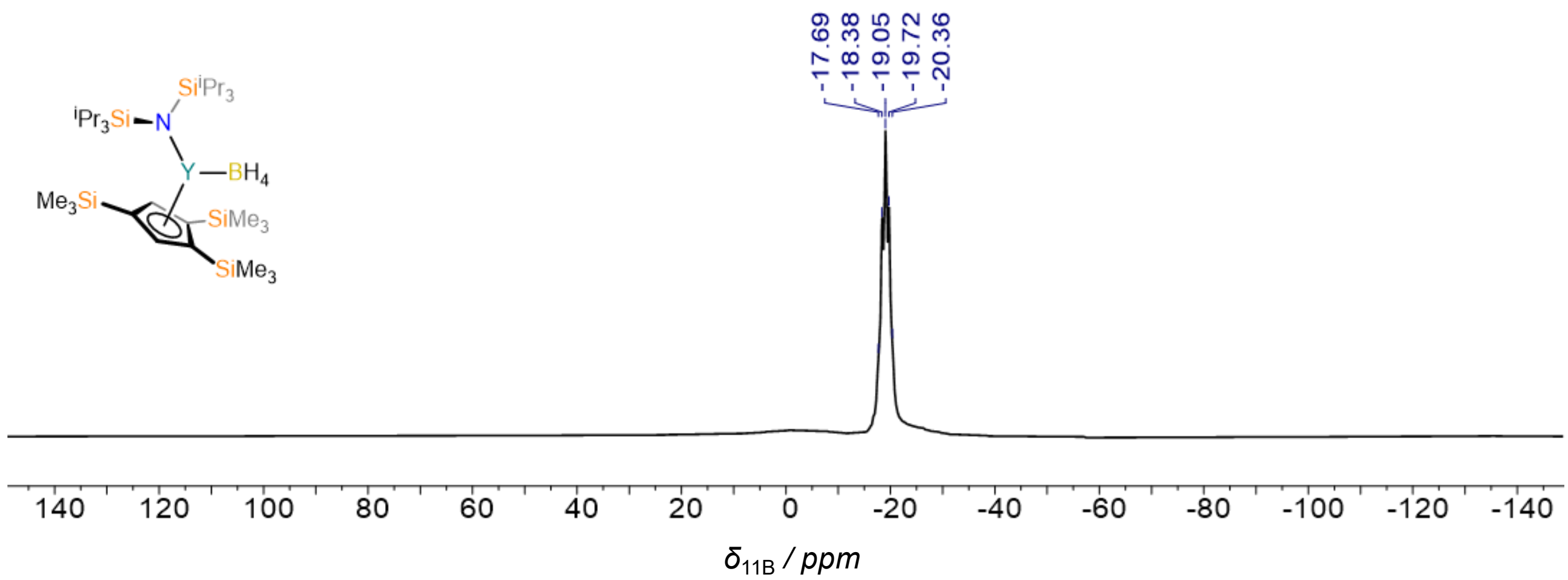

Figure S60. ^{11}B NMR spectrum (128.36 MHz, C_6D_6) of **4-Y** at 298 K.

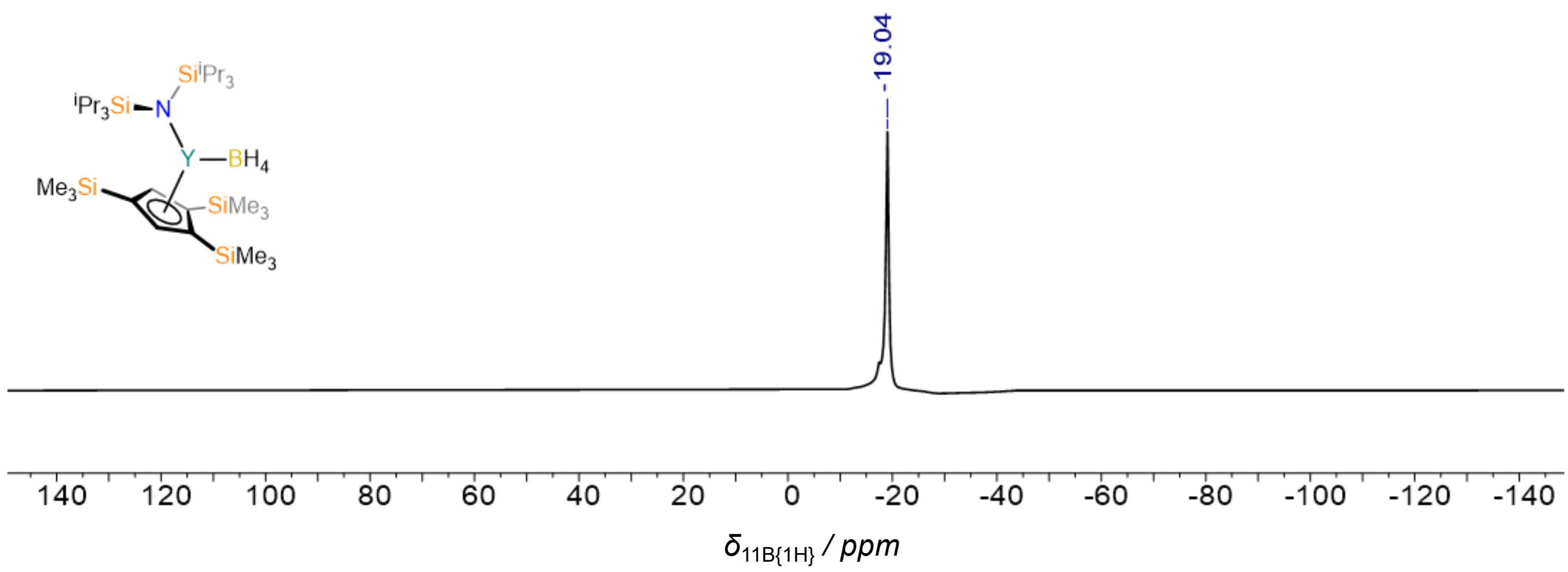

Figure S61. $^{11}B\{^1H\}$ NMR spectrum (128.36 MHz, C_6D_6) of **4-Y** at 298 K.

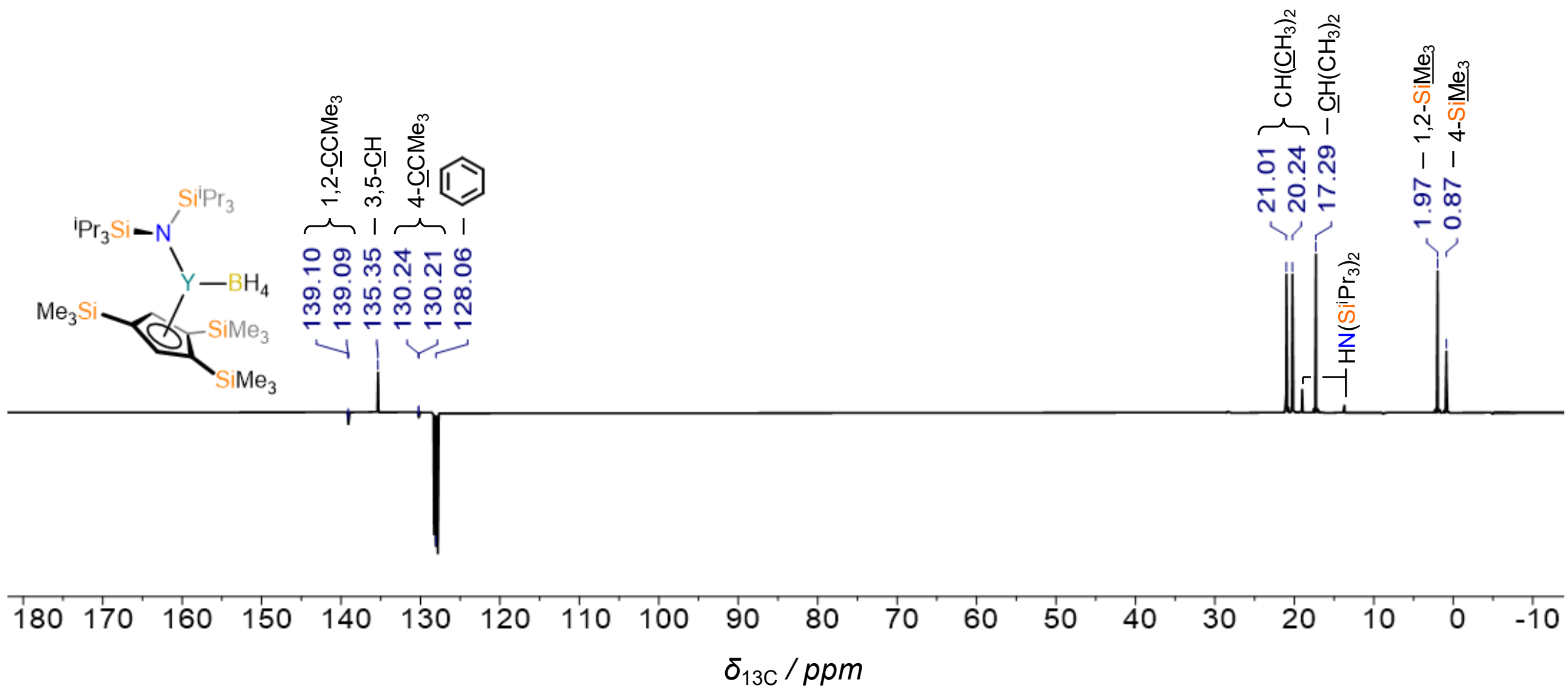

Figure S62. $^{13}C\{^1H\}_{DEPTQ}$ NMR spectrum (100.60 MHz, C_6D_6) of **4-Y** at 298 K.

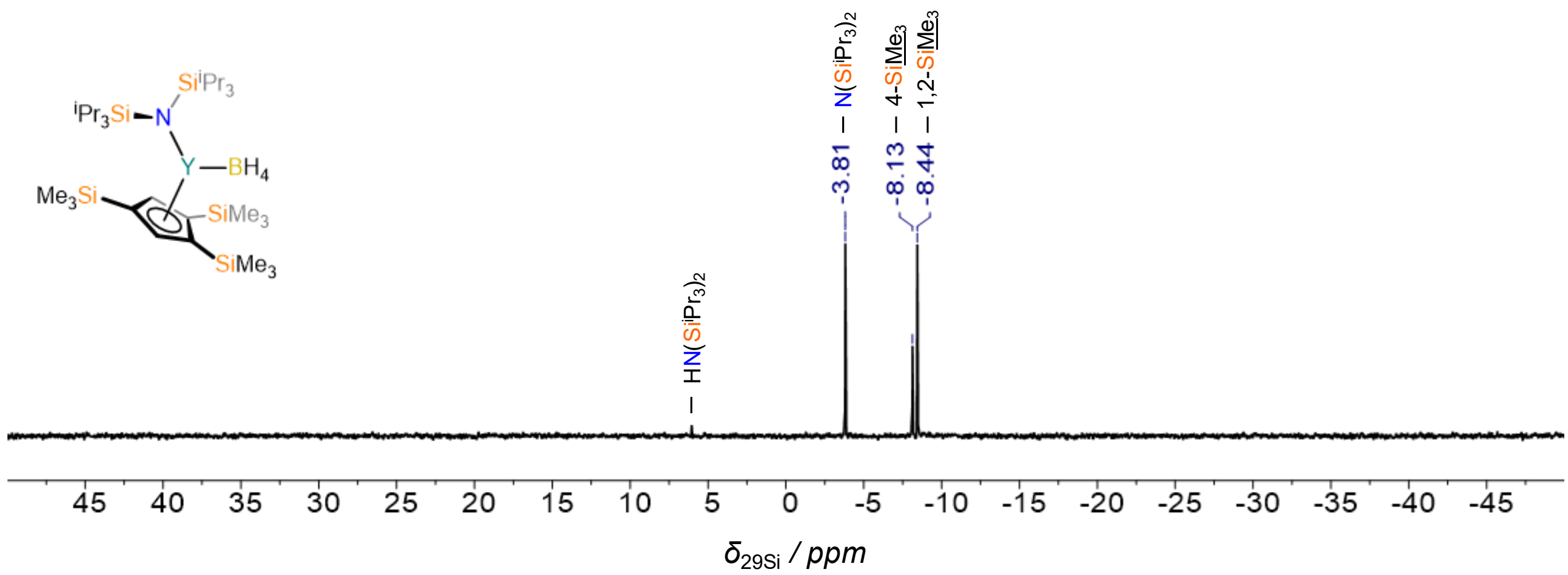

Figure S63. $^{29}Si\{^1H\}_{DEPT90}$ NMR spectrum (79.48 MHz, C_6D_6) of **4-Y** at 298 K.

5. Magnetism data

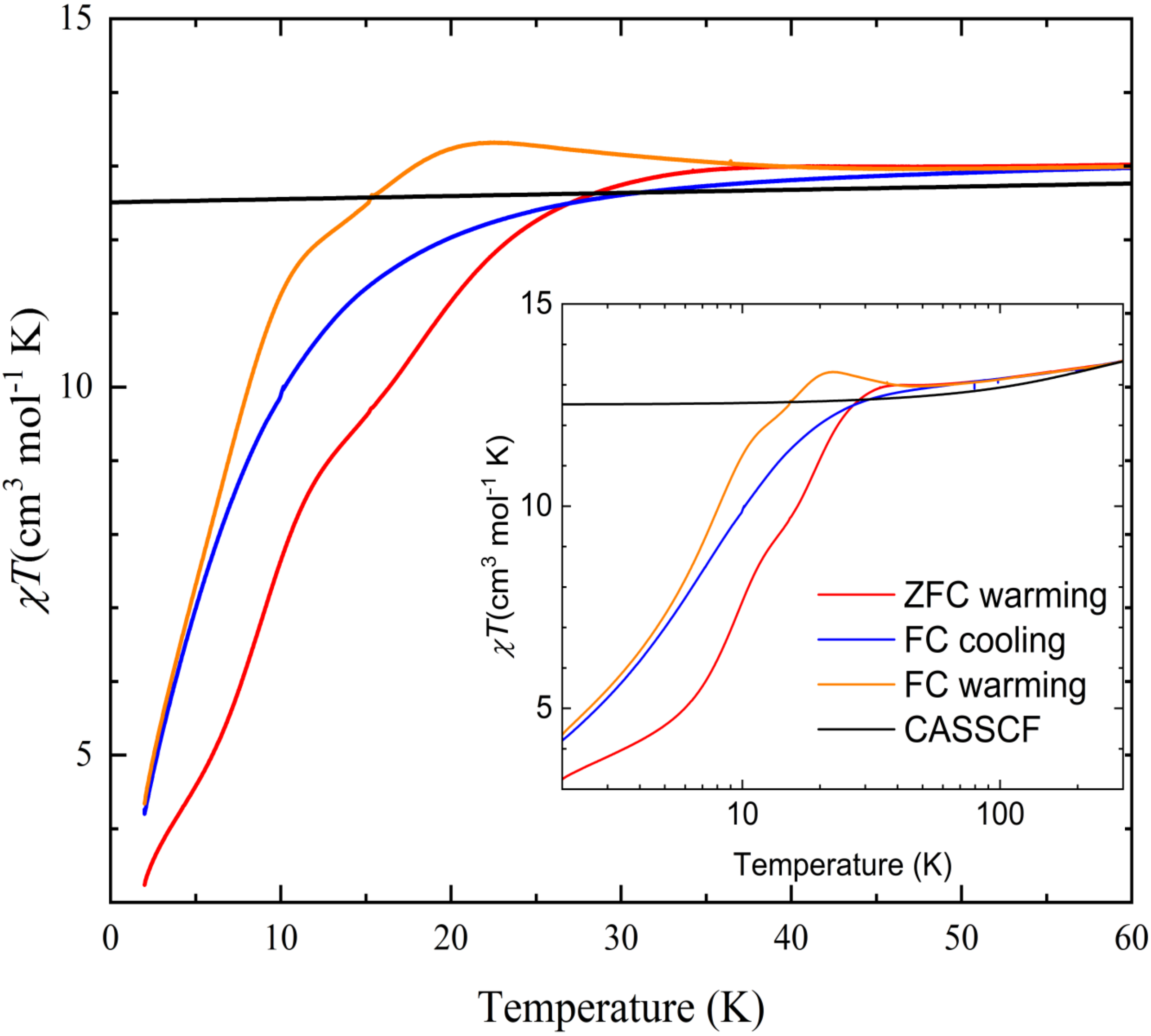

Figure S64. Molar magnetic susceptibility product (χ*T*) vs. temperature (*T*) for **1-Dy** performed at 0.1 T, showing ZFC and FC curves and CASSCF-calculated traces from 2–60 K on a linear scale and (inset) 2–300 K on a logarithmic scale.

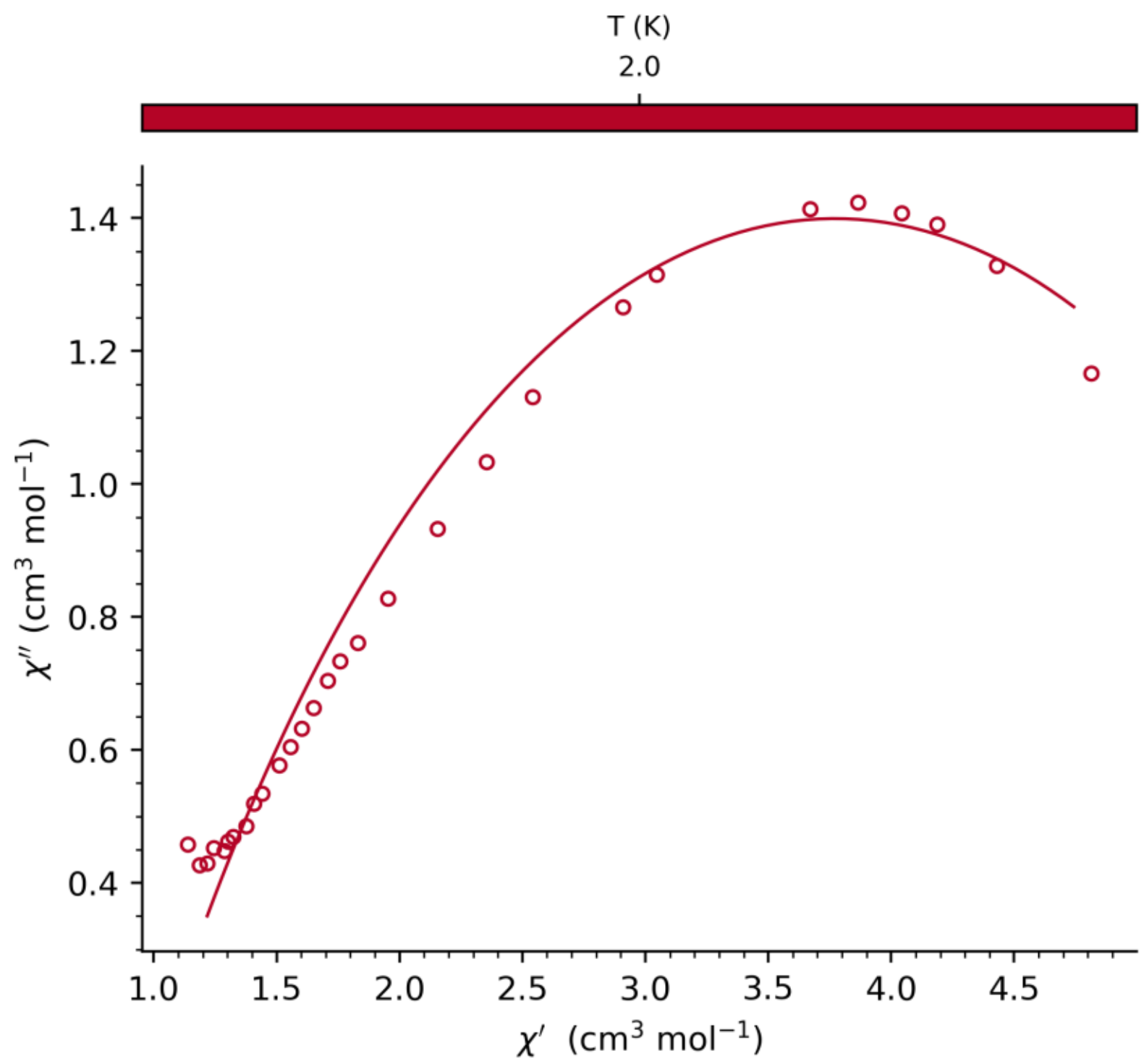

Figure S65. Cole-Cole plot of waveform susceptibility data of **1-Dy** performed at 2 K fitted to a generalised Debye model.

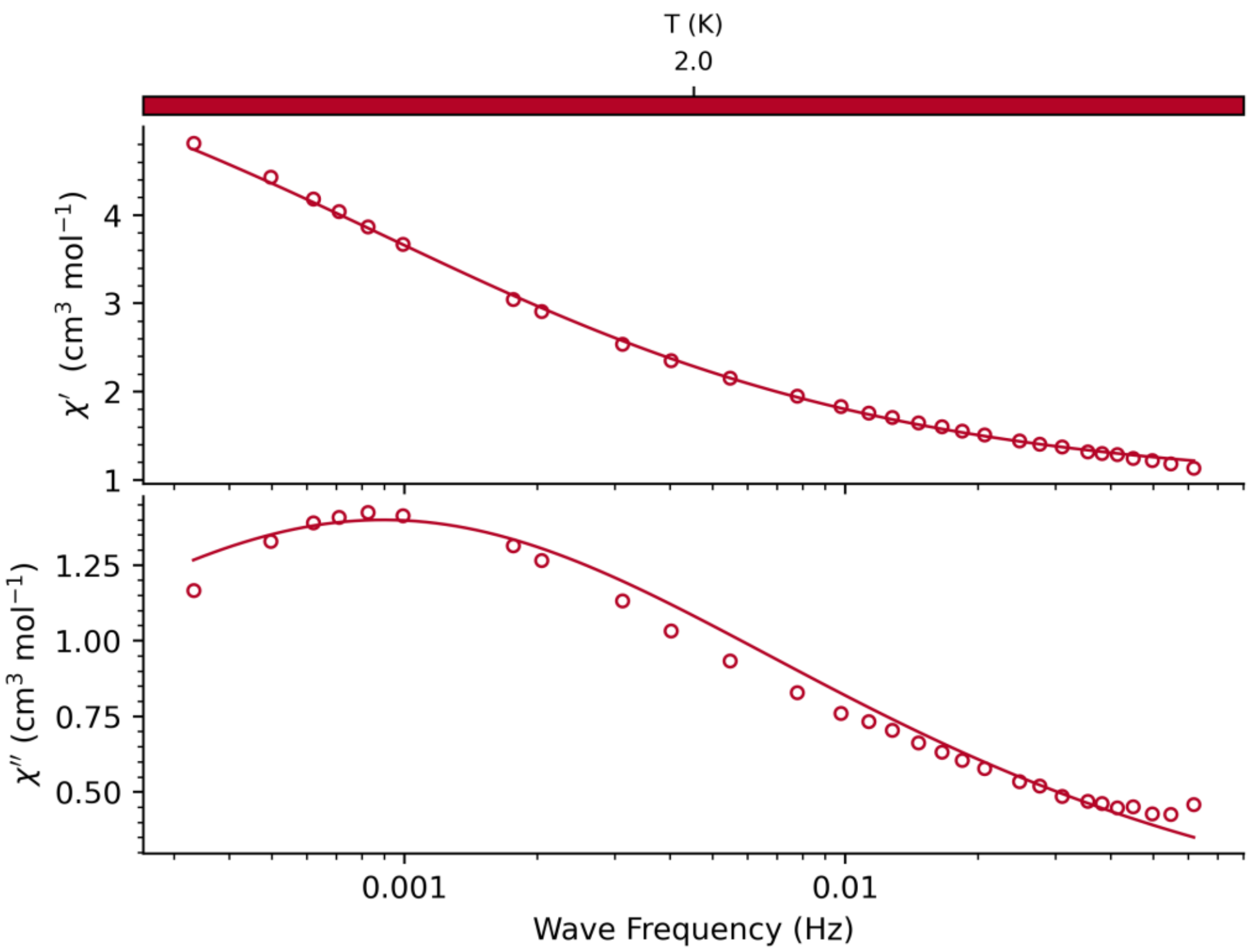

Figure S66. Susceptibility plot of waveform susceptibility data of **1-Dy** performed at 2 K fitted to a generalised Debye model.

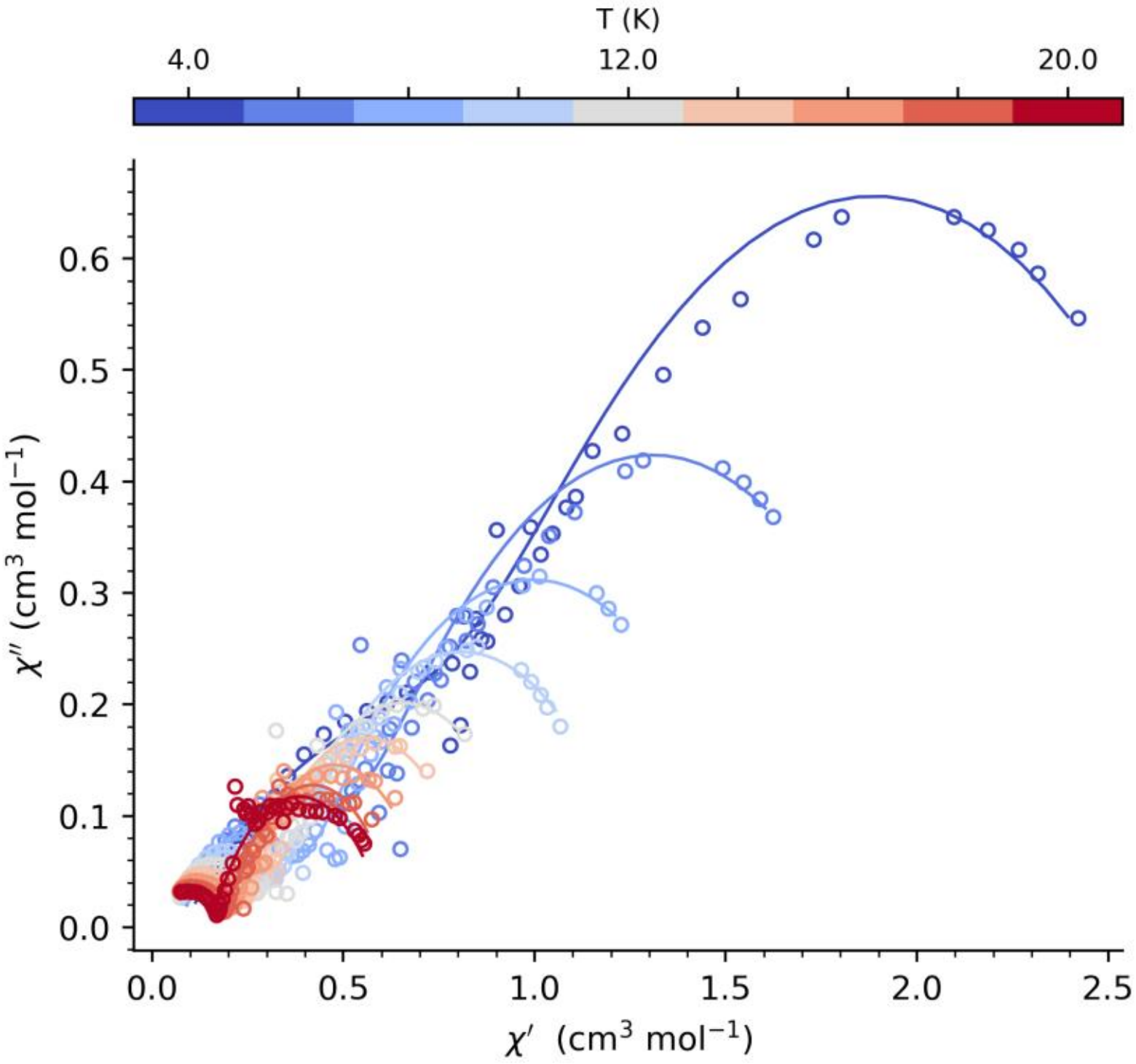

Figure S67. Cole-Cole plot of joint AC susceptibility and waveform susceptibility data of **1-Dy** performed at temperatures 4 ≤ T ≤ 20 K fitted to a double generalised Debye model with all parameters freely fitted.

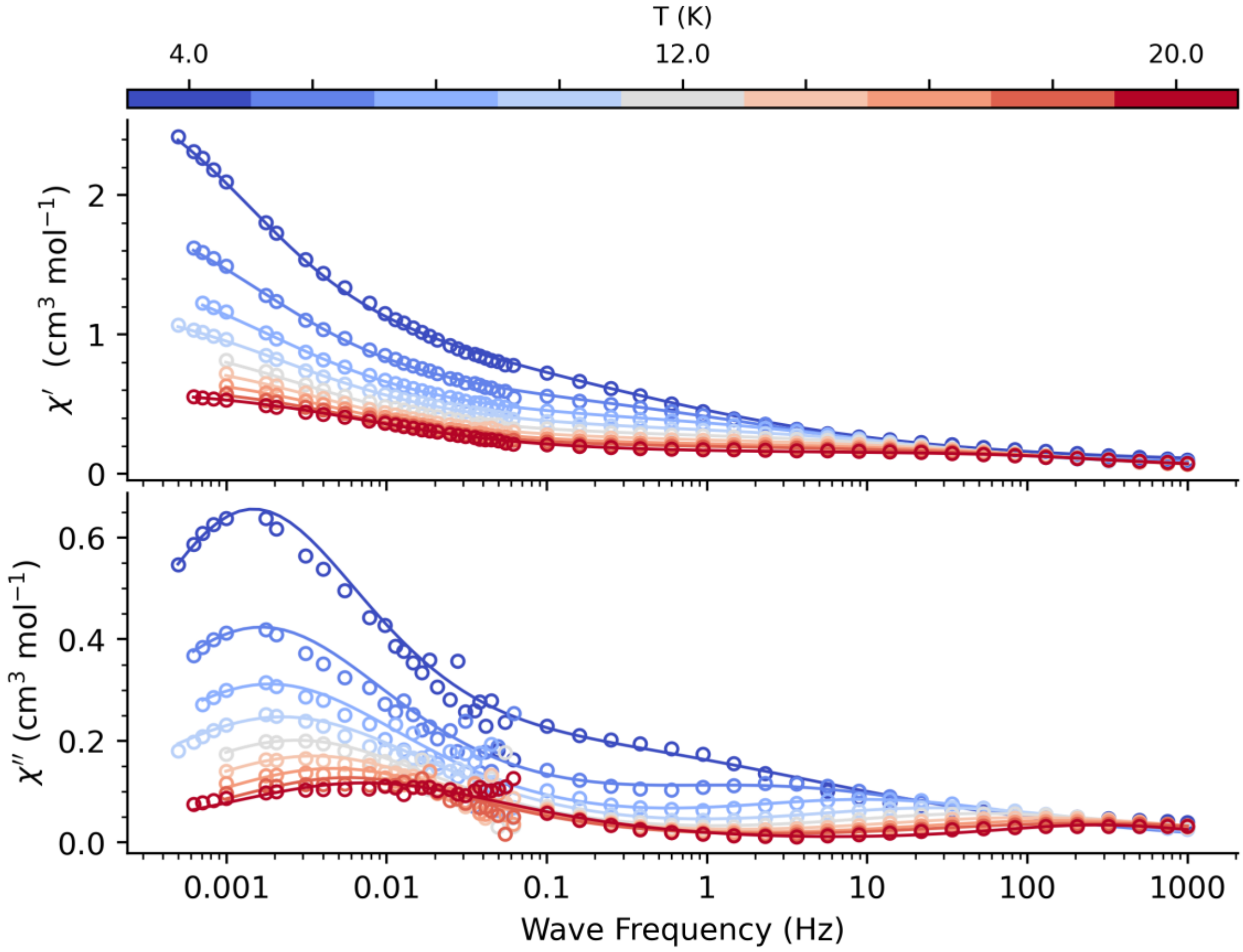

Figure S68. Susceptibility plots of joint AC susceptibility and waveform susceptibility data of **1-Dy** performed at temperatures 4 ≤ T ≤ 20 K fitted to a double generalised Debye model with all parameters freely fitted.

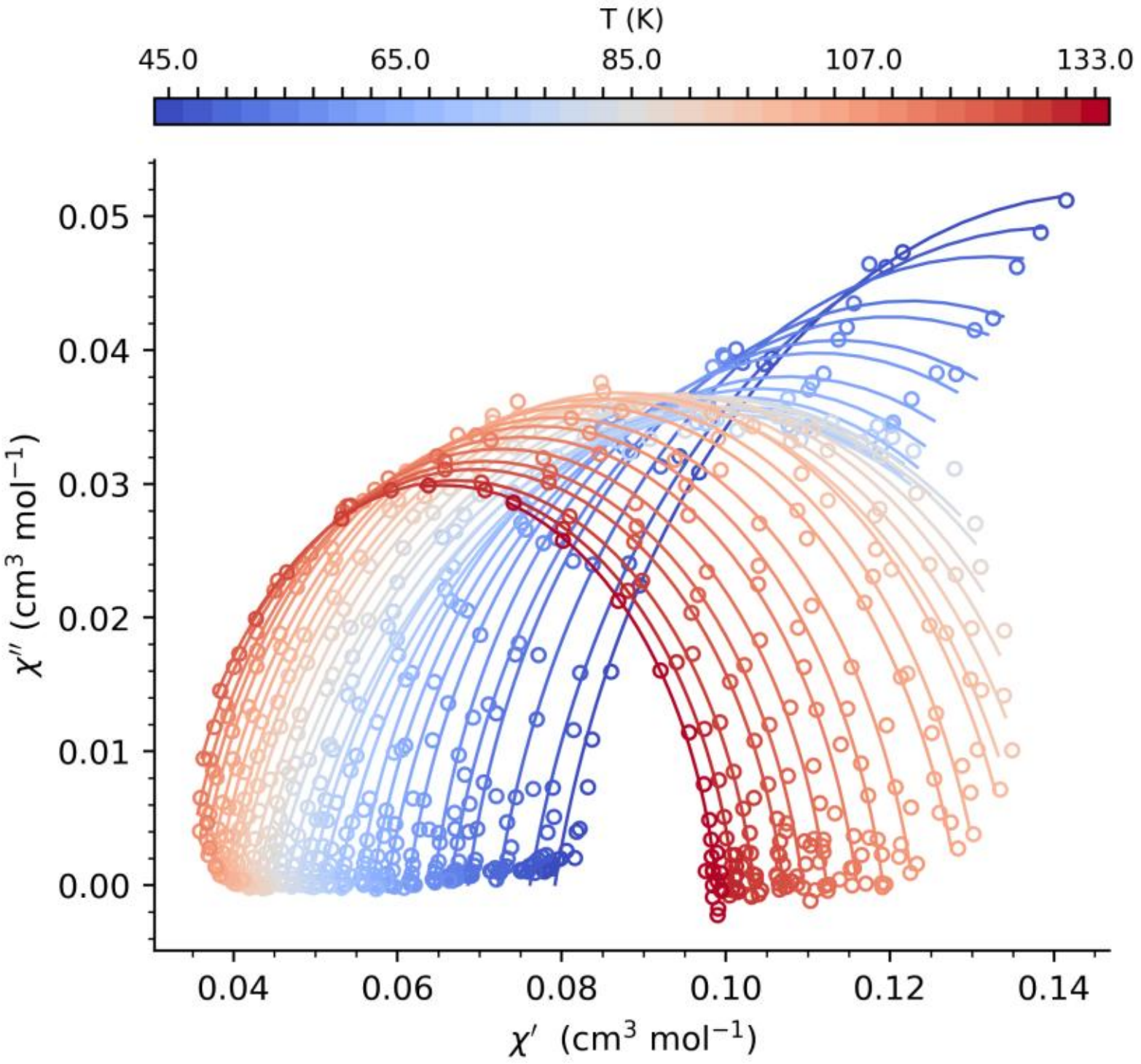

Figure S69. Cole-Cole plot of AC susceptibility data of **1-Dy** performed at temperatures 45 ≤ T ≤ 133 K fitted to a generalised Debye model with all parameters freely fitted.

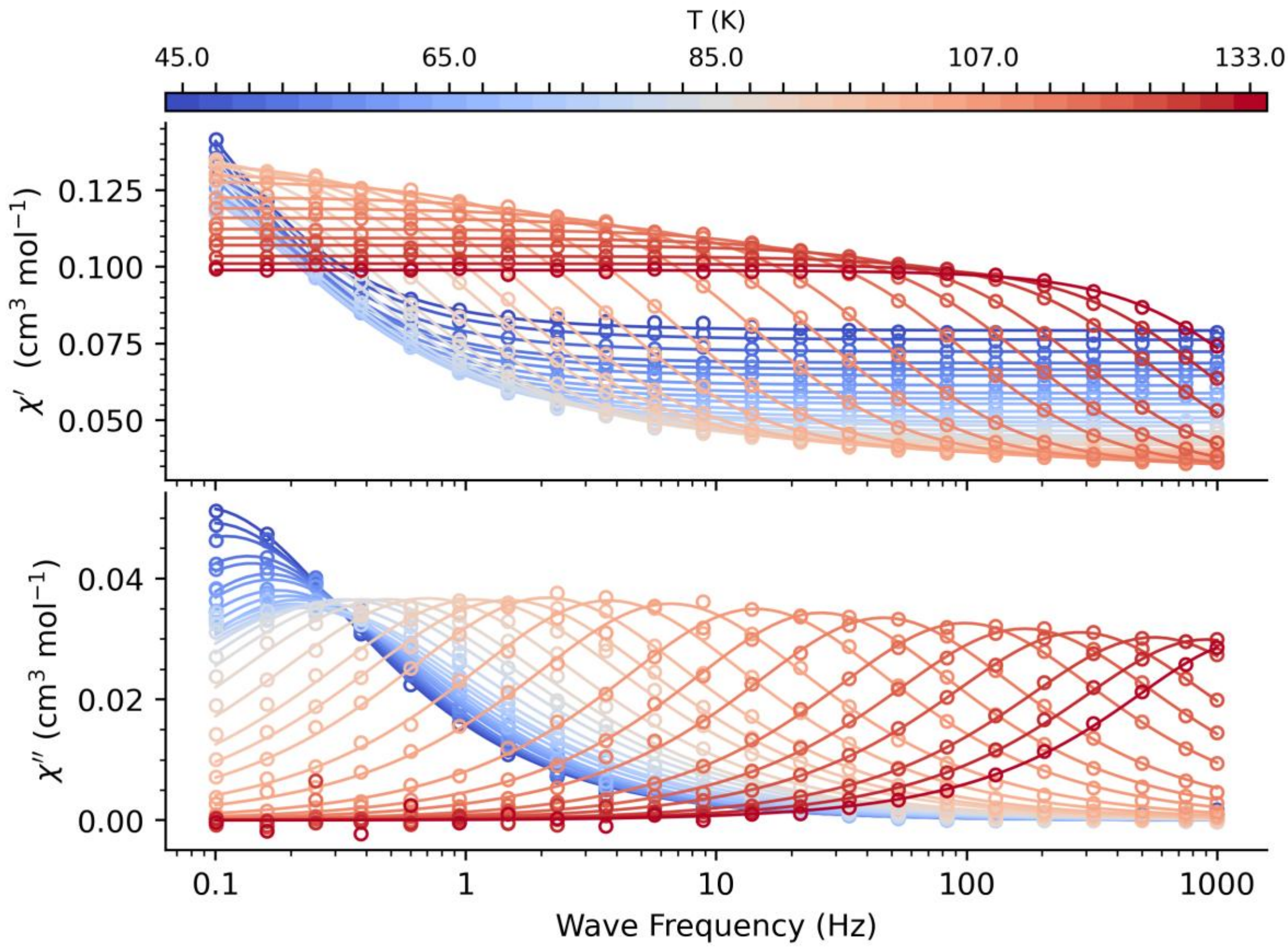

Figure S70. Susceptibility plots of AC susceptibility data of **1-Dy** performed at temperatures 45 ≤ T ≤ 133 K fitted to a generalised Debye model with all parameters freely fitted.

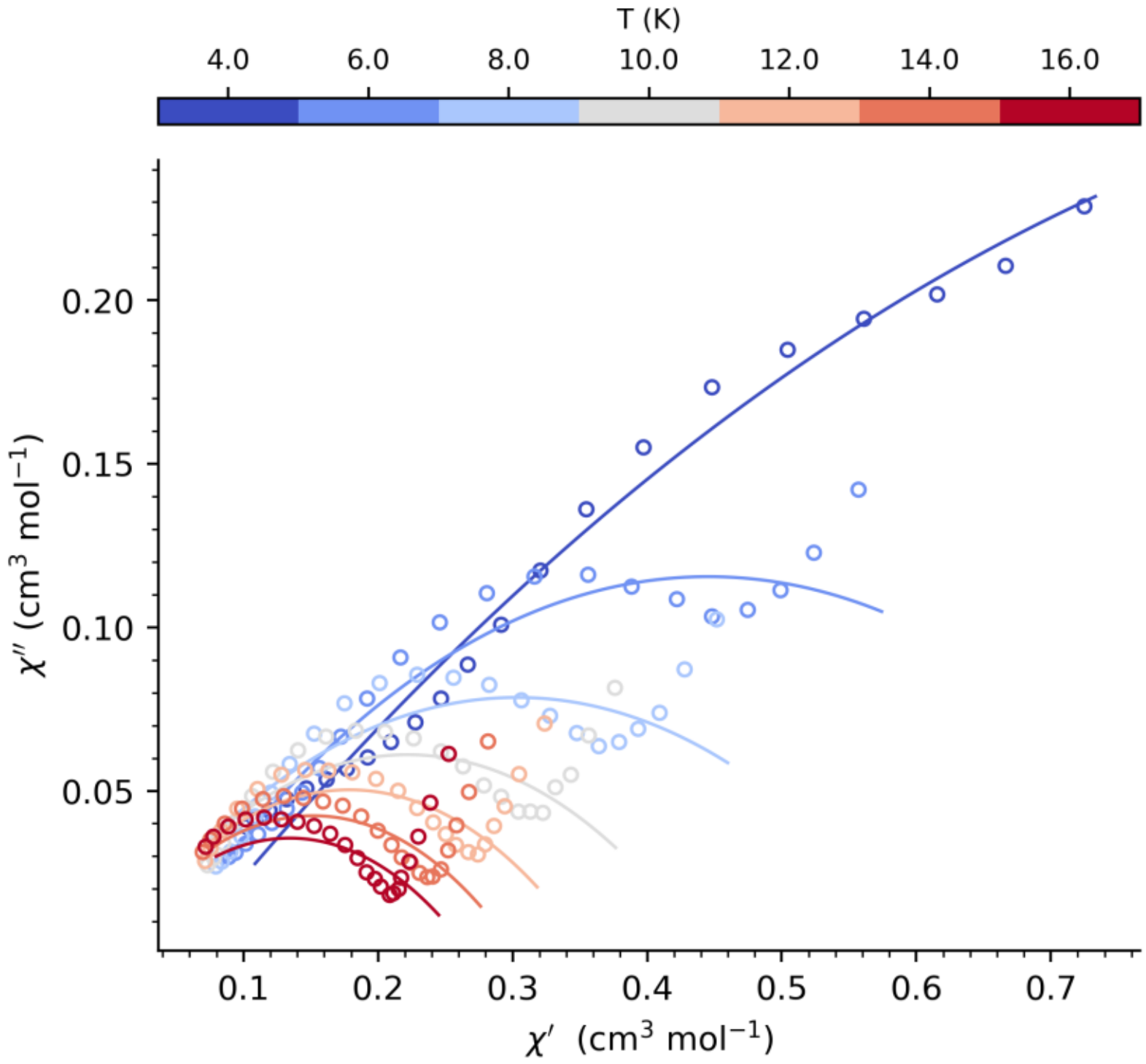

Figure S71. Cole-Cole plot of AC susceptibility of **1-Dy** performed at temperatures 4 ≤ T ≤ 16 K showing the relaxation of the fast process. The data fitted to a generalised Debye model with all parameters freely fitted.

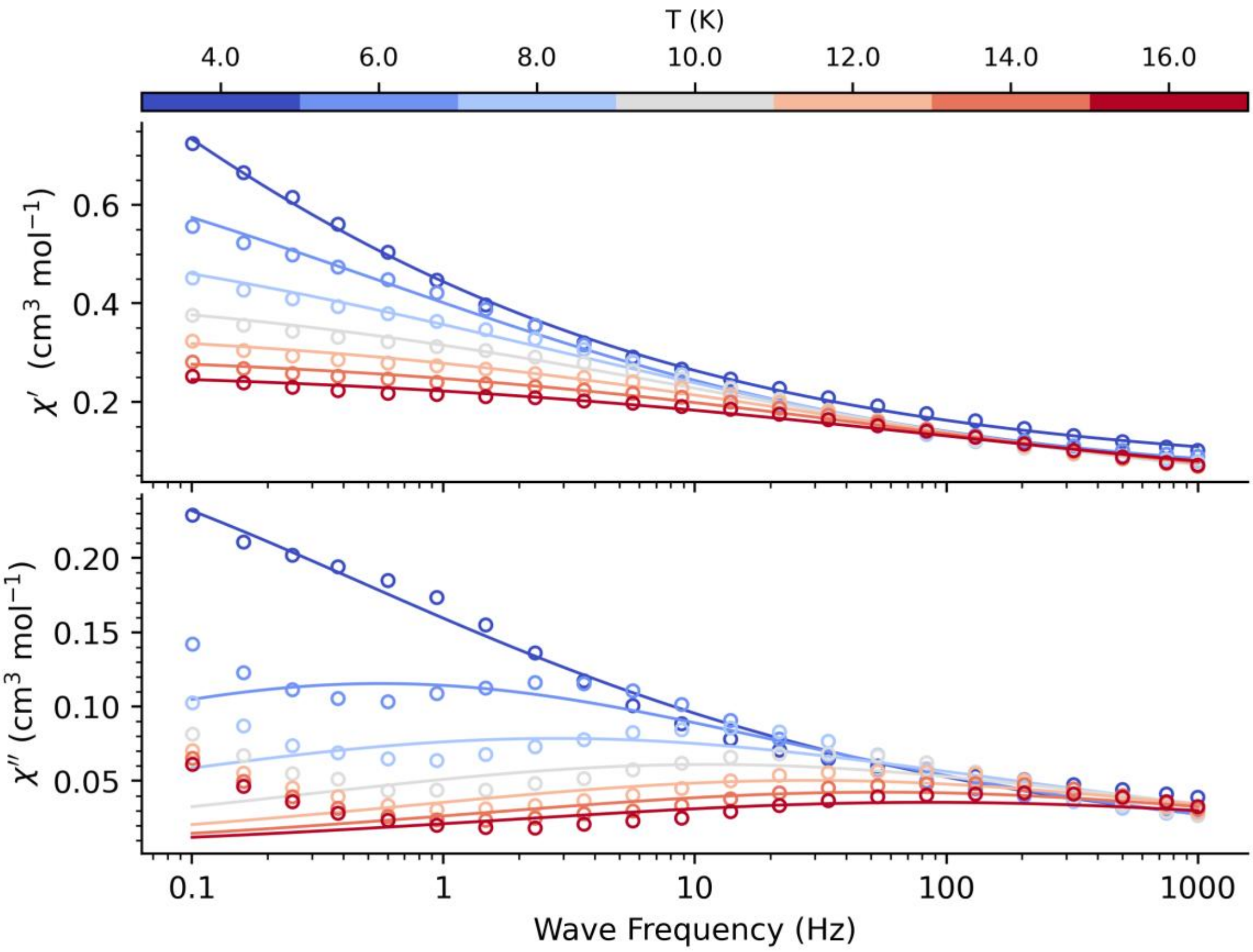

Figure S72. Susceptibility plots of AC susceptibility of **1-Dy** performed at temperatures 4 ≤ T ≤ 16 K showing the relaxation of the fast process. The data fitted to a generalised Debye model with all parameters freely fitted.

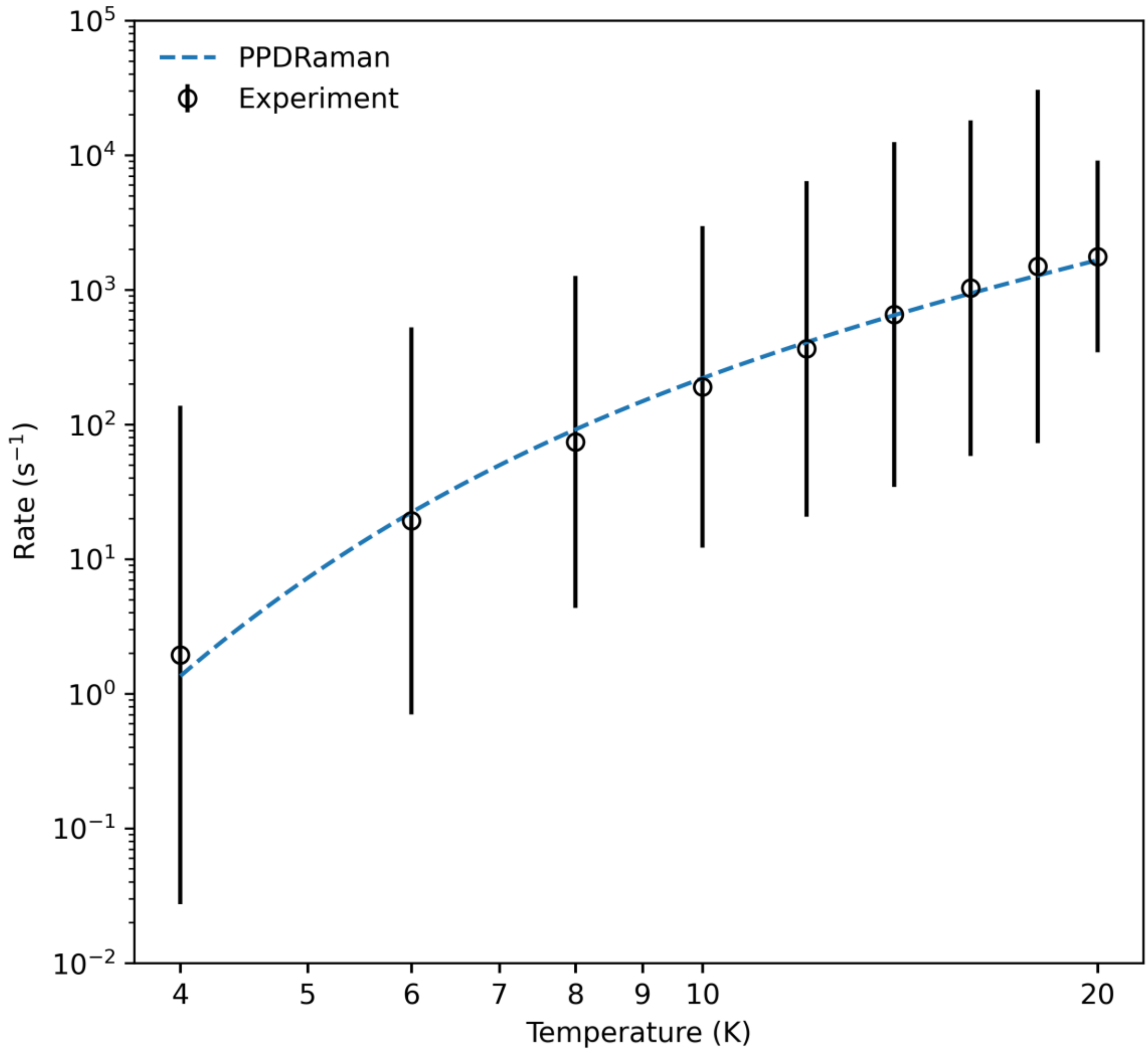

Figure S73. Relaxation profile of the fast process for **1-Dy** fitted to a phonon-pair driven Raman model ($\log[\tau^{-1}] = \log\left[10^R \frac{\exp(\omega/T)}{[\exp(\omega/T)-1]^2}\right]$) with extracted parameters $\omega = 33(1)$ K and $R = 3.77(6)$ log[s^{-1}].

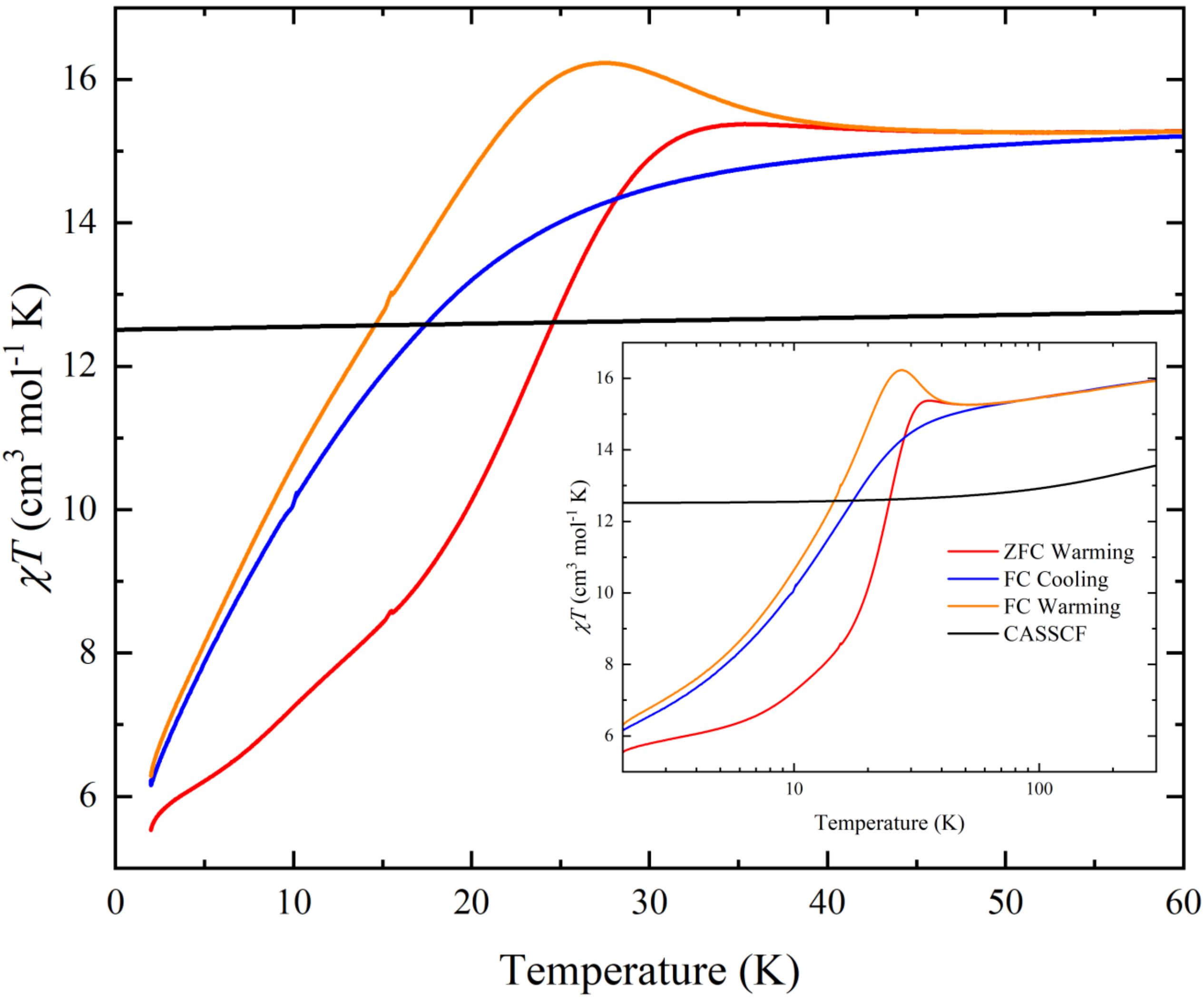

Figure S74. Molar magnetic susceptibility product (χ*T*) vs. temperature (*T*) for **2-Dy** performed at 0.1 T, showing ZFC and FC curves and CASSCF-calculated traces from 2–60 K on a linear scale and (inset) 2–300 K on a logarithmic scale.

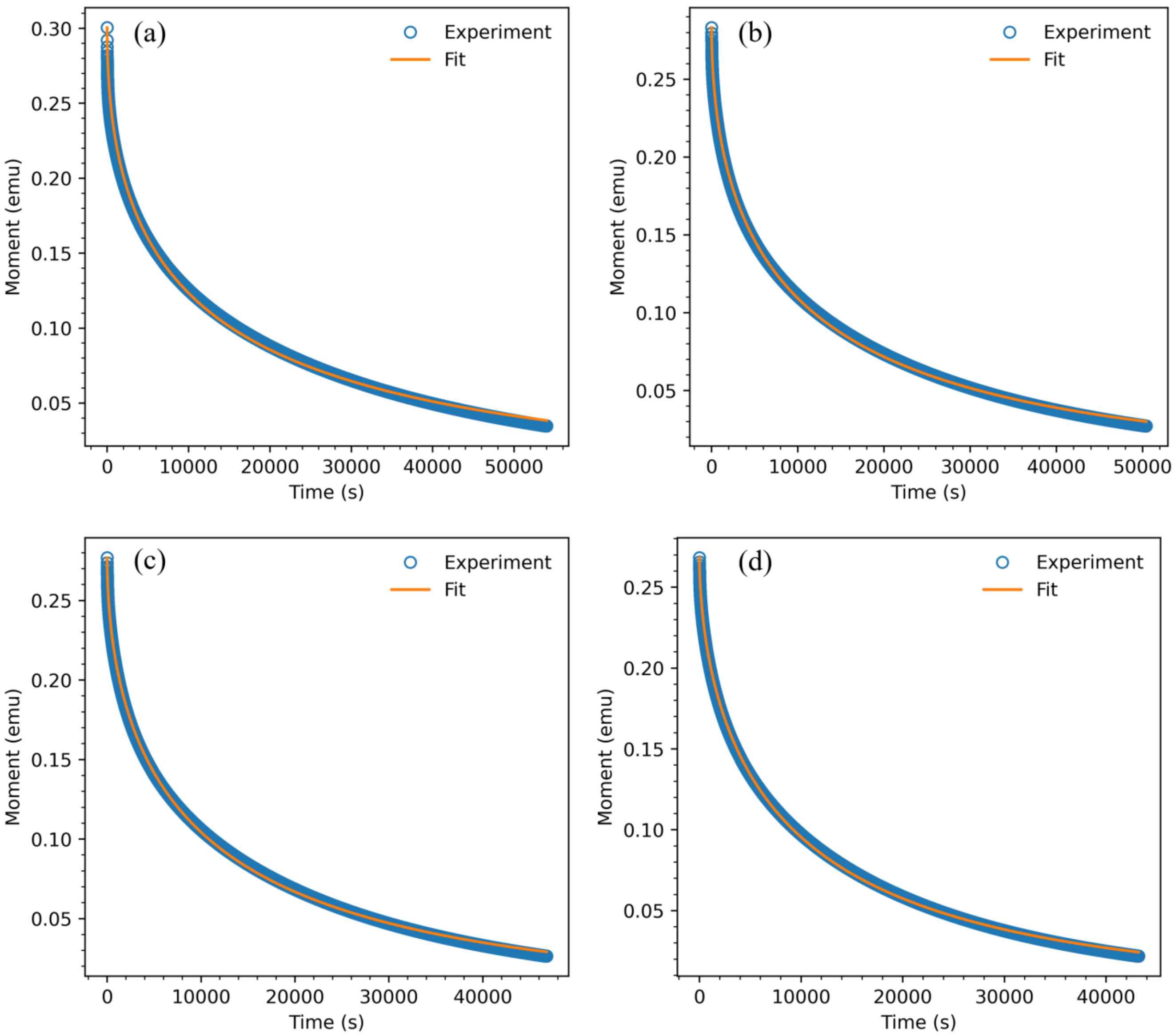

Figure S75. DC decay data of **2-Dy** performed at 2 K (a), 4 K (b), 6 K (c) and 8 K (d) fitted to a stretched exponential model.

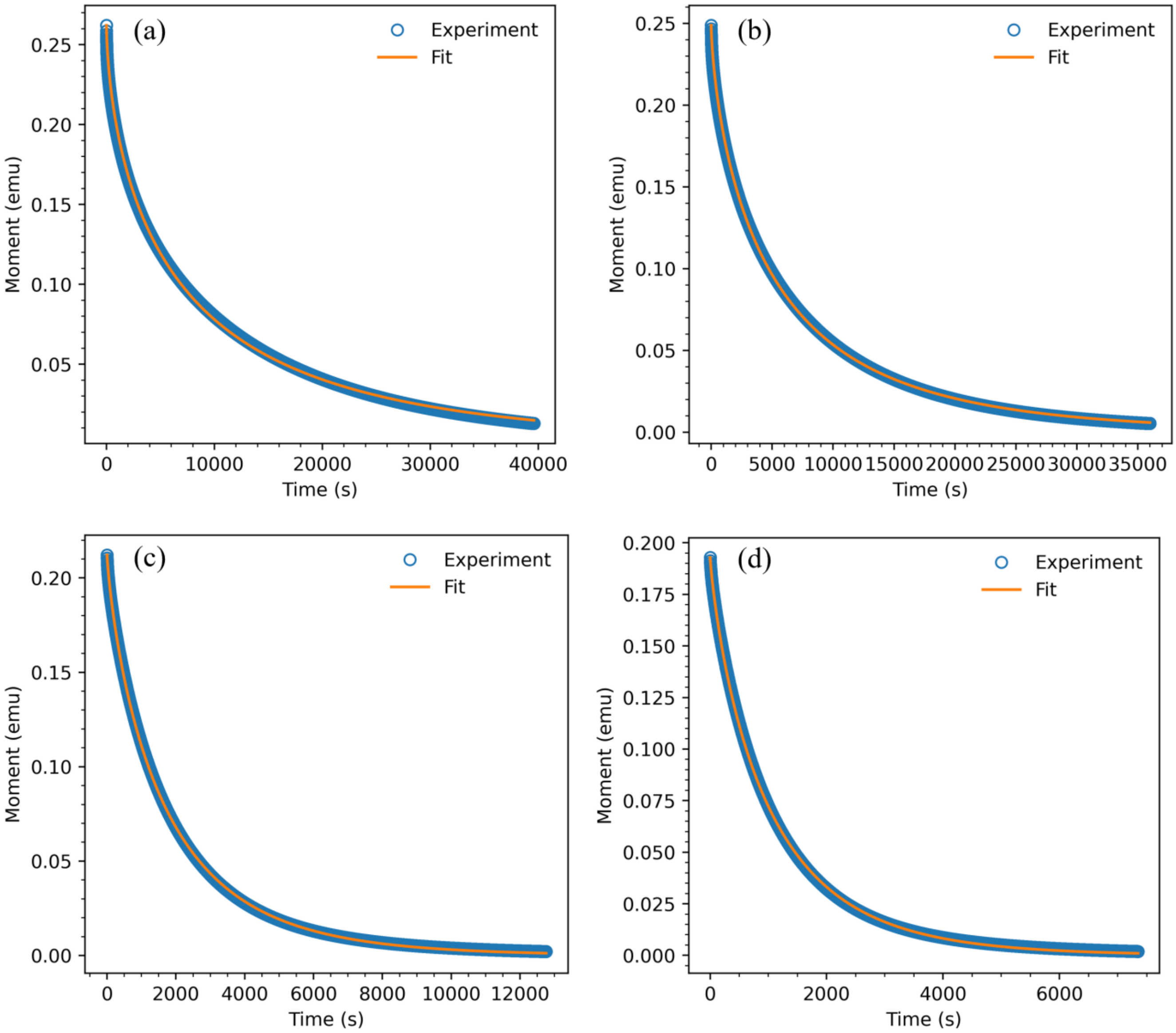

Figure S76. DC decay data of **2-Dy** performed at 10 K (a), 12 K (b), 18 K (c) and 21 K (d) fitted to a stretched exponential model.

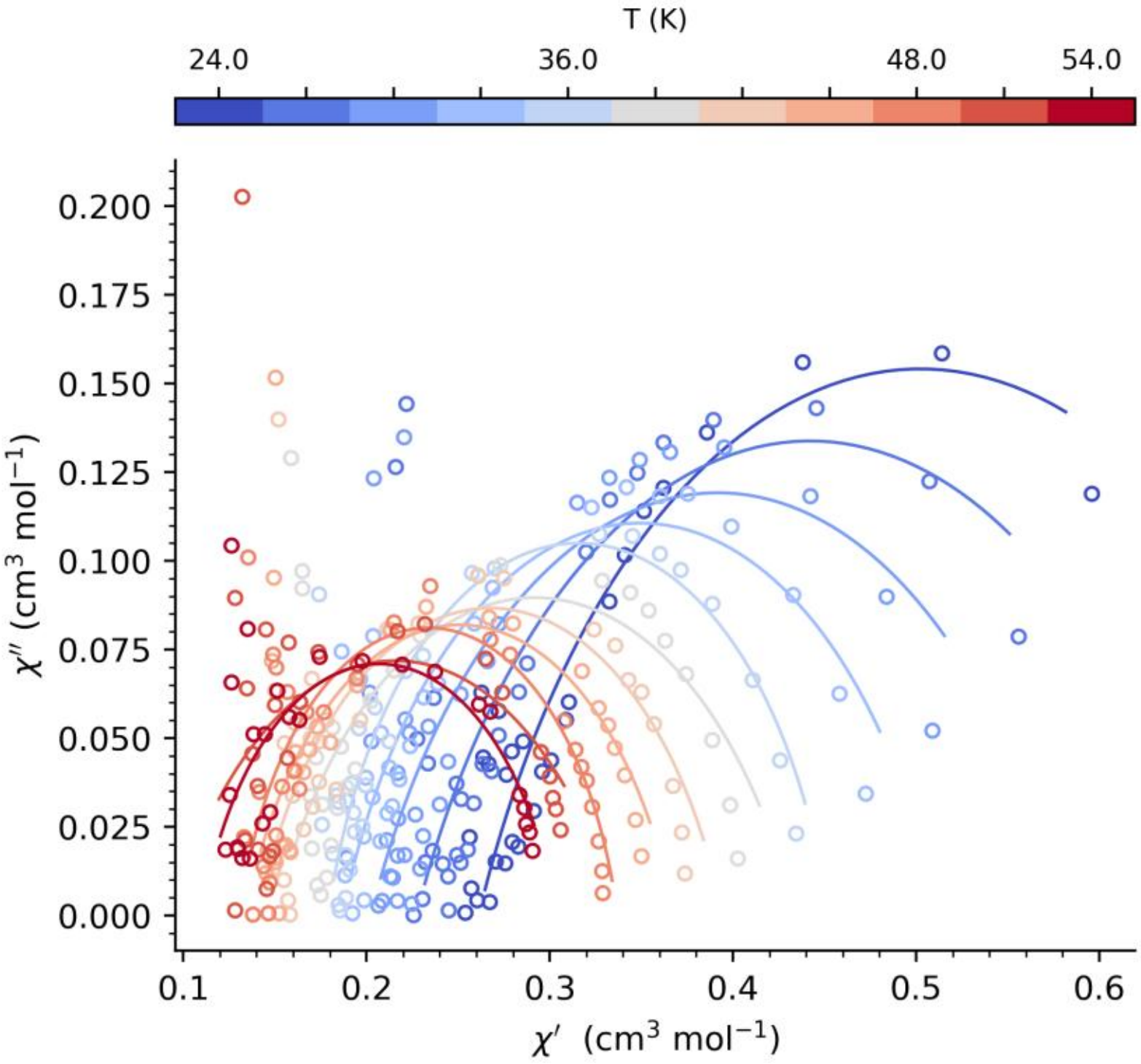

Figure S77. Cole-Cole plot of waveform susceptibility data of **2-Dy** performed at temperatures 24 ≤ *T* ≤ 54 K fitted to a generalised Debye model with all parameters freely fitted.

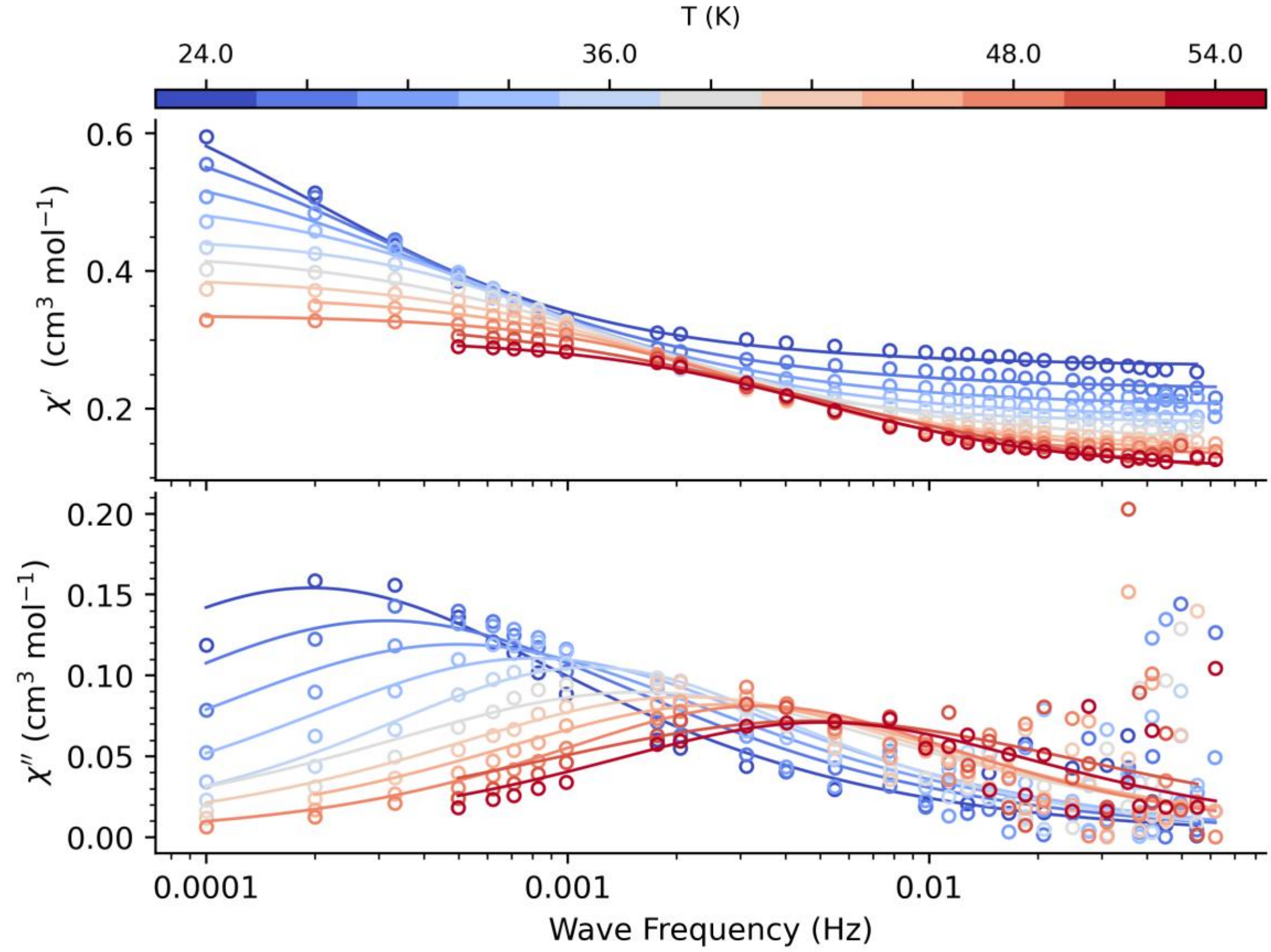

Figure S 78. Susceptibility plots of waveform susceptibility data of **2-Dy** performed at temperatures 24 ≤ *T* ≤ 54 K fitted to a generalised Debye model with all parameters freely fitted.

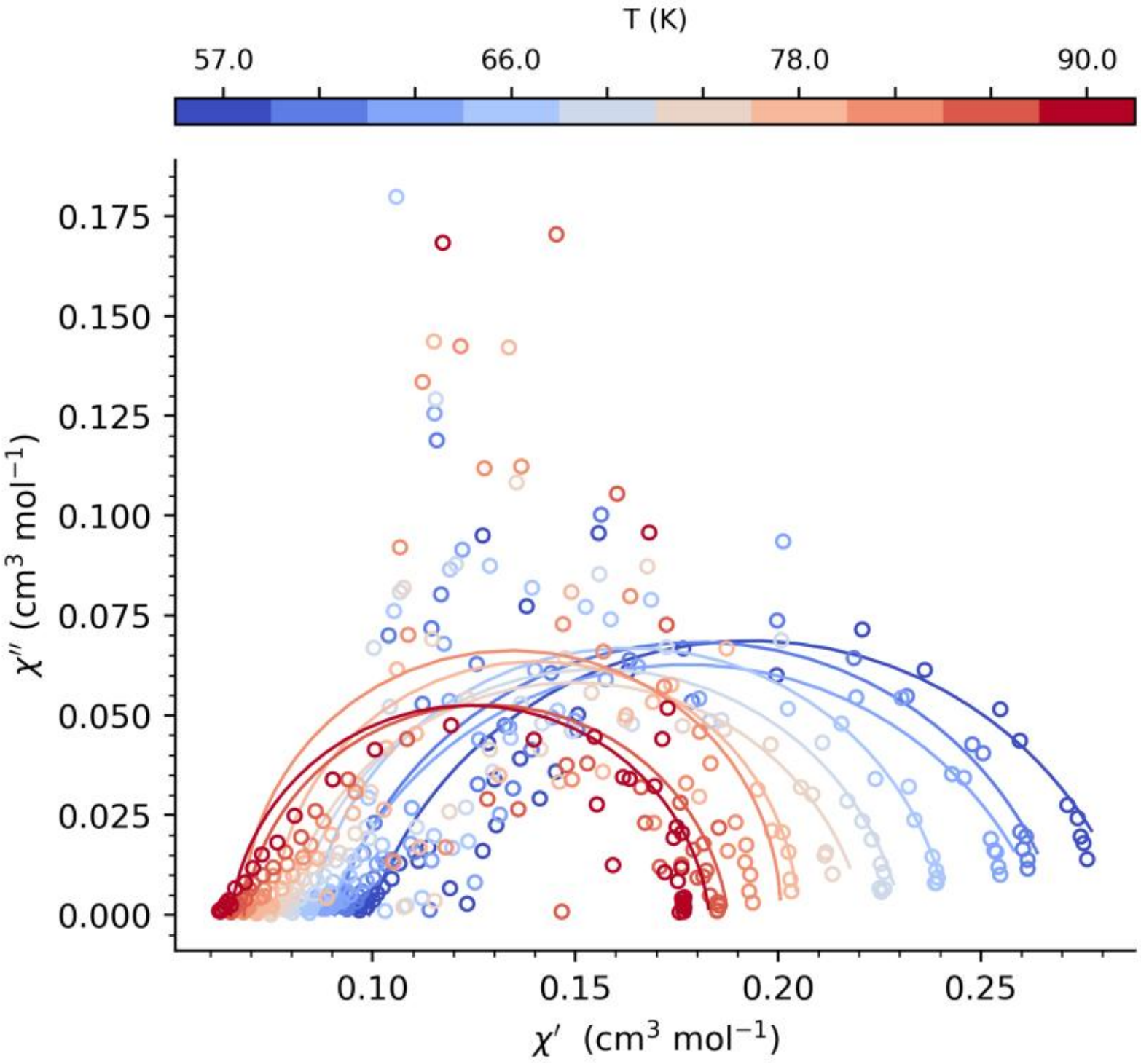

Figure S79. Cole-Cole plot of joint AC susceptibility and waveform susceptibility data of **2-Dy** performed at temperatures 57 ≤ T ≤ 90 K fitted to a generalised Debye model with all parameters freely fitted.

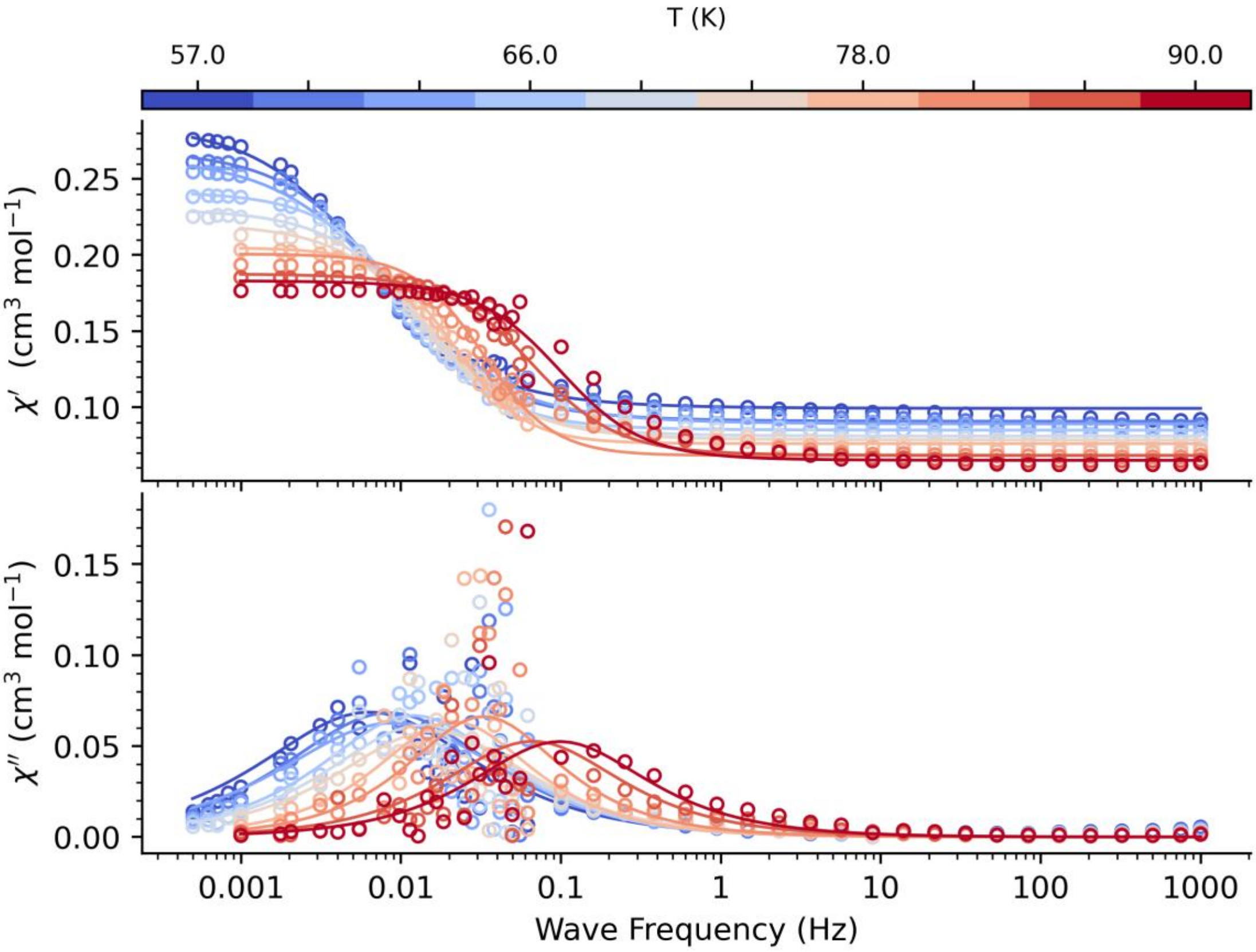

Figure S80. Susceptibility plots of joint AC susceptibility and waveform susceptibility data of **2-Dy** performed at temperatures 57 ≤ T ≤ 90 K fitted to a generalised Debye model with all parameters freely fitted.

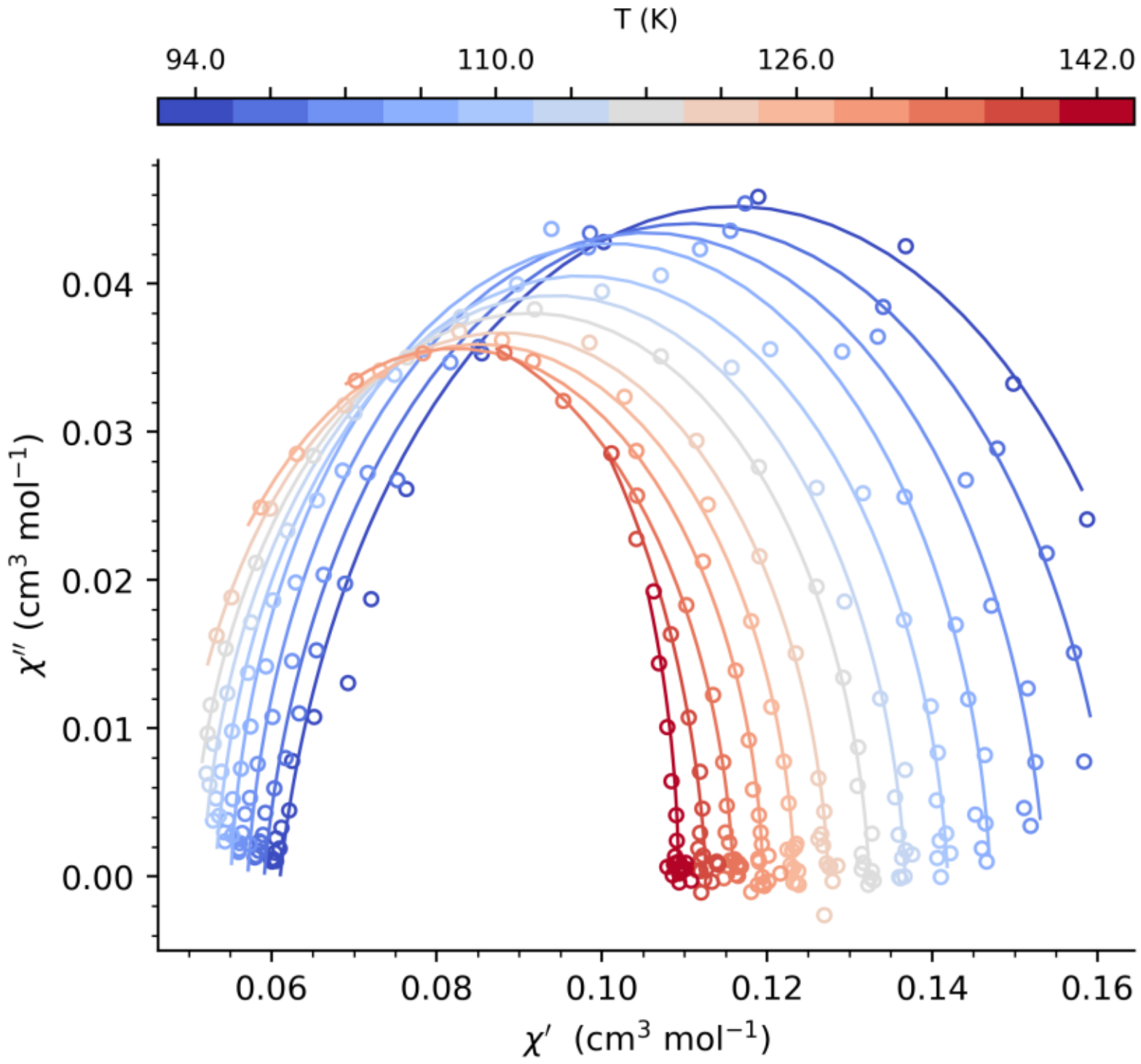

Figure S81. Cole-Cole plot of AC susceptibility data of **2-Dy** performed at temperatures 94 ≤ *T* ≤ 142 K fitted to a generalised Debye model with all parameters freely fitted.

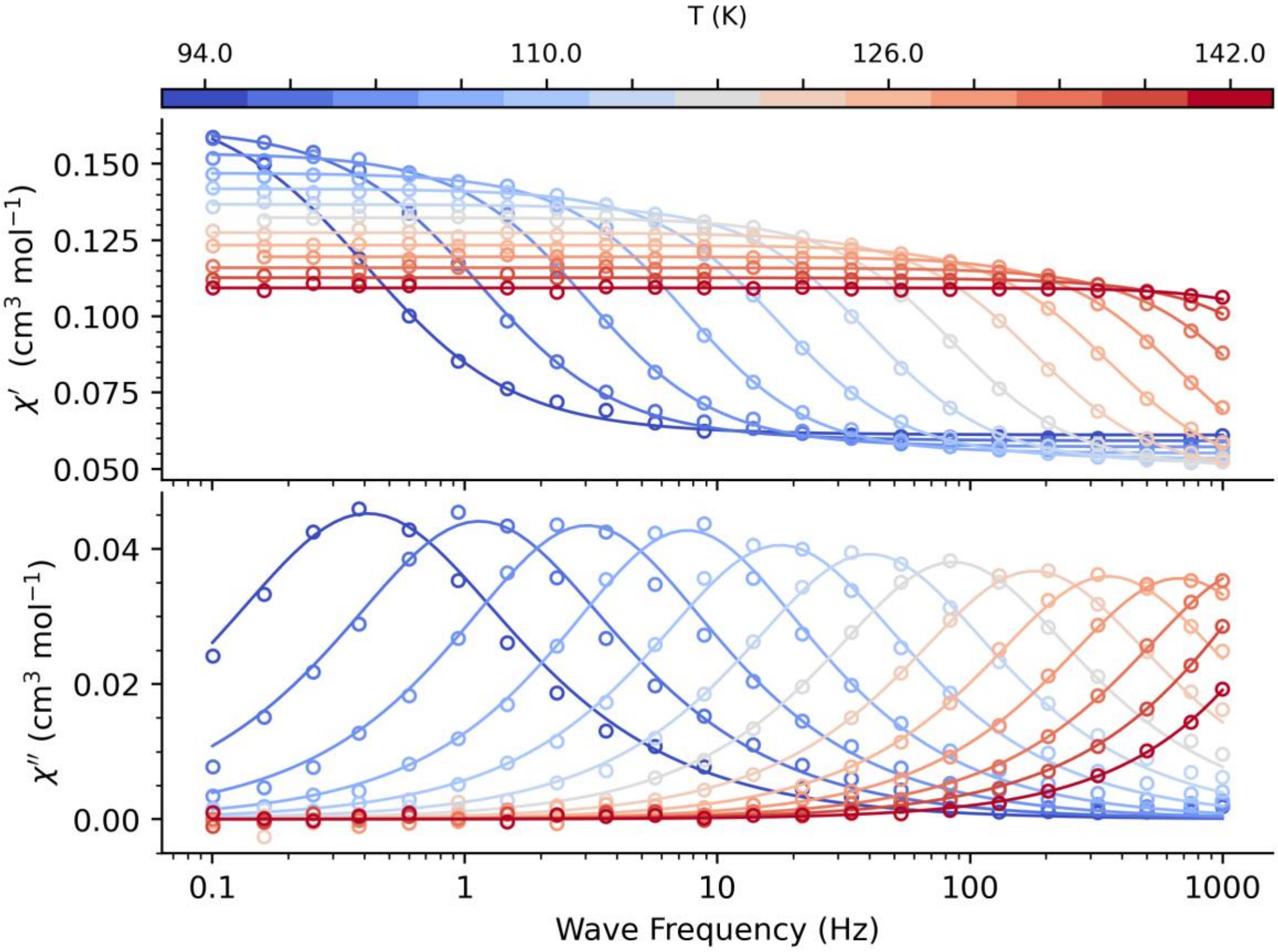

Figure S82. Susceptibility plots of AC susceptibility data of **2-Dy** performed at temperatures 94 ≤ *T* ≤ 142 K fitted to a generalised Debye model with all parameters freely fitted.

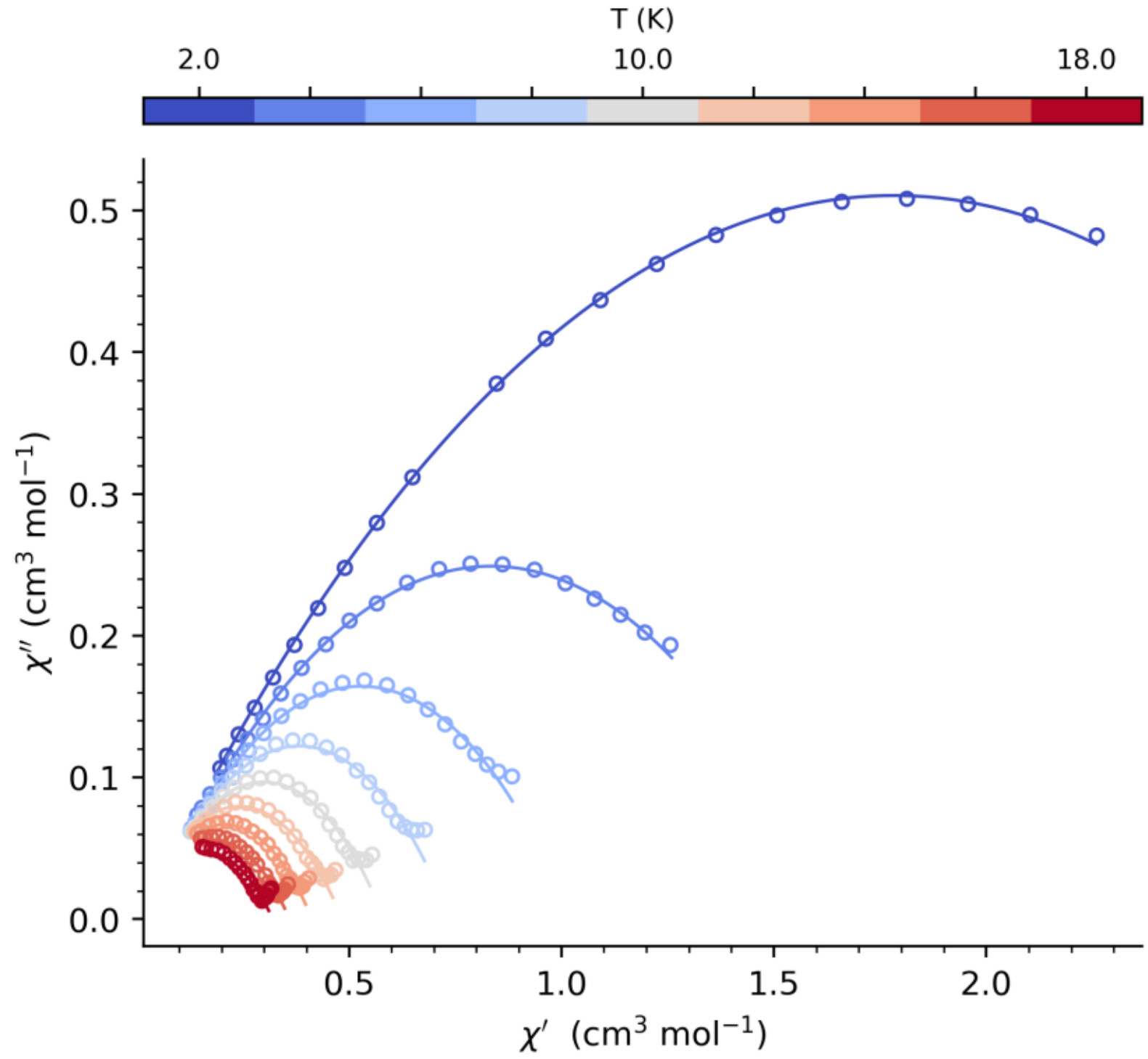

Figure S83. Cole-Cole plot of AC susceptibility of **2-Dy** performed at temperatures 2 ≤ *T* ≤ 18 K showing the relaxation of the fast process. The data are fitted to a generalised Debye model with all parameters freely fitted.

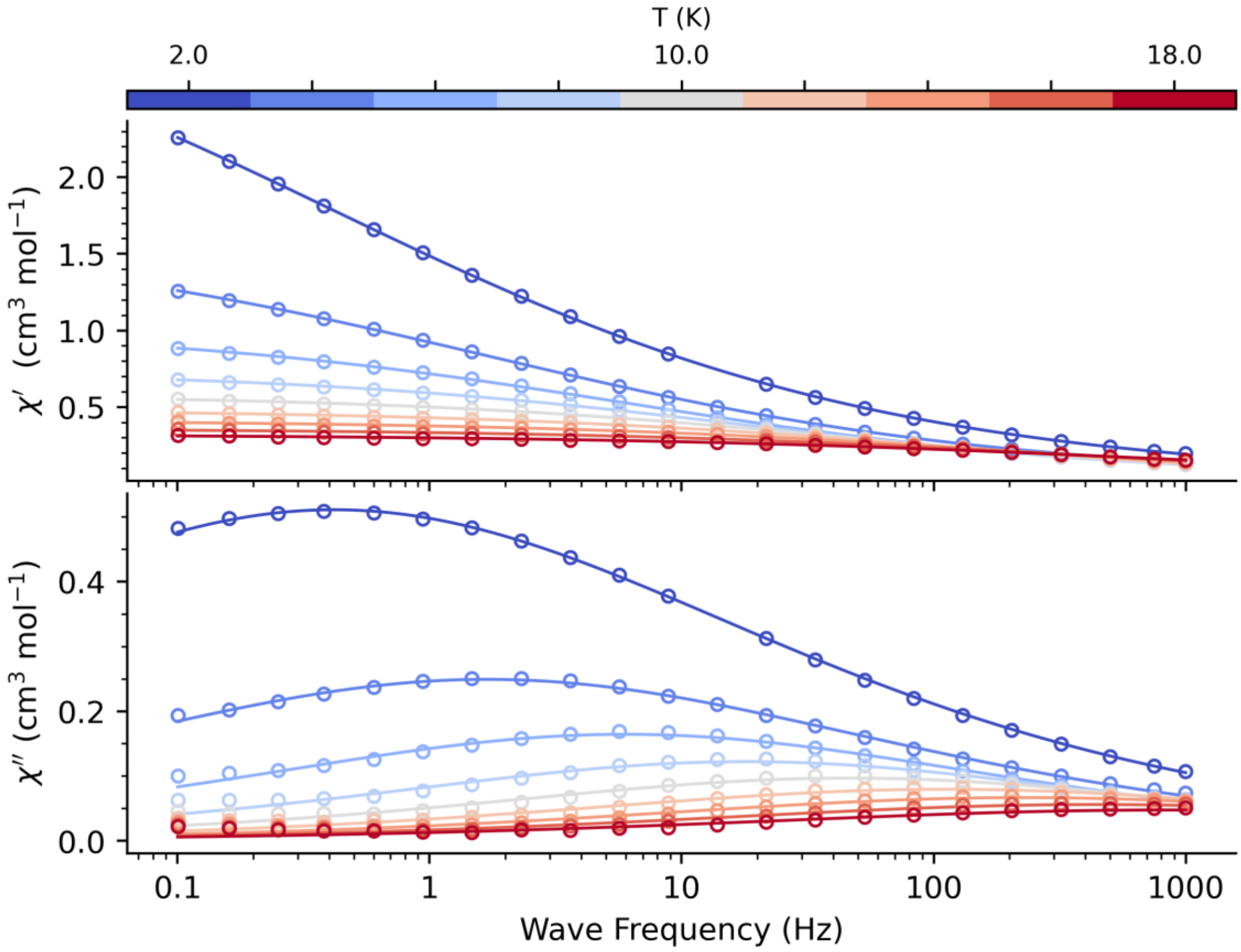

Figure S84. Susceptibility plots of AC susceptibility of **2-Dy** performed at temperatures 2 ≤ *T* ≤ 18 K showing the relaxation of the fast process. The data are fitted to a generalised Debye model with all parameters freely fitted.

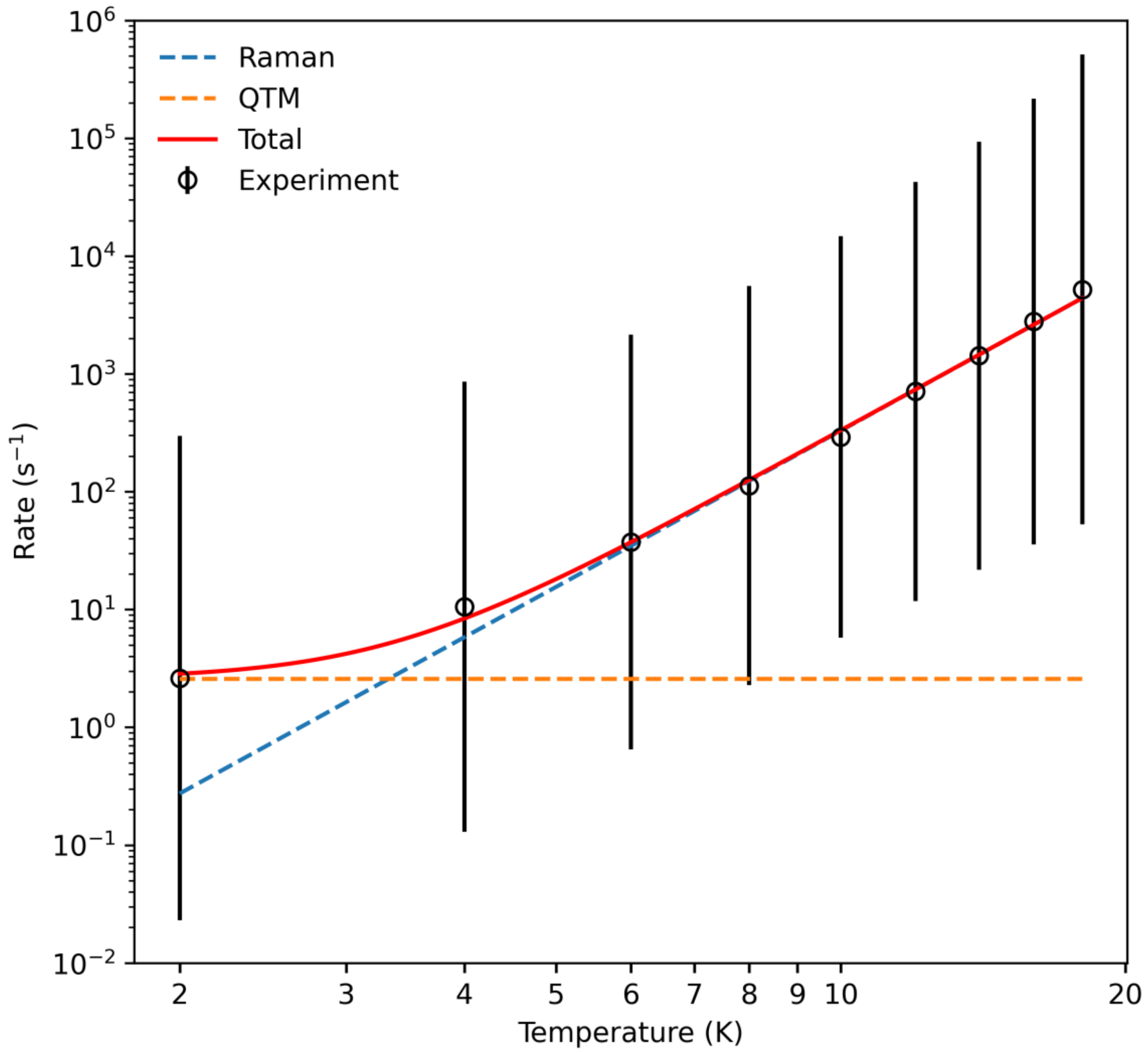

Figure S85. Relaxation profile for the fast process of **2-Dy** fitted to a model containing Raman and QTM contributions ($\log[\tau^{-1}] = \log[10^R T^n + 10^{-Q}]$) with extracted parameters $R = -1.9(1)$ log[s^{-1}K^{-n}], $n = 4.4(1)$ and $Q = -0.42(8)$ log[s].

Table S5. Relaxation parameters of **1-Dy** extracted from AC susceptibility and waveform data. The values in parentheses gives the error on the parameters. 2 K data is from a waveform-only dataset fit to a generalised Debye model. 4 – 20 K data are from a joint waveform/AC dataset and fitted to a double generalised Debye model. 45 – 133 K data are from an AC-only dataset fit to a generalised Debye model.

T / K	τ_{slow} / s	α_{slow}	τ_{fast} / s	α_{fast}
2	178(10)	0.421(11)		
4	118(5)	0.292(28)	5(3)×10^{-1}	0.61(4)
6	100(8)	0.361(29)	5(2)×10^{-2}	0.52(5)
8	87(8)	0.386(23)	1.4(3)×10^{-3}	0.46(5)
10	72(8)	0.401(10)	5.2(6)×10^{-3}	0.45(3)
12	59(8)	0.373(33)	2.7(8)×10^{-3}	0.47(8)
14	46(4)	0.369(22)	1.5(4)×10^{-3}	0.48(6)

16	34(3)	0.355(21)	1.0(3)×10^{-3}	0.47(7)
18	28(2)	0.345(23)	7(3)×10^{-4}	0.48(8)
20	22(1)	0.401(17)	6(3)×10^{-4}	0.3(1)
45	1.78(10)	0.160(12)		
47	1.64(7)	0.165(10)		
50	1.44(5)	0.152(10)		
53	1.18(4)	0.133(9)		
55	1.15(3)	0.146(7)		
57	0.98(3)	0.121(10)		
60	0.96(3)	0.142(12)		
63	0.90(3)	0.156(12)		
65	0.82(3)	0.154(14)		
67	0.75(3)	0.152(16)		
70	0.77(3)	0.192(13)		
73	0.74(4)	0.212(16)		
75	0.71(4)	0.213(18)		
77	0.66(3)	0.232(16)		
80	0.59(3)	0.237(15)		
83	0.49(2)	0.235(13)		
85	0.41(1)	0.233(11)		
87	0.326(7)	0.215(9)		
90	0.227(4)	0.200(8)		
93	0.147(2)	0.195(7)		
95	0.108(2)	0.184(7)		
97	7.56(10)×10^{-2}	0.170(6)		
100	4.29(5)×10^{-2}	0.156(6)		
103	2.42(3)×10^{-2}	0.154(5)		
107	1.11(1)×10^{-2}	0.138(5)		
110	6.08(6)×10^{-3}	0.129(5)		
113	3.42(5)×10^{-3}	0.126(7)		
117	1.62(2)×10^{-3}	0.124(7)		
120	9.36(14)×10^{-4}	0.126(8)		
123	5.63(6)×10^{-4}	0.121(5)		
127	2.85(14)×10^{-4}	0.116(19)		
130	1.76(7)×10^{-4}	0.109(12)		
133	1.11(11)×10^{-4}	0.099(28)		

Table S6. Relaxation parameters of **2-Dy** extracted from modelling DC decay data. The errors are smaller than the reported precision.

T / K	τ^* / s	β
2	1.26×10^{4}	0.497
4	1.10×10^{4}	0.529
6	1.05×10^{4}	0.542
8	9.45×10^{3}	0.576
10	7.35×10^{3}	0.626
12	5.39×10^{3}	0.696
18	1.71×10^{3}	0.815
21	1.02×10^{3}	0.842

Table S7. Relaxation parameters of **1-Dy** extracted from AC susceptibility and waveform data. The values in parentheses gives the error on the parameters. Asterisk (*) indicates errors larger than the reported values. 2 – 18 K data are from an AC-only dataset fitted to a generalised Debye model. 24 – 54 K data are from a waveform-only dataset fitted to a generalised Debye model. 57 – 90 K data are from a joint waveform/AC dataset fitted to a generalised Debye model. 94 – 142 K data are from an AC-only dataset fitted to a generalised Debye model.

T / K	τ_{slow} / s	α_{slow}	τ_{fast} / s	α_{fast}
2			$3.81(7)\times10^{-1}$	0.642(1)
4			$9.4(2)\times10^{-2}$	0.619(2)
6			$2.67(8)\times10^{-2}$	0.592(5)
8			$8.9(4)\times10^{-3}$	0.578(9)
10			$3.4(3)\times10^{-3}$	0.581(12)
12			$1.4(2)\times10^{-3}$	0.595(15)
14			$7(4)\times10^{-4}$	0.602(18)
16			$4(1)\times10^{-4}$	0.616(24)
18			$2(1)\times10^{-4}$	0.633(29)
24	812(144)	0.276(38)		
27	505(142)	0.291(67)		
30	321(71)	0.285(61)		
33	197(22)	0.240(41)		
36	138(11)	0.165(36)		
39	100(17)	0.284(65)		
42	75(10)	0.237(59)		
45	59(9)	0.222(71)		

48	47(4)	0.151(49)
51	34(9)	0.32(12)
54	32(4)	0.195(65)
57	25(2)	0.196(31)
60	19(2)	0.170(37)
62	18(2)	0.214(42)
66	13(1)	0.102(54)
70	11(1)	0.123(42)
74	10.0(9)	0.129(43)
78	7.4(6)	0.009*
82	4.8(4)	0.009*
86	2.4(3)	0.080(75)
90	1.6(2)	0.075(75)
94	$3.87(9)\times10^{-1}$	0.123(11)
98	$1.40(3)\times10^{-1}$	0.098(10)
102	$5.22\ (8)\times10^{-2}$	0.067(9)
106	$2.10(3)\times10^{-2}$	0.047(7)
110	$9.0(1)\times10^{-3}$	0.057(7)
114	$3.97(5)\times10^{-3}$	0.052(7)
118	$1.85(2)\times10^{-3}$	0.049(7)
122	$8.90(1)\times10^{-4}$	0.047(9)
126	$4.51(6)\times10^{-4}$	0.038(7)
130	$2.37(6)\times10^{-4}$	0.030(11)
134	$1.15(5)\times10^{-4}$	0.046(12)
138	$4.8(9)\times10^{-5}$	0.060(25)
142	$3(1)\times10^{-5}$	0.008*

6. Computational data

6.1 Static electronic structure calculations

Table S8. SA-CASSCF-SO-calculated energy levels of the $^6H_{15/2}$ multiplet for **1-Dy^a**, the cation of **1-Dy** with N–Dy–$Cp^{ttt}_{centroid}$ = 157.84(11)° at the crystal structure geometry.*

Energy / cm^{-1}	Energy / K	g_x	g_y	g_z	Angle (°)	$\langle J_z \rangle$	Wavefunction composition
0	0	0.00	0.00	20.00	0.00	7.50	100% |±15/2>
585	836	0.00	0.00	17.01	4.10	6.49	99% |±13/2>
1060	1515	0.00	0.00	14.20	3.65	5.48	97% |±11/2>
1418	2026	0.12	0.13	11.68	7.00	4.50	98% |±9/2>
1645	2351	1.20	1.68	9.91	27.24	3.41	94% |±7/2>
1787	2553	0.33	3.27	7.61	39.59	2.28	75% |±5/2> + 18% |±3/2>
1877	2682	3.13	5.23	7.31	89.55	1.21	47% |±3/2> + 33% |±1/2> + 15% |±5/2>
1945	2779	0.74	3.52	15.86	82.05	0.87	58% |±1/2> + 34% |±3/2>

Table S9. SA-CASSCF-SO-calculated energy levels of the $^6H_{15/2}$ multiplet for **1-Dy^b**, the cation of **1-Dy** with N–Dy–$Cp^{ttt}_{centroid}$ = 167.90(10)° at the crystal structure geometry.*

Energy / cm^{-1}	Energy / K	g_x	g_y	g_z	Angle (°)	$\langle J_z \rangle$	Wavefunction composition
0	0	0.00	0.00	20.00	0.00	7.50	100% |±15/2>
610	872	0.00	0.00	16.98	2.10	6.49	100% |±13/2>
1088	1554	0.00	0.00	14.20	1.74	5.49	99% |±11/2>
1434	2049	0.05	0.06	11.64	5.02	4.50	99% |±9/2>
1664	2378	0.99	1.21	9.28	16.60	3.44	97% |±7/2>
1807	2582	1.97	5.15	6.35	27.52	2.22	82% |±5/2> + 9% |±3/2>
1891	2702	2.23	6.01	10.67	86.99	0.88	58% |±3/2> + 17% |∓1/2> + 12% |±1/2> + 6% |±5/2> + 5% |∓5/2>
1991	2845	0.19	0.58	18.14	85.35	0.56	47% |±1/2> + 25% |±3/2> + 18% |∓1/2> + 5% |∓3/2>

Table S10. SA-CASSCF-SO-calculated energy levels of the $^6H_{15/2}$ multiplet for **2-Dy** at the crystal structure geometry.*

Energy / cm^{-1}	Energy / K	g_x	g_y	g_z	Angle (°)	$\langle J_z \rangle$	Wavefunction composition
0	0	0.00	0.00	20.00	0.00	7.50	100% |±15/2>
622	888	0.00	0.00	16.97	1.64	6.49	100% |±13/2>
1101	1574	0.01	0.01	14.19	2.18	5.49	99% |±11/2>
1446	2066	0.07	0.08	11.57	2.39	4.48	99% |±9/2>
1680	2400	0.62	0.75	9.07	8.51	3.48	98% |±7/2>
1829	2613	0.79	1.86	6.49	15.13	2.42	94% |±5/2>
1908	2726	2.82	7.43	8.79	87.96	1.09	76% |±3/2> + 17% |∓1/2>
1977	2825	0.38	1.23	17.66	88.14	0.26	63% |±1/2> + 14% |∓1/2> + 11% |∓3/2> + 10% |±3/2>

**Each row corresponds to a Kramers doublet and gives the energy (in cm^{-1} and K), effective g-values in the principal, x, y and z directions (g_x, g_y and g_z), the angle (°) between the g_z value of the excited Kramers doublet and the ground Kramers doublet, the expectation value of the total angular momentum along the z-direction of the ground Kramers doublet ($\langle J_z \rangle$) and the wavefunction composition. All data is directly from the CASSCF-SO calculation, except for the wavefunction, which is obtained by projection onto a model space of the $^6H_{15/2}$ term.*

6.2 Spin-phonon calculations

Table S11. Selected distances (Å) and angles (°) for the cations of **1-Dy**, **2-Dy**, and **[Dy{N(Si^{i}Pr$_3$)$_2$}(Cp*)]$^+$** at the periodic DFT optimised geometry, and crystal structure geometry (XRD). For **1-Dy**, the DFT geometry is a model using the packing of **2-Dy**, whose geometry is the closest match with **1-Dy**[a] (see methodology).

	[Dy{N(Si^{i}Pr$_3$)$_2$}(Cp*)]$^+$		1-Dy[a]		1-Dy[b]	2-Dy	
	XRD	DFT	XRD	DFT	XRD	XRD	DFT
Dy–N / Å	2.192(3),	2.196	2.185(3)	2.212	2.209(3)	2.204(3)	2.212
Dy–$Cp_{centroid}$ / Å	2.314(14)	2.322	2.345(2)	2.346	2.339(2)	2.333(2)	2.336
N–Dy–$Cp_{centroid}$ / °	152.5(3)	149.1	157.84(11)	158.9	167.90(10)	172.26(10)	170.9
Si–N–Si / °	143.0(2)	144.0	138.2(2)	141.1	141.6(2)	143.9(2)	145.7

[a] *Z*' = 2, cation A; [b] *Z*' = 2, cation B.

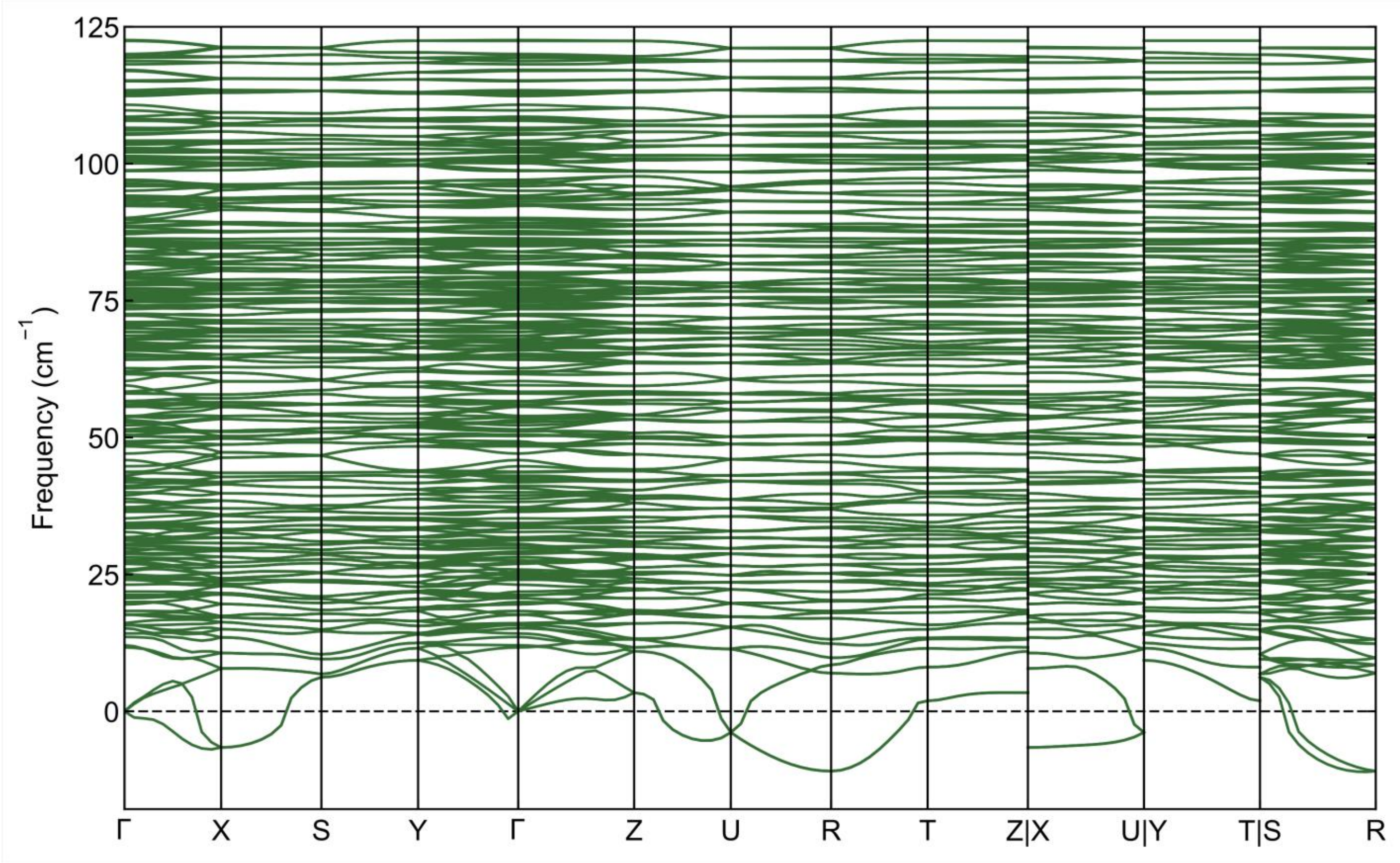

Figure S86. DFT-calculated low-energy phonon dispersion plot of the model of **1-Dy**.

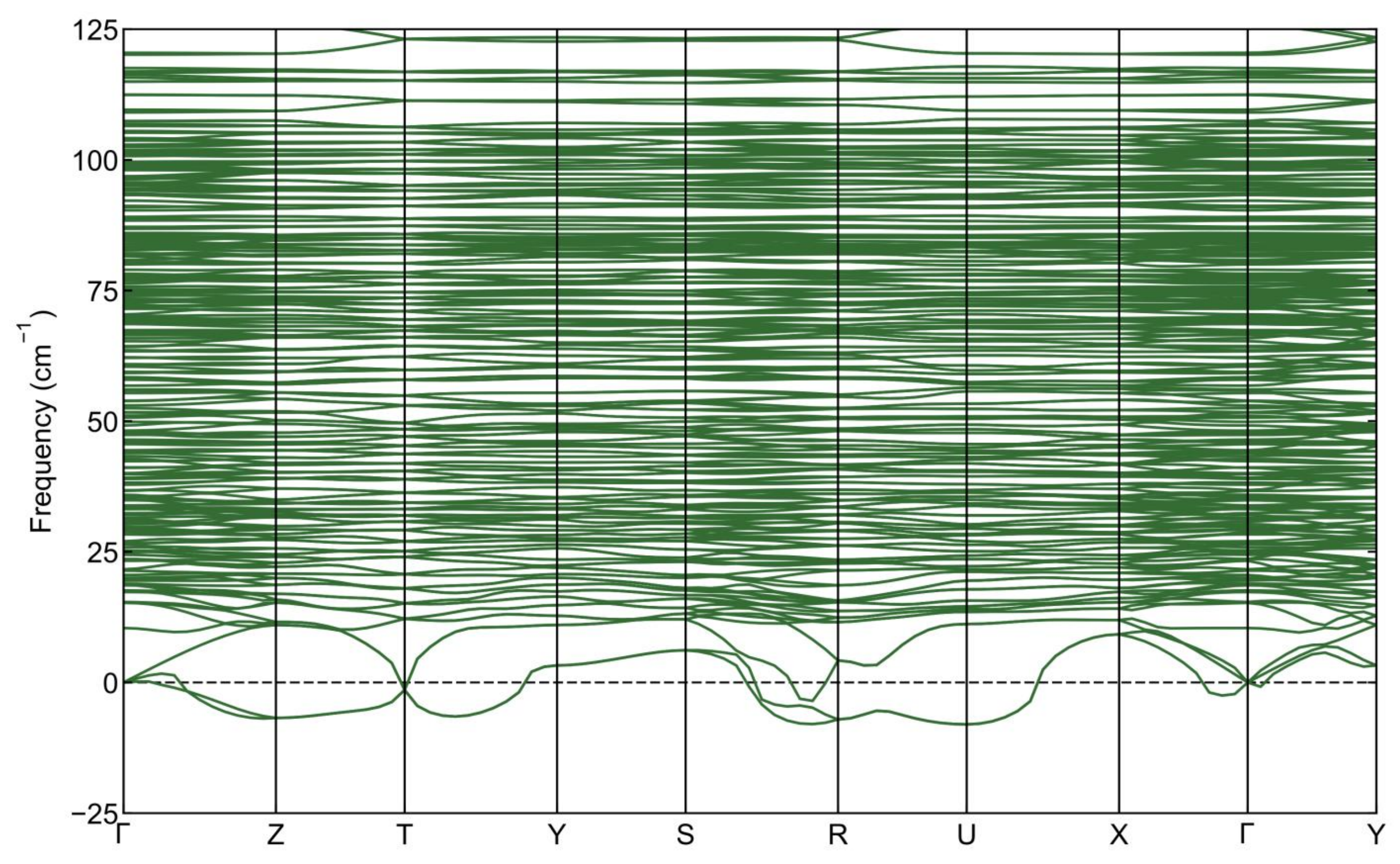

Figure S87. DFT-calculated low-energy phonon dispersion plot of **2-Dy**.

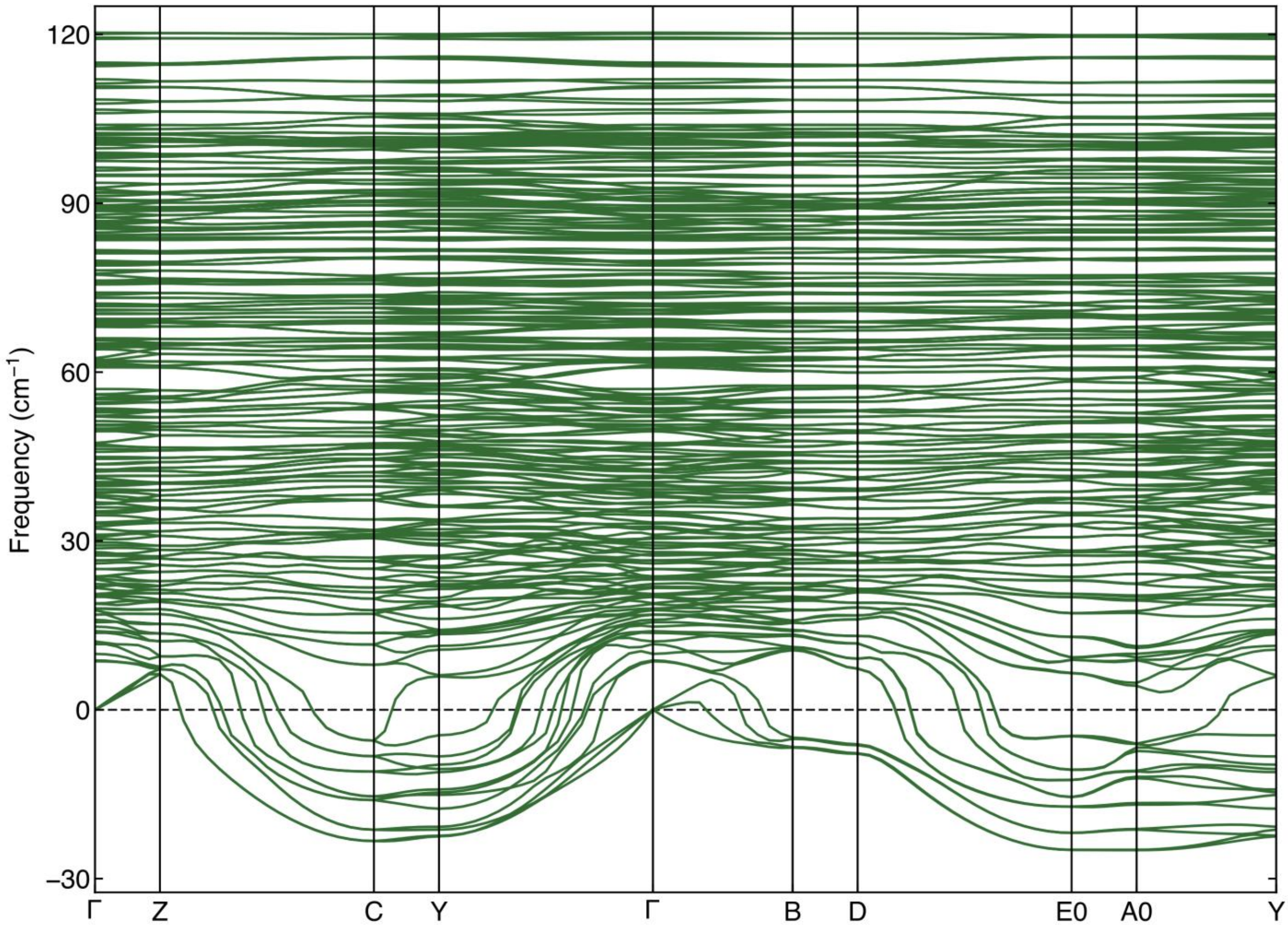

Figure S88. DFT-calculated low-energy phonon dispersion plot of **[Dy{N(Si^{i}Pr$_3$)$_2$}(Cp*)]$^+$**.

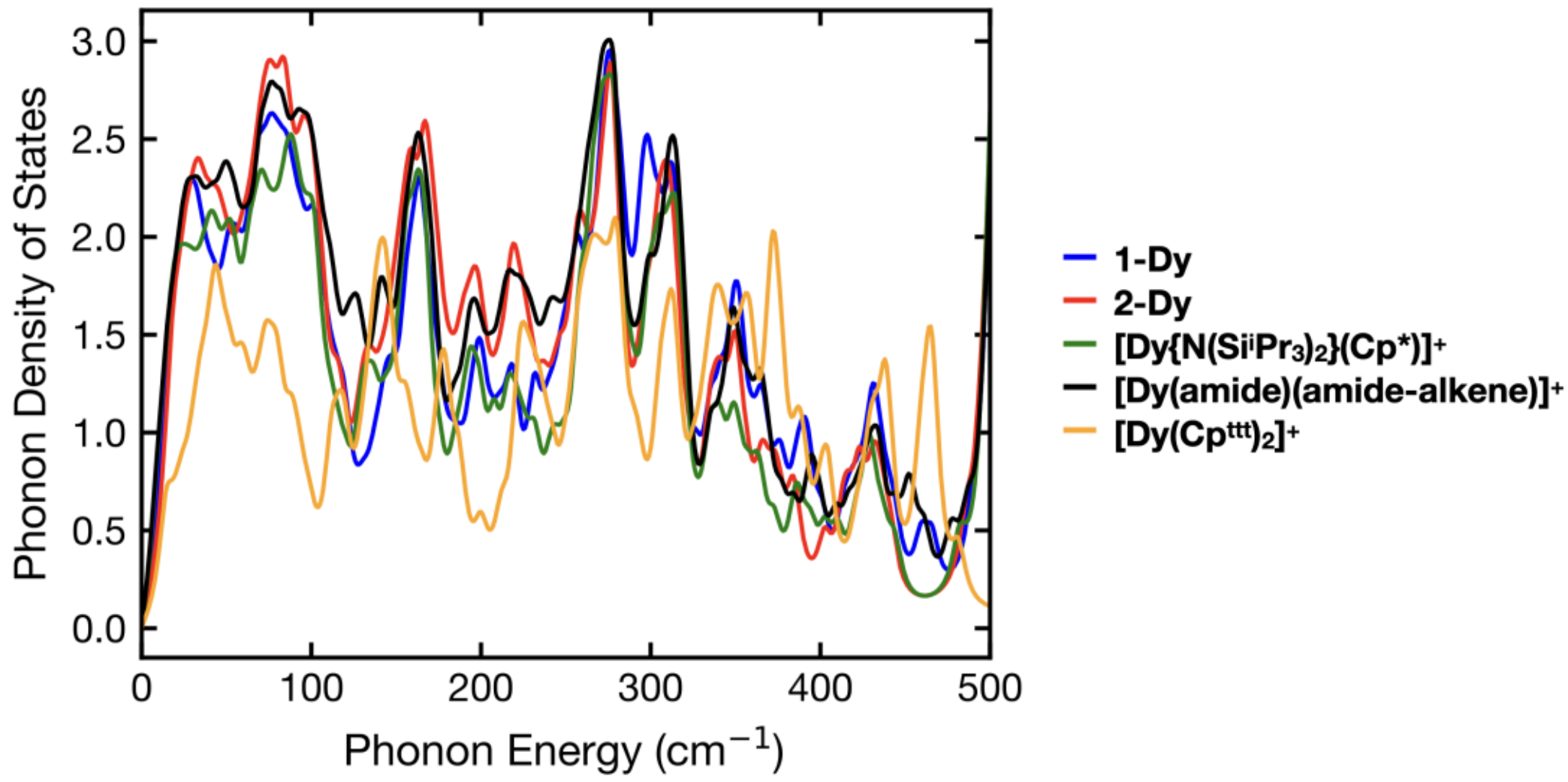

Figure S89. DFT-calculated phonon density of states (DoS) of the **1-Dy** model (blue), **2-Dy** (red), **[Dy{N(Si^{i}Pr$_3$)$_2$}(Cp*)]$^+$** (green), **[Dy(amide)(amide-alkene)]$^+$** (black) and **[Dy(Cp^{ttt})$_2$]$^+$** (yellow). Modes are broadened by anti-Lorentzian functions with a linewidth of 10 cm^{-1} and sampled on a 1×1×1 *q*-mesh. The DoS of **[Dy(Cp^{ttt})$_2$]$^+$** is doubled to account for the smaller unit cell (Z = 2) compared to the other four systems (Z = 4).

Table S12. SA-CASSCF-SO-calculated energy levels of the $^6H_{15/2}$ multiplet for **1-Dy** at the DFT optimised geometry.*

Energy / cm^{-1}	Energy / K	g_x	g_y	g_z	Angle (°)	<J_z>	Wavefunction composition
0	0	0.00	0.00	20.00	0.00	7.49	100% |±15/2>
547	781	0.00	0.00	17.03	3.73	6.48	99% |±13/2>
979	1399	0.01	0.01	14.24	2.83	5.49	98% |±11/2>
1296	1852	0.04	0.06	11.77	8.23	4.50	98% |±9/2>
1498	2140	0.93	1.14	9.97	27.43	3.42	93% |±7/2> + 6% |±5/2>
1629	2327	0.21	2.09	7.58	38.17	2.30	73% |±5/2> + 20% |±3/2> + 5% |±7/2>
1717	2453	3.20	5.62	6.79	88.58	1.24	45% |±3/2> + 35% |±1/2> + 16% |±5/2>
1782	2546	0.80	3.84	15.87	81.88	0.90	58% |±1/2> + 34% |±3/2>

Table S13. SA-CASSCF-SO-calculated energy levels of the $^6H_{15/2}$ multiplet for **2-Dy** at the DFT optimised geometry.*

Energy / cm^{-1}	Energy / K	g_x	g_y	g_z	Angle (°)	<J_z>	Wavefunction composition
0	0	0.00	0.00	20.00	0.00	7.50	100% |±15/2>
591	845	0.00	0.00	16.99	1.83	6.49	100% |±13/2>
1036	1481	0.01	0.01	14.23	2.15	5.48	99% |±11/2>
1349	1927	0.10	0.11	11.62	3.61	4.48	98% |±9/2>
1556	2223	0.88	1.07	9.12	11.39	3.45	97% |±7/2>
1686	2409	2.06	3.58	6.32	22.29	2.28	87% |±5/2> + 7% |±1/2>
1757	2510	2.31	6.66	10.46	89.48	0.89	67% |±3/2> + 19% |∓1/2>
1842	2632	0.17	0.43	18.33	87.25	0.34	46% |±1/2> + 23% |∓1/2> + 18% |±3/2> + 8% |∓3/2>

Table S14. SA-CASSCF-SO-calculated energy levels of the $^6H_{15/2}$ multiplet for **$[Dy\{N(Si^iPr_3)_2\}(Cp^*)]^+$** at the DFT optimised geometry.*

Energy / cm^{-1}	Energy / K	g_x	g_y	g_z	Angle (°)	<J_z>	Wavefunction composition
0	0	0.00	0.00	19.99	0.00	7.49	100% |±15/2>
478	683	0.00	0.00	17.09	5.06	6.48	99% |±13/2>
891	1273	0.01	0.01	14.26	2.92	5.49	97% |±11/2>
1203	1718	0.14	0.17	11.84	10.70	4.49	97% |±9/2>
1377	1967	0.40	0.94	10.88	38.00	3.28	85% |±7/2> + 12% |±5/2>
1470	2100	2.38	4.41	8.47	57.87	1.93	45% |±5/2> + 39% |±3/2> + 7% |±7/2> + 6% |∓1/2>
1556	2223	2.49	4.16	10.15	81.02	1.12	48% |±1/2> + 22% |±5/2> + 14% |±3/2> + 7% |±7/2> + 5% |∓1/2>
1645	2350	0.50	2.25	17.06	78.80	1.26	44% |±3/2> + 34% |±1/2> + 17% |±5/2>

Each row corresponds to a Kramers doublet and gives the energy (in cm^{-1} and K), effective g-values in the principal, x, y and z directions (g_x, g_y and g_z), the angle (°) between the g_z value of the excited Kramers doublet and the ground Kramers doublet, the expectation value of the total angular momentum along the z-direction of the ground Kramers doublet (<J_z>) and the wavefunction composition. All data is directly from the CASSCF-SO calculation, except for the wavefunction, which is obtained by projection onto a model space of the $^6H_{15/2}$ term.

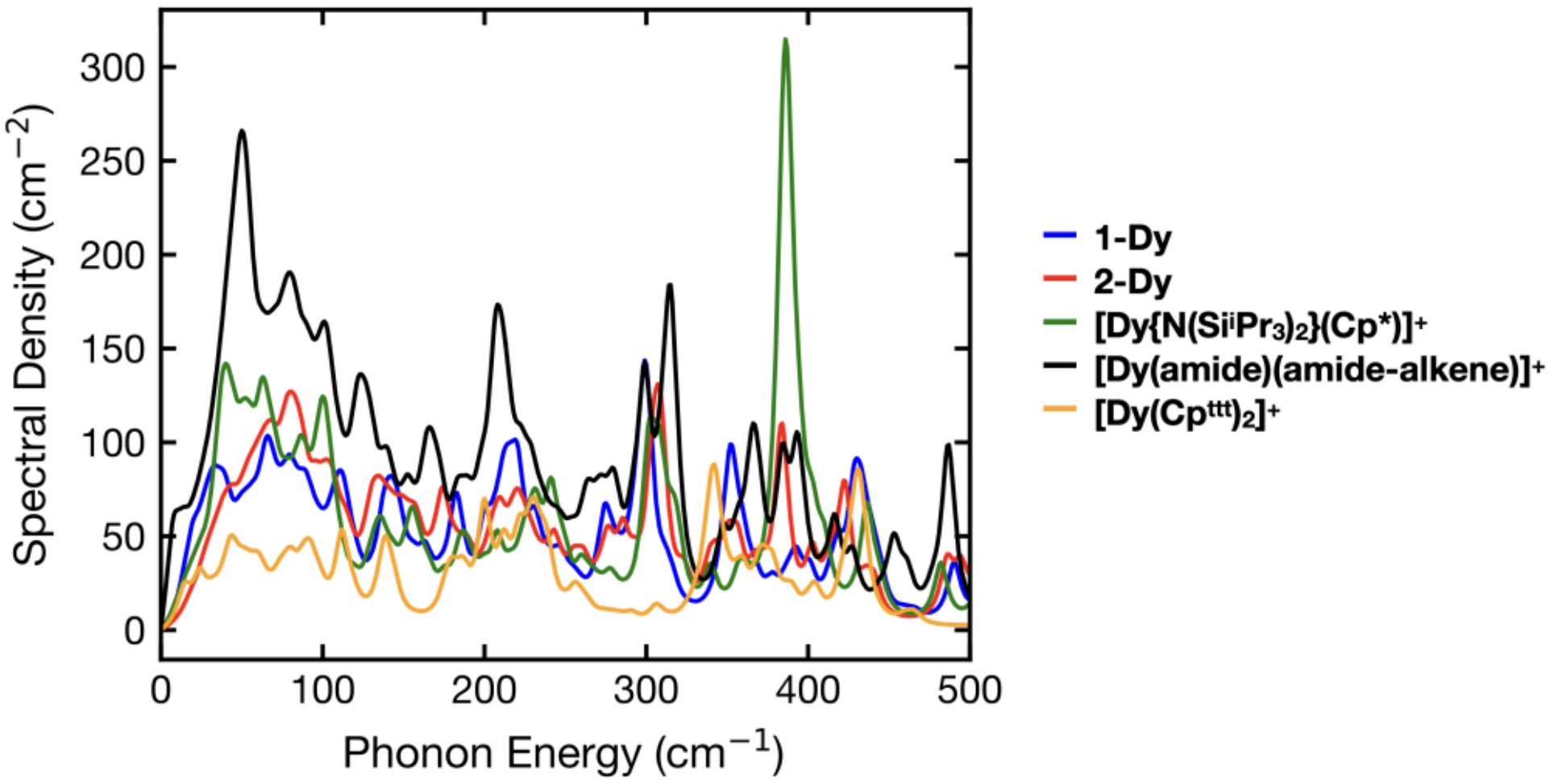

Figure S 90. One-phonon spectral density of the **1-Dy** model (blue), **2-Dy** (red), **[Dy{N(Si^{i}Pr$_3$)$_2$}(Cp*)]$^+$** (green), **[Dy(amide)(amide-alkene)]$^+$** (black) and **[Dy(Cp^{ttt})$_2$]$^+$** (yellow). The one phonon spectral density is the phonon DOS weighted by spin-phonon coupling strength, $J(\hbar\omega) = \sum_q \sum_{i,j} \left|\langle i|\hat{V}_q|j\rangle\right|^2 \rho_q(\hbar\omega)$. Modes are broadened by anti-Lorentzian functions with a linewidth of 10 cm^{-1} and sampled on a 1×1×1 *q*-mesh.

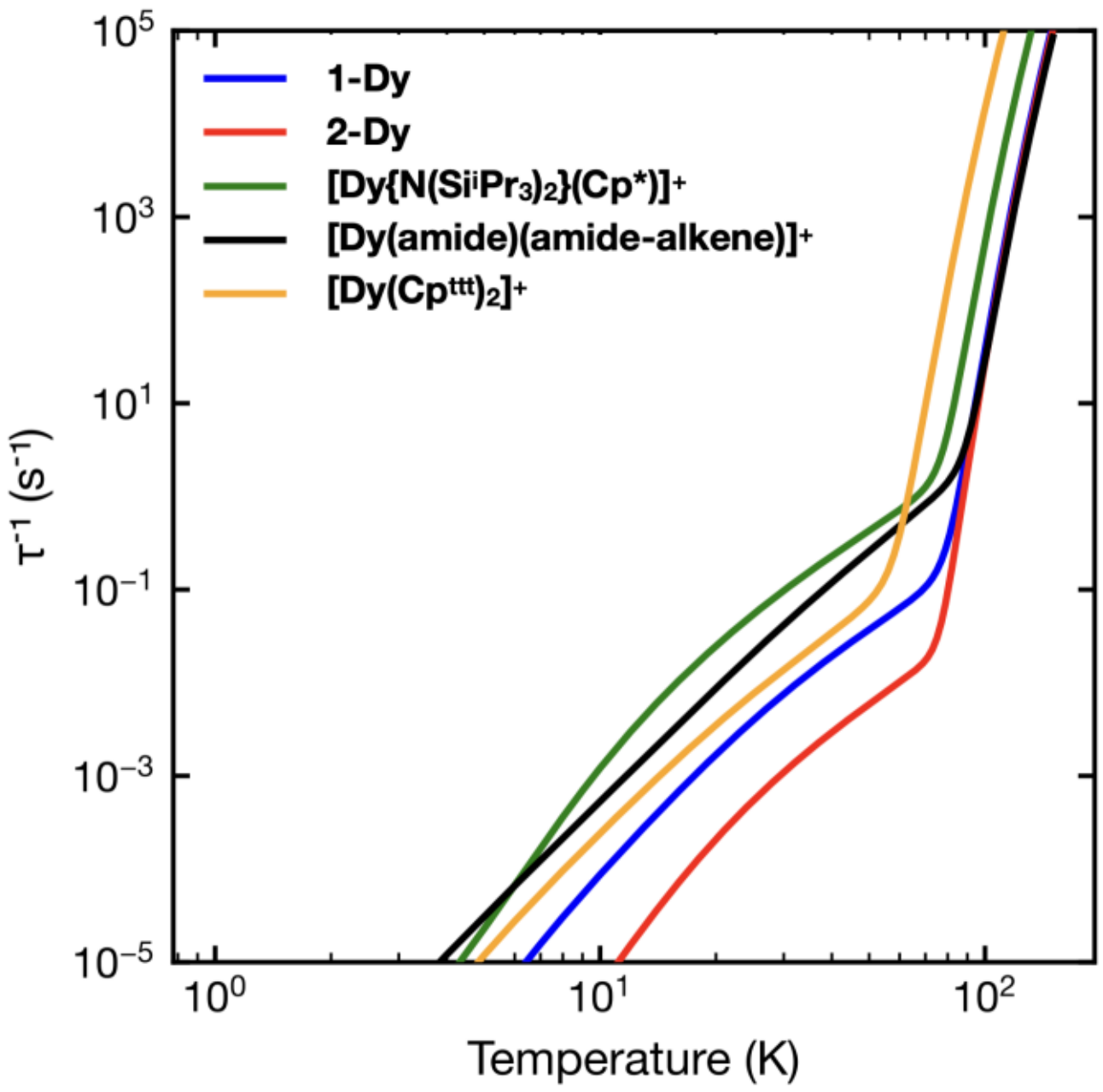

Figure S91. Calculated total (Orbach + Raman) magnetic relaxation rates of the **1-Dy** model (blue), **2-Dy** (red), **[Dy{N(Si^{i}Pr$_3$)$_2$}(Cp*)]$^+$** (green), **[Dy(amide)(amide-alkene)]$^+$** (black) and **[Dy(Cp^{ttt})$_2$]$^+$** (yellow). Rates calculated with phonons broadened by anti-Lorentzian functions with a linewidth of 10 cm^{-1} and sampled on a 1×1×1 *q*-mesh.

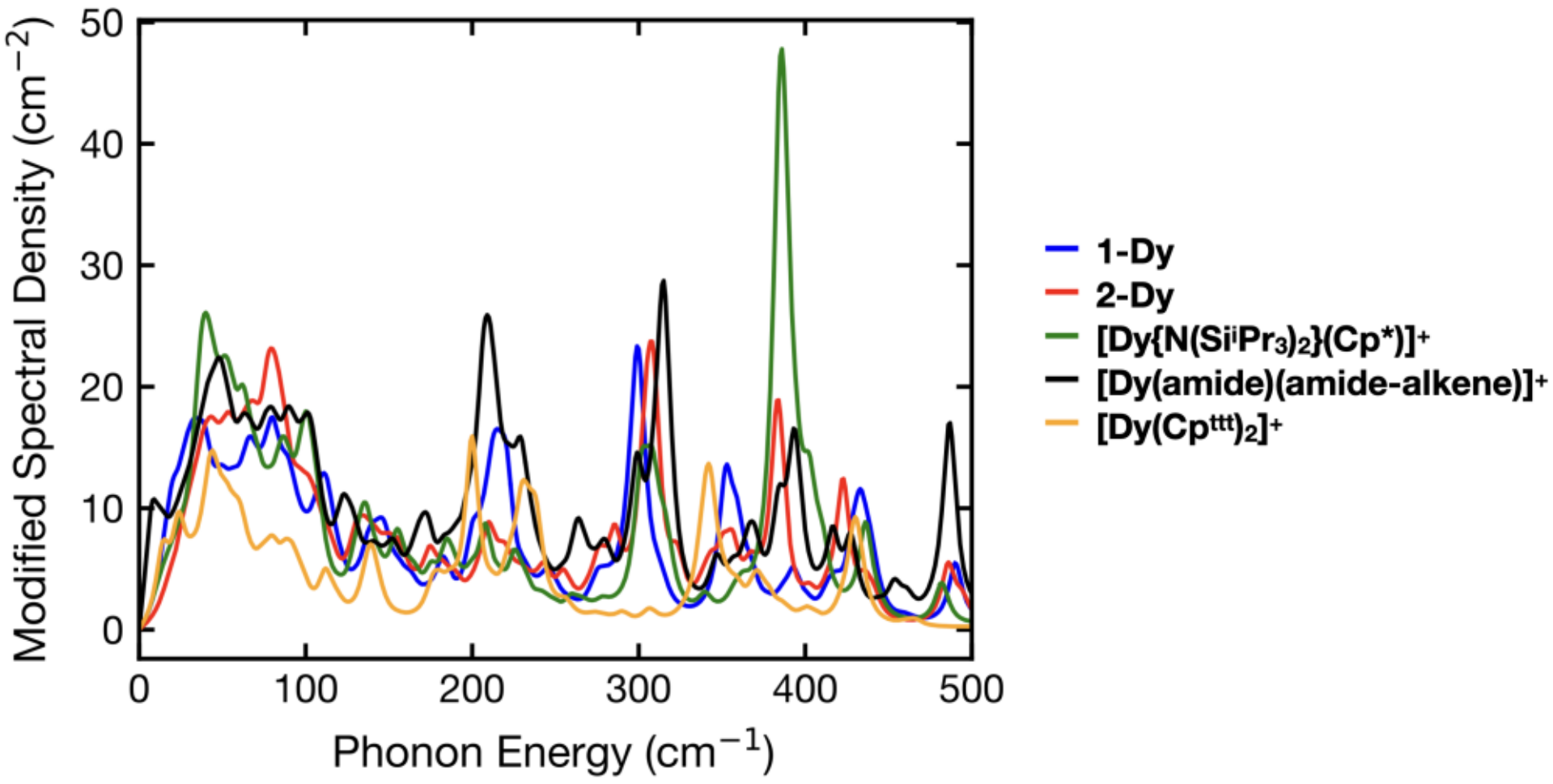

Figure S92. Truncated one-phonon spectral density of the **1-Dy** model (blue), **2-Dy** (red), **[Dy{N(Si^{i}Pr$_3$)$_2$}(Cp*)]$^+$** (green), **[Dy(amide)(amide-alkene)]$^+$** (black) and **[Dy(Cp^{ttt})$_2$]$^+$** (yellow). Here, the modified one-phonon spectral density only includes those matrix elements which contribute to Raman relaxation, *i.e.*:

$$J(\hbar\omega) = \sum_q \sum_{i,c} \left[\left| \langle i | \hat{V}_q | c \rangle \right|^2 + \left| \langle c | \hat{V}_q | i \rangle \right|^2 \right] \rho_q(\hbar\omega) \text{ where } i = \{0,1\} \text{ and } c = \{2..15\}$$

Modes are broadened by anti-Lorentzian functions with a linewidth of 10 cm^{-1} and sampled on a 1×1×1 *q*-mesh.

$$J_{fi}^{\pm\pm}(\hbar\omega_1, \hbar\omega_2) = \sum_{q,r} \left| \sum_c \left[\frac{\langle f | \hat{V}_q | c \rangle \langle c | \hat{V}_r | i \rangle}{E_c - E_i \pm_2 \hbar\omega_2} + \frac{\langle f | \hat{V}_r | c \rangle \langle c | \hat{V}_q | i \rangle}{E_c - E_i \pm_1 \hbar\omega_1} \right] \right|^2 \rho_q(\hbar\omega_1)\rho_r(\hbar\omega_2)$$

Equation S1. The Raman spectral density. $\pm_{1/2}$ denotes emission (-) or absorption (+) of phonon 1 or 2.

$$I_{fi}^{\pm\pm} = \frac{\pi}{\hbar} J_{fi}^{\pm\pm}(\hbar\omega_1)\bar{n}^{\pm 1}(\hbar\omega_1)\bar{n}^{\pm 2}(\hbar\omega_2)$$

Equation S2. The Raman rate integrand. $\bar{n}^{\pm 1}(\hbar\omega_1)$ is the Bose-Einstein phonon occupation factor.

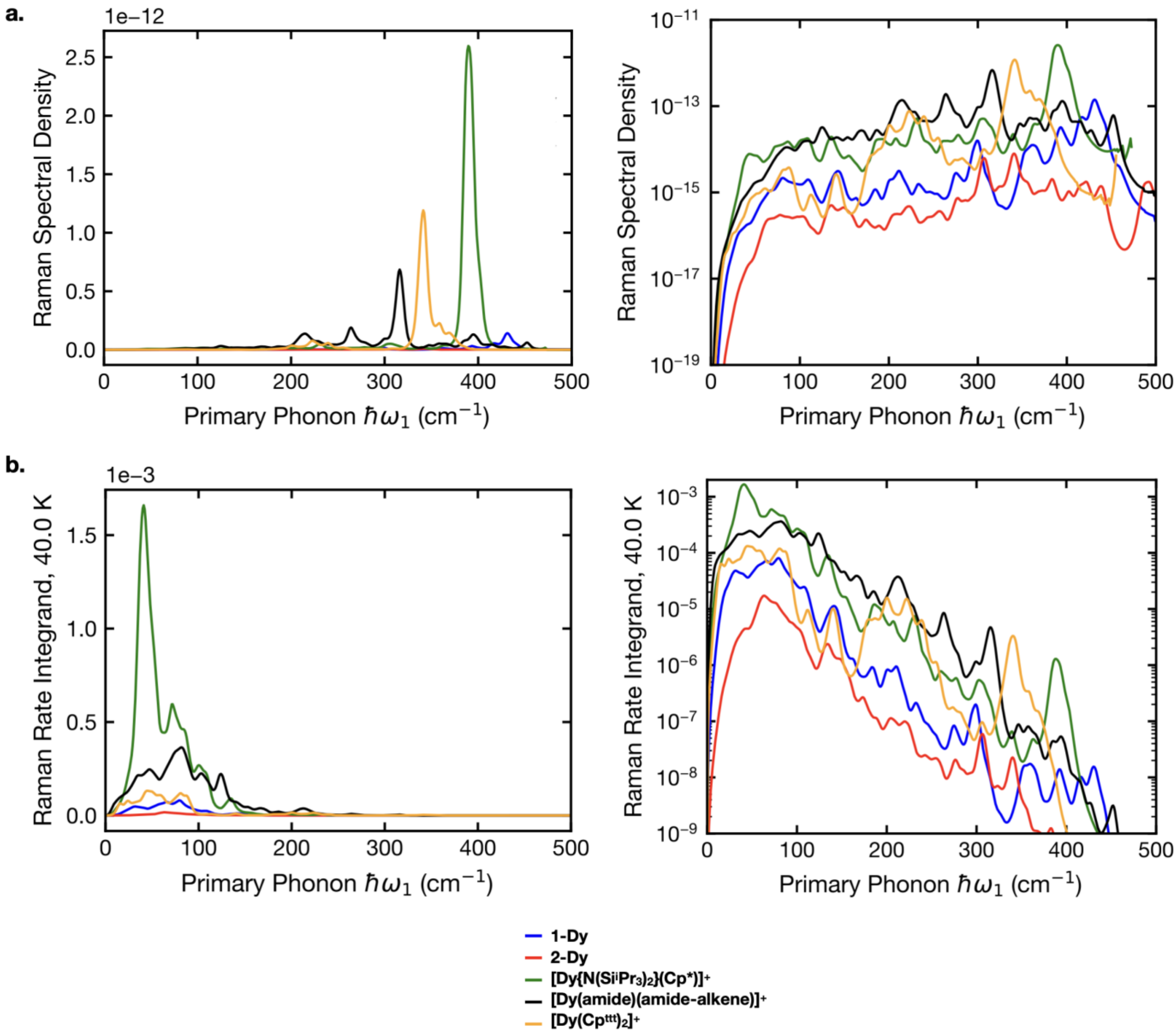

Figure S93. a. Raman spectral density and **b.** Raman rate integrand of the **1-Dy** model (blue), **2-Dy** (red), **[Dy{N(Si^{i}Pr$_3$)$_2$}(Cp*)]$^+$** (green), **[Dy(amide)(amide-alkene)]$^+$** (black) and **[Dy(Cp^{ttt})$_2$]$^+$** (yellow). Plotted on a linear y-axis (left), and logarithmic (right). Raman rates calculated with modes broadened by anti-Lorentzian functions with a linewidth of 10 cm^{-1} and sampled on a 1×1×1 *q*-mesh.

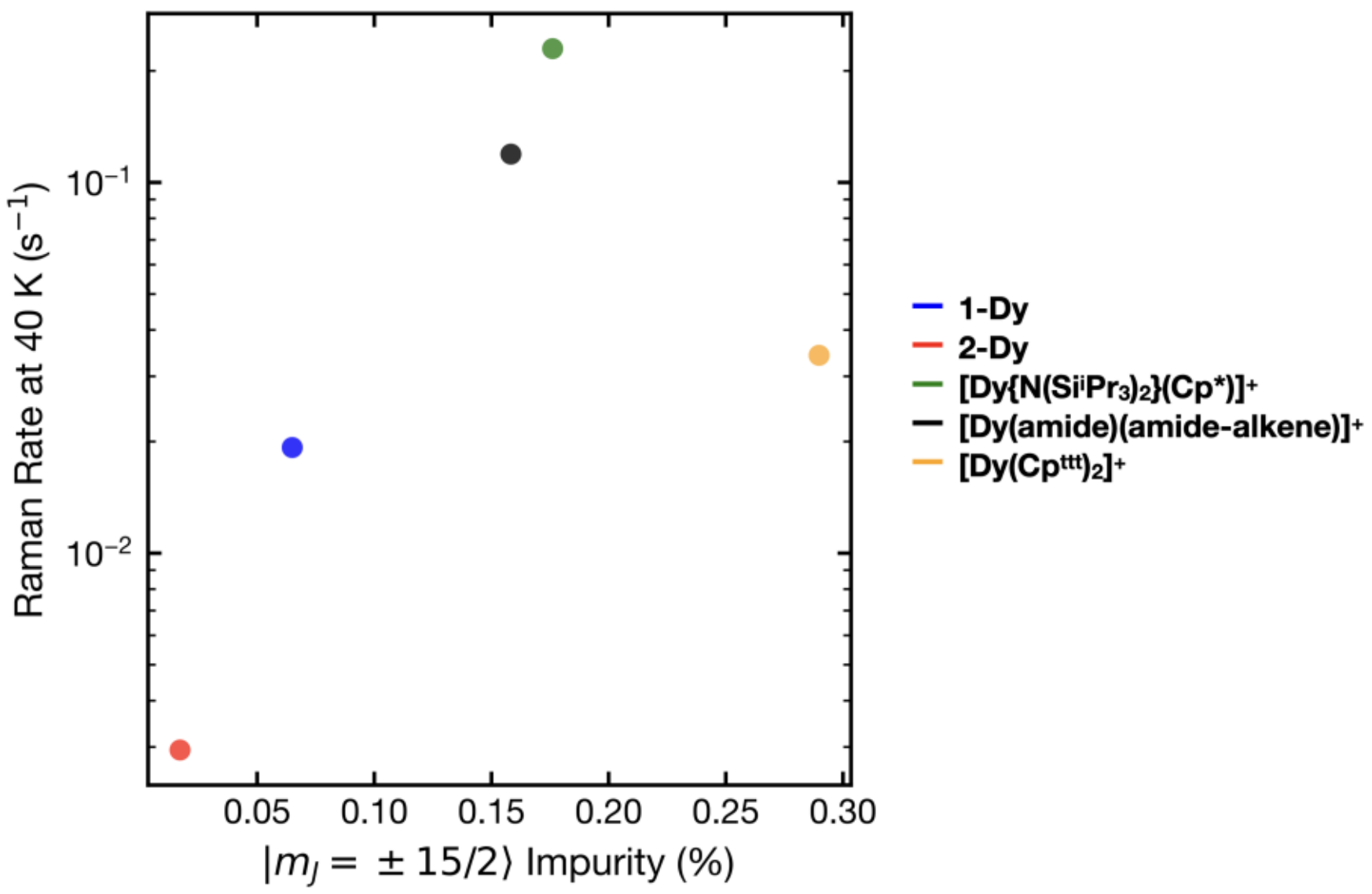

Figure S94. Raman rate at 40 K, vs. the impurity of the predominantly $\left|m_J = \pm\frac{15}{2}\right>$ ground state doublet (i.e. $\left|m_J \neq \pm\frac{15}{2}\right>$ character), for **1-Dy** model (blue), **2-Dy** (red), **[Dy{N(Si^{i}Pr$_3$)$_2$}(Cp*)]$^+$** (green), **[Dy(amide)(amide-alkene)]$^+$** (black) and **[Dy(Cp^{ttt})$_2$]$^+$** (yellow).

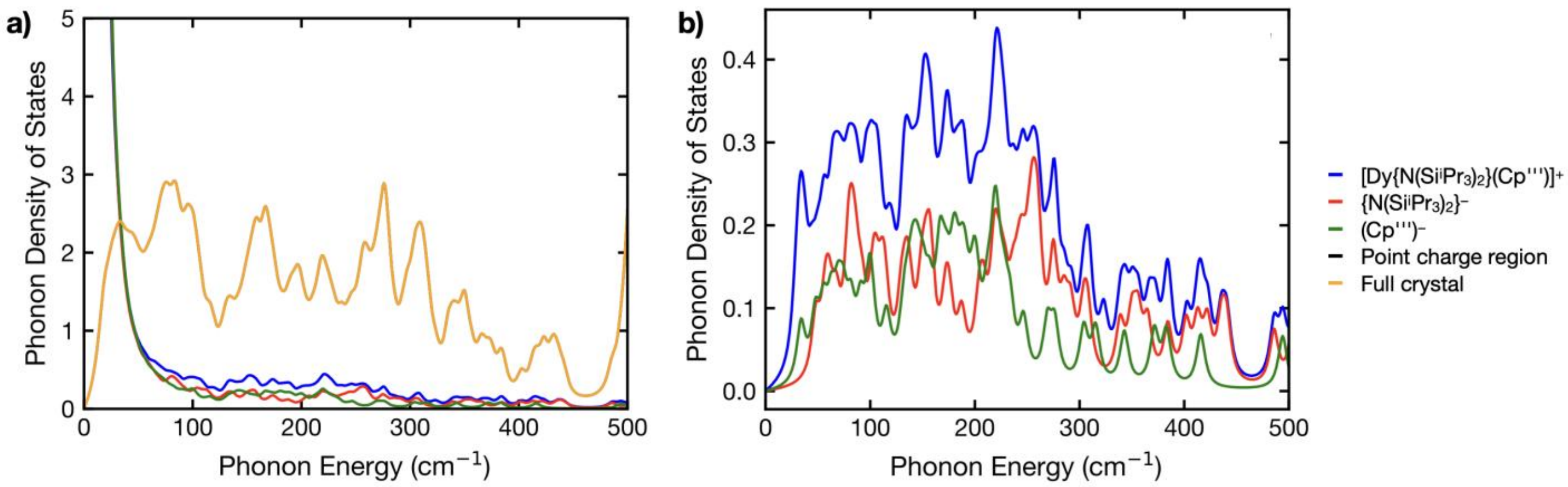

Figure S95. PHVA localized phonon DOS of **2-Dy.** Modes are broadened by anti-Lorentzian functions with a linewidth of 10 cm^{-1} and sampled on a 1×1×1 *q*-mesh, for the full [Dy{N(Si^{i}Pr$_3$)$_2$}(Cp''')]$^+$ cation (blue), the amide ligand (red), the Cp''' ligand (green), the point charge region (black) and the full crystal (yellow). Note the full crystal phonon DOS is indistinguishable from the point charge region, as no phonon localization is performed in this case. **a)** The full phonon DOS, **b)** the PHVA localized phonon DOS, excluding the low energy (< 0.1 cm^{-1}) frozen modes.

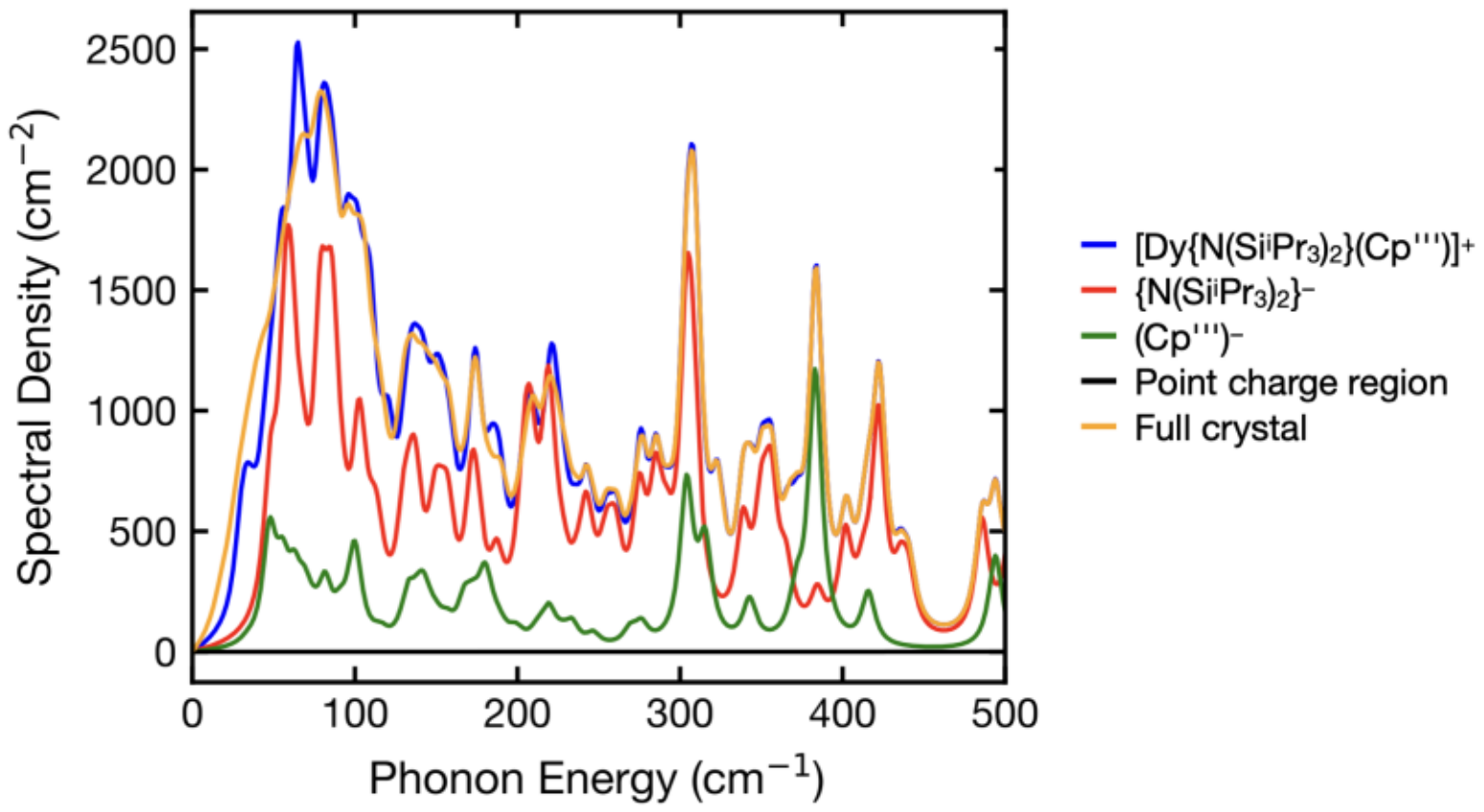

Figure S 96. PHVA localized one phonon spectral density of **2-Dy** for the full $[Dy\{N(Si^{i}Pr_3)_2\}(Cp''')]^+$ cation (blue), the amide ligand (red), the Cp''' ligand (green), the point charge region (black) and the full crystal (yellow).

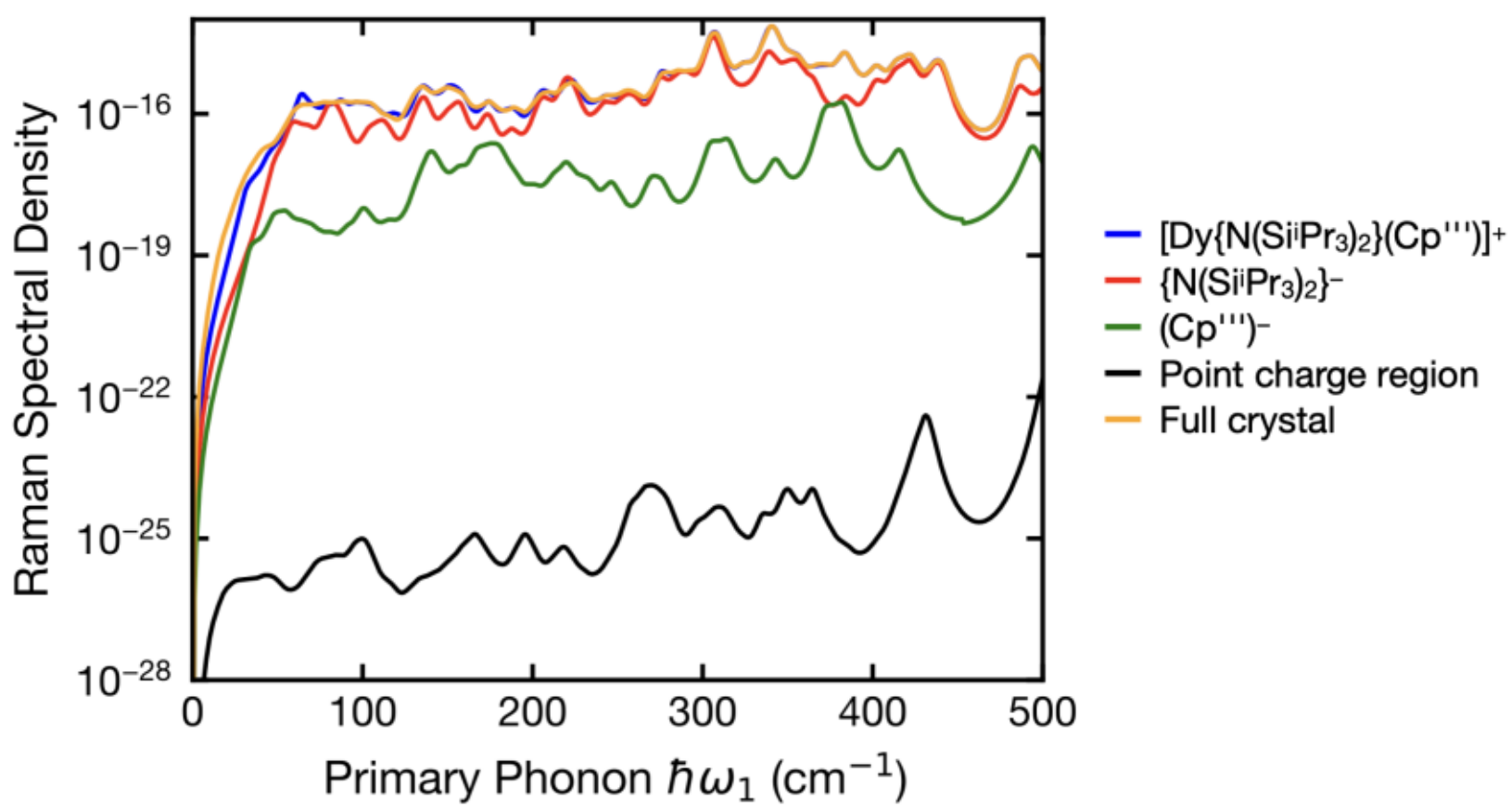

Figure S97. PHVA localized Raman spectral density of **2-Dy** for the full $[Dy\{N(Si^{i}Pr_3)_2\}(Cp''')]^+$ cation (blue), the amide ligand (red), the Cp''' ligand (green), the point charge region (black) and the full crystal (yellow).

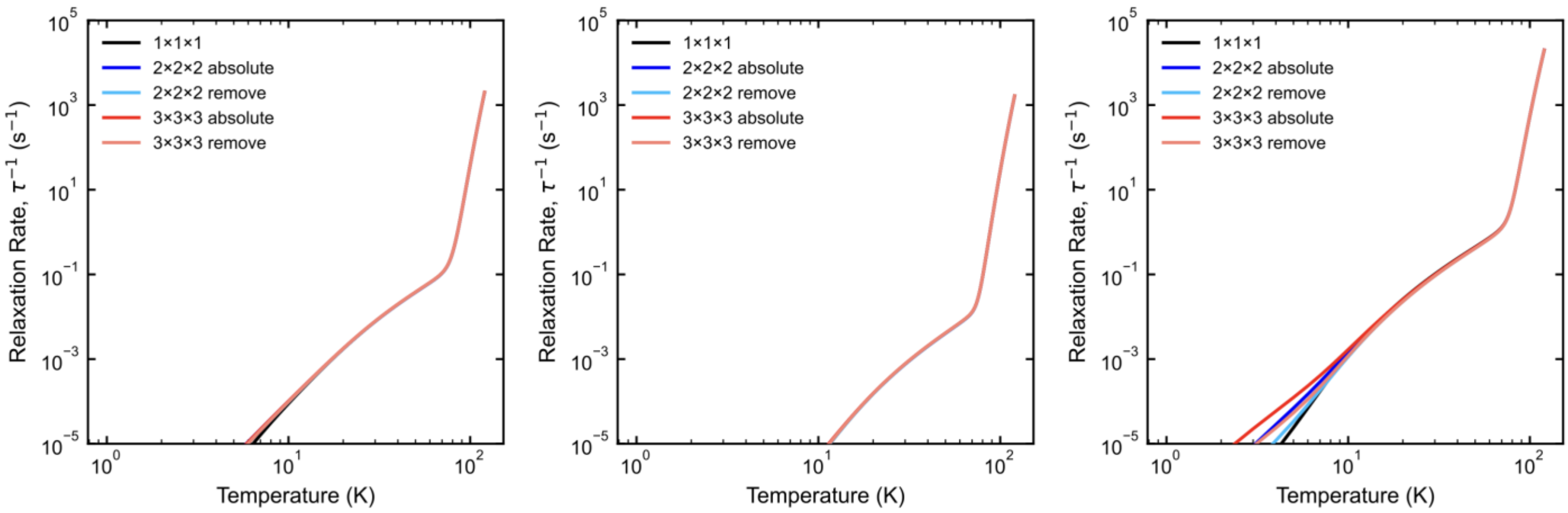

Figure S98. Total magnetic relaxation rates for different *q*-meshes, and both setting imaginary frequencies to their absolute value and removing them. **1-Dy** model (left), **2-Dy** (middle), and $\mathbf{[Dy\{N(Si^{i}Pr_3)_2\}(Cp^*)]^+}$ (right). No imaginary modes are present for the 1×1×1 *q*-mesh for any system.

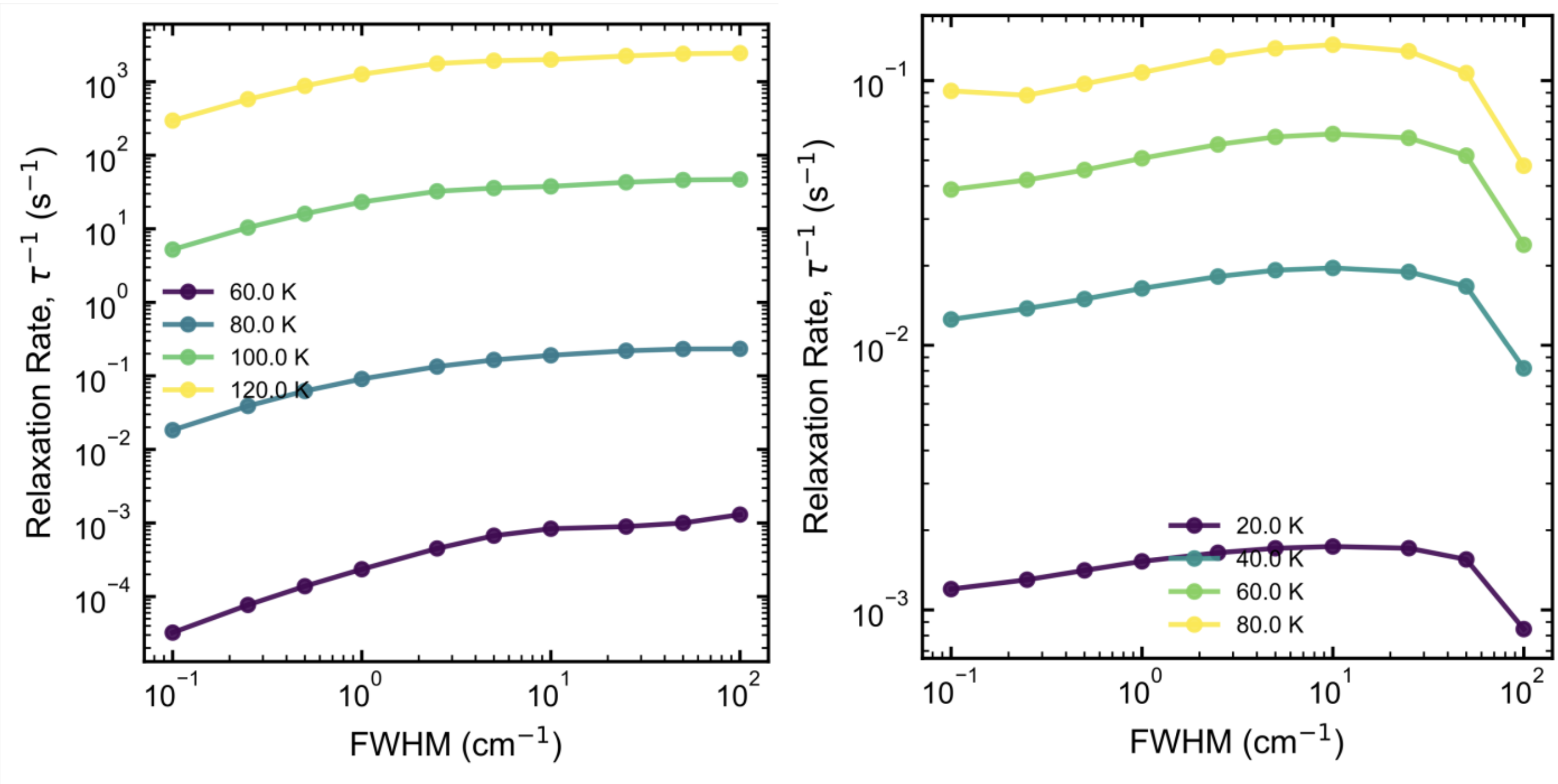

Figure S99. Magnetic relaxation rates of **1-Dy**, as a function of phonon lineshape FWHM. One-phonon relaxation (left) and two-phonon relaxation (right). Modes are broadened by anti-Lorentzian functions and sampled on a 1×1×1 *q*-mesh.

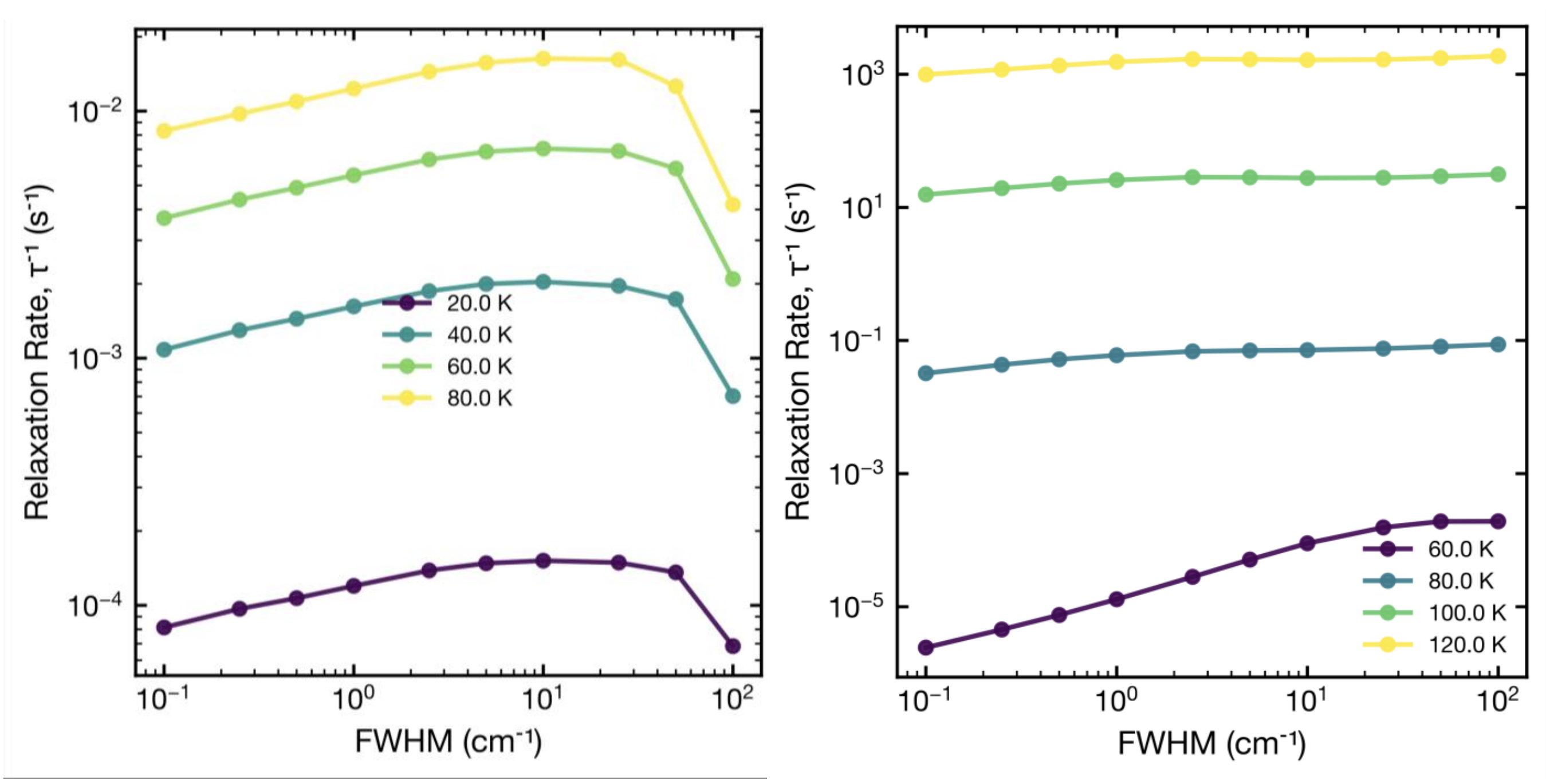

Figure S100. Magnetic relaxation rates of **2-Dy**, as a function of phonon lineshape FWHM. One-phonon relaxation (left) and two-phonon relaxation (right). Modes are broadened by anti-Lorentzian functions and sampled on a 1×1×1 *q*-mesh.

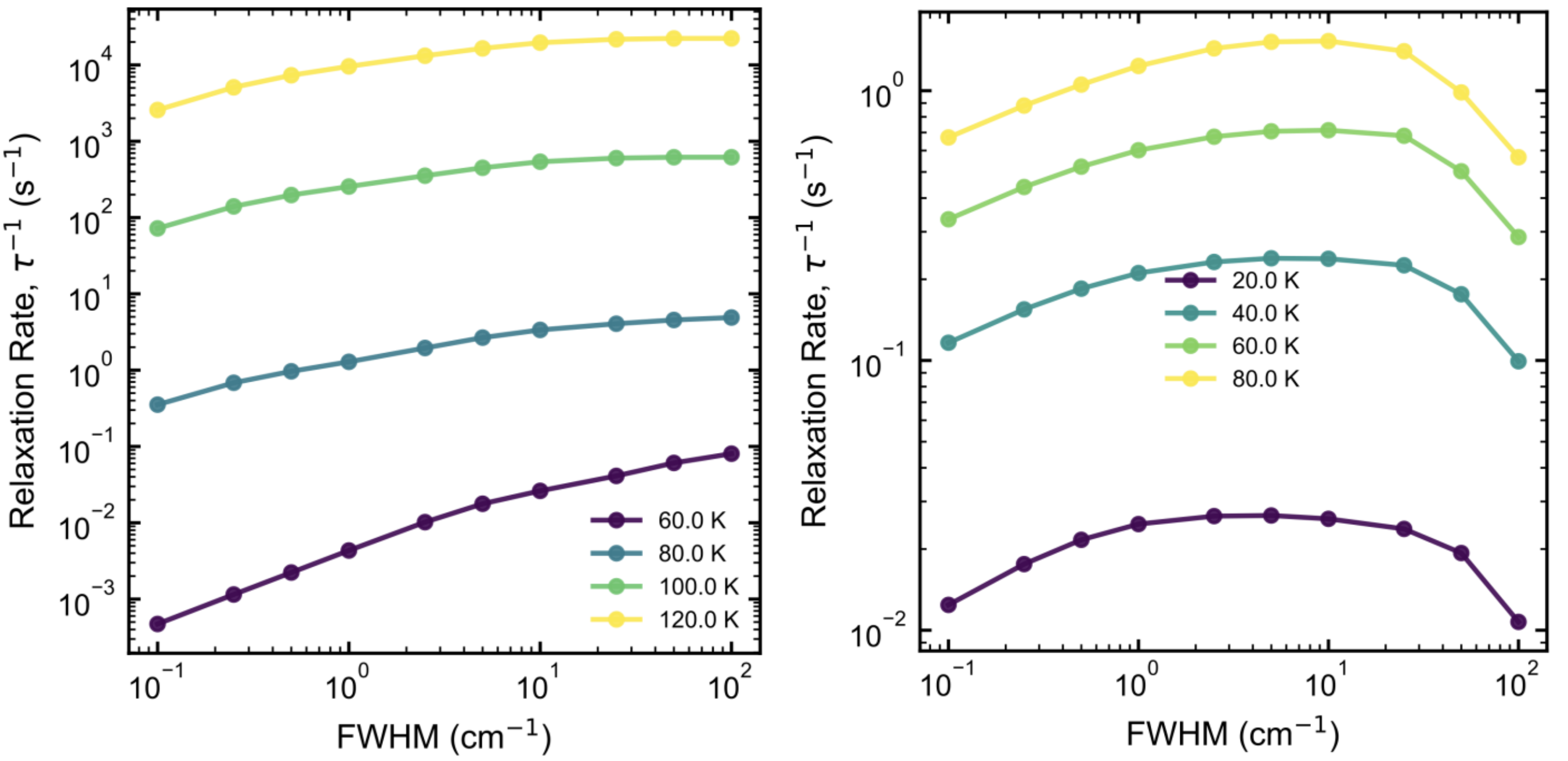

Figure S101. Magnetic relaxation rates of **$[Dy\{N(Si^iPr_3)_2\}(Cp^*)]^+$**, as a function of phonon lineshape FWHM. One-phonon relaxation (left) and two-phonon relaxation (right). Modes are broadened by anti-Lorentzian functions and sampled on a 1×1×1 *q*-mesh.

Fin
